# Structure–Activity Relationship Analysis and hERG Liability Assessment of Second‐Generation Antibiofilm Compounds Active Against *Salmonella*

**DOI:** 10.1002/cmdc.70363

**Published:** 2026-07-12

**Authors:** Amy Sorge, Aliyah N. Bennett, Sophia E. Gregory, Allysa L. Cole, Katherine J. Woolard, Ansley M. Nemeth, Andrew H. Crow, Roberta J. Melander, John S. Gunn, Christian Melander

**Affiliations:** ^1^ Department of Chemistry and Biochemistry University of Notre Dame Notre Dame Indiana USA; ^2^ Center for Microbe and Immunity Research Abigail Wexner Research Institute at Nationwide Children’s Hospital Columbus Ohio USA; ^3^ Infectious Diseases Institute The Ohio State University Columbus Ohio USA; ^4^ Department of Pediatrics College of Medicine The Ohio State University Columbus Ohio USA; ^5^ Department of Veterinary Biosciences The Ohio State University Columbus Ohio USA

**Keywords:** biofilm, biology, chronic infection, hERG, microbiology, *S* *almonella*, *S* *almonella enterica*, *S* *almonella* Typhi, serotype, typhoid fever

## Abstract

Typhoid fever is a life‐threatening illness caused by *Salmonella enterica* subspecies *enterica* serovar Typhi. The organ most implicated in chronic carriage and host transmission is the gallbladder due to bacterial biofilm formation on gallstones. Biofilms facilitate chronic infection because bacteria in biofilms are less susceptible to antibiotic treatment and immune factors in comparison to their planktonic brethren. Previously, we reported compounds that disrupt biofilms of *Salmonella enterica* serovar Typhimurium, a serovar closely related to *S*. Typhi, both in vitro and in vivo. This work extends the development of *Salmonella* antibiofilm agents through the further exploration of the parent scaffold. Lead antibiofilm compounds were tested for human ether‐a‐go‐go‐related gene (hERG) channel inhibition, from which we report new lead compounds with improved antibiofilm activity and reduced hERG channel inhibition.

## Graphical abstract

Typhoid fever, caused by *Salmonella enterica* serovar Typhi, infects millions of individuals per year. Chronic carriage of typhoid fever is associated with biofilm formation in the gallbladder. Herein, we identify novel antibiofilm agents active against *Salmonella enterica* serovar Typhimurium. **NDM‐47** exhibits improved biofilm inhibition and dispersion (activity with decreased hERG channel inhibition.

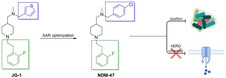

## 1 Introduction

*Salmonella enterica* serovar Typhi (*S*. Typhi) is the causative agent of typhoid fever [1], a systemic illness that can cause severe complications that include gastrointestinal bleeding, intestinal perforation, septic shock, and death [1, 2]. In 2017, there were over 10 million reported cases of typhoid fever, resulting in 136,000 deaths; however, these estimates are likely conservative due to limited documentation and the under‐reporting of infections [3, 4].

During acute typhoid infection, ingested pathogens that survive gastric transit can infect macrophages, which then translocate the bacteria to distal sites such as the spleen, bone marrow, pancreas, liver, and gallbladder [5, 7]. Once in the gallbladder, bile acid has a significant impact on gene regulation in *Salmonella,* and promotes biofilm formation on gallstones. A biofilm is an aggregate of cells that are attached to a surface and encased in matrix composed of self‐secreted extracellular polymeric substances (EPSs) [7, 9]. *S*. Typhi biofilm formation is strongly associated with the development of chronic carriage in infected patients. Even while asymptomatic, these individuals can be highly contagious for an extended period of time [7]. In areas with inadequate water sanitation, fecal shedding of the bacteria from chronic carriers is a critical source of water contamination and subsequent transmission to new hosts [10, 11].

Bacteria within a biofilm exhibit significantly higher tolerance to antibiotic treatment in comparison to planktonic cells, and may require higher antibiotic dosing and prolonged treatment courses [8]. Additionally, successful treatment of typhoid fever has become increasingly challenging due to the emergence of multidrug‐resistant (MDR) and extensively drug‐resistant (XDR) strains of *S*. Typhi [12]. XDR *S*. Typhi, defined as being resistant to first‐line antibiotics, fluoroquinolones, and third‐generation cephalosporins, was first reported in Pakistan in 2016, with cases rising to more than 10,000 as of August 2019 [12, 13]. Travel‐associated infections from Pakistan have been documented in Canada, Denmark, Australia, the United States, Germany, and the United Kingdom, establishing drug‐resistant *S*. Typhi as a critical global threat [12, 14–18].

**FIGURE 1:**
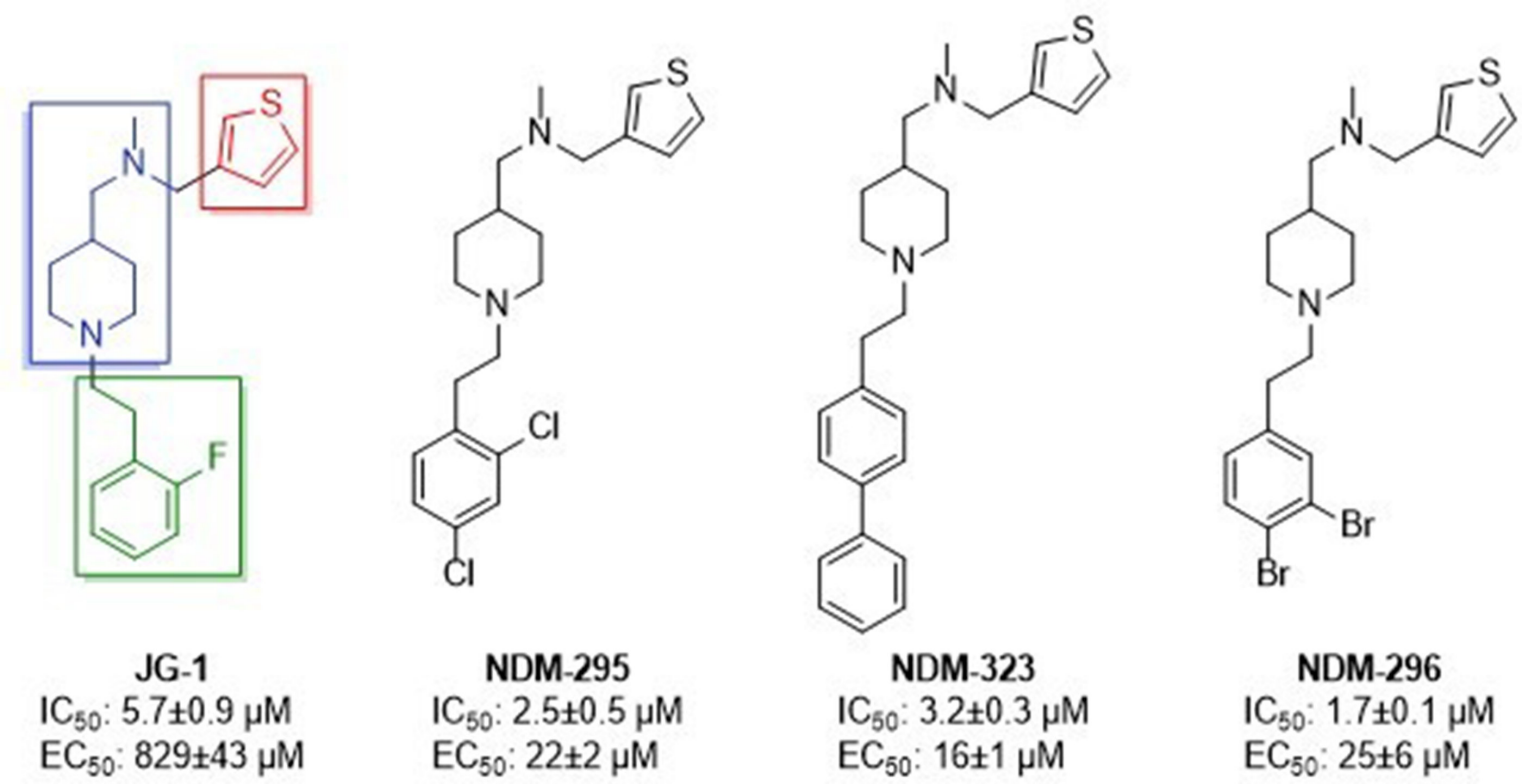
Structure of **JG‐1** showing tail region (green), core (blue), and head region (red) [9]. Three lead compounds that were tested in a murine model with their in vitro biofilm inhibition (IC_50_) and dispersion (EC_50_) data.

Due to the importance of biofilm formation in the *S.* Typhi infection cycle, targeting this phenotype with an antibiofilm agent is one potential way to increase antibiotic efficacy and eliminate chronic carriage. We previously reported **JG‐1** (Figure 1) as a lead compound that inhibits and disperses *S. enterica* serovar Typhimurium (a model organism for the human‐restricted *S*. Typhi) biofilms in vitro, and works synergistically with ciprofloxacin to clear *S*. Typhimurium infections in vivo in a murine chronic carriage model [9, 19]. A subsequent structure–activity relationship (SAR) study of **JG‐1** that focused on derivatizing the tail group (Figure 1) identified several analogs with improved antibiofilm activity compared to **JG‐1** both in vitro and in the chronic carriage model, with lead compounds **NDM‐323** and **NDM‐296** reducing the bacterial burden in the gallbladder 3.5–4 and 4.5–5 log‐fold respectively, when dosed at 5 mg/kg/day in combination with 1 mg/kg/day ciprofloxacin [9]. Herein, we examine the potential of **JG‐1** and these first‐generation leads for further development by evaluating their interaction with the human ether‐a‐go‐go‐related gene (hERG) channel, first through a patch clamp assay and then via a hERG fluorescence polarization assay. We report an additional SAR study on second‐generation analogs, in which the effect of changes to the thiophene head region of **JG‐1** on both in vitro *S*. Typhimurium antibiofilm activity and hERG inhibition were investigated. Derivatives that combine modifications from this study and our previously reported SAR study were then assembled and assayed for activity [9], ultimately identifying next‐generation leads with significantly reduced hERG channel binding.

## 2 Results and Discussion

To assess its suitability for further development, we evaluated the potential interaction between **JG‐1** and the hERG channel. Unwanted interaction with the hERG channel can cause cardiac arrhythmias, corresponding to prolongation of the QT interval, and is a major cause of drug attrition [20]. Utilizing a patch clamp assay [21], we uncovered that **JG‐1** inhibits the hERG channel by 93% at 30 µM. A higher throughput hERG fluorescence polarization assay, in which a redshift fluorescent tracer binds to the hERG channel and leads to polarization of light, which is reduced upon displacement by an inhibitor, was also utilized to provide a baseline value for comparison to novel analogs [22]. In this assay, **JG‐1** has an IC_50_ of 2.6 nM. Using the same fluorescence polarization assay, **NDM‐295**, **‐296**, and **‐323** returned hERG IC_50_s of 0.4 µM, 0.1 nM, and 1.0 nM respectively. Typically, the maximum acceptable hERG channel IC_50_ for drug candidate consideration is 10 μM; however, ≥40 μM provides a significantly increased safety margin [23]. Based upon this initial data, neither **JG‐1** nor any of our three lead compounds from our previous study are suitable for further advancement.

**SCHEME 1:**
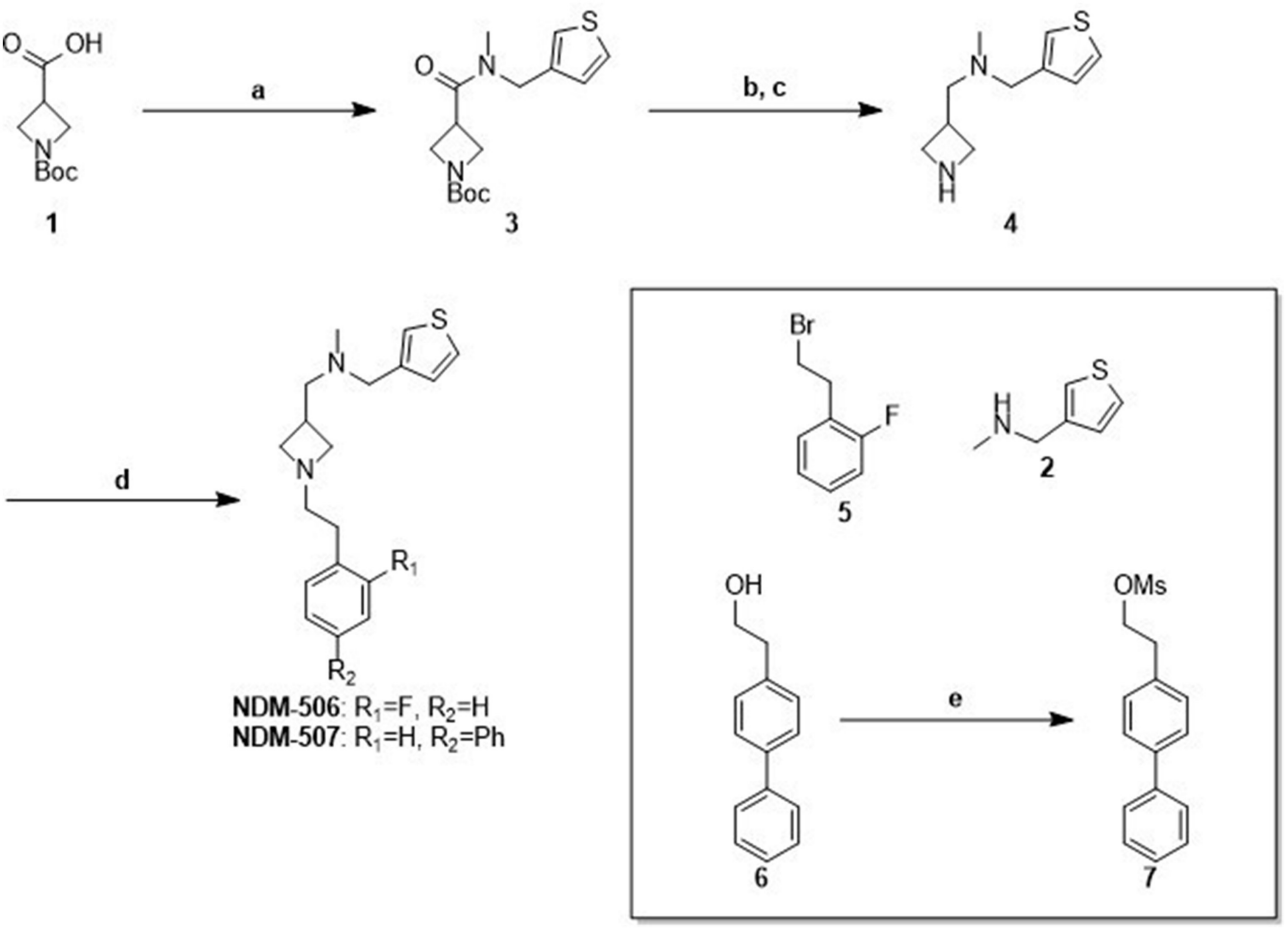
Synthesis of azetidine core compounds **NDM‐506** and **NDM‐507**. Reagents and conditions: (a) compound **2**, EDC, TEA, DMAP, DCM, rt, 24 h (87%); (b) TFA, DCM, rt, 3 h (98%); (c) LAH, THF 0°C–66°C, 4 h (40%); (d) compound **5** or **7**, K_2_CO_3_, MeCN, 82°C, 24 h (70%–80%); (e) MsCl, TEA, DCM, 0°C, 2 h (80%).

**SCHEME 2:**
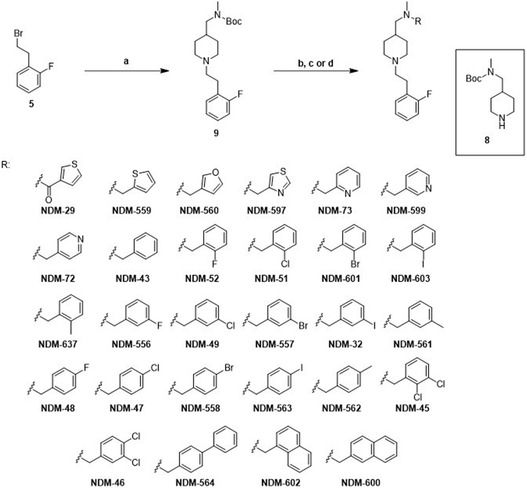
Synthesis of **JG‐1** and analogs. Reagents and conditions: (a) compound **8**, K_2_CO_3_, MeCN, reflux, O/N (60%); (b) TFA, DCM, rt, 3 h (98%); (c) RBr, K_2_CO_3_, MeCN, reflux, 1 h (20%–62%); (d) compound **11**, EDC, TEA, HOBt, DCM, rt, 24 h (62%).

Given the high hERG channel inhibition, we elected to perform additional analog synthesis to hopefully identify analogs that maintain antibiofilm activity while exhibiting reduced hERG inhibition. hERG channel binding is known to be driven by hydrophobicity and the presence of basic nitrogens that are positively charged at physiological pH [20]. **JG‐1**, **NDM‐295**, **‐296**, and **‐323** all contain two basic nitrogens; however, the molecules are fairly hydrophilic, having calculated cLogD_7.4_s <2. It has also been established that the piperidine moiety, which forms the core of our derivatives, can also drive hERG binding [24, 25, 26, 27]. Therefore, our first attempts to mitigate hERG activity involved exchanging the piperidine core with an azetidine ring (**NDM‐506** and **‐507**, Scheme 1) or replacing one of the basic nitrogens with an amide (**NDM‐29**, Scheme 2). The synthesis of the azetidine analogs is outlined in Scheme 1 and utilizes a modified approach to that used to synthesize **JG‐1** [9]. An EDC mediated coupling of carboxylic acid **1** with secondary amine **2** yields amide **3**, which was then Boc‐deprotected under acidic conditions before amide reduction to yield the tertiary amine **4**. Alkylation with 1‐(2‐bromoethyl)‐2‐fluorobenzene **5** or methanesulfonate **7** led to compounds **NDM‐506** and **NDM‐507**, respectively (Scheme 1). To access the amide derivative, a similar synthetic approach to that used in our original SAR study [9] was also utilized. Briefly, Boc‐protected aminopiperidine (**8**) was alkylated with 1‐(2‐bromoethyl)‐2‐fluorobenzene **5** to yield compound **9** (Scheme 2) [9]. Boc deprotection of **9** followed by an EDC‐mediated coupling with thiophene‐3‐carboxylic acid delivered the target amide **NDM‐29**.

The conditions utilized to determine IC_50_s and EC_50_s for **JG‐1** and all compounds reported here had to be altered slightly from the previously reported conditions to form a more standardized and reproducible protocol [9], leading to a different IC_50_ for **JG‐1** than previously reported. Under these new conditions, the incubation temperature was lowered from 30°C to 25°C, and the starting CFUs were lowered from a 1:100 dilution to a 1:2500 dilution. Previously, **JG‐1** was determined to have an IC_50_ of 5.7 ± 0.9 µM and an EC_50_ of 829 ± 43 µM (Figure 1) [9]; however, under these new conditions, **JG‐1** registered an IC_50_ of 52.4 µM (95% CI [41.6, 67.2]). The EC_50_ is >200 µM (highest concentration tested). However, given that JG‐1 is proven to have in vivo efficacy when dosed at 10 mg/kg [9], these new in vitro assays simply provide a more robust method to compare activity between analogs to allow identification of molecules with enhanced antibiofilm activity.

The three analogs (**NDM‐506**, **NDM‐507**, and **NDM‐29**) were then tested for inhibition and disruption of *S*. Typhimurium ATCC 14028 (JSG210) biofilms prior to hERG inhibition assessment. Inhibition of biofilm formation is assessed by growing bacteria in a 96‐well plate in the absence (control) or presence of test compounds. After incubation at 25°C for 24 h, the planktonic bacterial cells are removed through a washing step, and the remaining adhered bacterial structures (biofilm) are quantified with crystal violet (CV). Dose–response studies are used to calculate the concentration of compound necessary to elicit 50% inhibition of biofilm formation, which we define as the IC_50_. Biofilm dispersion is determined in a similar manner, except that biofilms are established for 24 h in the absence of compounds and then treated with compound for an additional 24 h. Wells are then washed, and the remaining biofilm is quantified with CV. Dose–response studies are employed to determine the concentration necessary to disperse 50% of the biofilm, defined as the EC_50_. These three compounds exhibited no biofilm inhibition or dispersion activity (>200 µM), and, as such, their hERG activities were not determined.

We next focused on analogs in which the aromatic head group was varied in an effort to overcome hERG liabilities while maintaining/enhancing antibiofilm activity. We began with replacing the thiophene head of **JG‐1** with alternative aromatic motifs, accessed via the same synthetic approach used to access **JG‐1** (Scheme 2). Boc deprotection of **9** was followed by alkylation with various commercially available alkyl bromides to deliver the library of **JG‐1** analogs depicted in Scheme 2.

Derivatives were initially screened for inhibition at 10 µM, and each analog was directly compared to **JG‐1** using a one‐way ANOVA with Dunnett’s multiple comparison test with a threshold of *p *< 0.1 (Table S1). Derivatives with *p *< 0.1 (14 of the 27 compounds in the first library) were then subjected to a dose–response assay from which in vitro IC_50_s and EC_50_s were determined (Table 1).

**TABLE 1:** Biofilm inhibition and dispersion data.

Compound	IC_50_, µM [95% CI]	EC_50_, µM [95% CI]
**JG‐1**	38.2 [32.1, 45.4]	>200
**NDM‐506**	>200	>200
**NDM‐507**	>200	>200
**NDM‐29**	>200	>200
**NDM‐559**	96.3 [86.1, 108]	>200
**NDM‐560**	95.2 [88.5, 103]	>200
**NDM‐599**	>200	>200
**NDM‐556**	27.9 [24.0, 32.5]	>200
**NDM‐557**	13.1 [11.5, 15.0]	186 [134, 257]
**NDM‐32**	9.8 [8.5, 11.3]	110 [94.2, 129]
**NDM‐561**	20.6 [17.3, 24.6]	>200
**NDM‐47**	10.6 [9.0, 12.4]	187 [148.6, 235]
**NDM‐558**	8.2 [7.6, 8.8]	>200
**NDM‐563**	6.6 [6.1, 7.2]	>200
**NDM‐562**	14.8 [12.9, 17.0]	>200
**NDM‐46**	7.0 [6.1, 7.9]	71.7 [48.4, 106]
**NDM‐564**	10.6 [10.1, 11.1]	138 [105, 181]
**NDM‐600**	4.0 [3.5, 4.6]	>200

*Note:* IC_50_ and EC_50_s for compounds that passed initial screening.

The thiophene ring was replaced with a series of heterocycles (furan, thiazole, and pyridines) to investigate the impact of hetercycle composition on antibiofilm activity. Modifying the electronic properties, hydrogen‐bonding potential, ring size, and heteroatom orientation may alter interactions with the biological target [28, 29]. No analog with an alternative heterocycle to 3‐thiophene (**NDM‐559**, **NDM‐560**, and **NDM‐599**) has improved biofilm inhibition. Analogs **NDM‐559** and **NDM‐560**, which harbor a 2‐thiophene and a 3‐furan, respectively, exhibit decreased biofilm inhibition (IC_50_ = 96.3 µM (95% CI [86.1, 108]) and 95.2 µM (95% CI [88.5, 103]), respectively) compared to **JG‐1**. The 3‐pyridine analog **NDM‐599** was inactive, with both IC_50_ and EC_50_ values >200 µM.

The thiophene ring was subsequently replaced with a phenyl ring, removing the heteroatom while maintaining an aromatic hydrophobic scaffold. This modification enabled a systematic evaluation of the electronic and steric effects of substituents on antibiofilm activity. Compounds with monosubstitution at the 3‐ or 4‐position, **NDM‐556** (3‐F), **NDM‐557** (3‐Br), **NDM‐32** (3‐I), **NDM‐561** (3‐Me), **NDM‐47** (4‐Cl), **NDM‐558** (4‐Br), **NDM‐563** (4‐I), **NDM‐562** (4‐Me), and **NDM‐564** (4‐Ph), exhibit an overall increased biofilm inhibition activity (Table 1). Biofilm activity is further increased when there is a disubstitution pattern at the 3‐ and 4‐positions, with the 3,4‐dichloro analog (**NDM‐46**) being the most potent, returning an IC_50_ of 7.0 µM (95% CI [6.1, 7.9]) and an EC_50_ of 71.7 µM (95% CI [48.4, 106]).

While these activities are promising, we wanted to assess if the decreased biofilm mass is due to specific antibiofilm effects, as predicted, or effects on cell viability. Biofilms were grown as above, but prior to the washing step, the optical density at 600 nm (OD_600 __nm_) was measured to quantify planktonic cell density. If decreased planktonic cell growth was observed along with decreased biofilm inhibition or dispersion, further testing was conducted to determine if compounds act by decreasing metabolic activity and/or overall viability of the bacteria. A bioluminescent strain of *Salmonella* Typhimurium (JSG 4922) was incubated with 10–40 µM compound for 16 h in a heated spectrophotometer to determine metabolic activity and further quantify the impact on growth. This previously described isolate requires ATP for light production, and therefore, quantification of bioluminescence can be used to track changes in gross metabolic activity [30]. Relative luminescence and OD_600 __nm_ of JSG 4922 were measured hourly and compared to bacteria incubated without compound to assess changes in cellular density and metabolic activity (Figure S1). Toxicity was defined as inhibition of cellular metabolic activity or stasis of cell density at concentrations >10 µM. Only compounds **NDM‐563** and **NDM‐564**, which have phenyl ring modifications harboring large substituents (iodine and phenyl, respectively) in the 4‐position, inhibit cellular growth and ATP production.

Select compounds were next tested in the hERG fluorescence polarization assay. Due to expense of the assay and instrumentation access, all hERG assays (as well as those delineated below) were performed in duplicate as a means to simply rank order compounds for future testing using the gold standard patch‐clamp assay. The hERG inhibition activity of **NDM‐51** was found to be essentially equipotent to that of **JG‐1** (Table 2). Additionally, **NDM‐32**, **NDM‐47**, and **NDM‐46** demonstrate the largest decrease in hERG inhibition compared to **JG‐1**, with **NDM‐47** (3‐chlorophenyl) showing the greatest reduction in inhibition, with an IC_50_ of 53.6 µM, thus establishing that we could potentially reduce hERG channel activity through modification of the head group while increasing antibiofilm activity (Figure S2).

**TABLE 2:** IC_50_s from the hERG fluorescence polarization assay.

Compound	IC_50_, µM
**JG‐1**	0.00266
**NDM‐51**	0.00293
**NDM‐556**	0.0265
**NDM‐32**	12.5
**NDM‐561**	0.181
**NDM‐47**	53.6
**NDM‐563**	0.00225
**NDM‐46**	4.65
**NDM‐564**	0.0751
**NDM‐600**	0.00064

Our previously reported **JG‐1** SAR study focused on phenyl tail region modifications and uncovered **NDM‐295** (IC_50_ = 2.5 ± 0.5 µM, EC_50_ = 22 ± 2 µM) and **NDM‐323** (IC_50_ = 3.2 ± 0.3 µM, EC_50_ = 16 ± 1 µM) as potent antibiofilm compounds (both antibiofilm assays done under previous conditions) (Figure 1) [9]. Under the new biofilm protocol, compound **NDM‐295** returned increased IC_50_ and EC_50_s (as seen with **JG‐1**) to those previously reported (IC_50_ 7.83 µM (95% CI [7.12, 8.61]), EC_50_ 101.7 µM (95% CI [78.3, 132])), while **NDM‐323** exhibited bacterial toxicity; therefore, antibiofilm activity was not determined (Table 3). To further augment activity, we investigated if combining these improved tail modifications with replacements for the thiophene head groups uncovered in this study with varying electronics and sterics could deliver antibiofilm activity while also potentially reducing hERG activity.

**TABLE 3:** Biofilm inhibition and dispersion data for the second set of compounds.

Compound	IC_50_, µM [95% CI]	EC_50_, µM [95% CI]
**NDM‐295**	8.9 [8.3, 9.5]	101.7 [78.0, 132]
**NDM‐61**	7.3 [3.6, 14.6]	68.5 [52.0, 90.3]
**NDM‐589**	10.4 [9.3, 11.7]	172.5 [122, 244]
**NDM‐55**	ND	45.5 [30.2, 68.5]
**NDM‐36**	6.1 [5.7, 6.5]	37.5 [29.9, 47.0]
**NDM‐38**	8.8 [8.2, 9.4]	69.0 [49.9, 95.7]
**NDM‐35**	5.0 [4.8, 5.8]	50.0 [36.9, 67.4]
**NDM‐41**	3.2 [2.5, 4.1]	91.2 [48.6, 174]
**NDM‐210**	6.8 [6.1, 7.4]	66.5 [49.1, 90.1]
**NDM‐40**	ND	54.5 [37.6, 79.1]
**NDM‐592**	ND	65.2 [50.6, 83.9]
**NDM‐591**	3.6 [3.0, 4.2]	46.8 [32.0, 68.5]
**NDM‐606**	6.9 [5.9, 8.1]	137.2 [84.7, 222]
**NDM‐565**	7.4 [4.8, 11.4]	88.5 [64.5, 121]
**NDM‐594**	12.1 [10.3, 14.2]	180 [120, 268]
**NDM‐605**	ND	36.7 [21.5, 62.8]
**NDM‐593**	14.5 [11.1, 18.7]	160 [76.8, 334]

*Note:* IC_50_ and EC_50_s for compounds that passed the screening. All values are in micromolar.

Abbreviation: ND, no significant effect on biofilm inhibition.

**SCHEME 3:**
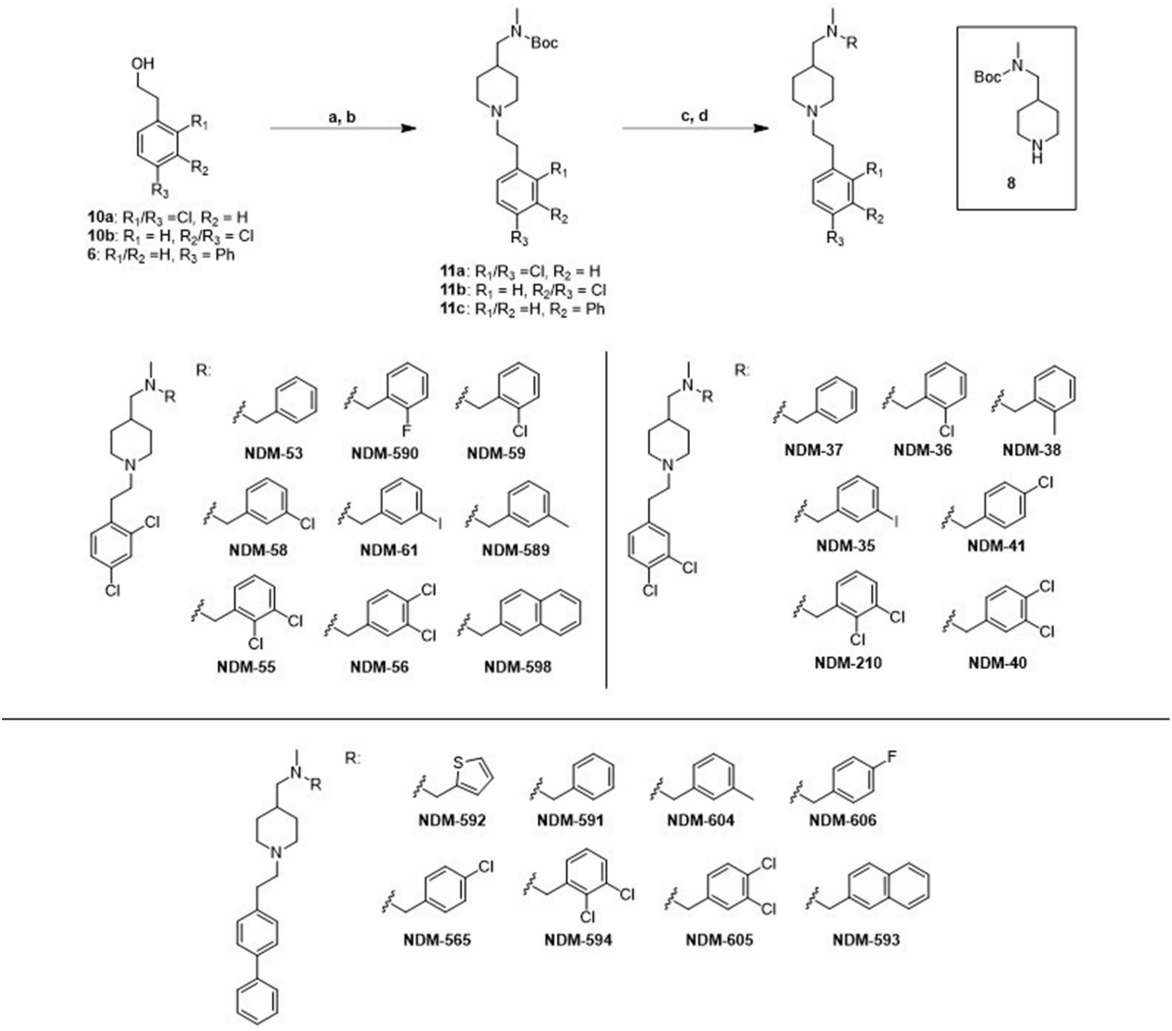
Synthesis of additional **JG‐1** analogs. Reagents and conditions: (a) MsCl, TEA, DCM, 0°C, 2 h (80%); (b) compound **8**, K_2_CO_3_, MeCN, reflux, O/N (44%–97%); (c) TFA, DCM, rt, 3 h (98%); (d) RBr, K_2_CO_3_, MeCN, reflux, 1 h (20%–64%).

This library was synthesized via a similar route to the library described above, with the exception that the synthesis initiated from primary alcohols **10a**, **10b**, or **6**, which were activated as their methanesulfonates prior to alkylation with compound **8** (Scheme 3). This set of compounds was screened at 10 µM and for dispersion 100 µM and compared to **JG‐1** via a one‐way ANOVA with Dunnett’s multiple comparison test with a threshold of *p *< 0.1 (Table S1). From this second library, two‐thirds of the compounds (16 of 24) passed the screen by having *p  *< 0.1 and were moved forward for further biofilm testing (Table 3).

Compounds with a 2,4‐dichlorophenyl tail and a 3‐substituted phenyl ring head (**NDM‐61**, **NDM‐589**) have comparable biofilm inhibition activity to that of **NDM‐295** (Table 3). With the same tail and a methyl at the 3‐position of the head phenyl ring (**NDM‐589**), biofilm dispersion activity decreases. Compounds with a 3,4‐dichlorophenyl tail all have IC_50s_ <10 µM, with **NDM‐41** (4‐chlorophenyl head group), having the lowest IC_50_ of 3.2 µM (95% CI [2.5, 4.1]), and overall improved EC_50_ (91.2 μM (95% CI [48.6, 174])) compared to **NDM‐295**. Compounds with the biphenyl motif showed varying improvement to biofilm inhibition and dispersion activity (Table 3). The analog with the biphenyl tail and a phenyl ring head (**NDM‐591**) has improved inhibition and dispersion activity compared to **NDM‐295** with an IC_50_ of 3.6 µM (95% CI [3.0, 4.2]) and an EC_50_ of 46.8 µM (95% CI [32.0, 68.5]). Improved biofilm dispersion compared to **NDM‐295** was also achieved by **NDM‐592** (2‐thiophene) and **NDM‐605** (3,4‐dichlorophenyl). Compounds **NDM‐606** (4‐fluorophenyl) and **NDM‐594** (2,3‐dichlorophenyl) both showed diminished dispersion activity with EC_50_s of 137.2 µM (95% CI [84.7, 222]) and 179.5 µM (95% CI [120, 268]), respectively. When the biphenyl tail was paired with a naphthalene head group (**NDM‐593**), the overall antibiofilm activity also decreased (Table 3).

As above, if a decrease in planktonic cells was observed, the compounds were evaluated for toxicity to the bacteria (Figure S1). We determined that **NDM‐55**, **NDM‐40**, **NDM‐592**, and **NDM‐605** have no significant effect on biofilm inhibition. Additionally, **NDM‐41**, **NDM‐565**, and **NDM‐606** were interestingly determined to have a killing effect at concentrations above the IC_50_. Similar to the the two previous compounds that exhibit toxicity (**NDM‐563** and **NDM‐564**), these compounds have substitution at the 4‐position of the phenyl ring of the head group. However, no further comparison can be drawn as the substituents differ in both electronics and sterics. Due to the toxicity of compound **NDM‐605**, an IC_50_ could not be determined.

Select compounds from this set were then assessed for inhibition of the hERG channel via the hERG fluorescence polarization assay (Table 4). We found that the addition of the naphthalene (**NMD‐589**) or a biphenyl motif (**NDM‐592**, **NDM‐591**, **NDM‐606**, **NDM‐594**, and **NDM‐593**) increased hERG channel binding, which is unsurprising given the increased hydrophobicity [31]. **NDM‐55**, however, has the least affinity for the hERG channel and returns an IC_50_ of 71.8 µM, demonstrating that in the context of biofilm dispersion, we can potentially significantly reduce hERG channel binding while maintaining/augmenting antibiofilm activity (Figure S2 and Table 4).

**TABLE 4:** IC_50_s for compounds from the hERG fluorescence polarization assay.

Compound	IC_50_, µM
**NDM‐295**	0.442
**NDM‐61**	0.579
**NDM‐589**	0.000134
**NDM‐55**	71.8
**NDM‐36**	0.00154
**NDM‐38**	0.195
**NDM‐35**	1.57
**NDM‐210**	0.00618
**NDM‐40**	4.17
**NDM‐592**	7.7e^−5^
**NDM‐591**	0.000119
**NDM‐606**	0.000567
**NDM‐594**	0.000172
**NDM‐593**	0.000606

Compounds **NDM‐47** (53.6) and **NDM‐55** (71.8 µM) demonstrated the lowest interaction with the hERG channel and exceeded the safety margin IC_50_ of ≥40 µM, which prompted examination for cytotoxicity against HepG2 (human liver) cells using a cell viability and proliferation XTT assay. This cell line is commonly used in screening for drug toxicity, as the liver is key in drug metabolism [32]. The IC_50_s of **NDM‐47** and **NDM‐55** against HepG2 are 104.2 ± 3.4 and 118.8 ± 8.9 µM, respectively (Figure S3), indicating that these compounds have significantly reduced toxicity toward mammalian cells in comparison to the antibiofilm activity.

## 3 Conclusion

Additional SAR studies conducted on **JG‐1** have led to the discovery of novel derivatives with improved biofilm inhibition and dispersion activity. The compound with the highest biofilm inhibition activity with an IC_50_ of 3.2 µM (95% CI [2.5, 4.1]) (**NDM‐41**) and the compound with the highest biofilm dispersion activity (while maintaining inhibitory activity) with an EC_50_ of 37.5 µM (95% CI [29.9, 47.0]) (**NDM‐36**) both harbor a 3,4‐dichlorophenyl tail group and have chloro substituents on the phenyl head group in the 2‐position or 4‐position, respectively.

The effects of **JG‐1** and analogs on hERG channel inhibition were also preliminarily investigated using a fluorescence‐based assay to rank order compounds for future testing using the patch‐clamp assay. Several compounds exhibit decreased inhibition of the hERG channel, with the most notable containing a 3‐iodophenyl (**NDM‐32** and **NDM‐35**), a dichlorophenyl (**NDM‐46**, **NDM‐55**, and **NDM‐40**), or a 4‐chlorophenyl head group (**NDM‐47**). **NDM‐55** exhibits the greatest decrease in hERG channel inhibition with an IC_50_ of 71.8 µM and enhanced dispersion activity. As dispersion is more biologically relevant activity, this makes **NDM‐55** a promising lead for further modification to further improve dispersal activity and decrease hERG channel inhibition that can be subsequently evaluated using our murine chronic carriage model.

## Funding

This study was supported by the National Institutes of Health (RO1AI116917, T32TR004543, and T32AI165391).

## Conflicts of Interest

The authors declare no conflicts of interest.

## Supporting information

Supplementary Material

## Data Availability

The data that supports the findings of this study are available in the Supporting Information of this article.

## References

[1] J. D. Stanaway , R. C. Reiner , B. F. Blacker , et al., “The Global Burden of Typhoid and Paratyphoid Fevers: A Systematic Analysis for the Global Burden of Disease Study,” The Lancet Infectious Diseases, 19 (2017): 369–381. 10.1016/S1473-3099(18)30685-6 PMC6437314 30792131

[2] Z. A. Bhutta , “Impact of Age and Drug Resistance on Mortality in Typhoid Fever,” Archives of Disease in Childhood 75 (1996): 214–217. 8976660 10.1136/adc.75.3.214 PMC1511710

[3] R. Chatterjee , A. R. Chowdhury , D. Mukherjee , D. Chakravortty , “From *Eberthella Typhi* to *Salmonella*, Typhi: The Fascinating Journey of the Virulence and Pathogenicity of *Salmonella* Typhi,” ACS Omega 8 (2023): 25674–25697. 37521659 10.1021/acsomega.3c02386 PMC10373206

[4] J.‐H. Kim , J. Im , P. Parajulee , et al., “A Systematic Review of Typhoid Fever Occurrence in Africa,” Clinical Infectious Diseases 69 (2019): 492–498. 10.1093/cid/ciz525 PMC6821235 31665777

[5] S. A. Hoffman , M. J. Sikorski , and M. M. Levine , “Chronic *Salmonella* Typhi Carriage at Sites other than the Gallbladder,” PLOS Neglected Tropical Diseases 17 (2023): e0011168. 36952437 10.1371/journal.pntd.0011168 PMC10035749

[6] K. V. Hoang , K. Woolard , C. Yang , C. Melander , and J. S. Gunn , “Identification of a Host‐Targeted Compound to Control Typhoid Fever,” American Society for Microbiology 10 (2022): e00619–00622. 10.1128/spectrum.00619-22 PMC9241869 35579463

[7] F. Jahan , S. V. Chinni , S. Samuggam , L. V. Reddy , M. Solayappan , and L. S. Yin , “The Complex Mechanism of the Salmonella typhi Biofilm Formation That Facilitates Pathogenicity: A Review,” International Journal of Molecular Sciences 23, no. 12 (2022): 6462. 35742906 10.3390/ijms23126462 PMC9223757

[8] A. Prinzi and R. Rohde , “The Role of Bacterial Biofilms in Antimicrobial Resistance,” American Society for Microbiology (2023), https://asm.org/Articles/2023/March/The‐Role‐of‐Bacterial‐Biofilms‐in‐Antimicrobial‐Re.

[9] K. J. Woolard , J. L. Sandala , R. J. Melander , J. S. Gunn , and C. Melander , “Development of Small Molecules that Work Cooperatively with Ciprofloxacin to Clear Salmonella Biofilms in Chronic Gallbladder Carriage Model,” European Journal of Medicinal Chemistry 232 (2022): 114203. 35219950 10.1016/j.ejmech.2022.114203 PMC8930541

[10] J. S. Gunn , J. M. Marshall , S. Baker , S. Dongol , R. C. Charles , and E. T. Ryan , “ *Salmonella* Chronic Carriage: Epidemiology, Diagnosis, and Gallbladder Persistence,” Trends in Microbiology 22, no. 11 (2014): 648–655, 10.1016/j.tim.2014.06.007. 25065707 PMC4252485

[11] G. Gonzalez‐Escobedo , J. M. Marshall , and J. S. Gunn , “Chronic and Acute Infection of the Gall Bladder by *Salmonella* Typhi: Understanding the Carrier State,” Nature Reviews Microbiology 9, no. 1 (2011): 9–14, 10.1038/nrmicro2490. 21113180 PMC3255095

[12] C. Marchello , S. D. Carr , and J. A. Crump , “A Systematic Review on Antimicrobial Resistance Amoung *Salmonella* Typhi Worldwide,” The American Journal of Tropical Medicine and Hygiene 103 (2020): 2518–2527. 32996447 10.4269/ajtmh.20-0258 PMC7695120

[13] World Health Organization Regional Office for the Eastern Mediterranean. Drug resistant *Salmonella* infections in Pakistan: update., Weekly epidemiologic monitor, Issue 34, 2019.

[14] W. Wong , H. A. Rawahi , S. Patel , et al., “The First Canadian Pediatric Case of Extensively Drug‐Resistant, *Salmonella*, Typhi Orginiating from an Outbreak in Pakistan and its Implication for Empiric Antimicrobial Choices,” ID Cases 15 (2019): e00492. 30815359 10.1016/j.idcr.2019.e00492 PMC6378779

[15] A. L. Engsbro , H. S. R. Jespersen , M. I. Goldschmidt , et al., “Ceftriaxone‐Resistant *Salmonella* enterica Serotype Typhi in a Pregnant Travellwe Returning from Karachi, Pakistan to Denmark, 2019,” European Surveillance 24 (2019): 1900289. 10.2807/1560-7917.ES.2019.24.21.1900289 PMC6540646 31138366

[16] A. Howard‐Jones , A. M. Kesson , A. C. Outhred , and P. N. Britton , “First Reported Case of Extensively Drug‐Resistant Typhoid in Australia,” The Medical Journal of Australia 211 (2019): 286. 31441053 10.5694/mja2.50316

[17] K. Chatham‐Stephens , F. Medalla , M. Hughes , et al., “Emergence of Extensively Drug‐Resistant *Salmonella* Typhi Infections among Travelers to or from Pakistan ‐ United States,” Morbidity and Mortality Weekly Report 68 (2019): 11–13. 30629573 10.15585/mmwr.mm6801a3 PMC6342547

[18] C.‐E. Kleine , S. Schlabe , G. T. R. Hischebeth , et al., “Succesful Therapy of a Multidrug‐Resistant Extended‐Spectrum Beta‐Lactamaze‐Producing and Fluoroquinolone‐Resistant *Salmonella* enterica Subspecies enterica serovar Typhi Infection Using Combination Therapy of Meropenem and Fosfomycin,” Clinical Infectious Diseases 65 (2017): 1754–1756. 29020162 10.1093/cid/cix652

[19] J. L. Sandala , B. W. Eichar , L. G. Kuo , et al., “A Dual‐Therapy Appraoch for the Treatment of Biofilm‐Mediated *Salmonella* Gallbladder Carriage,” PLOS Pathogens 16 (2020): e1009192. 33370414 10.1371/journal.ppat.1009192 PMC7793255

[20] A. Garrido , A. Lepailleur , S. M. Mignani , P. Dallemagne , and C. Rochais , “hERG Toxicity Assessment: Useful Guidelines for Drug Design,” European Journal of Medicinal Chemistry 195 (2020): 112290. 32283295 10.1016/j.ejmech.2020.112290

[21] E. Neher and B. Sakmann , “The Patch Clamp Technique,” Scientific American 266 (1992): 44–51. 1374932 10.1038/scientificamerican0392-44

[22] D. R. Piper , S. R. Duff , H. C. Eliason , et al., “Development of the Predictor hERG Fluorescence Polarization Assay Using a Membrane Protein Enrichment Approach,” ASSAY and Drug Development Technologies 6 (2008): 213–223. 18471075 10.1089/adt.2008.137

[23] F. W. Goldberg , A. K. T. Ting , D. Beattie , et al., “Optimization of hERG and Pharmacokinetic Properties for Basic Dihydro‐8H‐Purin‐8‐One Inhibitors of DNS‐PK,” ACS Medicinal Chemistry Letters 13 (2022): 1295–1301. 35978693 10.1021/acsmedchemlett.2c00172 PMC9377022

[24] X. Dong , W. Zhan , M. Zhao , et al., “Discovery of 3,4,6‐Trisubstituted Piperidine Derivatives as Orally Active, Low hERG Blocking Akt Inhibitors via Conformational Restriction and Structure‐Based Design,” Journal of Medicinal Chemistry 62 (2019): 7264–7288. 31298542 10.1021/acs.jmedchem.9b00891

[25] M. Furber , A.‐K. Tiden , P. Gardiner , et al., “Cathepsin C Inhibitors: Property Optimization and Identification of a Clinical Candidate,” Journal of Medicinal Chemistry 57 (2014): 2357–2367. 24592859 10.1021/jm401705g

[26] Z.‐Q. Yang , J. C. Barrow , W. D. Shipe , et al., “Discovery of 1,4‐Substituted Piperidines as Potent and Selective Inhibitors of T‐Type Calcium Channels,” Journal of Medicinal Chemistry 51, no. 20 (2008): 6471–6477, 10.1021/jm800830n. 18817368

[27] A. Johansson , C. Lofberg , M. Antonsson , et al., “Discovery of (3‐(4‐(2‐Oxa‐6‐azaspiro[3.3]heptan‐6‐ylmethyl)phenoxy)azetidin‐1‐yl)methanone (AZD1979), a Melanin Concentrating Hormone Receptor 1 (MCHr1) Antagonist with Favorable Physicochemical Properties,” Journal of Medicinal Chemistry 59 (2016): 2497–2511. 26741166 10.1021/acs.jmedchem.5b01654

[28] A. F. Pozharskii , “Concept of π‐Deficiency in the Chemistry of Heteroaromatic Compounds (Review),” Chemistry of Heterocyclic Compounds 15 (1979): 939–954.

[29] S. Kumar and A. Das , “Effect of Acceptor Heteroatoms on π‐Hydrogen Bonding Interactions: A Study of Indole⋅⋅⋅Thiophene Heterodimer in a Supersonic Jet,” The Journal of Chemical Physics 137, no. 9 (2012): 094309, 10.1063/1.4748818. 22957571

[30] A. N. Bennett , B. Laipply , and J. S. Gunn , “Methods for Detecting and Monitoring Salmonella Infection and Chronic Carriage in Living Mice Using Bioluminescent In Vivo Imaging,” Access Microbiology 6, no. 11 (2024): 000913.v3, 10.1099/acmi.0.000913.v3. PMC11605878 39619287

[31] Y. Kawai , S. Tsukamoto , J. Ito , K. Akimoto , and M. Takahashi , “A Risk Assessment of Human Ether‐a‐Go‐Go‐Related Gene Potassium Channel Inhibition by Using Lipophilicity and Basicity for Drug Discovery,” Chemical and Pharamaceutical Bulletin 59 (2011): 1110–1116. 10.1248/cpb.59.1110 21881254

[32] S. Miret , E. M. De Groene , and W. Klaffke , “Comparison of In Vitro Assays of Cellular Toxicity in the Human Hepatic Cell Line HepG2,” Journal of Biomolecular Screening 11, no. 2 (2006): 184–193, 10.1177/1087057105283787. 16314402

