# Supplementary material for: Structure–Activity Relationship Analysis and hERG Liability Assessment of Second‐Generation Antibiofilm Compounds Active Against Salmonella
Source: ChemMedChem. 2026 Jul 12;21(13):e70363. doi: 10.1002/cmdc.70363 (PMC13357029; doi:10.1002/cmdc.70363)
Supplement: Supplementary file 1 — Supplementary Material [file CMDC-21-e70363-s001.pdf]

Supplementary Information

Structure-Activity Relationship Analysis and hERG Liability Assessment of Second-Generation Anti-Biofilm Compounds Active Against *Salmonella*

Amy Sorge,[1] Aliyah N. Bennett[2,3,5], Sophia E. Gregory,[1] Allysa L. Cole[2,3,4,5], Katherine J. Woolard,[1] Ansley M. Nemeth,[1] Andrew H. Crow,[1] Roberta J. Melander,[1] John S. Gunn,[2,3,5*] Christian Melander[1*]

[1]Department of Chemistry and Biochemistry, University of Notre Dame, Notre Dame, IN, United States
[2]Center for Microbe and Immunity Research, Abigail Wexner Research Institute at Nationwide Children's Hospital, Columbus, OH, United States
[3]Infectious Diseases Institute, The Ohio State University, Columbus, OH, United States
[4]Department of Veterinary Biosciences, The Ohio State University, Columbus, OH, United States
[5]Department of Pediatrics, College of Medicine, The Ohio State University, Columbus, OH, United States
[*]Corresponding Author(s): John.Gunn@nationwidechildrens.org; cmelande@nd.edu

Table of Contents

Biological Data S2

Table S1. P-value from one-way ANOVA with Dunnett's multiple comparison .. S2

Figure S1. Bacterial toxicity of **JG-1**, **NDM-41**, **NDM-605**, **NDM-606**, **NDM-563**, **NDM-563**, **NDM-564**, and **NDM-565** S4

Figure S2. hERG channel fluorescence polarization S5

Figure S3. Cytotoxicity for **NDM-47** and **NDM-55** S5

Methods and Materials.. S6

Synthesis of previously reported compounds S10

Characterization of novel compounds S14

^{1}H and ^{13}C NMR spectra.. S48

References.. S150

Biological Data

Table S1. Compounds were screened for inhibitory activity at 10 µM. Compounds that showed promising activity were dose response assay from which *in vitro* IC_{50}s and EC_{50}s were determined and described in Table 1. The derivative compounds' IC_{50}/EC_{50} values were compared to JG-1 via a one-way ANOVA with Dunnett's multiple comparison testing. The resulting P-values of each comparison are described below. NP = not performed

**Compound**	**IC_{50} p-value**	**EC_{50} p-value**
**NDM-32**	<0.0001	<0.0001
**NDM-35**	<0.0001	<0.0001
**NDM-36**	<0.0001	<0.0001
**NDM-37**	NP	NP
**NDM-38**	<0.0001	0.0001
**NDM-40**	<0.0001	0.0002
**NDM-41**	<0.0001	0.0066
**NDM-43**	>0.1	>0.1
**NDM-45**	>0.1	>0.1
**NDM-46**	<0.0001	<0.0001
**NDM-47**	<0.0001	<0.0001
**NDM-48**	>0.1	>0.1
**NDM-49**	>0.1	>0.1
**NDM-51**	>0.1	>0.1
**NDM-52**	>0.1	>0.1
**NDM-53**	>0.1	>0.1
**NDM-55**	<0.0001	0.0002
**NDM-56**	>0.1	>0.1
**NDM-58**	>0.1	>0.1
**NDM-59**	>0.1	>0.1
**NDM-61**	<0.0001	<0.0001
**NDM-72**	>0.1	>0.1
**NDM-73**	>0.1	>0.1
**NDM-210**	<0.0001	<0.0001
**NDM-294**	>0.1	>0.1
**NDM-295**	<0.0001	<0.0001
**NDM-556**	<0.0001	<0.0001
**NDM-557**	<0.0001	<0.0001
**NDM-558**	<0.0001	<0.0001
**NDM-559**	<0.0001	0.061
**NDM-560**	<0.0001	0.069
**NDM-561**	<0.0001	<0.0001
**NDM-562**	<0.0001	0.0023
**NDM-563**	<0.0001	<0.0001
**NDM-564**	<0.0001	<0.0001

**NDM-565**	<0.0001	<0.0001
**NDM-589**	<0.0001	0.0334
**NDM-590**	>0.1	>0.1
**NDM-591**	<0.0001	0.0001
**NDM-592**	<0.0001	<0.0001
**NDM-593**	0.0002	0.0055
**NDM-594**	<0.0001	0.0012
**NDM-597**	>0.1	>0.1
**NDM-598**	>0.1	>0.1
**NDM-599**	0.0006	>0.1
**NDM-600**	<0.0001	>0.1
**NDM-601**	>0.1	>0.1
**NDM-602**	>0.1	>0.1
**NDM-603**	>0.1	>0.1
**NDM-604**	>0.1	>0.1
**NDM-605**	<0.0001	0.0019
**NDM-606**	<0.0001	0.0398
**NDM-637**	NP	NP

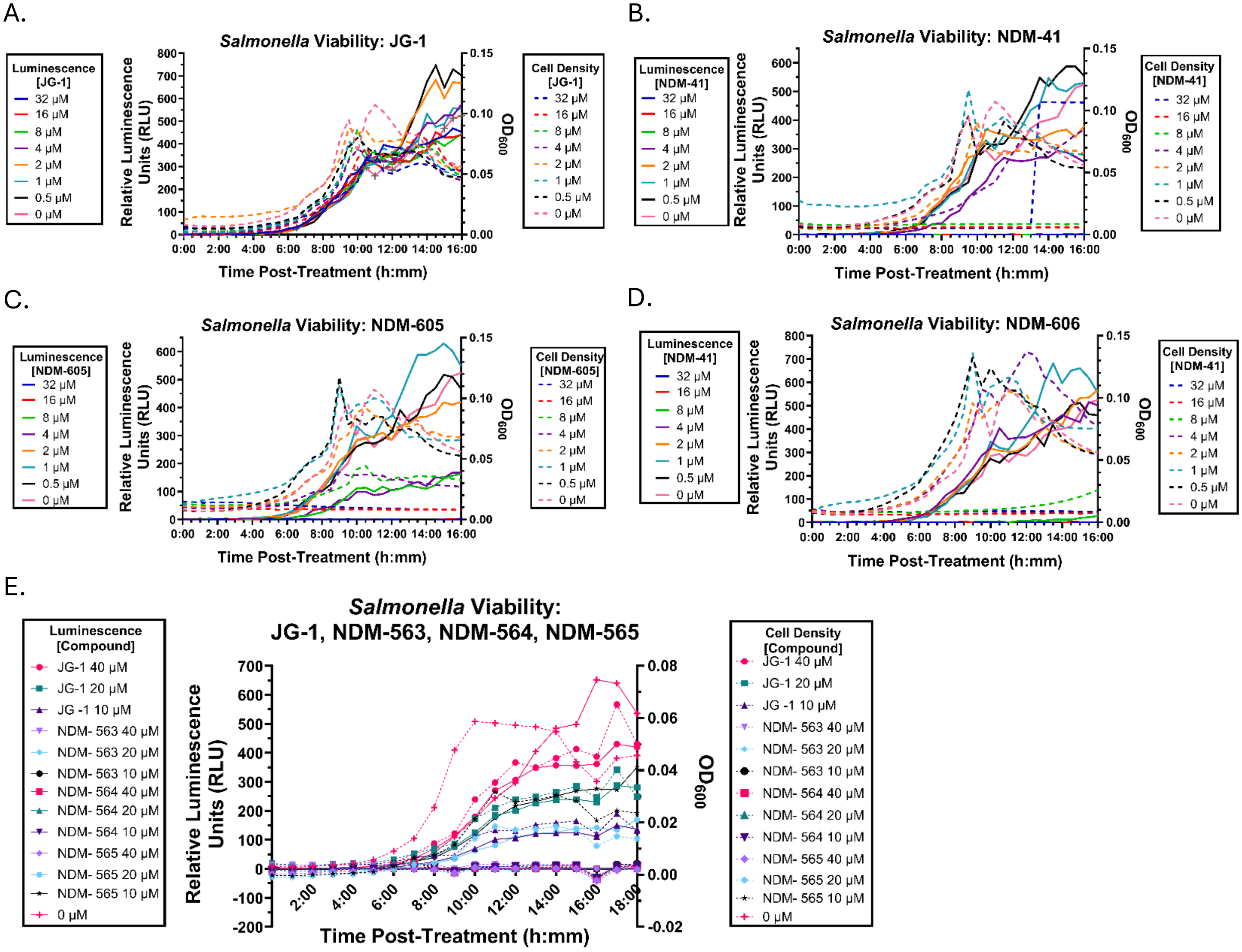

Figure S1. Bacterial toxicity of **JG-1**, **NDM-41**, **NDM-605**, **NDM-606**, **NDM-563**, **NDM-564**, and **NDM-565.** A bioluminescent isolate of *Salmonella* Typhimurium (JSG 4922) was incubated with compounds **(A)** JG-1, **(B)** NDM-41, **(C)** NDM-605, and **(D)** NDM-606, at concentrations between 0.5-32 µM, or vehicle alone (0 µM), in the same conditions as the biofilm inhibition assays, but using a 96-black well, clear bottom, non-treated polystyrene plate. The plate was incubated in a Spectramax M3 plate reader at 25°C, and the relative luminescence units (RLU) and cell density (OD_{600}) of each well were measured every 30 minutes for 16 hours, shaking the plate between reads. **(E)** JSG 4922 was incubated with JG-1, NDM-563, NDM-564, and NDM-565 at concentrations of 10- 40 µM or vehicle alone (0 µM), in the same conditions as above. Relative luminescence units (RLU) and OD_{600} of each well were measured hourly for 18 hours, shaking the plate between reads. n=2 biological replicates. Solid lines represent the RLU curve (left y-axis) and dashed lines represent OD_{600} curve (right y-axis) for each compound/concentration during the experiment. Symbols represent the mean RLU or OD_{600} at each time point. n=2 biological replicates.

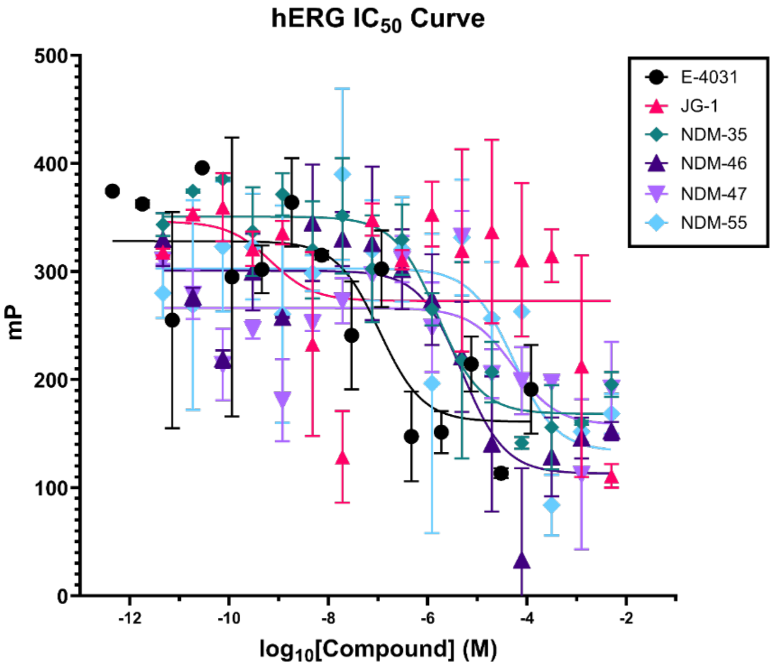

Figure S2. hERG channel fluorescence polarization. The hERG channel affinity of JG-1 and several other derivative compounds was measured using the Predictor^{TM} hERG Fluorescence Polarization Assay Kit. The hERG channel affinity of E-4031, a known hERG inhibitor, included with the kit, was measured in tandem. Symbols represent the mean and standard error of fluorescence polarization (mP). From this data, IC$_{50}$ curves were plotted using a non-linear regression model. Graphs created using GraphPad Prism, version 10.3.1.

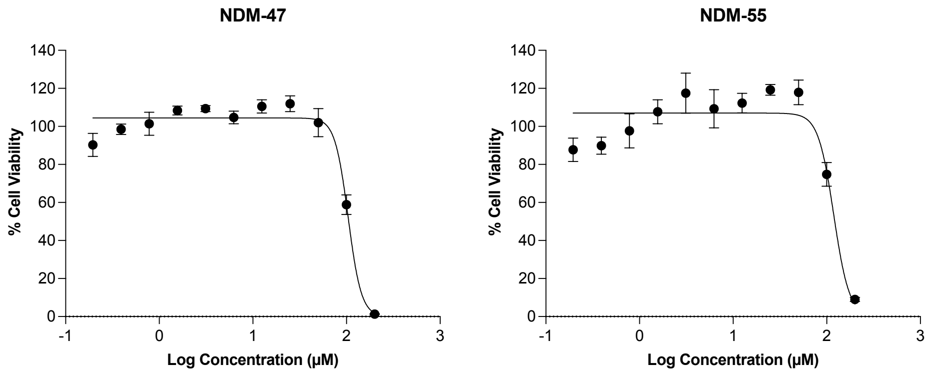

Figure S3. HepG2 cell viability for **NDM-47** and **NDM-55**

Methods and Materials

Bacterial strains and antimicrobial agents: *S.* Typhimurium ATCC 14028 (JSG210) was obtained from Roy Curtiss III from University of Florida, College of Veterinary Medicine. The bioluminescent isolate of *S.* Typhimurium used in this study, JSG 4922, was previously described by Bennett, A. N. et al. [8] Colonies were grown on Luria Bertani broth and agar (LB) [Thermo Scientific Cat No.BP1426 and BP1423-2] and Tryptic soy broth (TSB) [Thermo Scientific Cat No.DF0370-007-5]...

Biofilm Dispersion and Inhibition Assays. Individual *S.* Typhimurium ATCC 14028 colonies were used to inoculate tryptic soy broth (TSB) for overnight liquid cultures grown at 37 °C with aeration in a rolling drum. For disruption assays, the overnight cultures were normalized to an optical density at 600 nm (OD_{600}) of 0.8 in TSB before a 1:2500 dilution in 1:20 TSB. 100 µL of diluted culture was added to each well of a 96-well non-treated polystyrene plate (Corning Cat No. 7200656) for inoculation of biofilms. Biofilms were grown for 24 hours at 25 °C on a rotating nutator prior to treatment. At 24 hours, spent media was removed and replaced with 1:20 TSB containing either compounds or vehicle (DMSO) at designated concentrations. Biofilms were allowed to grow for an additional 24 hours prior to analysis via crystal violet staining. For inhibition assays, biofilms were grown as described above in the presence of compounds or vehicle at designated tested concentrations at the time of plate inoculation. Biofilms were grown for 24 hours at 25 °C on a rotating nutator prior to analysis via crystal violet staining.

Biofilm Mass Calculations. Biofilm mass was quantified using the previously reported crystal violet assay.[1] Briefly, after incubation, the supernatant in each well was discarded and the planktonic cells were removed by a wash through submerging the 96-well plate in fresh dH_2O. Biofilms were heat fixed at 60 °C for 1 hour. 100 µL of crystal violet solution (6 mL phosphate buffered saline, 3.3 mL 1% (w/v) crystal violet solution [Aqua Solutions, C8126], 333 µL isopropanol, and 333 µL methanol) was added to each well and incubated for 5 minutes at room temperature. The crystal violet stain was discarded and the plates were washed twice with dH_2O. The bound crystal violet was solubilized with 100 µL of 33% glacial acetic acid (Fisher Scientific Cat No. A38-212) solution. The OD_{570} of each well was then measured using a Spectramax M3 plate reader (VWR).

IC_{50}/EC_{50} Determination. The concentration of a compound needed to achieve a response halfway between baseline and maximum, defined as 50% biofilm formed or remaining relative to the vehicle control, was calculated for compounds in the inhibition (IC_{50}) and disruption (EC_{50}) assays based on crystal violet staining quantification. IC_{50}/EC_{50} curves were calculated via a nonlinear regression model, log(inhibitor) vs. normalized response-Variable slope, using the software, GraphPad Prism, version 10.3.1. IC_{50}/EC_{50} values are reported with their respective confidence intervals (95%). Using this regression model, some compounds demonstrated unstable parameters and were unable to fit a curve, therefore IC_{50}/EC_{50} values are reported as not determined (ND).

Bacterial Toxicity Kinetic Assays. Individual colonies of a bioluminescent isolate of *Salmonella* Typhimurium (JSG 4922) were inoculated in TSB for overnight liquid cultures grown at 37 °C with aeration in a rolling drum. JSG 4922 has a chromosomally inserted *lux* operon, causing it to

constitutively produce light when O_2 and sufficient conditions to produce ATP are present. Normalization and dilution of the overnight culture was performed the same as the inhibition assays described above, but using a 96-black well, clear bottom, non-treated polystyrene plate (Corning Cat No. 07-200-567). The plate was incubated in the same plate reader as above, at 25°C. Relative luminescence units (RLU) and OD_{600} of each well were measured serially, shaking the plate between reads, to measure changes in cell metabolic activity (RLU) and cell density (OD_{600}) of compound treated bacteria. Graphs were created using GraphPad Prism, version 10.3.1.

hERG Channel Activity Measurement. hERG channel affinity was measured using the Predictor™ hERG Fluorescence Polarization Assay Kit (Invitrogen, PV5365) according to their established protocol. Briefly, a range of concentrations of compounds (4.7×10^{-9} to 5 mM) were added to wells with the hERG channel ligand membrane and a fluorescent tracer and were incubated for 2 hours. Additionally, E-4031 (1.1×10^{-7} to 120 mM), a known hERG inhibitor included with the kit, and vehicle control (DMSO) were incubated in wells with membrane and tracer under the same conditions as the compounds. The amount of binding between the compound and hERG channel ligand was measured on a fluorescent plate reader (Tecan, Spark) with excitement at 530 nm and emission at 585 nm.IC_{50} was calculated with the non-linear regression model, log(inhibitor) vs. response (three parameters) and compared to JG-1 via an Extra sum-of-squares F-test ($p<0.05$), using GraphPad Prism, version 10.3.1.

XTT Assay with HepG2 Cells. Cell viability of hepatocellular carcinoma cell line, HepG2 (ATTC HB-8065) was investigated as described previously with minor changes.[2] HepG2 cells were incubated and maintained at 37 ˚C under 5% CO_2. Culture medium, Dulbecco's High Glucose Modified Eagles Medium (DMEM; Cytiva) supplemented with 10% heat-inactivated fetal bovine serum (FBS; Cytiva) and 1% penicillin-streptomycin (Cytiva), was changed every 2-3 days. Cells were seeded into a 96-well plate at a concentration of 5×10^4 cells/well in 100 µL culture medium. Plates were incubated for 24 h at 37 ˚C under 5% CO_2. The media was removed from each well, and to the first row of wells containing cells, 200 µL medium containing compound to be tested at the highest desired concentration was added. To the remaining wells containing cells, 100 µL fresh DMEM was added. A serial dilution was completed leaving the last two rows unmixed. One unmixed row contained fresh media and cells which was used as a positive control and the other unmixed row contained only fresh media which was used as a blank. After serial dilution, plates were incubated for 24 h at 37 ˚C under 5% CO_2. An estimation of viable cells was enumerated using an XTT Cell Viability Kit (Biotium). Activated XTT solution (50 µL of 25 µL activation reagent mixed with 5 mL XTT reagent) was added each well, followed by incubation for 2 – 4 hr at 37 ˚C under 5% CO_2. After incubation, the plate was shaken gently to evenly distribute the dye in the wells. Absorbance of each well was measured at both 475 nm and 660 nm using a BioTek Synergy HTX Multimode plate reader. Using these absorbances, percent viability was calculated. Percent viability (y-axis) was plotted versus compound concentration (x-axis, log-scale) and IC_{50}s were determined using GraphPad Prism. Assays were completed in triplicate a minimum of two times.
The percent viability was calculated as follows:
Specific Absorbance = A_{475} (test) – A_{475} (blank average) – A_{660} (test)
Percent Viability = Specific Abs. (test)/Average Specific Abs. (untreated cells) x 100

Chemistry Experimental: All reagents used for chemical synthesis were purchased from commercially available sources (VWR U.S., Fisher Scientific U.S., Ambeed, Oakwood Chemical, Matrix Scientific, or Sigma Aldrich U.S.) and used without further purification. Flash chromatography was performed using 60 Å mesh standard grade silica gel from Sorbetch. NMR solvents were obtained from Cambridge Isotope Labs and used as is. All ^{1}H NMR and ^{13}C NMR were recorded at 25 ˚C on Bruker AVANCE III HD spectrometers (400 MHz or 500 MHz). Chemical shifts (δ) are given in parts per million (ppm) relative to the respective NMR solvent; coupling constants (*J*) are in hertz (Hz). Abbreviations used are s, singlet; d, doublet; dd, doublet of doublets; ddd, doublet of doublet of doublets; t, triplet; m, multiplet. High-resolution mass spectrometry measurements were obtained at the Notre Dame Department of Chemistry Mass Spectrometry and Proteomics Facility. Infrared spectra were obtained on a Bruker Alpha II FTIR spectrophotometer (ν_{max} in cm^{-1}). UV absorbance was recorded on a Genesys 10 scanning UV/visible spectrophotometer (λ_{max} in nm). The purities of the tested compounds were all verified to be ≥ 95% by LC-MS analysis on an Advion LC-MS 2020 with Kinetex, 2.6 mm, C_{18} 50 × 2.10 mm, using 20-100% acetonitrile/water with 0.1% formic acid for either 5 or 3 minutes.

General Synthetic Procedure for *N*-methyl-1-(thiophen-3-yl)methanamine Addition. The Boc-protected carboxylic acid (1.85 mmol) was added to a 100 mL round bottom flask with TEA (7.45 mmol), EDC (4.65 mmol), *N*-methyl-1-(thiophen-3-yl)methanamine (3.90 mmol), DMAP (205 µmol), and 20 mL dichloromethane. The reaction was stirred at room temperature for 24 hours. The crude product was washed with brine (1x30 mL) and extracted with DCM (3x30 mL), dried over anhydrous sodium sulfate, filtered, concentrated in vacuo, and purified via flash column chromatography using 2% MeOH saturated with ammonia and 98% DCM to yield the purified product.

General Synthetic Procedure for Boc-Deprotection. Boc-protected intermediate (0.40 mmol) was dissolved in 1 mL dichloromethane. Trifluoroacetic acid (0.2 mL) was added, and the reaction was allowed to stir at room temperature open to atmosphere for 1 hour. The reaction was concentrated under vacuo, methanol (2 mL) was then added, and the mixture was evaporated in vacuo to dryness. Addition of ethanol followed by evaporation was repeated four times (or until no further vapors evolved upon addition of solvent). The crude intermediate was dried for 18 hours under high vacuum and used without further purification.

General Synthetic Procedure for Amide Reduction to Tertiary Amine. Lithium aluminum hydride (7.8 mmol, 3 molar) and 5 mL anhydrous THF were added to an oven-dried two-neck round bottom flask under argon. The reaction was heated to reflux. Amide compound (1.5 mmol) was dissolved in 1 mL of anhydrous THF and added to the reaction flask dropwise under argon. The reaction was stirred at 66 °C for 2.5 hrs. The reaction was then cooled to 0 °C and an aqueous saturated solution of Rochelle salt (7 mL) was added dropwise over 30 minutes to quench excess LAH. The reaction mixture was then stirred at rt for 1 hr. The crude mixture was filtered under vacuum, washed with THF (20 mL), and the filtrate concentrated in vacuo. The amine was then purified via flash chromatography with 2% MeOH saturated with ammonia and 98% DCM to yield the purified product.

General Synthetic Procedure for Alcohol Mesylation. The alcohol (3.0 mmol) was dissolved in 12 mL anhydrous dichloromethane and added to an oven-dried round bottom flask under argon.

Anhydrous triethylamine (6.0 mmol) was added and the solution was cooled to 0 °C. Methanesulfonyl chloride (9.0 mmol) was added in one portion, and the solution was allowed to stir for 30 minutes at 0 °C followed by 2 hours as it warmed to room temperature. The mixture was quenched with 20 mL di-H_2O and extracted with chloroform (3x30 mL). Organic layers were combined, washed with brine, dried over anhydrous sodium sulfate, filtered, and concentrated in vacuo. The crude mixture was then purified via flash chromatography with 30% ethyl acetate and 70% hexanes.

General Synthetic Procedure for Amine Alkylation. Deprotected intermediate (0.40 mmol) was dissolved in 23 mL anhydrous acetonitrile and transferred to an oven-dried 100 mL three-necked flask under argon. Potassium carbonate (1.50 mmol) was added and the mixture was heated to reflux while stirring. Alkyl bromide (0.45 mmol) was dissolved in 2 mL anhydrous acetonitrile and added to the reaction dropwise over one hour. The reaction was checked for completion by TLC after full addition of bromide, then cooled and transferred to a single-necked 250 mL round bottom flask and evaporated in vacuo. The crude product was then taken up in di-H_2O (50 mL) and extracted with chloroform (3x30 mL). The organic layers were combined and washed with brine (30 mL), dried over anhydrous sodium sulfate, filtered, and evaporated under reduced pressure. The crude mixture was purified via flash chromatography using 2% methanol, 2% triethylamine, and 96% chloroform.

General Synthetic Procedure for Piperidine Alkylation. To an oven-dried two-necked 100 mL round bottom flask under argon was added anhydrous acetonitrile (30 mL) and *tert*-butyl methyl(piperidin-4-ylmethyl)carbamate (0.50 mmol). Oven-dried potassium carbonate (1.5 mmol) was added to the flask in one portion, and the resulting mixture was heated to reflux. Alkyl bromide or mesylated compound was added neat in one portion (1.25 mmol) and the reaction mixture was stirred under reflux for 24 hours. The reaction was then cooled and transferred to a single-necked 250 mL round bottom flask to be concentrated under reduced pressure. The crude product was then taken up in di-H_2O (50 mL) and extracted with chloroform (3x30 mL). The organic layers were combined and washed with brine (30 mL), dried over anhydrous sodium sulfate, filtered, evaporated in vacuo, and purified via flash chromatography using 2% methanol, 2% triethylamine, and 96% chloroform.

General Synthetic Procedure for Formation of Alkyl Bromides. An alcohol (4.0 mmol) was dissolved in diethyl ether (20 mL) and cooled to 0 °C. Phosphorus tribromide (4.0 mmol) was added dropwise to the solution. The mixture was stirred at 0 °C for 1.66 hours and then warmed to room temperature to stir for an additional 0.5 hours. The reaction was then quenched with saturated aqueous sodium bicarbonate (10 mL) and extracted with diethyl ether (10 mL x 3). The organic layers were combined, evaporated in vacuo, and immediately stored for short periods at -20 °C with no further purification. Over time the alkyl bromides degraded.

General Synthetic Procedure for Formation of HCl Salts. Pure product was dissolved in 1 mL methanol and concentrated hydrochloric acid (0.2 mL) was added. The solvent was evaporated in vacuo. Ethanol addition and subsequent evaporation was repeated six times (until no further fumes evolved upon solvent addition). The resulting solid was dried under vacuum for 24-48 hours to yield the salt.

Synthesis of previously reported compounds

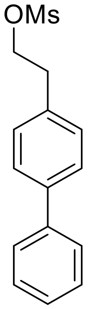

2-([1,1'-Biphenyl]-4-yl)ethyl methanesulfonate (7): 2-([1,1'-biphenyl]-4-yl)ethan-1-ol (**6**) was reacted with methanesulfonyl chloride in anhydrous dichloromethane following the general synthetic procedure for alcohol mesylation. Spectral data were consistent with previous reports.[7] ^{1}H NMR (400 MHz, chloroform-d) δ 7.60 – 7.54 (m, 4H), 7.44 (dd, *J* = 8.4, 6.8 Hz, 2H), 7.38 – 7.34 (m, 1H), 7.34 – 7.29 (m, 2H), 4.46 (t, *J* = 6.9 Hz, 2H), 3.11 (t, *J* = 6.9 Hz, 2H), 2.89 (s, 3H). HRMS *m/z* calculated for $C_{15}H_{17}O_3S$ [M+H]$^+$: 276.0820, found 276.0818.

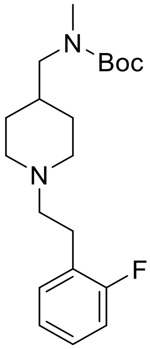

***tert*-Butyl ((1-(2-fluorophenethyl)piperidin-4-yl)methyl)(methyl)carbamate (9):** Compound **8** was reacted with 1-(2-bromoethyl)-2-fluorobenzene and potassium carbonate in acetonitrile following the general procedure for piperidine alkylation.[1] Spectral data were consistent with previous reports.[1] ^{1}H NMR (400 MHz, chloroform-d) δ 7.23 – 7.12 (m, 2H), 7.08 – 6.95 (m, 2H), 3.10 (d, *J* = 8.9 Hz, 2H), 3.03 (d, *J* = 9.9 Hz, 2H), 2.89 – 2.82 (m, 5H), 2.63 – 2.54 (m, 2H), 2.08 – 1.97 (m, 2H), 1.65 (d, *J* = 12.3 Hz, 3H), 1.45 (s, 9H), 1.33 – 1.28 (m, 2H). HRMS *m/z* calculated for $C_{20}H_{32}FN_2O_2$ [M+H]$^+$: 351.2442, found: 351.2440.

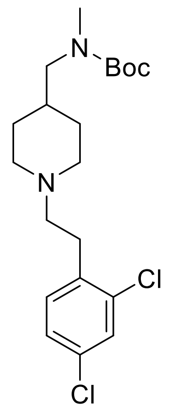

***tert*-Butyl ((1-(2,4-dichlorophenethyl)piperidin-4-yl)methyl)(methyl)carbamate (11a):** Compound **10a** was reacted with compound **8** and potassium carbonate in acetonitrile following the general procedure for piperidine alkylation.[3] Spectral data were consistent with previous reports.[3] ^{1}H NMR (400 MHz, methanol-d_4) δ 7.44 (d, *J* = 2.0 Hz, 1H), 7.31 (s, 1H), 7.28 (dd, *J* = 8.2, 2.0 Hz, 1H), 3.20 – 3.13 (m, 2H), 3.07 (d, *J* = 10.9, 2H), 3.00 – 2.93 (m, 2H), 2.92 – 2.84 (m, 3H), 2.63 – 2.54 (m, 2H), 2.13 (t, *J* = 10.8 Hz, 2H), 1.77 – 1.65 (m, 3H), 1.47 (s, 9H), 1.40 – 1.27 (m, 2H). HRMS *m/z* calculated for $C_{20}H_{31}Cl_2N_2O_2$ $[M+H]^+$: 401.1757, found: 401.1772.

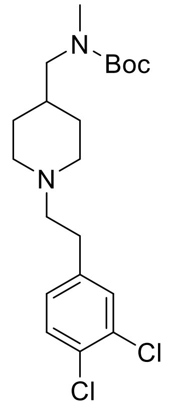

***tert*-Butyl ((1-(3,4-dichlorophenethyl)piperidin-4-yl)methyl)(methyl)carbamate (11b):** Compound **10b** was reacted with compound **8** and potassium carbonate in acetonitrile following the general procedure for piperidine alkylation.[3] Spectral data were consistent with previous reports.[3] ^{1}H NMR (400 MHz, methanol-d_4) δ 7.41 (d, *J* = 6.3 Hz, 1H), 7.40 (s, 1H), 7.16 (dd, *J* = 8.2, 2.0 Hz, 1H), 3.14 (s, 2H), 3.03 (d, *J* = 10.6 Hz, 2H), 2.89 – 2.83 (m, 3H), 2.83 – 2.76 (m, 2H), 2.61 – 2.54 (m, 2H), 2.07 (t, *J* = 11.3 Hz, 2H), 1.72 – 1.59 (m, 3H), 1.45 (s, 9H), 1.37 – 1.21 (m, 2H). HRMS *m/z* calculated for $C_{20}H_{31}Cl_2N_2O_2$ $[M+H]^+$: 401.1757, found: 401.1766.

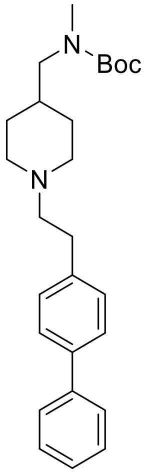

tert-Butyl ((1-(2-([1,1'-biphenyl]-4-yl)ethyl)piperidin-4-yl)methyl)(methyl)carbamate (11c): Compound **11c** was reacted with compound **8** and potassium carbonate in acetonitrile following the general procedure for piperidine alkylation.[3] Spectral data were consistent with previous reports.[3] ^{1}H NMR (400 MHz, chloroform-d) δ 7.60 – 7.55 (m, 2H), 7.52 (d, J = 7.9 Hz, 2H), 7.43 (t, J = 7.5 Hz, 2H), 7.33 (t, J = 7.3 Hz, 1H), 7.28 (d, J = 8.1 Hz, 2H), 3.12 (d, J = 6.8 Hz, 2H), 3.09 – 3.00 (m, 2H), 2.86 (s, 3H), 2.63 (m, 2H), 2.02 (m, 2H), 1.72 – 1.63 (m 2H), 1.62 – 1.52 (m, 3H), 1.46 (s, 9H), 1.40 – 1.27 (m, 2H). HRMS *m/z* calculated for $C_{26}H_{37}N_2O_2$ $[M+H]^+$: 409.2850, found 409.2851.

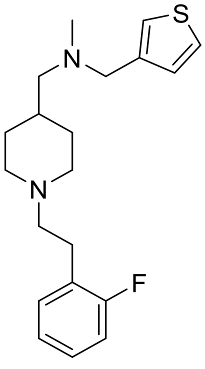

1-(1-(2-Fluorophenethyl)piperidin-4-yl)-*N*-methyl-*N*-(thiophen-3-ylmethyl)methanamine (JG-1): Compound **9** was Boc-deprotected with trifluoroacetic acid in dichloromethane following the general synthetic procedure for Boc-deprotection and subsequently reacted with 3-(bromomethyl)thiophene and potassium carbonate in acetonitrile following the general procedure for amine alkylation.[3] The product was transformed into a salt using the general procedure for making HCl salts.[3] Spectral data were consistent with previous reports.[3] ^{1}H NMR (400 MHz, methanol-d$_4$) δ 7.39 – 7.34 (m, 1H), 7.31 – 7.19 (m, 3H), 7.14 – 7.01 (m, 3H), 3.56 (s, 2H), 3.07 (d, J = 11.2 Hz, 2H), 2.93 – 2.84 (m, 2H), 2.67 – 2.58 (m, 2H), 2.27 – 2.21 (m, 5H), 2.17 (t, J =11.5 Hz, 2H), 1.85 (d, J = 13.1 Hz, 2H), 1.70 – 1.57 (m, 1H), 1.40 – 1.17 (m, 2H). HRMS *m/z* calculated for $C_{20}H_{28}FN_2S$ $[M+H]^+$: 347.1952, found: 347.1945.

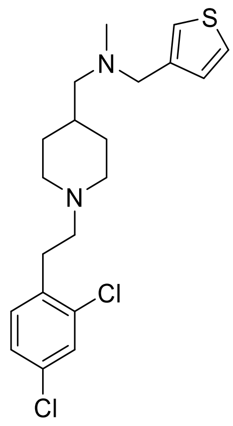

1-(1-(2,4-Dichlorophenethyl)piperidin-4-yl)-*N*-methyl-*N*-(thiophen-3-ylmethyl)methanamine--1-(1-(2-([1,1'-biphenyl]-4-yl)ethyl)piperidin-4-yl)-*N*-methyl-*N*-(thiophen-3-ylmethyl)methanamine (NDM-295): Compound **11a** was Boc-deprotected with trifluoroacetic acid in dichloromethane following the general synthetic procedure for Boc-deprotection and subsequently reacted with 3-(bromomethyl)thiophene and potassium carbonate in acetonitrile following the general procedure for amine alkylation.[3] The product was transformed into a salt using the general procedure for making HCl salts.[3] Spectral data were consistent with previous reports.[3] ^{1}H NMR (400 MHz, methanol-d$_4$) δ 7.82 (s, 1H), 7.68 – 7.62 (m, 1H), 7.54 (d, J = 1.9 Hz, 1H), 7.45 (d, J = 8.3 Hz, 1H), 7.41 – 7.33 (m, 2H), 4.52 (d, J = 13.3 Hz, 1H), 4.42 (d, J = 13.4 Hz, 1H), 3.76 (d, J = 12.3 Hz, 2H), 3.58 – 3.40 (m, 1H), 3.36 (d, J = 2.4 Hz, 1H), 3.31 – 3.25 (m, 2H), 3.25 – 3.08 (m, 4H), 2.91 (s, 3H), 2.40 – 2.26 (m, 1H), 2.17 (dd, J = 2.7, 1.5 Hz, 2H), 1.69 - 1.60 (m, 2H). HRMS *m/z* calculated for $C_{20}H_{27}Cl_2N_2S$ [M+H]$^+$: 397.1267, found: 397.1258.

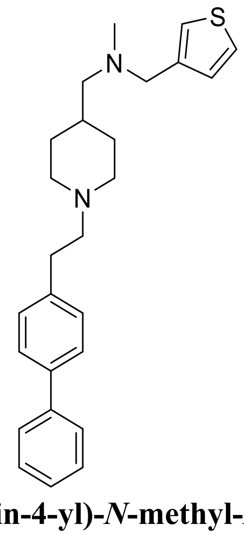

1-(1-(2,4-Dichlorophenethyl)piperidin-4-yl)-*N*-methyl-*N*-(thiophen-3-ylmethyl)methanamine--1-(1-(2-([1,1'-biphenyl]-4-yl)ethyl)piperidin-4-yl)-*N*-methyl-*N*-(thiophen-3-ylmethyl)methanamine (NDM-323): Compound **11c** was Boc-deprotected with trifluoroacetic acid in dichloromethane following the general synthetic procedure for Boc-deprotection and subsequently reacted with 3-(bromomethyl)thiophene and potassium carbonate in acetonitrile following the general procedure for amine alkylation.[3] The product was transformed

into a salt using the general procedure for making HCl salts.[3] Spectral data were consistent with previous reports.[3] ^{1}H NMR (400 MHz, methanol-d$_4$) δ 7.77 (s, 1H), 7.66 – 7.57 (m, 5H), 7.48 – 7.36 (m, 4H), 7.35 – 7.28 (m, 2H), 4.50 (d, *J* = 12.7 Hz, 1H), 4.39 (d, *J* = 12.5 Hz, 1H), 3.75 (d, *J* = 12.4 Hz, 2H), 3.40 (dd, *J* = 10.5, 6.4 Hz, 2H), 3.19 – 3.06 (m, 6H), 2.89 (s, 3H), 2.34 – 2.21 (m, 1H), 2.14 (d, *J* = 13.5 Hz, 2H), 1.70 – 1.49 (m, 2H). HRMS (ESI) *m/z* calculated for $C_{26}H_{33}N_2S$ $[M+H]^+$: 405.2359, found 405.2357.

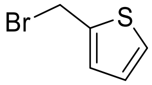

2-(Bromomethyl) thiophene (S1): Thiophen-2-ylmethanol reacted with phosphorus tribromide in diethyl ether following the general procedure for formation of alkyl bromides. The reaction was concentrated to a yellow oil and was prone to decomposition and was used with no further purification.[4]

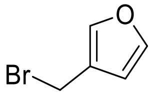

3-(Bromomethyl)furan (S2): Furan-3-carbaldehyde (10.4 mmol) was dissolved in methanol (20 mL) and cooled to 0 °C. Sodium borohydride (5.2 mmol) was added to the solution and was stirred for 15 minutes. The reaction was quenched with di-H_2O (20 mL) and extracted with ethyl acetate (20 mL x 3). The organic layers were combined and washed with brine (20 mL). The organic solution was dried over anhydrous sodium sulfate, filtered, and evaporated in vacuo. The resulting crude alcohol was dissolved in diethyl ether and reacted with phosphorus tribromide following the general procedure for formation of alkyl bromides.[5]

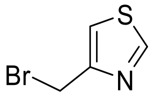

4-(Bromomethyl)thiazole (S3): Thiazol-4-ylmethanol was reacted with phosphorus tribromide in diethyl ether following the general procedure for formation of alkyl bromides. The product was prone to decomposition and was used with no further purification.[6]

Characterization of novel compounds

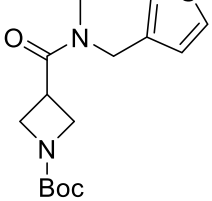

***tert*-Butyl 3-(methyl(thiophen-3-ylmethyl)carbamoyl)azetidine-1-carboxylate (3):** Compound **1** was reacted with *N*-methyl-1-(thiophen-3-yl)methanamine (**2**) and EDC following the general synthetic procedure for *N*-methyl-1-(thiophen-3-yl)methanamine addition. The product was

isolated as a rotameric mixture (60:40) as a yellow oil (87%). ^{1}H NMR (400 MHz, methanol-d$_4$) δ 7.46 - 7.35 (m, 1H, both rotamers), 7.27 - 7.20 (m, 1H, both rotamers), 7.00 – 6.95 (m, 1H, both rotamers), 4.57 (s, 1.2H, major rotamer), 4.48 (s, 0.8H, minor rotamer), 4.13 – 3.98 (m, 4H, both rotamers), 3.83 – 3.66 (m, 1H, both roamers), 2.97 (s, 1.2H, minor rotamer), 2.86 (s, 1.8H, major rotamer), 1.44 (s, 5.4H, major rotamer), 1.42 (s, 3.6H, minor rotamer); ^{13}C NMR (100.6 MHz, methanol-d$_4$) δ 171.9, 156.6, 137.8, 127.2, 126.2, 122.7, 79.8, 45.9, 33.4, 30.8, 30.3, 27.4; IR ν_{max} (cm^{-1}) 2943, 1691, 1136, 758; UV (λ_{max} nm) 250; LC Trace: 100%; HRMS *m/z* calculated for $C_{15}H_{23}N_2O_3S$ [M+H]$^+$: 311.1424, found: 311.1436.

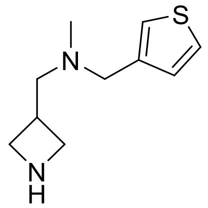

1-(Azetidin-3-yl)-*N*-methyl-*N*-(thiophen-3-ylmethyl)methanamine (4): Compound **3** was Boc-deprotected with trifluoroacetic acid in dichloromethane following the general synthetic procedure for Boc-deprotection and subsequently the amide was reduced with LAH following the general synthetic procedure for amide reduction to tertiary amine. The product was a yellow oil (20%). ^{1}H NMR (400 MHz, chloroform-d) δ 7.46 – 7.44 (m, 1H), 7.26 – 7.24 (m, 1H), 7.21 – 7.18 (m, 1H), 4.32 – 4.16 (m, 3H), 3.74 – 3.63 (m, 3H), 2.74 – 2.70 (m, 1H), 2.45 – 2.43 (m, 2H), 2.33 (s, 3H); ^{13}C NMR (100.6 MHz, chloroform-d) δ 137.5, 127.4, 126.8, 126.0, 49.2, 47.9, 34.7, 34.3, 33.9; IR ν_{max} (cm^{-1}) 2958, 2530, 1125, 710; UV (λ_{max} nm) 248; LC Trace: 100%; HRMS *m/z* calculated for $C_{10}H_{17}N_2S$ [M+H]$^+$: 197.1107, found 197.1103.

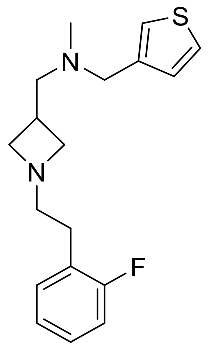

1-(1-(2-Fluorophenethyl)azetidin-3-yl)-*N*-methyl-*N*-(thiophen-3-ylmethyl)methanamine (NDM-506): Compound **4** was reacted with 1-(2-bromoethyl)-2-fluorobenzene and potassium carbonate in acetonitrile following the general procedure for amine alkylation. The product was transformed into a salt using the general procedure for making HCl salts. The product presented as a tan solid (70%). ^{1}H NMR (400 MHz, chloroform-d) δ 7.49 - 7.42 (m, 3H), 7.30- 7.27 (m, 2H), 7.20 – 7.16 (m, 1H), 7.09-7.00 (m, 1H), 4.20 – 4.11 (m, 3H), 3.75-3.68 (m, 4H) 2.94 – 2.87 (m, 1H), 2.80 (d, *J* = 8.0 Hz, 2H), 2.39 (s, 3H), 1.40 – 1.38 (m, 3H); ^{13}C NMR (100.6 MHz, chloroform-d) δ 162.5 (d, *J* = 195.2 Hz), 132.2, 131.0, 130.7 (d, *J* = 6.0 Hz), 130.3 (d, *J* = 23.1 Hz), 128.9, 125.9 (d, *J* = 3.0 Hz), 124.3, 123.9, 116.6 (d, *J* = 17.1 Hz), 60.8, 57.7, 55.6, 53.1, 41.1, 30.7, 28.5; IR ν_{max} (cm^{-1}) 2930, 1520, 1350, 724; UV (λ_{max} nm) 256; LC Trace: 100%; HRMS *m/z* calculated for $C_{18}H_{24}FN_2S$ [M+H]$^+$: 319.1639, found: 319.1645.

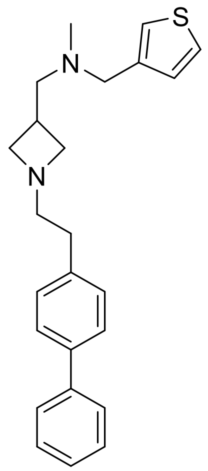

1-(1-(2-([1,1'-Biphenyl]-4-yl)ethyl)azetidin-3-yl)-*N*-methyl-*N*-(thiophen-3-ylmethyl)methanamine (NDM-507): Compound **4** was reacted with 2-([1,1'-biphenyl]-4-yl)ethyl methanesulfonate (**7**) and potassium carbonate in acetonitrile following the general procedure for amine alkylation. The product was transformed into a salt using the general procedure for making HCl salts. The product presented as a white solid (80%). ^{1}H NMR (400 MHz, chloroform-d) δ 7.49 – 7.30 (m, 5H), 7.27 – 7.23 (m, 3H), 7.17 – 7.13 (m, 4H), 4.36 – 4.31 (m, 3H), 3.40 – 3.38 (m, 3H), 3.00 – 2.74 (m, 4H), 2.43 (s, 3H), 2.10 – 2.08 (m, 1H), 1.35 - 1.16 (m, 2H); ^{13}C NMR (100.6 MHz, chloroform-d) δ 140.6, 140.0, 139.8, 135.5, 129.5, 128.9, 128.4, 127.4, 127.4, 127.0, 125.6, 122.6, 70.3, 42.4, 37.3, 35.3, 31.0, 28.5, 27.2; IR ν_{max} (cm^{-1}) 2930, 2509, 1414, 673; UV (λ_{max} nm) 258; LC Trace: 100%; HRMS *m/z* calculated for $C_{24}H_{29}N_2S$ [M+H]$^+$: 377.2046, found: 377.2065.

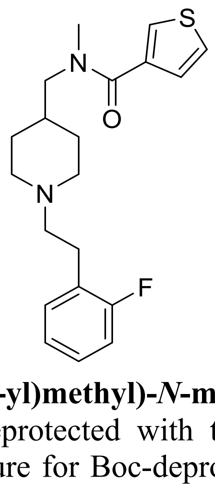

***N*-((1-(2-Fluorophenethyl)piperidin-4-yl)methyl)-*N*-methylthiophene-3-carboxamide (NDM-29):** Compound **9** was Boc-deprotected with trifluoroacetic acid in dichloromethane following the general synthetic procedure for Boc-deprotection. Subsequently, the intermediate amine (0.97 mmol) reacted with thiophene-3-carboxylic acid (0.80 mmol), triethylamine (0.80 mmol), hydroxybenzotriazole (HOBt, 0.80 mmol), and 1-ethyl-3- (3-dimethylaminopropyl) carbodiimide hydrochloride (EDC*HCl, 1.21 mmol) in dichloromethane (7 mL). The reaction was stirred at room temperature overnight. The reaction mixture was then washed sequentially with 1 M aqueous hydrochloric acid (2x15 mL), saturated aqueous sodium bicarbonate (3x15 mL), and saturated aqueous sodium chloride (3x15 mL). The organic layer was dried over anhydrous sodium

sulfate, filtered, evaporated under reduced pressure, and purified via flash chromatography using 2% methanol, 2% triethylamine, and 96% chloroform. The product was transformed into a salt using the general procedure for making HCl salts. The product presented as an off-white solid (40%). ^{1}H NMR (400 MHz, methanol-d$_4$) δ 7.78 (s, 1H), 7.59 – 7.51 (m, 1H), 7.42 – 7.26 (m, 3H), 7.24 – 7.11 (m, 2H), 3.76 (d, *J* = 11.3 Hz, 1H), 3.55 (d, *J* = 7.7 Hz, 1H), 3.52 – 3.42 (m, 2H), 3.23 – 3.04 (m, 6H), 2.26 – 2.13 (m, 1H), 2.08 (d, *J* = 12.8 Hz, 1H), 1.61 (d, *J* = 14.7 Hz, 2H), 1.36 – 1.26 (m, 4H); ^{13}C NMR (100.6 MHz, methanol-d$_4$) δ 166.5, 161.1 (d, *J* = 244.9 Hz), 143.2, 130.7 (d, *J* = 4.4 Hz), 128.8, 126.7 (d, *J* = 1.8 Hz), 125.8 (d, *J* = 18.5 Hz), 125.3, 119.2, 114.8 (d, *J* = 22.6 Hz) 109.5, 58.5, 58.2, 52.7, 52.6, 28.8, 28.7, 25.5; ^{19}F NMR (376.4 Hz, methanol-d$_4$) -121.0; UV (λ_{max} nm) 259; LC Trace: 100%; HRMS (ESI) *m/z* calculated for $C_{20}H_{26}FN_2OS$ $[M+H]^+$: 361.1744, found 361.1736.

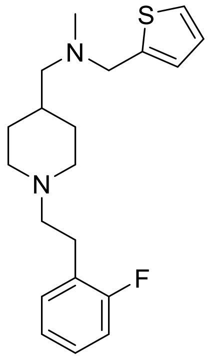

1-(1-(2-Fluorophenethyl)piperidin-4-yl)-*N*-methyl-*N*-(thiophen-2-ylmethyl)methanamine (NDM-559): Compound **9** was Boc-deprotected with trifluoroacetic acid in dichloromethane following the general synthetic procedure for Boc-deprotection and subsequently reacted with 2-(bromomethyl)thiophene and potassium carbonate in acetonitrile following the general procedure for amine alkylation. The product was transformed into a salt using the general procedure for making HCl salts. The product presented as a yellow oil (33%). ^{1}H NMR (400 MHz, methanol-d$_4$) δ 7.21 – 7.18 (m, 1H), 7.17 – 7.09 (m, 2H), 7.02 – 6.90 (m, 2H), 6.85 – 6.81 (m, 2H), 3.61 (s, 2H), 2.97 (d, *J* = 11.5 Hz, 2H), 2.82 – 2.74 (m, 2H), 2.58 – 2.49 (m, 2H), 2.17 – 2.12 (m, 5H), 2.08 (t, *J* = 11.5 Hz, 2H), 1.75 (d, *J* = 13.0 Hz, 2H), 1.60 – 1.45 (m, 1H), 1.19 – 1.07 (m, 2H); ^{13}C NMR (100.6 MHz, methanol-d$_4$) δ 163.1 (d, *J* = 246.5 Hz), 130.4 (d, *J* = 8.3 Hz), 129.6, 129.0, 128.8, 127.5 (d, *J* = 4.1 Hz), 124.3 (d, *J* = 2.9 Hz), 115.2 (d, *J* = 21.8 Hz), 113.7 (d, *J* = 21.2 Hz), 59.4, 57.4, 54.2, 51.8, 51.7, 39.7, 29.5, 29.3, 27.1, 27.0; ^{19}F NMR (376.4 Hz, methanol-d$_4$) δ -114.8; UV (λ_{max} nm) 248; LC Trace: 100%; HRMS (ESI) *m/z* calculated for $C_{20}H_{28}FN_2S$ $[M+H]^+$: 347.1952, found 347.1952.

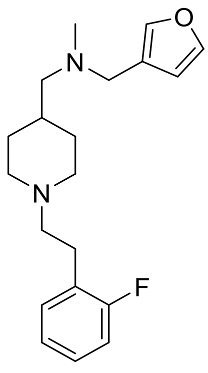

1-(1-(2-Fluorophenethyl)piperidin-4-yl)-*N*-(furan-3-ylmethyl)-*N*-methylmethanamine (NDM-560): Compound **9** was Boc-deprotected with trifluoroacetic acid in dichloromethane following the general synthetic procedure for Boc-deprotection and subsequently reacted with 3-(bromomethyl)furan and potassium carbonate in acetonitrile following the general procedure for amine alkylation. The product was transformed into a salt using the general procedure for making HCl salts. The product presented as a white solid (28%). ^{1}H NMR (400 MHz, methanol-d$_4$) δ 7.48 – 7.46 (m, 1H), 7.43 (s, 1H), 7.32 – 7.19 (m, 2H), 7.13 – 7.02 (m, 2H), 6.43 (s, 1H), 3.41 (s, 2H), 3.06 (d, *J* = 11.4, 2H), 2.92 – 2.85 (m, 2H), 2.64 – 2.57 (m, 2H), 2.27 – 2.19 (m, 5H), 2.14 (t, *J* = 11.6, 2H), 1.83 (d, *J* = 12.9, 2H), 1.68 – 1.55 (m, 1H), 1.32 – 1.17 (m, 2H) ; ^{13}C NMR (100.6 MHz, methanol-d$_4$) δ 161.3 (d, *J* = 187.1 Hz), 159.8, 130.8 (d, *J* = 4.1 Hz), 129.5, 129.3 (d, *J* = 8.2 Hz), 125.4, 124.5 (d, *J* = 3.5 Hz), 122.8 (d, *J* = 16.0 Hz), 115.2 (d, *J* = 21.9 Hz), 56.9, 56.2, 52.4, 34.6, 33.2, 32.1, 27.1, 23.7, 23.6; ^{19}F NMR (376.4 Hz, methanol-d$_4$) δ – 114.9; UV (λ_{max} nm) 268; LC Trace: 100%; HRMS (ESI) *m/z* calculated for $C_{20}H_{28}FN_2O$ [M+H]$^+$: 331.2180, found 331.2188.

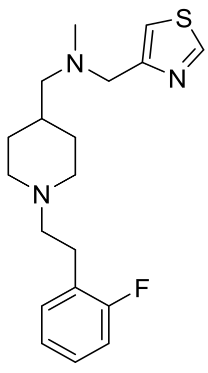

1-(1-(2-Fluorophenethyl)piperidin-4-yl)-*N*-methyl-*N*-(thiazol-4-ylmethyl)methanamine (NDM-597): Compound **9** was Boc-deprotected with trifluoroacetic acid in dichloromethane following the general synthetic procedure for Boc-deprotection and subsequently reacted with 4-(bromomethyl)thiazole and potassium carbonate in acetonitrile following the general procedure for amine alkylation. The product was transformed into a salt using the general procedure for making HCl salts. The product presented as an off-white solid (23%). ^{1}H NMR (400 MHz, methanol-d$_4$) δ 8.99 (s, 1H), 7.48 (s, 1H), 7.35 – 7.22 (m, 2H), 7.13 (t, *J* = 7.4 Hz, 1H), 7.07 (t, *J* = 9.3 Hz, 1H), 3.76 (s, 2H), 3.16 (d, *J* = 11.6 Hz, 2H), 2.97 – 2.89 (m, 2H), 2.78 – 2.68 (m, 2H), 2.37 – 2.24 (m, 7H), 1.91 (d, *J* = 13.0 Hz, 2H), 1.77 – 1.62 (m, 1H), 1.34 – 1.22 (m. 2H); ^{13}C NMR (100.6 MHz, methanol-d$_4$) δ 163.0 (d, *J* = 245.3 Hz), 138.9 (d, *J* = 6.6 Hz), 130.4 (d, *J* = 8.2 Hz), 129.6, 128.8, 127.5, 124.3 (d, *J* = 3.1 Hz), 115.2 (d, *J* = 21.7 Hz), 113.7 (d, *J* = 21.1 Hz), 59.4,

57.3, 54.2, 51.8, 39.7, 29.5, 29.3, 27.2; ^{19}F NMR (376.4 Hz, methanol-d$_4$) δ – 115.0; UV (λ_{max} nm) 251; LC Trace: 96%; HRMS (ESI) *m/z* calculated for $C_{19}H_{27}FN_3S$ $[M+H]^+$: 348.1784, found 348.1782.

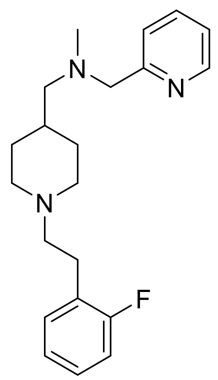

1-(1-(2-Fluorophenethyl)piperidin-4-yl)-*N*-methyl-*N*-(pyridin-2-ylmethyl)methanamine (NDM-73): Compound **9** was Boc-deprotected with trifluoroacetic acid in dichloromethane following the general synthetic procedure for Boc-deprotection and subsequently reacted with 2-(bromomethyl)pyridine and potassium carbonate in acetonitrile following the general procedure for amine alkylation. The product was transformed into a salt using the general procedure for making HCl salts. The product presented as a brown solid (58%). ^{1}H NMR (400 MHz, methanol-d$_4$) δ 8.47 (dd, *J* = 5.0, 0.9 Hz, 1H), 7.84 (td, *J* = 7.7, 1.8 Hz, 1H), 7.59 (d, *J* = 7.8 Hz, 1H), 7.33 (ddd, *J* = 7.5, 5.0, 1.2 Hz, 1H), 7.30 – 7.20 (m, 2H), 7.11 (td, *J* = 7.5, 1.2 Hz, 1H), 7.05 (ddd, *J* = 9.5, 8.2, 1.2 Hz, 1H), 3.65 (s, 2H), 3.04 (d, *J* = 10.7 Hz, 2H), 2.93 – 2.82 (m, 2H), 2.65 – 2.54 (m, 2H), 2.29 (d, *J* = 7.2 Hz, 2H), 2.26 (s, 3H), 2.12 (td, *J* = 11.9, 2.5 Hz, 2H), 1.91 – 1.83 (m, 2H), 1.67 – 1.59 (m, 1H), 1.28 – 1.16 (m, 2H); ^{13}C NMR (100.6 MHz, methanol-d$_4$) δ 161.1 (d, *J* = 243.5 Hz), 159.2, 147.9, 137.2, 130.8 (d, *J* = 4.8 Hz), 128.0 (d, *J* = 8.2 Hz), 126.2 (d, *J* = 16.0 Hz), 124.1 (d, *J* = 3.5 Hz), 123.6, 122.4, 114.8 (d, *J* = 22.3 Hz), 63.4, 63.3, 58.6, 33.4, 29.9, 29.8, 25.7, 25.7; IR ν_{max} (cm^{-1}) 2924, 2793, 1588, 1491, 1228, 753; UV (λ_{max} nm) 258; LC Trace: 100%; HRMS (ESI) *m/z* calculated for $C_{21}H_{29}FN_3$ $[M+H]^+$: 342.2340, found 342.2332.

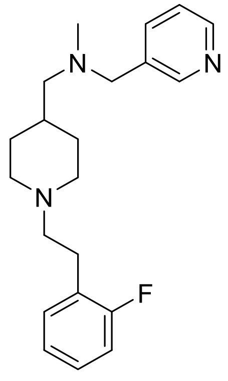

1-(1-(2-Fluorophenethyl)piperidin-4-yl)-*N*-methyl-*N*-(pyridin-3-ylmethyl)methanamine (NDM-599): Compound **9** was Boc-deprotected with trifluoroacetic acid in dichloromethane following the general synthetic procedure for Boc-deprotection and subsequently reacted with 3-(bromomethyl)pyridine and potassium carbonate in acetonitrile following the general procedure for amine alkylation. The product was transformed into a salt using the general procedure for

making HCl salts. The product presented as a brown solid (58%). ^{1}H NMR (400 MHz, methanol-d_4) δ 9.33 (d, *J* = 2.0 Hz, 1H), 9.08 – 8.94 (m, 2H), 8.24 (dd, *J* = 8.2, 5.8 Hz, 1H), 7.45 – 7.27 (m, 2H), 7.24 – 7.04 (m, 2H), 3.75 (d, *J* = 12.3 Hz, 2H), 3.53 – 3.42 (m, 1H), 3.38 – 3.31 (m, 5H), 3.24 – 3.09 (m, 4H), 2.95 (s, 3H), 2.49 – 2.14 (m, 3H), 1.79 – 1.62 (m, 2H); ^{13}C NMR (100.6 MHz, methanol-d_4) δ 161.1 (d, *J* = 244.4 Hz), 149.9, 144.6, 142.9, 130.9, 129.8, 129.2 (d, *J* = 8.1 Hz), 127.6, 124.5 (d, *J* = 3.7 Hz), 122.9 (d, *J* = 15.8 Hz), 115.2 (d, *J* = 21.9 Hz), 60.5, 56.3, 55.9, 51.7, 39.4, 29.3, 27.3, 23.6; IR ν_{max} (cm^{-1}) 3425, 3354, 3014, 2938, 2608, 2492, 1454, 759; UV (λ_{max} nm) 256; LC Trace: 100%; HRMS (ESI) *m/z* calculated for $C_{21}H_{29}FN_3$ [M+H]$^+$: 342.2340, found 342.2350.

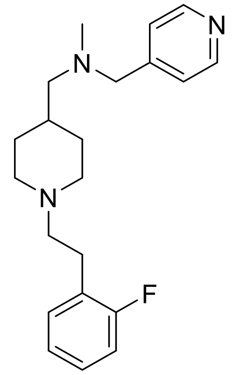

1-(1-(2-Fluorophenethyl)piperidin-4-yl)-*N*-methyl-*N*-(pyridin-4-ylmethyl)methanamine (NDM-72): Compound **9** was Boc-deprotected with trifluoroacetic acid in dichloromethane following the general synthetic procedure for Boc-deprotection and subsequently reacted with 4-(bromomethyl)pyridine and potassium carbonate in acetonitrile following the general procedure for amine alkylation. The product was transformed into a salt using the general procedure for making HCl salts. The product presented as an off-white solid (62%). ^{1}H NMR (400 MHz, methanol-d_4) δ 8.50 – 8.41 (m, 2H), 7.48 – 7.38 (m, 2H), 7.32 – 7.18 (m, 2H), 7.09 (td, *J* = 7.5, 1.2 Hz, 1H), 7.04 (ddd, *J* = 9.6, 8.2, 1.2 Hz, 1H), 3.55 (s, 2H), 3.11 – 3.02 (m, 2H), 2.91 – 2.83 (m, 2H), 2.66 – 2.53 (m, 2H), 2.25 (d, *J* = 7.2 Hz, 2H), 2.21 (s, 3H), 2.13 (td, *J* = 11.9, 2.5 Hz, 2H), 1.91 – 1.83 (m, 2H), 1.63 (ttt, *J* = 18.3, 7.2, 3.9 Hz, 1H), 1.28 – 1.17 (m, 2H); ^{13}C NMR (100.6 MHz, methanol-d_4) δ 161.1 (d, *J* = 243.5 Hz), 150.4, 148.8, 148.5, 130.8 (d, *J* = 4.8 Hz), 130.0, 127.9 (d, *J* = 8.1 Hz), 126.4 (d, *J* = 15.8 Hz), 124.2, 124.0 (d, *J* = 3.5 Hz), 114.8 (d, *J* = 22.3 Hz), 63.4, 61.1, 58.7, 53.1, 33.5, 29.9, 25.8, 25.8; IR ν_{max} (cm^{-1}) 3406, 3022, 2929, 2504, 1602, 1493, 1229, 956, 759; UV (λ_{max} nm) 288; LC Trace: 100%; HRMS (ESI) *m/z* calculated for $C_{21}H_{29}FN_3$ [M+H]$^+$: 342.2340, found 342.2332.

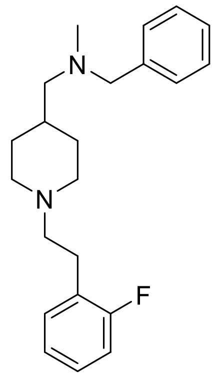

***N*-Benzyl-1-(1-(2-fluorophenethyl)piperidin-4-yl)-*N*-methylmethanamine (NDM-43):** Compound **9** was Boc-deprotected with trifluoroacetic acid in dichloromethane following the general synthetic procedure for Boc-deprotection and subsequently reacted with (bromomethyl)benzene and potassium carbonate in acetonitrile following the general procedure for amine alkylation. The product was transformed into a salt using the general procedure for making HCl salts. The product presented as a white solid (40%). ^{1}H NMR (500 MHz, methanol-d$_4$) δ 7.34 – 7.31 (m, 4H), 7.31 – 7.21 (m, 3H), 7.11 (td, *J* = 7.4, 1.3 Hz, 1H), 7.06 (ddd, *J* = 10.4, 8.1, 1.3 Hz, 1H), 3.51 (s, 2H), 3.11 – 3.03 (m, 2H), 2.92 – 2.85 (m, 2H), 2.66 – 2.58 (m, 2H), 2.25 (d, *J* = 7.1 Hz, 2H), 2.21 (s, 3H), 2.20 – 2.12 (m, 2H), 1.91 – 1.83 (m, 2H), 1.71 – 1.60 (m, 1H), 1.30 – 1.16 (m, 2H); ^{13}C NMR (126 MHz, methanol-d$_4$) δ 161.1 (d, *J* = 245.5 Hz), 138.6, 130.7 (d, *J* = 5.0 Hz), 128.9, 127.9 (d, *J* = 10.1 Hz), 127.8, 126.8, 126.4 (d, *J* = 16.0 Hz), 124.0, 114.8 (d, *J* = 22.3 Hz), 63.0, 62.3, 58.7, 53.1, 33.3, 29.9, 27.3, 25.7; IR ν_{max} (cm^{-1}) 3418, 2935, 2500, 1493, 1452, 1232, 743, 697; UV (λ_{max} nm) 256; LC Trace: 100%; HRMS (ESI) *m/z* calculated for $C_{22}H_{30}FN_2$ [M+H]$^+$: 340.2254, found 340.2249.

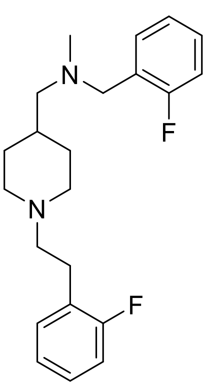

***N*-(2-Fluorobenzyl)-1-(1-(2-fluorophenethyl)piperidin-4-yl)-*N*-methylmethanamine (NDM-52):** Compound **9** was Boc-deprotected with trifluoroacetic acid in dichloromethane following the general synthetic procedure for Boc-deprotection and subsequently reacted with 1-(bromomethyl)-2-fluorobenzene and potassium carbonate in acetonitrile following the general procedure for amine alkylation. The product was transformed into a salt using the general procedure for making HCl salts. The product presented as a white solid (56%). ^{1}H NMR (500 MHz, methanol-d$_4$) δ 7.69 – 7.64 (m, 1H), 7.62 – 7.57 (m, 1H), 7.40 – 7.29 (m, 4H), 7.18 (td, *J* = 7.5, 1.2 Hz, 1H), 7.13 (ddd, *J* = 10.5, 8.2, 1.2 Hz, 1H), 4.64 (d, *J* = 13.4 Hz, 1H), 4.37 (d, *J* = 13.3 Hz, 1H), 3.76 (d, *J* = 12.3 Hz, 2H), 3.39 – 3.33 (m, 2H), 3.28 – 3.25 (m, 1H), 3.22 (d, *J* = 6.6 Hz, 1H), 3.20 – 3.13 (m, 4H), 2.89 (d, *J* = 0.8 Hz, 3H), 2.37 (t, *J* = 3.5 Hz, 1H), 2.17 (t, *J* = 14.7 Hz, 2H), 1.74 – 1.61 (m, 2H); ^{13}C NMR (126 MHz, methanol-d$_4$) δ 162.0 (d, *J* = 248.3 Hz), 161.3 (d, *J* = 244.4 Hz), 133.7, 133.2 (d, *J* = 8.6 Hz), 131.1, 129.5 (d, *J* = 8.2 Hz), 125.3 (d, *J* = 3.5 Hz), 124.7 (d, *J* = 3.5 Hz), 116.4 (d, *J* = 14.4 Hz), 116.1 (d, *J* = 21.7 Hz), 115.9, 115.4 (d, *J* = 21.7 Hz), 60.4, 56.5, 53.3, 51.9, 29.5, 27.3, 23.8, 23.8; IR ν_{max} (cm^{-1}) 3393, 2929, 2476, 1494, 1455, 1231, 751; UV (λ_{max} nm) 256; LC Trace: 100%; HRMS (ESI) *m/z* calculated for $C_{22}H_{29}F_2N_2$ [M+H]$^+$: 359.2293, found 359.2296.

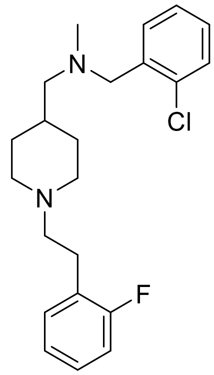

***N*-(2-Chlorobenzyl)-1-(1-(2-fluorophenethyl)piperidin-4-yl)-*N*-methylmethanamine (NDM-51):** Compound **9** was Boc-deprotected with trifluoroacetic acid in dichloromethane following the general synthetic procedure for Boc-deprotection and subsequently reacted with 1-(bromomethyl)-2-chlorobenzene and potassium carbonate in acetonitrile following the general procedure for amine alkylation. The product was transformed into a salt using the general procedure for making HCl salts. The product presented as a white solid (21%). ^{1}H NMR (400 MHz, methanol-d$_4$) δ 7.49 (dd, *J* = 7.4, 2.0 Hz, 1H), 7.37 (dd, *J* = 7.6, 1.8 Hz, 1H), 7.32 – 7.21 (m, 4H), 7.12 (td, *J* = 7.5, 1.2 Hz, 1H), 7.07 (ddd, *J* = 9.5, 8.1, 1.2 Hz, 1H), 3.60 (s, 2H), 3.26 (d, *J* = 12.1 Hz, 2H), 2.96 (dt, *J* = 10.7, 6.4 Hz, 2H), 2.92 – 2.84 (m, 2H), 2.49 (td, *J* = 12.3, 2.8 Hz, 2H), 2.32 (d, *J* = 7.2 Hz, 2H), 2.23 (s, 3H), 2.01 – 1.93 (m, 2H), 1.75 (dt, *J* = 11.0, 7.5, Hz, 1H), 1.31 – 1.25 (m, 2H); ^{13}C NMR (100.6 MHz, methanol-d$_4$) δ 161.1 (d, *J* = 243.8 Hz), 136.4, 134.1, 131.0, 130.8 (d, *J* = 4.5 Hz), 129.1, 128.5 (d, *J* = 8.2 Hz), 128.2, 126.4, 125.0 (d, *J* = 15.8 Hz), 124.2 (d, *J* = 3.6 Hz), 114.9 (d, *J* = 22.1 Hz), 62.9, 59.0, 57.6, 52.8, 41.7, 32.7, 29.0, 25.0; IR ν_{max} (cm^{-1}) 3398, 2926, 2636, 2565, 1444, 1231, 753; UV (λ_{max} nm) 258; LC Trace: 100%; HRMS (ESI) *m/z* calculated for $C_{22}H_{29}ClFN_2$ [M+H]$^+$: 375.1998, found 375.2003.

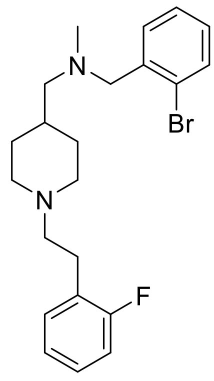

***N*-(2-Bromobenzyl)-1-(1-(2-fluorophenethyl)piperidin-4-yl)-*N*-methylmethanamine (NDM-601):** Compound **9** was Boc-deprotected with trifluoroacetic acid in dichloromethane following the general synthetic procedure for Boc-deprotection and subsequently reacted with 1-(bromomethyl)-2-bromobenzene and potassium carbonate in acetonitrile following the general procedure for amine alkylation. The product was transformed into a salt using the general procedure for making HCl salts. The product presented as a white solid (36%). ^{1}H NMR (400 MHz, methanol-d$_4$) δ 7.81 (dt, *J* = 8.0, 1.8 Hz, 2H), 7.55 (td, *J* = 7.5, 1.3 Hz, 1H), 7.50 – 7.38 (m, 2H), 7.38 – 7.31 (m, 1H), 7.22 – 7.11 (m, 2H), 4.81 (d, *J* = 13.4 Hz, 1H), 4.48 (d, *J* = 13.3 Hz, 1H), 3.77 (d, *J* = 12.2 Hz, 2H), 3.42 – 3.33 (m, 4H), 3.26 – 3.15 (m, 4H), 2.95 (s, 3H), 2.51 – 2.40 (m, 1H), 2.25 (dt, *J* = 14.1, 3.0 Hz, 2H), 1.85 – 1.64 (m, 2H); ^{13}C NMR (100.6 MHz, methanol-

d_4) δ 161.1 (d, J = 244.5 Hz), 133.7, 133.6, 132.1, 130.9, (d, J = 4.2 Hz), 129.3, 129.1 (d, J = 9.0 Hz), 128.4, 125.8, 124.6, 123.0 (d, J = 15.3 Hz), 115.2 (d, J = 21.8 Hz), 61.1, 59.4, 56.3, 51.7, 40.4, 29.3, 27.2, 23.6; IR ν_{max} (cm^{-1}) 3404, 2927, 2510, 1511, 1453, 1230, 752; UV (λ_{max} nm) 252; LC Trace: 100%; HRMS (ESI) *m/z* calculated for $C_{22}H_{29}BrFN_2$ $[M+H]^+$: 419.1493, found 419.1498.

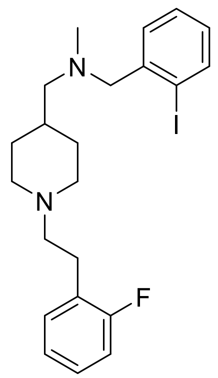

1-(1-(2-Fuorophenethyl)piperidin-4-yl)-*N*-(2-iodobenzyl)-*N*-methylmethanamine (NDM-603): Compound **9** was Boc-deprotected with trifluoroacetic acid in dichloromethane following the general synthetic procedure for Boc-deprotection and subsequently reacted with 1-(bromomethyl)-2-iodobenzene and potassium carbonate in acetonitrile following the general procedure for amine alkylation. The product was transformed into a salt using the general procedure for making HCl salts. The product presented as a white solid (23%). ^{1}H NMR (400 MHz, methanol-d_4) δ 7.86 (dd, J = 7.9, 1.3 Hz, 1H), 7.44 (dd, J = 7.7, 1.8 Hz, 1H), 7.39 – 7.23 (m, 3H), 7.16 – 7.05 (m, 2H), 7.00 (td, J = 7.6, 1.9 Hz, 1H), 3.54 (s, 2H), 3.21 (d, J = 11.8 Hz, 2H), 2.95 (dd, J = 10.8, 5.7 Hz, 2H), 2.82 (dd, J = 11.2, 5.6 Hz, 2H), 2.43 (t, J = 11.9 Hz, 2H), 2.32 (d, J = 7.3 Hz, 2H), 2.25 (s, 3H), 1.97 (d, J = 14.5 Hz, 2H), 1.73 (ddd, J = 11.2, 7.3, 3.8 Hz, 1H), 1.33 – 1.22 (m, 2H); ^{13}C NMR (100.6 MHz, methanol-d_4) δ 161.1 (d, J = 243.8 Hz), 141.2, 139.4, 130.8 (d, J = 4.6 Hz), 130.5, 128.5, 128.3 (d, J = 8.1 Hz), 127.7, 125.3 (d, J = 15.9 Hz), 124.2 (d, J = 3.6 Hz), 114.9 (d, J = 22.2 Hz), 99.9, 66.4, 62.6, 57.9, 52.8, 41.5, 32.9, 29.3, 25.1; IR ν_{max} (cm^{-1}) 3389, 2930, 2549, 1493, 1230, 750; UV (λ_{max} nm) 252; LC Trace: 100%; HRMS (ESI) *m/z* calculated for $C_{22}H_{29}FIN_2$ $[M+H]^+$: 467.1354, found 467.1359.

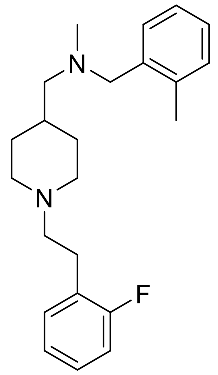

1-(1-(2-Fluorophenethyl)piperidin-4-yl)-*N*-methyl-*N*-(2-methylbenzyl)methanamine (NDM-637): Compound **9** was Boc-deprotected with trifluoroacetic acid in dichloromethane following the general synthetic procedure for Boc-deprotection and subsequently reacted with 1-(bromomethyl)-2-methylbenzene and potassium carbonate in acetonitrile following the general

procedure for amine alkylation. The product was transformed into a salt using the general procedure for making HCl salts. The product presented as a white solid (53%). ^{1}H NMR (400 MHz, methanol-d_4) δ 7.31 – 7.20 (m, 3H), 7.18 – 7.10 (m, 4H), 7.10 – 7.02 (m, 1H), 3.46 (s, 2H), 3.09 – 3.01 (m, 2H), 2.92 – 2.85 (m, 2H), 2.67 – 2.60 (m, 2H), 2.38 (s, 3H), 2.25 (d, *J* = 7.2 Hz, 2H), 2.23 – 2.14 (m, 5H), 1.85 (d, *J* = 12.0 Hz, 2H), 1.69 – 1.56 (m, 1H), 1.25 – 1.13 (m, 2H); ^{13}C NMR (100.6 MHz, methanol-d_4) δ 161.1 (d, *J* = 244.5 Hz), 138.6, 131.7, 131.2, 130.9 (d, *J* = 4.1 Hz), 130.1, 129.2 (d, *J* = 8.2 Hz), 127.8, 126.6, 124.5 (d, *J* = 3.6 Hz), 123.0 (d, *J* = 15.5 Hz), 115.1 (d, *J* = 21.8 Hz), 60.4, 57.3, 56.2, 51.7, 40.1, 29.3, 27.3, 23.5, 18.5; IR ν_{max} (cm^{-1}) 3418, 2935, 2500, 1493, 1452, 1232, 743, 697; UV (λ_{max} nm) 250; LC Trace: 100%; HRMS (ESI) *m/z* calculated for $C_{23}H_{32}FN_2$ $[M+H]^+$: 355.2544, found 355.2535.

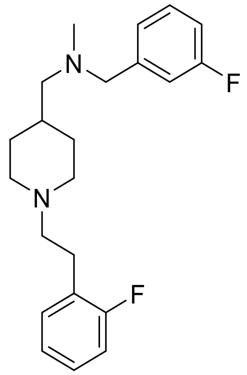

***N*-(3-Fluorobenzyl)-1-(1-(2-fluorophenethyl)piperidin-4-yl)-*N*-methylmethanamine (NDM-556):** Compound **9** was Boc-deprotected with trifluoroacetic acid in dichloromethane following the general synthetic procedure for Boc-deprotection and subsequently reacted with 1-(bromomethyl)-3-fluorobenzene and potassium carbonate in acetonitrile following the general procedure for amine alkylation. The product was transformed into a salt using the general procedure for making HCl salts. The product presented as a white solid (55%). ^{1}H NMR (400 MHz, methanol-d_4) δ 7.35 – 7.19 (m, 3H), 7.16 – 7.01 (m, 4H), 7.00 – 6.93 (m, 1H), 3.49 (s, 2H), 3.05 (d, *J* = 11.6 Hz, 2H), 2.92 – 2.84 (m, 2H), 2.65 – 2.57 9 (m, 2H), 2.23 (d, *J* = 7.1 Hz, 2H), 2.20 (s, 3H), 2.18 – 2.10 (m, 2H), 1.86 (d, *J* = 12.8 Hz, 2H), 1.69 – 1.56 (m, 1H), 1.31 – 1.14 (m, 2H); ^{13}C NMR (100.6 MHz, methanol-d_4) δ 162.9 (d, *J* = 244.1 Hz), 161.1 (d, *J* = 243.5 Hz), 142.0 (d, *J* = 6.9 Hz), 130.7 (d, *J* = 4.6 Hz), 129.5 (d, *J* = 8.2 Hz), 128.2 (d, *J* = 8.1 Hz), 125.6 (d, *J* = 15.8 Hz), 124.4 (d, *J* = 2.8 Hz), 124.1 (d, *J* = 3.6 Hz), 115.1 (d, *J* = 21.4 Hz), 114.8 (d, *J* = 22.2 Hz), 113.3 (d, *J* = 21.4 Hz), 62.8, 61.8, 61.7, 58.7, 41.7, 33.0, 29.4, 25.3; ^{19}F NMR (376.4 Hz, methanol-d_4) δ -115.9, -121.0; UV (λ_{max} nm) 252; LC Trace: 100%; HRMS (ESI) *m/z* calculated for $C_{22}H_{29}F_2N_2$ $[M+H]^+$: 359.2293, found 359.2292.

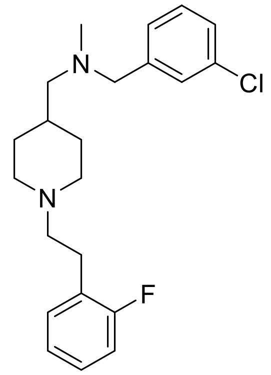

***N*-(3-Chlorobenzyl)-1-(1-(2-fluorophenethyl)piperidin-4-yl)-*N*-methylmethanamine (NDM-49):** Compound **9** was Boc-deprotected with trifluoroacetic acid in dichloromethane following the general synthetic procedure for Boc-deprotection and subsequently reacted with 1-(bromomethyl)-3-chlorobenzene and potassium carbonate in acetonitrile following the general procedure for amine alkylation. The product was transformed into a salt using the general procedure for making HCl salts. The product presented as a white solid (54%). ^{1}H NMR (500 MHz, methanol-d_4) δ 7.68 (s, 1H), 7.60 – 7.46 (m, 3H), 7.38 – 7.31 (m, 2H), 7.22 – 7.08 (m, 2H), 4.52 (d, *J* = 12.9 Hz, 1H), 4.32 (d, *J* = 13.1 Hz, 1H), 3.74 (d, *J* = 12.3 Hz, 2H), 3.35 (dd, *J* = 10.7, 6.4 Hz, 2H), 3.24 – 3.08 (m, 6H), 2.88 (s, 3H), 2.37 – 2.28 (m, 1H), 2.16 (dd, *J* = 39.5, 14.5 Hz, 2H), 1.73 – 1.51 (m, 2H); ^{13}C NMR (126 MHz, methanol-d_4) δ 161.3 (d, *J* = 244.1 Hz), 135.0, 131.3, 131.3, 131.0 (d, *J* = 4.1 Hz), 130.8, 130.4, 129.8, 129.5 (d, *J* = 8.2 Hz), 124.8 (d, *J* = 3.4 Hz), 123.0, 115.4 (d, *J* = 21.8 Hz), 60.2, 59.6, 56.5, 51.9, 39.9, 29.4, 27.4, 23.8; IR ν_{max} (cm^{-1}) 3391, 2928, 2498, 1493, 1453, 1230, 758; UV (λ_{max} nm) 264; LC Trace: 100%; HRMS (ESI) *m/z* calculated for $C_{22}H_{29}ClFN_2$ $[M+H]^+$: 375.1998, found 375.1988.

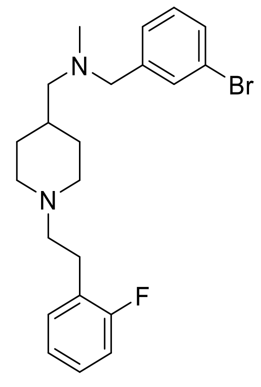

***N*-(3-Bromobenzyl)-1-(1-(2-fluorophenethyl)piperidin-4-yl)-*N*-methylmethanamine (NDM-557):** Compound **9** was Boc-deprotected with trifluoroacetic acid in dichloromethane following the general synthetic procedure for Boc-deprotection and subsequently reacted with 1-(bromomethyl)-3-bromobenzene and potassium carbonate in acetonitrile following the general procedure for amine alkylation. The product was transformed into a salt using the general procedure for making HCl salts. The product presented as a white solid (44%). ^{1}H NMR (400 MHz, methanol-d_4) δ 7.74 (s, 1H), 7.60 (d, *J* = 8.4 Hz, 1H), 7.50 (d, *J* = 7.7 Hz, 1H), 7.35 (t, *J* = 7.9 Hz, 1H), 7.31 – 7.19 (m, 2H), 7.11 – 6.99 (m, 2H), 4.44 – 4.15 (m, 2H), 3.64 (d, *J* = 12.2 Hz, 2H), 3.29 – 3.22 (m, 2H), 3.13 – 2.95 (m, 6H), 2.77 (s, 3H), 2.29 – 2.16 (m, 1H), 2.15 – 1.98 (m, 2H), 1.65 – 1.42 (m, 2H); ^{13}C NMR (100.6 MHz, methanol-d_4) δ 161.1 (d, *J* = 244.4 Hz), 134.1, 133.2, 131.4, 130.9 (d, *J* = 4.2 Hz), 130.8, 130.1, 129.3 (d, *J* = 8.4 Hz), 124.5 (d, *J* = 3.6 Hz), 122.9

(d, J = 15.3 Hz), 122.7, 115.2 (d, J = 21.9 Hz), 60.0, 59.3, 58.4, 56.3, 39.7, 29.2, 27.2, 23.6; ^{19}F NMR (376.4 Hz, methanol-d$_4$) δ -120.4; UV (λ_{max} nm) 247; LC Trace: 100%; HRMS (ESI) *m/z* calculated for $C_{22}H_{29}BrFN_2$ $[M+H]^+$: 419.1493, found 419.1490.

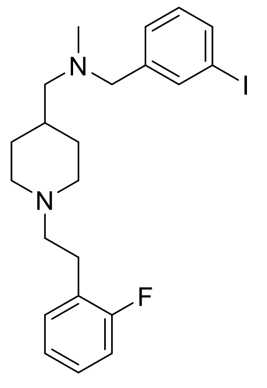

1-(1-(2-Fluorophenethyl)piperidin-4-yl)-*N*-(3-iodobenzyl)-*N*-methylmethanamine (NDM-32): Compound **9** was Boc-deprotected with trifluoroacetic acid in dichloromethane following the general synthetic procedure for Boc-deprotection and subsequently reacted with 1-(bromomethyl)-3-iodobenzene and potassium carbonate in acetonitrile following the general procedure for amine alkylation. The product was transformed into a salt using the general procedure for making HCl salts. The product presented as a white solid (20%). ^{1}H NMR (400 MHz, methanol-d$_4$) δ 8.05 (s, 1H), 7.91 (dd, J = 8.1, 1.6 Hz, 1H), 7.64 (d, J = 7.7 Hz, 1H), 7.46 – 7.26 (m, 3H), 7.26 – 7.08 (m, 2H), 4.51 (d, J = 12.9 Hz, 1H), 4.30 (d, J = 12.9 Hz, 1H), 3.76 (d, J = 12.2 Hz, 2H), 3.41 – 3.34 (m, 2H), 3.21 – 3.15 (m, 6H), 2.89 (s, 3H), 2.35 (s, 1H), 2.24 – 2.13 (m, 2H), 1.78 – 1.55 (m, 2H); ^{13}C NMR (100.6 MHz, methanol-d$_4$) δ 162.2 (d, J = 236.0 Hz), 140.0, 139.2, 131.3, 130.9, 130.7, 130.6, 129.2, 124.5, 115.3, 115.1, 94.2, 60.0, 59.2, 56.3, 51.7, 39.7, 29.2, 27.2, 23.6; IR ν_{max} (cm^{-1}) 3390, 2924, 2500, 1493, 1453, 1230, 759; UV (λ_{max} nm) 204; LC Trace: 100%; HRMS (ESI) *m/z* calculated for $C_{22}H_{29}FIN_2$ $[M+H]^+$: 467.1354, found 467.1348.

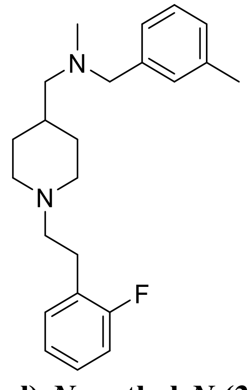

1-(1-(2-Fluorophenethyl)piperidin-4-yl)-*N*-methyl-*N*-(3-methylbenzyl)methanamine (NDM-561): Compound **9** was Boc-deprotected with trifluoroacetic acid in dichloromethane following the general synthetic procedure for Boc-deprotection and subsequently reacted with 1-(bromomethyl)-3-methylbenzene and potassium carbonate in acetonitrile following the general procedure for amine alkylation. The product was transformed into a salt using the general procedure for making HCl salts. The product presented as a white solid (30%). ^{1}H NMR (400 MHz, methanol-d$_4$) δ 7.35 – 6.92 (m, 8H), 3.46 (s, 2H), 3.05 (d, J = 11.4 Hz, 2H), 2.94 – 2.85 (m, 2H), 2.65 - 2.56 (m, 2H), 2.34 (s, 3H), 2.25 (d, J = 7.06 Hz, 2H), 2.21 (s, 3H), 2.19 – 2.11 (m, 2H),

1.86 (d, J = 13.6 Hz, 2H), 1.71 – 1.59 (m, 1H), 1.36 – 1.16 (m, 2H);^{13}C NMR (100.6 MHz, methanol-d$_4$) δ 163.1 (d, J = 221.3 Hz), 142.0, 137.2, 130.7 (d, J = 4.7 Hz), 129.4 (d, J = 8.3 Hz), 128.0, 127.9, 124.7, 124.4, 123.9 (d, J = 3.6 Hz), 114.9 (d, J = 14.2 Hz), 113.3 (d, J = 21.7 Hz), 63.0, 61.8, 57.9, 53.1, 41.7, 33.4, 30.0, 25.7, 24.1; fluorine NMR; UV (λ_{max} nm) 261; LC Trace: 100%; HRMS (ESI) *m/z* calculated for $C_{23}H_{32}FN_2$ [M+H]$^+$: 355.2544, found 355.2542.

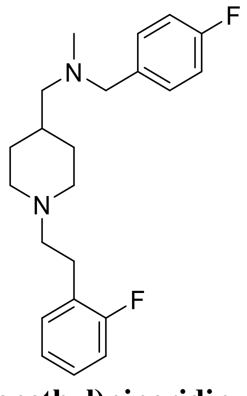

***N*-(4-Fluorobenzyl)-1-(1-(2-fluorophenethyl)piperidin-4-yl)-*N*-methylmethanamine (NDM-48):** Compound **9** was Boc-deprotected with trifluoroacetic acid in dichloromethane following the general synthetic procedure for Boc-deprotection and subsequently reacted with 1-(bromomethyl)-4-fluorobenzene and potassium carbonate in acetonitrile following the general procedure for amine alkylation. The product was transformed into a salt using the general procedure for making HCl salts. The product presented as a white solid (54%). ^{1}H NMR (400 MHz, methanol-d$_4$) δ 7.69 – 7.62 (m, 2H), 7.42 – 7.32 (m, 2H), 7.28 (t, J = 8.5 Hz, 2H), 7.23 – 7.11 (m, 2H), 4.55 – 4.30 (m, 2H), 3.76 (d, J = 12.4 Hz, 2H), 3.40 – 3.34 (m, 2H), 3.23 – 3.08 (m, 6H), 2.89 (s, 3H), 2.40 – 2.23 (m, 1H), 2.25 – 2.10 (m, 2H), 1.76 – 1.51 (m, 2H); ^{13}C NMR (100.6 MHz, methanol-d$_4$) δ 161.1 (d, J = 244.6 Hz), 144.6, 133.7, 133.6, 130.9, 129.3, 129.3, 125.0, 124.6, 116.1, 115.8, 115.2 (d, J = 21.1 Hz), 59.7, 59.3, 56.3, 51.7, 39.5, 29.2, 27.2, 23.6; IR ν_{max} (cm^{-1}) 2925, 2502, 1511, 1451, 1228, 753; UV (λ_{max} nm) 260; LC Trace: 100%; HRMS (ESI) *m/z* calculated for $C_{22}H_{29}F_2N_2$ [M+H]$^+$: 359.2293, found 359.2293.

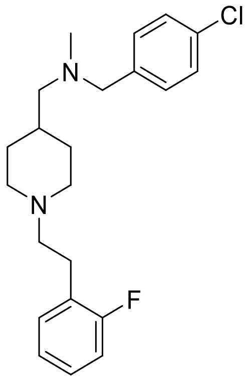

***N*-(4-Chlorobenzyl)-1-(1-(2-fluorophenethyl)piperidin-4-yl)-*N*-methylmethanamine (NDM-47):** Compound **9** was Boc-deprotected with trifluoroacetic acid in dichloromethane following the general synthetic procedure for Boc-deprotection and subsequently reacted with 1-(bromomethyl)-4-chlorobenzene and potassium carbonate in acetonitrile following the general procedure for amine alkylation. The product was transformed into a salt using the general procedure for making HCl salts. The product presented as a white solid (39%). ^{1}H NMR (400 MHz, methanol-d$_4$) δ 7.67

– 7.62 (m, 2H), 7.56 – 7.52 (m, 2H), 7.43 – 7.31 (m, 2H), 7.21 – 7.11 (m, 2H), 4.53 (d, *J* = 13.0 Hz, 1H), 4.37 (d, *J* = 12.7 Hz, 1H), 3.74 (d, *J* = 12.1 Hz, 2H), 3.41 – 3.34 (m, 2H), 3.24 – 3.10 (m, 6H), 2.90 (s, 3H), 2.37 (t, *J* = 4.5 Hz, 1H), 2.24 (d, *J* = 14.4 Hz, 1H), 2.19 – 2.11 (m, 1H), 1.79 – 1.55 (m, 2H); ^{13}C NMR (100.6 MHz, methanol-d$_4$) δ 161.2 (d, *J* = 244.4 Hz), 136.1, 133.0 (d, *J* = 10.1 Hz), 131.0, 130.9 (d, *J* = 4.1 Hz), 129.3, 127.7, 124.5 (d, *J* = 3.6 Hz), 123.0 (d, *J* = 15.1 Hz), 115.2 (d, *J* = 21.8 Hz), 59.7, 59.2, 56.2, 51.6, 39.7, 29.2, 27.2, 23.6; IR ν_{max} (cm^{-1}) 3027, 2926, 2499, 1493, 1456, 1232, 750; UV (λ_{max} nm) 268; LC Trace: 100%; HRMS (ESI) *m/z* calculated for $C_{22}H_{29}ClFN_2$ [M+H]$^+$: 375.1998, found 375.1996.

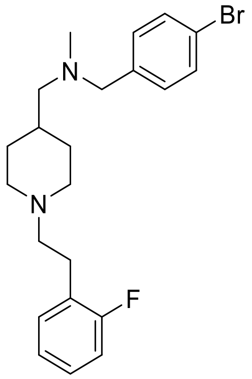

***N*-(4-Bromobenzyl)-1-(1-(2-fluorophenethyl)piperidin-4-yl)-*N*-methylmethanamine (NDM-558):** Compound **9** was Boc-deprotected with trifluoroacetic acid in dichloromethane following the general synthetic procedure for Boc-deprotection and subsequently reacted with 1-(bromomethyl)-4-bromobenzene and potassium carbonate in acetonitrile following the general procedure for amine alkylation. The product was transformed into a salt using the general procedure for making HCl salts. The product presented as a white solid (45%). ^{1}H NMR (400 MHz, methanol-d$_4$) δ 7.46 (d, *J* = 8.3 Hz, 2H), 7.31 – 7.19 (m, 4H), 7.14 – 7.01 (m, 2H), 3.45 (s, 2H), 3.03 (d, *J* = 11.6 Hz, 2H), 2.92 – 2.83 (m, 2H), 2.63 - 2.54 (m, 2H), 2.22 (d, *J* = 7.1 Hz, 2H), 2.19 (s, 3H), 2.16 – 2.06 (m, 2H), 1.84 (d, *J* = 12.4 Hz, 2H), 1.68 – 1.56 (m, 1H), 1.28 – 1.14 (m, 2H); ^{13}C NMR (100.6 MHz, methanol-d$_4$) δ 161.1 (d, *J* = 244.3 Hz), 133.2, 132.9, 131.1, 130.8 (d, *J* = 4.4 Hz), 129.2 (d, *J* = 8.00 Hz), 124.5 (d, *J* = 3.6 Hz), 125.4, 116.9, 115.2 (d, *J* = 21.8 Hz), 60.0, 58.8, 56.3, 51.7, 39.6, 29.2, 27.2, 23.6; ^{19}F NMR (376.4 Hz, methanol-d$_4$) δ -120.4; UV (λ_{max} nm) 261; LC Trace: 100%; HRMS (ESI) *m/z* calculated for $C_{22}H_{29}BrFN_2$ [M+H]$^+$: 419.1493, found 419.1483.

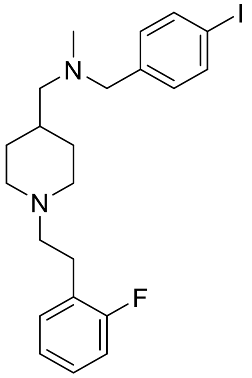

1-(1-(2-Fluorophenethyl)piperidin-4-yl)-*N*-(4-iodobenzyl)-*N*-methylmethanamine (NDM-563): Compound **9** was Boc-deprotected with trifluoroacetic acid in dichloromethane following

the general synthetic procedure for Boc-deprotection and subsequently reacted with 1-(bromomethyl)-4-iodobenzene and potassium carbonate in acetonitrile following the general procedure for amine alkylation. The product was transformed into a salt using the general procedure for making HCl salts. The product presented as a white solid (35%). ^{1}H NMR (400 MHz, methanol-d_4) δ 7.66 (d, *J* = 8.0 Hz, 2H), 7.31 - 7.20 (m, 2H), 7.19 – 6.99 (m, 4H), 3.44 (s, 2H), 3.06 (d, *J* = 11.6 Hz, 2H), 2.96 – 2.84 (m, 2H), 2.66 – 2.58 (m, 2H), 2.22 (d, *J* = 7.14, 2H), 2.19 (s, 3H), 2.18 – 2.11 (m, 2H), 1.86 (d, *J* = 12.9 Hz, 2H), 1.69 – 1.56 (m, 1H), 1.28 – 1.15 (m, 2H); ^{13}C NMR (100.6 MHz, methanol-d_4) δ 161.1 (d, *J* = 240.1 Hz), 134.8, 131.1, 130.8 (d, *J* = 4.8 Hz), 130.6, 129.6, 129.3 (d, *J* = 9.3 Hz), 127.1 (d, *J* = 19.8 Hz), 124.5 (d, *J* = 3.6 Hz), 115.2 (d, *J* = 22.0 Hz), 60.0, 59.4, 56.2, 51.7, 39.7, 29.2, 27.2, 23.6; ^{19}F NMR (376.4 Hz, methanol-d_4) δ -115.8; UV (λ_{max} nm) 262; LC Trace: 100%; HRMS (ESI) *m/z* calculated for $C_{22}H_{29}FIN_2$ $[M+H]^+$: 467.1354, found 467.1346.

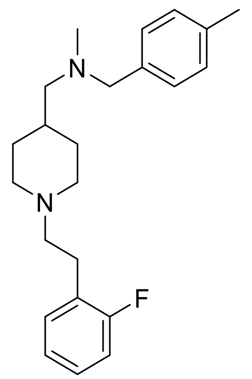

1-(1-(2-Fluorophenethyl)piperidin-4-yl)-*N*-methyl-*N*-(4-methylbenzyl)methanamine (NDM-562): Compound **9** was Boc-deprotected with trifluoroacetic acid in dichloromethane following the general synthetic procedure for Boc-deprotection and subsequently reacted with 1-(bromomethyl)-4-iodobenzene and potassium carbonate in acetonitrile following the general procedure for amine alkylation. The product was transformed into a salt using the general procedure for making HCl salts. The product presented as a white solid (34%). ^{1}H NMR (400 MHz, methanol-d_4) δ 7.28 – 7.15 (m, 4H), 7.13 – 7.07 (m, 3H), 7.07 – 6.99 (m, 1H), 3.46 – 3.39 (m, 2H), 3.07 – 2.95 (m, 2H), 2.90 – 2.81 (m, 2H), 2.62 – 2.52 (m, 2H), 2.30 (s, 3H), 2.23 – 2.15 (m, 5H), 2.13 – 2.06 (m, 2H), 1.82 (d, *J* = 13.1 Hz, 2H), 1.67 – 1.53 (m, 1H), 1.26 – 1.12 (m, 2H); ^{13}C NMR (100.6 MHz, methanol-d_4) δ 163.0 (d, *J* = 241.9 Hz), 142.0, 137.2, 130.7 (d, *J* = 4.7 Hz), 129.5, 127.9 (d, *J* = 8.4 Hz), 124.4, 124.0 (d, *J* = 3.6 Hz), 115.3 (d, *J* = 18.9 Hz), 114.7 (d, *J* = 22.2 Hz), 63.1, 61.8, 58.0, 53.0, 41.7, 33.4, 29.9, 25.7, 24.1; ^{19}F NMR (376.4 Hz, methanol-d_4) δ -116.0; UV (λ_{max} nm) 253; LC Trace: 100%; HRMS (ESI) *m/z* calculated for $C_{23}H_{32}FN_2$ $[M+H]^+$: 355.2544, found 355.2546.

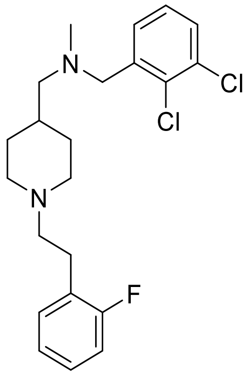

***N*-(2,3-Dichlorobenzyl)-1-(1-(2-fluorophenethyl)piperidin-4-yl)-*N*-methylmethanamine (NDM-45):** Compound **9** was Boc-deprotected with trifluoroacetic acid in dichloromethane following the general synthetic procedure for Boc-deprotection and subsequently reacted with 1-(bromomethyl)-2,3-dichlorobenzene and potassium carbonate in acetonitrile following the general procedure for amine alkylation. The product was transformed into a salt using the general procedure for making HCl salts. The product presented as a white solid (46%). ^{1}H NMR (400 MHz, methanol-d$_4$) δ 7.49 – 7.37 (m, 2H), 7.32 – 7.19 (m, 3H), 7.15 – 7.01 (m, 2H), 3.60 (s, 2H), 3.16 (d, *J* = 11.6 Hz, 2H), 2.92 (dd, *J* = 10.6, 6.0 Hz, 2H), 2.81 – 2.70 (m, 2H), 2.41 – 2.27 (m, 4H), 2.22 (d, *J* = 2.4 Hz, 3H), 1.91 (d, *J* = 13.6 Hz, 2H), 1.69 (ddd, *J* = 11.9, 8.0, 4.2 Hz, 1H), 1.33 – 1.18 (m, 2H); ^{13}C NMR (100.6 MHz, methanol-d$_4$) δ 161.1 (d, J = 243.7 Hz), 139.3, 132.6, 132.0, 130.8 (d, *J* = 4.7 Hz), 129.0, 128.8, 128.2 (d, *J* = 8.2 Hz), 127.0, 125.6 (d, *J* = 16.1 Hz), 124.1 (d, *J* = 3.5 Hz), 114.9 (d, *J* = 22.2 Hz), 63.2, 59.9, 58.1, 52.9, 41.7, 33.1, 29.4, 25.3; IR ν_{max} (cm^{-1}) 3403, 2925, 2516, 1449, 1427, 1230, 754; UV (λ_{max} nm) 258; LC Trace: 100%; HRMS (ESI) *m/z* calculated for $C_{22}H_{28}Cl_2FN_2$ [M+H]$^+$: 409.1608, found 409.1610.

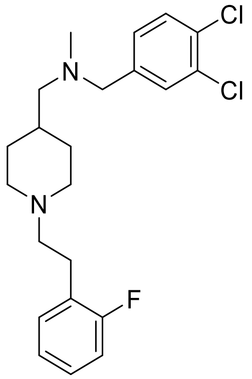

***N*-(3,4-Dichlorobenzyl)-1-(1-(2-fluorophenethyl)piperidin-4-yl)-*N*-methylmethanamine (NDM-46):** Compound **9** was Boc-deprotected with trifluoroacetic acid in dichloromethane following the general synthetic procedure for Boc-deprotection and subsequently reacted with 1-(bromomethyl)-3,4-dichlorobenzene and potassium carbonate in acetonitrile following the general procedure for amine alkylation. The product was transformed into a salt using the general procedure for making HCl salts. The product presented as a white solid (42%). ^{1}H NMR (400 MHz, methanol-d$_4$) δ 7.90 (d, *J* = 2.1 Hz, 1H), 7.70 (d, *J* = 8.3 Hz, 1H), 7.61 (dd, *J* = 8.3, 2.1 Hz, 1H), 7.46 – 7.29 (m, 2H), 7.25 – 7.10 (m, 2H), 4.55 (d, *J* = 12.9 Hz, 1H), 4.36 (d, *J* = 12.9 Hz, 1H), 3.75 (d, *J* = 12.4 Hz, 2H), 3.40 – 3.34 (m, 3H), 3.25 – 3.15 (m, 5H), 2.90 (s, 3H), 2.41 – 2.31 (m, 1H), 2.26 (d, *J* = 14.5 Hz, 1H), 2.15 (d, *J* = 14.6 Hz, 1H), 1.78 – 1.59 (m, 2H); ^{13}C NMR (100.6 MHz, methanol-d$_4$) δ 161.1 (d, *J* = 244.6 Hz), 147.1, 134.2, 133.3, 132.8, 131.1 (d, *J* = 8.7 Hz),

130.9 (d, J = 4.2 Hz), 129.6, 129.2 (d, J = 8.2 Hz), 124.5, 123.0 (d, J = 15.4 Hz), 115.2 (d, J = 21.7 Hz), 60.0, 58.7, 56.2, 51.7, 39.6, 29.2, 27.2, 23.6; IR ν_{max} (cm^{-1}) 2929, 2513, 1493, 1453, 1230, 755; UV (λ_{max} nm) 258; LC Trace: 100%; HRMS (ESI) *m/z* calculated for $C_{22}H_{28}Cl_2FN_2$ [M+H]$^+$: 409.1608, found 409.1617.

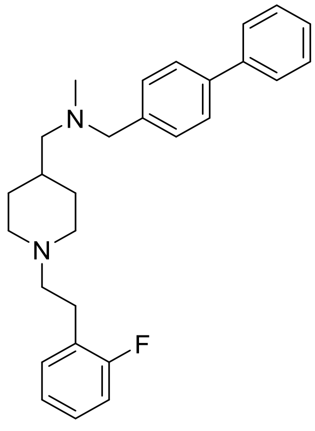

1-([1,1'-Biphenyl]-4-yl)-*N*-((1-(2-fluorophenethyl)piperidin-4-yl)methyl)-*N*-methylmethanamine (NDM-564): Compound **9** was Boc-deprotected with trifluoroacetic acid in dichloromethane following the general synthetic procedure for Boc-deprotection and subsequently reacted with 4-(bromomethyl)-1,1'-biphenyl and potassium carbonate in acetonitrile following the general procedure for amine alkylation. The product was transformed into a salt using the general procedure for making HCl salts. The product presented as a white solid (28%). ^{1}H NMR (400 MHz, methanol-d$_4$) δ 7.65 – 7.56 (m, 4H), 7.47 – 7.39 (m, 4H), 7.36 – 7.20 (m, 3H), 7.14 – 7.02 (m, 2H), 3.56 (s, 2H), 3.17 – 3.02 (m, 2H), 2.94 – 2.85 (m, 2H), 2.72 – 2.62 (m, 2H), 2.33 – 2.17 (m, 6H), 1.90 (d, J = 12.9 Hz, 2H), 1.78 – 1.61 (m, 1H), 1.33 – 1.19 (m, 3H); ^{13}C NMR (100.6 MHz, methanol-d$_4$) δ 161.9, 161.6, 161.1 (d, J = 244.3 Hz), 141.6, 140.1, 132.6, 130.8 (d, J = 4.2 Hz), 129.1 (d, J = 8.0 Hz), 128.6, 126.6, 124.4 (d, J = 3.6 Hz), 123.4 (d, J = 15.6 Hz), 118.3, 115.1 (d, J = 21.9 Hz), 60.3, 58.3, 54.3, 53.5, 39.8, 34.9, 28.8, 25.6; IR ν_{max} (cm^{-1}) 3030, 2945, 2793, 1673, 1493, 1196, 1128, 758; UV (λ_{max} nm) 282; LC Trace: 100%; HRMS (ESI) *m/z* calculated for $C_{28}H_{34}FN_2$ [M+H]$^+$: 417.2701 found 417.2705.

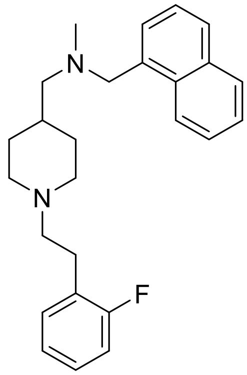

1-(1-(2-Fluorophenethyl)piperidin-4-yl)-*N*-methyl-*N*-(naphthalen-1-ylmethyl)methanamine (NDM-602): Compound **9** was Boc-deprotected with trifluoroacetic acid in dichloromethane following the general synthetic procedure for Boc-deprotection and subsequently reacted with 1-(bromomethyl)naphthalene and potassium carbonate in acetonitrile following the general procedure for amine alkylation. The product was transformed into a salt using the general

procedure for making HCl salts. The product presented as a white solid (51%). ^{1}H NMR (400 MHz, methanol-d$_4$) δ 8.38 – 8.30 (m, 1H), 7.94 – 7.83 (m, 1H) 7.82 – 7.77 (m, 1H), 7.58 – 7.38 (m, 4H), 7.29 – 7.19 (m, 2H), 7.13 – 7.01 (m, 2H), 3.91 (s, 2H), 2.96 (d, J = 11.6 Hz, 2H), 2.89 – 2.79 (m, 2H), 2.62 – 2.51 (m, 2H), 2.32 (d, J = 7.2 Hz, 2H), 2.24 (s, 3H), 2.10 (t, J = 11.7 Hz, 2H), 1.81 (d, J = 12.7 Hz, 2H), 1.69 – 1.56 (m, 1H), 1.19 – 1.04 (m, 2H); ^{13}C NMR (100.6 MHz, methanol-d$_4$) δ 161.1 (d, J = 243.6 Hz), 134.9, 134.0, 132.5, 130.8 (d, J = 4.8 Hz), 128.0, 127.9 (d, J = 8.1 Hz), 127.7, 127.2, 126.5, 126.3, 125.2 (d, J = 11.2 Hz), 124.8, 124.7, 124.0 (d, J = 3.5 Hz), 114.8 (d, J = 22.2 Hz), 63.4, 61.3, 58.7, 52.9, 41.6, 33.4, 29.9, 25.8; IR ν_{max} (cm^{-1}) 3044, 2919, 2790, 1491, 1228, 1124, 753; UV (λ_{max} nm) 274; LC Trace: 100%; HRMS (ESI) *m/z* calculated for $C_{26}H_{32}FN_2$ [M+H]$^+$: 391.2544, found 391.2561.

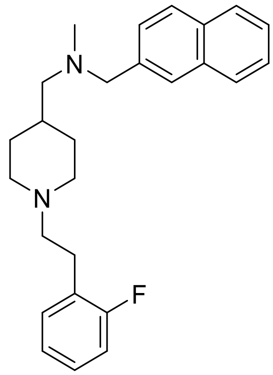

1-(1-(2-Fluorophenethyl)piperidin-4-yl)-*N*-methyl-*N*-(naphthalen-2-ylmethyl)methanamine (NDM-600): Compound **9** was Boc-deprotected with trifluoroacetic acid in dichloromethane following the general synthetic procedure for Boc-deprotection and subsequently reacted with 2-(bromomethyl)naphthalene and potassium carbonate in acetonitrile following the general procedure for amine alkylation. The product was transformed into a salt using the general procedure for making HCl salts. The product presented as a white solid (58%). ^{1}H NMR (400 MHz, methanol-d$_4$) δ 7.85 – 7.80 (m, 3H), 7.75 (s, 1H), 7.53 – 7.49 (m, 1H), 7.48 – 7.42 (m, 2H), 7.28 – 7.19 (m, 2H), 7.09 (t, J = 7.3 Hz, 1H), 7.07 – 7.01 (m, 1H), 3.66 (s, 2H), 3.05 (d, J = 11.6 Hz, 2H), 2.89 – 2.83 (m, 2H), 2.66 – 2.56 (m, 2H), 2.29 (d, J = 7.1 Hz, 2H), 2.26 (s, 3H), 2.20 – 2.11 (m, 2H), 1.89 (d, J = 13.6 Hz, 2H), 1.72 – 1.62 (m, 1H), 1.26 – 1.18 (m, 2H); ^{13}C NMR (100.6 MHz, methanol-d$_4$) δ 161.1 (d, J = 243.5 Hz), 136.4, 133.4, 132.9, 130.7 (d, J = 4.8 Hz), 128.0 (d, J = 8.1 Hz), 127.4 (d, J = 7.0 Hz), 127.3 (d, J = 7.9 Hz), 127.1, 126.3, 126.1, 125.7, 125.3, 124.1, 124.0, 114.8 (d, J = 22.2 Hz), 63.0, 62.5, 58.6, 53.0, 42.0, 33.3, 29.8, 25.7; IR ν_{max} (cm^{-1}) 3055, 2942, 2838, 1491, 1122, 749; UV (λ_{max} nm) 264; LC Trace: 100%; HRMS (ESI) *m/z* calculated for $C_{26}H_{32}FN_2$ [M+H]$^+$: 391.2544, found 391.2538.

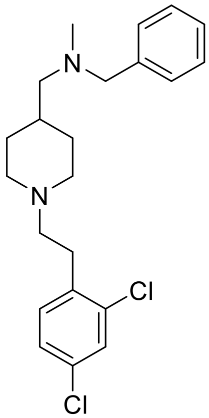

***N*-Benzyl-1-(1-(2,4-dichlorophenethyl)piperidin-4-yl)-*N*-methylmethanamine (NDM-53):** Compound **11a** was Boc-deprotected with trifluoroacetic acid in dichloromethane following the general synthetic procedure for Boc-deprotection and subsequently reacted with (bromomethyl)benzene and potassium carbonate in acetonitrile following the general procedure for amine alkylation. The product was transformed into a salt using the general procedure for making HCl salts. The product presented as a white solid (43%). ^{1}H NMR (400 MHz, methanol-d_4) δ 7.43 (d, *J* = 2.1 Hz, 1H), 7.35 – 7.20 (m, 7H), 3.49 (s, 2H), 3.03 (d, *J* = 11.5 Hz, 2H), 2.99 – 2.92 (m, 2H), 2.61 – 2.54 (m, 2H), 2.23 (d, *J* = 7.1 Hz, 2H), 2.20 (s, 3H), 2.17 – 2.12 (m, 2H), 1.85 (d, *J* = 13.4 Hz, 2H), 1.68 – 1.57 (m, 1H), 1.26 – 1.15 (m, 2H); ^{13}C NMR (100.6 MHz, methanol-d_4) δ 138.6, 137.0, 136.3, 134.3, 132.5, 132.1, 131.8, 128.9, 128.7, 127.8, 127.1, 126.8, 63.0, 62.3, 58.0, 53.1, 41.9, 33.4, 30.0, 29.6; IR ν_{max} (cm^{-1}) 3027, 2924, 2785, 1471, 1100, 697; UV (λ_{max} nm) 256; LC Trace: 100%; HRMS (ESI) *m/z* calculated for $C_{22}H_{29}Cl_2N_2$ $[M+H]^+$: 391.1702, found 391.1705.

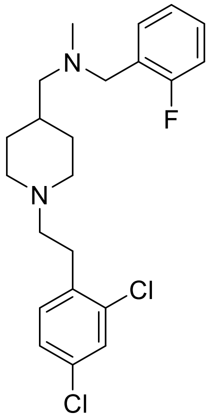

1-(1-(2,4-Dichlorophenethyl)piperidin-4-yl)-*N*-(2-fluorobenzyl)-*N*-methylmethanamine (NDM-590): Compound **11a** was Boc-deprotected with trifluoroacetic acid in dichloromethane following the general synthetic procedure for Boc-deprotection and subsequently reacted with 1-(bromomethyl)-2-fluorobenzene and potassium carbonate in acetonitrile following the general procedure for amine alkylation. The product was transformed into a salt using the general procedure for making HCl salts. The product presented as a white solid (37%). ^{1}H NMR (400 MHz, methanol-d_4) δ 7.36 (s, 1H), 7.34 – 7.28 (m, 1H), 7.28 – 7.14 (m, 3H), 7.08 – 7.01 (m, 1H), 7.00 – 6.92 (m, 1H), 3.49 (s, 2H), 3.15 (d, *J* = 11.2, 2H), 2.98 – 2.89 (m, 2H), 2.75 – 2.68 (m, 2H), 2.44 – 2.32 (m, 2H), 2.21 (d, *J* = 7.1 Hz, 2H), 2.12 (s, 3H), 1.84 (d, *J* = 13.7 Hz, 2H), 1.71 – 1.57 (m, 1H), 2.22 – 2.17 (m, 2H) ; ^{13}C NMR (100.6 MHz, methanol-d_4) δ 161.7 (d, *J* = 248.2 Hz),

134.4, 133.8, 133.5 (d, J = 2.2 Hz), 132.9 (d, J = 8.7 Hz), 132.6, 132.0, 129.1, 127.6, 125.0 (d, J = 3.9 Hz), 116.1 (d, J = 14.4 Hz), 115.8 (d, J = 21.6 Hz), 62.1, 60.1, 55.6, 52.1, 39.9, 29.2, 27.2, 27.0; ^{19}F NMR (376.4 Hz, methanol-d$_4$) δ -116.3; UV (λ_{max} nm) 255; LC Trace: 100%; HRMS (ESI) *m/z* calculated for $C_{22}H_{28}Cl_2FN_2$ [M+H]$^+$: 409.1608, found 409.1605.

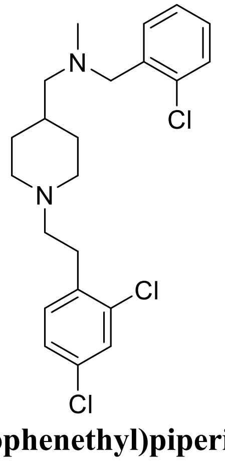

***N*-(2-Chlorobenzyl)-1-(1-(2,4-dichlorophenethyl)piperidin-4-yl)-*N*-methylmethanamine (NDM-59):** Compound **11a** was Boc-deprotected with trifluoroacetic acid in dichloromethane following the general synthetic procedure for Boc-deprotection and subsequently reacted with 1-(bromomethyl)-2-chlorobenzene and potassium carbonate in acetonitrile following the general procedure for amine alkylation. The product was transformed into a salt using the general procedure for making HCl salts. The product presented as a white solid (31%). ^{1}H NMR (400 MHz, methanol-d$_4$) δ 7.49 (dd, J = 7.5, 2.0 Hz, 1H), 7.42 (d, J = 2.1 Hz, 1H), 7.36 (dd, J = 7.6, 1.7 Hz, 1H), 7.34 – 7.18 (m, 4H), 3.59 (s, 2H), 3.02 (d, J = 11.5 Hz, 2H), 2.99 – 2.90 (m, 2H), 2.63 – 2.51 (m, 2H), 2.29 (d, J = 7.2 Hz, 2H), 2.22 (s, 3H), 2.14 (td, J = 11.8, 2.5 Hz, 2H), 1.95 – 1.79 (m, 2H), 1.68 – 1.55 (m, 1H), 1.27 – 1.14 (m, 2H); ^{13}C NMR (100.6 MHz, methanol-d$_4$) δ 136.5, 136.2, 134.3, 134.1, 132.5, 131.8, 130.9, 129.1, 128.8, 128.1, 127.1, 126.4, 63.5, 63.4, 59.0, 58.0, 41.8, 33.5, 30.0, 29.4; IR ν_{max} (cm^{-1}) 2923, 2791, 1471, 1052, 751; UV (λ_{max} nm) 254; LC Trace: 100%; HRMS (ESI) *m/z* calculated for $C_{22}H_{28}Cl_3N_2$ [M+H]$^+$: 425.1313, found 425.1307.

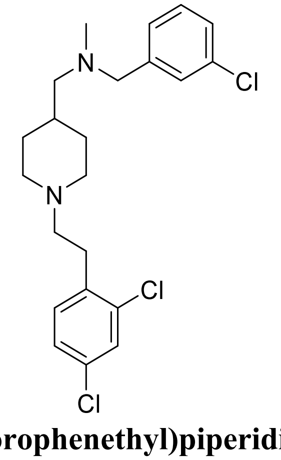

***N*-(3-Chlorobenzyl)-1-(1-(2,4-dichlorophenethyl)piperidin-4-yl)-*N*-methylmethanamine (NDM-58):** Compound **11a** was Boc-deprotected with trifluoroacetic acid in dichloromethane following the general synthetic procedure for Boc-deprotection and subsequently reacted with 1-(bromomethyl)-3-chlorobenzene and potassium carbonate in acetonitrile following the general procedure for amine alkylation. The product was transformed into a salt using the general

procedure for making HCl salts. The product presented as a white solid (31%). ^{1}H NMR (400 MHz, methanol-d$_4$) δ 7.43 (d, *J* = 2.1 Hz, 1H), 7.36 (s, 1H), 7.34 – 7.19 (m, 5H), 3.48 (s, 2H), 3.05 (d, *J* = 11.4 Hz, 2H), 3.01 – 2.89 (m, 2H), 2.64 – 2.54 (m, 2H), 2.22 (d, *J* = 7.2 Hz, 2H), 2.19 (s, 3H), 2.19 – 2.13 (m, 2H), 1.91 – 1.81 (m, 2H), 1.62 (ddt, *J* = 11.2, 7.6, 3.8 Hz, 1H), 1.27 – 1.15 (m, 2H); ^{13}C NMR (100.6 MHz, methanol-d$_4$) δ 141.6, 136.0, 134.3, 133.8, 132.6, 131.8, 129.4, 128.8, 128.6, 127.2, 127.1, 126.8, 63.0, 61.7, 57.8, 53.0, 41.7, 33.3, 29.8, 29.4; IR ν_{max} (cm^{-1}) 3062, 2925, 2791, 1472, 1100, 778; UV (λ_{max} nm) 254; LC Trace: 100%; HRMS (ESI) *m/z* calculated for $C_{22}H_{28}Cl_3N_2$ [M+H]$^+$: 425.1313, found 425.1324.

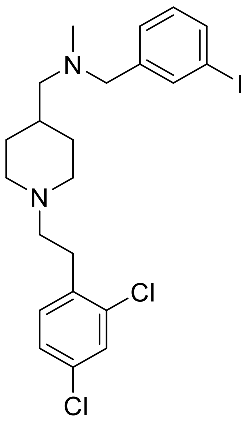

1-(1-(2,4-Dichlorophenethyl)piperidin-4-yl)-*N*-(3-iodobenzyl)-*N*-methylmethanamine (NDM-61): Compound **11a** was Boc-deprotected with trifluoroacetic acid in dichloromethane following the general synthetic procedure for Boc-deprotection and subsequently reacted with 1-(bromomethyl)-3-iodobenzene and potassium carbonate in acetonitrile following the general procedure for amine alkylation. The product was transformed into a salt using the general procedure for making HCl salts. The product presented as a white solid (32%). ^{1}H NMR (400 MHz, methanol-d$_4$) δ 7.74 (s, 1H), 7.61 (d, *J* = 8.0 Hz, 1H), 7.45 (d, *J* = 2.1 Hz, 1H), 7.38 – 7.25 (m, 3H), 7.10 (t, *J* = 7.7 Hz, 1H), 3.46 (s, 2H), 3.05 (d, *J* = 11.5 Hz, 2H), 3.02 – 2.91 (m, 2H), 2.66 – 2.55 (m, 2H), 2.23 (d, *J* = 7.2 Hz, 2H), 2.21 (s, 3H), 2.20 – 2.13 (m, 2H), 1.86 (d, *J* = 13.4 Hz, 2H), 1.64 (ddd, *J* = 11.2, 7.3, 3.8 Hz, 1H), 1.29 – 1.17 (m, 2H); ^{13}C NMR (100.6 MHz, methanol-d$_4$) δ 141.8, 137.7, 136.2, 135.8, 134.3, 132.5, 131.8, 129.8, 128.8, 128.1, 127.1, 93.5, 62.9, 61.6, 58.0, 53.1, 41.8, 33.3, 29.9, 29.6; IR ν_{max} (cm^{-1}) 3055, 2922, 2789, 1470, 1100, 772; UV (λ_{max} nm) 254; LC Trace: 100%; HRMS (ESI) *m/z* calculated for $C_{22}H_{28}Cl_2IN_2$ [M+H]$^+$: 517.0669, found 517.0676.

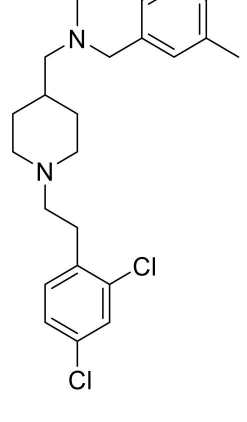

1-(1-(2,4-Dichlorophenethyl)piperidin-4-yl)-*N*-methyl-*N*-(3-methylbenzyl)methanamine (NDM-589): Compound **11a** was Boc-deprotected with trifluoroacetic acid in dichloromethane following the general synthetic procedure for Boc-deprotection and subsequently reacted with 1-(bromomethyl)-3-methylbenzene and potassium carbonate in acetonitrile following the general procedure for amine alkylation. The product was transformed into a salt using the general procedure for making HCl salts. The product presented as a white solid (30%). ^{1}H NMR (400 MHz, methanol-d$_4$) δ 7.33 (d, *J* = 1.9 Hz, 1H), 7.23 – 7.14 (m, 2H), 7.12 -7.05 (m, 1H), 7.03 (s, 1H), 7.01 – 6.93 (m, 2H), 3.38 (s, 2H), 2.98 (d, *J* = 11.5 Hz, 2H), 2.89 – 2.83 (m, 2H), 2.58 – 2.49 (m, 2H), 2.22 (s, 3H), 2.17 – 2.10 (m, 5H), 1.76 (d, *J* = 11.7 Hz, 2H), 1.62 – 1.50 (m, 1H), 1.33 – 1.16 (m, 2H), 1.15 – 1.06 (m, 2H); ^{13}C NMR (100.6 MHz, methanol-d$_4$) δ 147.1, 141.5, 139.2, 134.5, 133.8, 132.7, 132.1, 131.7, 130.7, 129.1, 128.2, 127.4, 60.3, 59.8, 55.6, 51.9, 39.9, 29.2, 27.3, 19.9; IR ν_{max} (cm^{-1}) 2925, 2795, 1476, 1113, 786; UV (λ_{max} nm) 252; LC Trace: 100%; HRMS (ESI) *m/z* calculated for $C_{23}H_{31}Cl_2N_2$ $[M+H]^+$: 405.1859, found 405.1851.

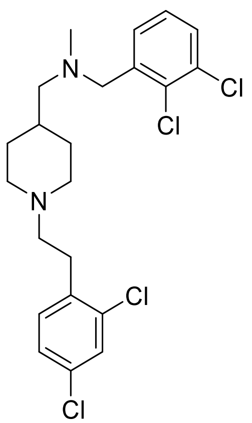

***N*-(2,3-Dichlorobenzyl)-1-(1-(2,4-dichlorophenethyl)piperidin-4-yl)-*N*-methylmethanamine (NDM-55):** Compound **11a** was Boc-deprotected with trifluoroacetic acid in dichloromethane following the general synthetic procedure for Boc-deprotection and subsequently reacted with 1-(bromomethyl)-2,3-dichlorobenzene and potassium carbonate in acetonitrile following the general procedure for amine alkylation. The product was transformed into a salt using the general procedure for making HCl salts. The product presented as a white solid (29%). ^{1}H NMR (400 MHz, methanol-d$_4$) δ 7.48 – 7.42 (m, 3H), 7.32 – 7.24 (m, 3H), 3.61 (s, 2H), 3.02 (d, *J* = 11.4 Hz, 2H), 2.98 – 2.87 (m, 2H), 2.62 – 2.52 (m, 2H), 2.30 (d, *J* = 7.2 Hz, 2H), 2.22 (s, 3H), 2.13 (t, *J* = 11.6 Hz, 2H), 1.86 (d, *J* = 13.4 Hz, 2H), 1.66 – 1.54 (m, 1H), 1.27 – 1.15 (m, 2H); ^{13}C NMR (100.6 MHz, methanol-d$_4$) δ 139.4, 136.0, 134.3, 132.6, 131.9, 131.8, 129.0, 128.8, 128.7, 127.2, 127.0, 63.4, 59.9, 57.8, 53.0, 41.7, 33.4, 29.8, 29.4; IR ν_{max} (cm^{-1}) 2924, 2792, 1471, 1050, 777; UV (λ_{max} nm) 254; LC Trace: 100%; HRMS (ESI) *m/z* calculated for $C_{22}H_{27}Cl_4N_2$ [M+H]+: 459.0923, found 459.0920.

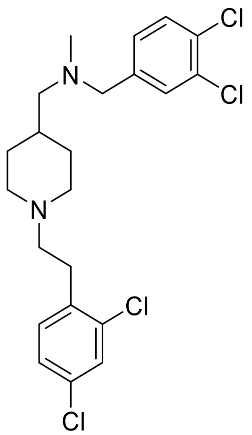

N-(3,4-Dichlorobenzyl)-1-(1-(2,4-dichlorophenethyl)piperidin-4-yl)-N-methylmethanamine (NDM-56): Compound **11a** was Boc-deprotected with trifluoroacetic acid in dichloromethane following the general synthetic procedure for Boc-deprotection and subsequently reacted with 1-(bromomethyl)-3,4-dichlorobenzene and potassium carbonate in acetonitrile following the general procedure for amine alkylation. The product was transformed into a salt using the general procedure for making HCl salts. The product presented as a white solid (20%). ^{1}H NMR (400 MHz, methanol-d_4) δ 7.50 (d, J = 2.0 Hz, 1H), 7.48 – 7.40 (m, 2H), 7.34 – 7.21 (m, 3H), 3.46 (s, 2H), 3.05 (d, J = 11.8 Hz, 2H), 3.00 – 2.88 (m, 2H), 2.64 – 2.52 (m, 2H), 2.22 (d, J = 7.1 Hz, 2H), 2.19 (s, 3H), 2.10 – 2.10 (m, 2H), 1.91 – 1.80 (m, 2H), 1.61 (ddd, J = 11.0, 7.4, 3.5 Hz, 1H), 1.26 – 1.14 (m, 2H); ^{13}C NMR (100.6 MHz, methanol-d_4) δ 140.3, 136.2, 134.3, 132.5, 131.8, 131.7, 130.5, 130.2, 129.9, 128.7, 128.4, 127.1, 63.0, 61.1, 58.0, 53.1, 41.7, 33.4, 29.9, 27.3; IR ν_{max} (cm^{-1}) 2925, 2797, 1471, 1126, 1030, 815; UV (λ_{max} nm) 254; LC Trace: 100%; HRMS (ESI) *m/z* calculated for $C_{22}H_{27}Cl_4N_2$ [M+H]$^+$: 459.0923, found 459.0929.

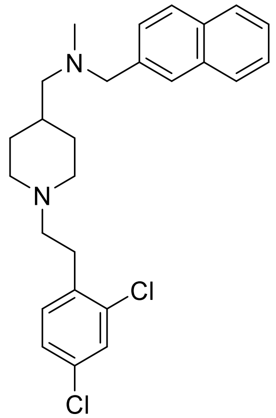

1-(1-(2,4-Dichlorophenethyl)piperidin-4-yl)-N-methyl-N-(naphthalen-2-ylmethyl)methanamine (NDM-598): Compound **11a** was Boc-deprotected with trifluoroacetic acid in dichloromethane following the general synthetic procedure for Boc-deprotection and subsequently reacted with 1-(bromomethyl)-3-methylbenzene and potassium carbonate in acetonitrile following the general procedure for amine alkylation. The product was transformed into a salt using the general procedure for making HCl salts. The product presented as a white solid (64%). ^{1}H NMR (400 MHz, methanol-d_4) δ 7.88 – 7.81 (m, 3H), 7.77 (s, 1H), 7.54 – 7.50 (m, 1H), 7.49 – 7.44 (m, 3H), 7.34 – 7.26 (m, 2H), 3.70 (s, 2H), 3.19 – 3.06 (m, 2H) 3.02 – 2.93 (m, 2H), 2.73 – 2.61 (m, 2H), 2.36 – 2.24 (m, 7H), 1.93 (d, J = 13.4 Hz, 2H), 1.78 – 1.67 (m, 1H), 1.33 –

1.28 (m, 2H); ^{13}C NMR (100.6 MHz, methanol-d_4) δ 134.4, 133.8, 133.1, 132.5, 131.9, 131.4, 129.1, 128.9, 127.9, 127.6, 127.5, 127.3, 127.2, 126.8, 126.2, 60.4, 59.7, 55.5, 51.7, 51.6, 39.9, 29.2, 27.3, 27.2, 27.1; IR ν_{max} (cm^{-1}) 3070, 2927, 1471, 1103, 790; UV (λ_{max} nm) 277; LC Trace: 100%; HRMS (ESI) *m/z* calculated for $C_{26}H_{31}Cl_2N_2$ $[M+H]^+$: 441.1786, found 441.1782.

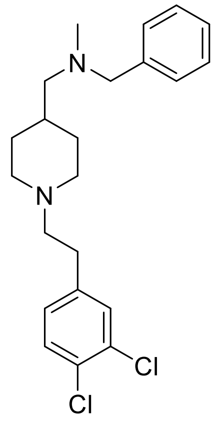

***N*-Benzyl-1-(1-(3,4-dichlorophenethyl)piperidin-4-yl)-*N*-methylmethanamine (NDM-37):** Compound **11b** was Boc-deprotected with trifluoroacetic acid in dichloromethane following the general synthetic procedure for Boc-deprotection and subsequently reacted with (bromomethyl)benzene and potassium carbonate in acetonitrile following the general procedure for amine alkylation. The product was transformed into a salt using the general procedure for making HCl salts. The product presented as a white solid (35%). ^{1}H NMR (400 MHz, methanol-d_4) δ 7.60 (dd, *J* = 6.9, 3.1 Hz, 2H), 7.57 – 7.51 (m, 5H), 7.28 (dd, *J* = 8.3, 2.1 Hz, 1H), 4.51 (d, *J* = 13.0 Hz, 1H), 4.35 (d, *J* = 12.9 Hz, 1H), 3.77 – 3.69 (m, 2H), 3.41 – 3.35 (m, 2H), 3.25 – 3.05 (m, 6H), 2.90 (s, 3H), 2.38 – 2.24 (m, 1H), 2.22 – 2.09 (m, 2H), 1.73 – 1.50 (m, 2H); ^{13}C NMR (100.6 MHz, methanol-d_4) δ 137.0, 132.3, 131.7, 131.2, 130.9, 130.6, 130.6, 130.1, 129.1, 128.9, 128.5, 128.5, 60.2, 59.7, 57.1, 51.8, 39.8, 29.2, 28.9, 27.1; IR ν_{max} (cm^{-1}) 2925, 2487, 1470, 1032, 699; UV (λ_{max} nm) 280; LC Trace: 100%; HRMS (ESI) *m/z* calculated for $C_{22}H_{29}Cl_2N_2$ $[M+H]^+$: 391.1702, found 391.1693.

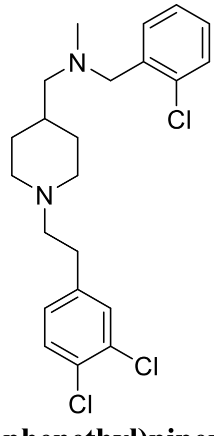

***N*-(2-Chlorobenzyl)-1-(1-(3,4-dichlorophenethyl)piperidin-4-yl)-*N*-methylmethanamine (NDM-36):** Compound **11b** was Boc-deprotected with trifluoroacetic acid in dichloromethane following the general synthetic procedure for Boc-deprotection and subsequently reacted with 1-(bromomethyl)-2-chlorobenzene and potassium carbonate in acetonitrile following the general procedure for amine alkylation. The product was transformed into a salt using the general

procedure for making HCl salts. The product presented as a white solid (55%). ^{1}H NMR (400 MHz, methanol-d$_4$) δ 7.49 (dd, *J* = 7.3, 2.1 Hz, 1H), 7.46 – 7.40 (m, 2H), 7.36 (dd, *J* = 7.6, 1.7 Hz, 1H), 7.32 – 7.19 (m, 2H), 7.16 (dd, *J* = 8.2, 2.1 Hz, 1H), 3.59 (s, 2H), 3.04 (d, *J* = 11.3 Hz, 2H), 2.87 – 2.77 (m, 2H), 2.70 – 2.60 (m, 2H), 2.29 (d, *J* = 7.2 Hz, 2H), 2.22 (s, 3H), 2.19 – 2.11 (m, 2H), 1.88 (d, *J* = 12.9 Hz, 2H), 1.64 (ddt, *J* = 11.1, 7.5, 3.8 Hz, 1H), 1.26 – 1.23 (m, 2H); ^{13}C NMR (100.6 MHz, methanol-d$_4$) δ 140.6, 136.5, 134.1, 131.8, 131.0, 130.2, 129.7, 129.1, 128.4, 128.3, 128.1, 126.4, 63.4, 59.5, 59.0, 53.1, 41.8, 33.4, 31.4, 29.9; IR ν_{max} (cm^{-1}) 3422, 2924, 2461, 1471, 1446, 1032, 754; UV (λ_{max} nm) 282; LC Trace: 100%; HRMS (ESI) *m/z* calculated for $C_{22}H_{28}Cl_3N_2$ [M+H]$^+$: 425.1313, found 425.1302.

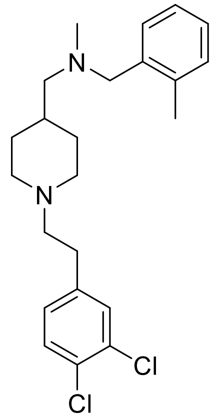

1-(1-(3,4-Dichlorophenethyl)piperidin-4-yl)-*N*-methyl-*N*-(2-methylbenzyl)methanamine (NDM-38): Compound **11b** was Boc-deprotected with trifluoroacetic acid in dichloromethane following the general synthetic procedure for Boc-deprotection and subsequently reacted with 1-(bromomethyl)-2-methylbenzene and potassium carbonate in acetonitrile following the general procedure for amine alkylation. The product was transformed into a salt using the general procedure for making HCl salts. The product presented as a white solid (30%). ^{1}H NMR (400 MHz, methanol-d$_4$) δ 7.46 – 7.37 (m, 2H), 7.21 (dd, *J* = 6.5, 1.9 Hz, 1H), 7.20 – 7.06 (m, 4H), 3.46 (s, 2H), 3.09 (d, *J* = 11.7 Hz, 2H), 2.89 – 2.81 (m, 2H), 2.78 – 2.67 (m, 2H), 2.36 (s, 2H), 2.32 (d, *J* = 4.6 Hz, 1H), 2.30 – 2.27 (m, 1H), 2.25 (d, *J* = 7.1 Hz, 2H), 2.18 (s, 3H), 1.94 – 1.82 (m, 2H), 1.66 (ddd, *J* = 11.2, 7.3, 3.8 Hz, 1H), 1.31 (d, *J* = 14.9 Hz, 1H), 1.27 – 1.12 (m, 2H); ^{13}C NMR (100.6 MHz, methanol-d$_4$) δ 140.0, 137.3, 136.8, 131.9, 130.4, 130.2, 129.9, 128.3, 127.3, 126.9, 125.5, 125.1, 61.9, 60.9, 59.1, 52.9, 41.5, 32.9, 31.0, 29.5, 18.0; IR ν_{max} (cm^{-1}) 2925, 2523, 1472, 1447, 1031, 745; UV (λ_{max} nm) 284; LC Trace: 100%; HRMS (ESI) *m/z* calculated for $C_{23}H_{31}Cl_2N_2$ [M+H]$^+$: 405.1859, found 405.1866.

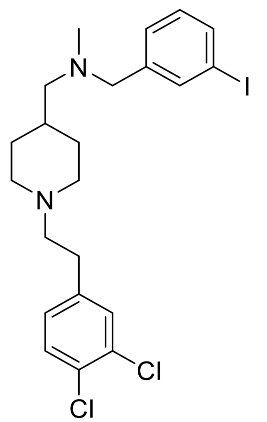

1-(1-(3,4-Dichlorophenethyl)piperidin-4-yl)-*N*-(3-iodobenzyl)-*N*-methylmethanamine (NDM-35): Compound **11b** was Boc-deprotected with trifluoroacetic acid in dichloromethane following the general synthetic procedure for Boc-deprotection and subsequently reacted with 1-(bromomethyl)-3-methylbenzene and potassium carbonate in acetonitrile following the general procedure for amine alkylation. The product was transformed into a salt using the general procedure for making HCl salts. The product presented as a white solid (29%). ^{1}H NMR (400 MHz, methanol-d$_4$) δ 7.72 (d, *J* = 1.9 Hz, 1H), 7.60 (d, *J* = 7.8 Hz, 1H), 7.49 – 7.39 (m, 2H), 7.31 (d, *J* = 7.9 Hz, 1H), 7.18 (dd, *J* = 8.3, 2.1 Hz, 1H), 7.09 (t, *J* = 7.7 Hz, 1H), 3.45 (s, 2H), 3.18 (d, *J* = 11.6 Hz, 2H), 2.91 – 2.83 (m, 2H), 2.83 – 2.77 (m, 2H), 2.38 (t, *J* = 11.9 Hz, 2H), 2.29 – 2.17 (m, 5H), 1.98 – 1.85 (m, 2H), 1.70 (ddd, *J* = 11.1, 7.3, 3.8 Hz, 1H), 1.34 – 1.19 (m, 2H); ^{13}C NMR (100.6 MHz, methanol-d$_4$) δ 141.7, 139.8, 137.7, 135.9, 131.9, 130.5, 130.3, 130.0, 129.8, 128.4, 128.1, 93.5, 62.6, 61.5, 58.9, 52.9, 32.8, 30.8, 29.3; IR ν_{max} (cm^{-1}) 3435, 2926, 2554, 1470, 1437, 1030, 775; UV (λ_{max} nm) 282; LC Trace: 100%; HRMS (ESI) *m/z* calculated for $C_{22}H_{28}Cl_2IN_2$ [M+H]$^+$: 517.0669, found 517.0661.

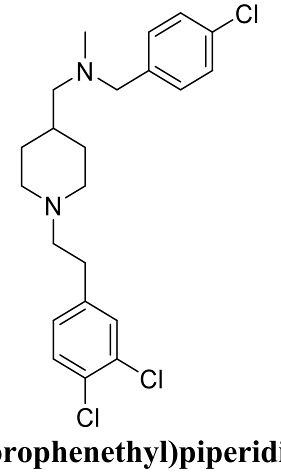

***N*-(4-Chlorobenzyl)-1-(1-(3,4-dichlorophenethyl)piperidin-4-yl)-*N*-methylmethanamine (NDM-41):** Compound **11b** was Boc-deprotected with trifluoroacetic acid in dichloromethane following the general synthetic procedure for Boc-deprotection and subsequently reacted with 1-(bromomethyl)-4-chlorobenzene and potassium carbonate in acetonitrile following the general procedure for amine alkylation. The product was transformed into a salt using the general procedure for making HCl salts. The product presented as a white solid (34%). ^{1}H NMR (400 MHz, methanol-d$_4$) δ 7.46 – 7.42 (m, 2H), 7.34 – 7.31 (m, 4H), 7.18 (dd, *J* = 8.2, 2.1 Hz, 1H), 3.49 (s, 2H), 3.09 (d, *J* = 11.4 Hz, 2H), 2.89 – 2.80 (m, 2H), 2.73 – 2.65 (m, 2H), 2.27 – 2.17 (m, 7H),

1.88 (d, J = 13.4 Hz, 2H), 1.66 (ddd, J = 11.2, 7.4, 3.8 Hz, 1H), 1.27 – 1.16 (m, 2H); ^{13}C NMR (100.6 MHz, methanol-d$_4$) δ 140.5, 137.7, 132.4, 131.8, 130.4, 130.4, 130.2, 129.7, 129.3, 128.4, 128.3, 127.9, 62.9, 61.5, 59.4, 53.0, 41.7, 33.2, 31.3, 29.8; IR ν_{max} (cm^{-1}) 3363, 2933, 2552, 1471, 1435, 1031, 820; UV (λ_{max} nm) 252; LC Trace: 100%; HRMS (ESI) *m/z* calculated for $C_{22}H_{28}Cl_3N_2$ [M+H]$^+$: 425.1313, found 425.1306.

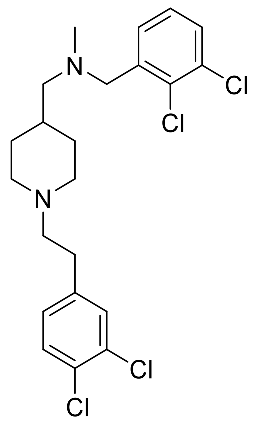

***N*-(2,3-Dichlorobenzyl)-1-(1-(3,4-dichlorophenethyl)piperidin-4-yl)-*N*-methylmethanamine (NDM-210):** Compound **11b** was Boc-deprotected with trifluoroacetic acid in dichloromethane following the general synthetic procedure for Boc-deprotection and subsequently reacted with 1-(bromomethyl)-2,3-dichlorobenzene and potassium carbonate in acetonitrile following the general procedure for amine alkylation. The product was transformed into a salt using the general procedure for making HCl salts. The product presented as a white solid (32%). ^{1}H NMR (400 MHz, methanol-d$_4$) δ 7.48 – 7.40 (m, 4H), 7.26 (t, J = 8.1 Hz, 1H), 7.18 (dd, J = 8.2, 2.2 Hz, 1H), 3.62 (s, 2H), 3.19 (d, J = 11.6 Hz, 2H), 2.91 – 2.76 (m, 4H), 2.39 (t, J = 12.0 Hz, 2H), 2.31 (dd, J = 7.1, 1.9 Hz, 2H), 2.26 – 2.17 (m, 3H), 1.93 (d, J = 13.7 Hz, 2H), 1.78 – 1.64 (m, 1H), 1.33 – 1.21 (m, 2H); ^{13}C NMR (100.6 MHz, methanol-d$_4$) δ 139.7, 139.3, 132.6, 132.0, 131.9, 130.5, 130.3, 130.0, 129.1, 128.8, 128.4, 127.0, 63.1, 59.9, 58.8, 52.9, 41.7, 33.0, 30.8, 29.3; IR ν_{max} (cm^{-1}) 3410, 2924, 2467, 1427, 1032, 786; UV (λ_{max} nm) 280; LC Trace: 100%; HRMS (ESI) *m/z* calculated for $C_{22}H_{27}Cl_4N_2$ [M+H]$^+$: 459.0923, found 459.0922.

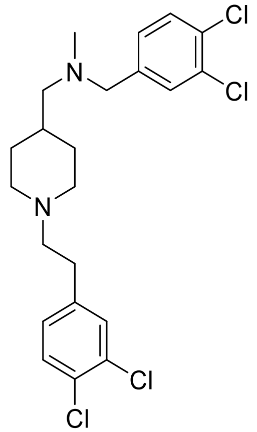

***N*-(3,4-Dichlorobenzyl)-1-(1-(3,4-dichlorophenethyl)piperidin-4-yl)-*N*-methylmethanamine (NDM-40):** Compound **11b** was Boc-deprotected with trifluoroacetic acid in dichloromethane following the general synthetic procedure for Boc-deprotection and subsequently reacted with 1-

(bromomethyl)-3,4-dichlorobenzene and potassium carbonate in acetonitrile following the general procedure for amine alkylation. The product was transformed into a salt using the general procedure for making HCl salts. The product presented as a white solid (32%). ^{1}H NMR (400 MHz, methanol-d$_4$) δ 7.51 – 7.48 (m, 1H), 7.47 – 7.42 (m, 3H), 7.29 – 7.21 (m, 1H), 7.18 (dd, *J* = 8.3, 2.1 Hz, 1H), 3.47 (s, 2H), 3.20 (d, *J* = 11.6 Hz, 2H), 2.93 – 2.77 (m, 4H), 2.40 (dt, *J* = 11.5, 6.3 Hz, 2H), 2.28 – 2.16 (m, 5H), 1.97 – 1.87 (m, 2H), 1.77 – 1.64 (m, 1H), 1.32 – 1.20 (m, 2H); ^{13}C NMR (100.6 MHz, methanol-d$_4$) δ 140.2, 139.7, 131.9, 131.7, 131.0, 130.5, 130.3, 130.0, 130.0, 128.4, 128.4, 62.6, 61.0, 58.8, 52.9, 41.6, 32.8, 30.7, 29.2, 27.6; IR ν_{max} (cm^{-1}) 3427, 2929, 2665, 2524, 1472, 1136, 1032, 814; UV (λ_{max} nm) 280; LC Trace: 100%; HRMS (ESI) *m/z* calculated for $C_{22}H_{27}Cl_4N_2$ [M+H]$^+$: 459.0923, found 459.0913.

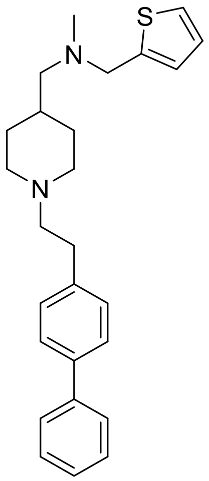

1-(1-(2-([1,1'-Biphenyl]-4-yl)ethyl)piperidin-4-yl)-*N*-methyl-*N*-(thiophen-2-ylmethyl)methanamine (NDM-592): Compound **11c** was Boc-deprotected with trifluoroacetic acid in dichloromethane following the general synthetic procedure for Boc-deprotection and subsequently reacted with 2-(bromomethyl)thiophene and potassium carbonate in acetonitrile following the general procedure for amine alkylation. The product was transformed into a salt using the general procedure for making HCl salts. The product presented as a white solid (15%). ^{1}H NMR (400 MHz, methanol-d$_4$) δ 7.62 – 7.50 (m, 4H), 7.41 (dd, *J* = 8.4, 6.9 Hz, 2H), 7.34 – 7.26 (m, 4H), 6.97 – 6.90 (m, 2H), 3.72 (s, 2H), 3.09 (d, *J* = 11.6 Hz, 2H), 2.92 – 2.83 (m, 2H), 2.72 – 2.62 (m, 2H), 2.26 (d, *J* = 7.2 Hz, 2H), 2.24 (s, 3H), 2.23 – 2.13 (m, 2H), 1.91 – 1.81 (m, 2H), 1.67 – 1.61 (m, 1H), 1.30 – 1.19 (m, 2H); ^{13}C NMR (100.6 MHz, methanol-d$_4$) δ 140.5, 140.2, 135.2, 132.9, 129.5, 129.1, 128.9, 128.5, 127.6, 127.1, 127.1, 126.5, 59.1, 57.8, 53.7, 51.8, 39.5, 29.6, 29.3, 27.0; IR ν_{max} (cm^{-1}) 2924, 2853, 1487, 1458, 1174; UV (λ_{max} nm) 284; LC Trace: 100%; HRMS (ESI) *m/z* calculated for $C_{26}H_{33}N_2S$ [M+H]$^+$: 405.2355, found 405.2359.

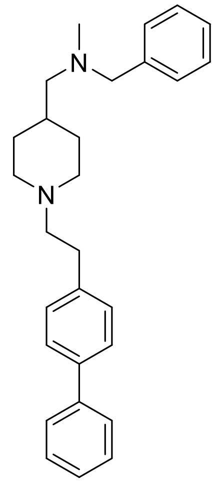

1-(1-(2-([1,1'-Biphenyl]-4-yl)ethyl)piperidin-4-yl)-*N*-benzyl-*N*-methylmethanamine (NDM-591): Compound **11c** was Boc-deprotected with trifluoroacetic acid in dichloromethane following the general synthetic procedure for Boc-deprotection and subsequently reacted with (bromomethyl)benzene and potassium carbonate in acetonitrile following the general procedure for amine alkylation. The product was transformed into a salt using the general procedure for making HCl salts. The product presented as a white solid (45%). ^{1}H NMR (400 MHz, methanol-d_4) δ 7.63 – 7.51 (m, 4H), 7.41 (t, *J* = 7.6 Hz, 2H), 7.30 (td, *J* = 7.9, 3.1 Hz, 7H), 7.24 (dd, *J* = 6.2, 2.6 Hz, 1H), 3.49 (s, 2H), 3.06 (d, *J* = 11.4 Hz, 2H), 2.91 – 2.82 (m, 2H), 2.65 (dd, *J* = 10.6, 6.2 Hz, 2H), 2.23 (d, *J* = 7.1 Hz, 2H), 2.20 (s, 3H), 2.14 (d, *J* = 11.8 Hz, 2H), 1.91 – 1.82 (m, 2H), 1.64 (t, *J* = 11.1 Hz, 1H), 1.30 – 1.18 (m, 2H).; ^{13}C NMR (126 MHz, methanol-d_4) δ 141.0, 139.3, 139.1, 138.8, 129.1, 129.0, 128.7, 128.0, 126.9, 126.9, 126.9, 126.6, 63.2, 62.5, 60.5, 53.4, 42.1, 33.6, 32.3, 30.2; IR ν_{max} (cm^{-1}) 2935, 2793, 2756, 1489, 1123; UV (λ_{max} nm) 286; LC Trace: 100%; HRMS (ESI) *m/z* calculated for $C_{28}H_{35}N_2$ $[M+H]^+$: 399.2798, found 399.2795.

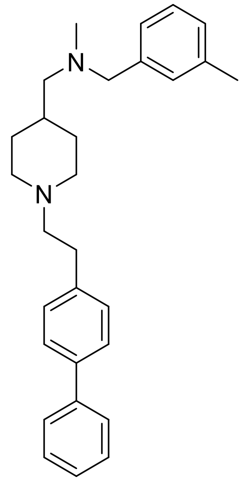

1-(1-(2-([1,1'-Biphenyl]-4-yl)ethyl)piperidin-4-yl)-*N*-methyl-*N*-(3-methylbenzyl)methanamine (NDM-604): Compound **11c** was Boc-deprotected with trifluoroacetic acid in dichloromethane following the general synthetic procedure for Boc-deprotection and subsequently reacted with 1-(bromomethyl)-3-methylbenzene and potassium carbonate in acetonitrile following the general procedure for amine alkylation. The product was transformed into a salt using the general procedure for making HCl salts. The product presented

as a white solid (39%). ^{1}H NMR (400 MHz, chloroform-d) δ 7.62 – 7.56 (m, 2H), 7.56 – 7.50 (m, 2H), 7.44 (dd, *J* = 8.4, 6.9 Hz, 2H), 7.37 – 7.31 (m, 1H), 7.31 – 7.27 (m, 2H), 7.21 (t, *J* = 7.5 Hz, 1H), 7.16 – 7.09 (m, 2H), 7.06 (d, *J* = 7.4 Hz, 1H), 3.44 (s, 2H), 3.08 (dd, *J* = 10.8, 4.3 Hz, 2H), 2.94 – 2.85 (m, 2H), 2.71 – 2.61 (m, 2H), 2.36 (s, 3H), 2.24 (d, *J* = 7.2 Hz, 2H), 2.19 (s, 3H), 2.14 – 2.02 (m, 2H), 1.91 – 1.82 (m, 2H), 1.59 (ddd, *J* = 11.2, 7.4, 3.8 Hz, 1H), 1.36 – 1.27 (m, 2H). ^{13}C NMR (100.6 MHz, methanol-d$_4$) δ 140.5, 140.2, 139.2, 135.2, 131.7, 130.7, 128.9, 128.9, 128.5, 128.14, 127.1, 127.1, 126.5, 60.2, 59.8, 57.8, 51.8, 39.8, 29.6, 29.3, 27.1, 19.9; IR ν_{max} (cm^{-1})2970, 2922, 1489, 1438, 1066; UV (λ_{max} nm) 286; LC Trace: 100%; HRMS (ESI) *m/z* calculated for $C_{29}H_{37}N_2$ [M+H]$^+$: 413.2879, found 413.2878.

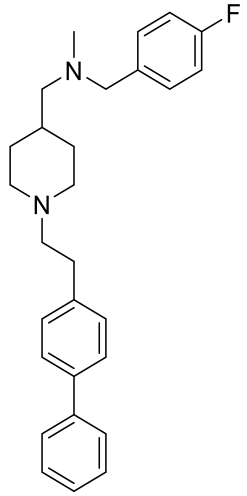

1-(1-(2-([1,1'-Biphenyl]-4-yl)ethyl)piperidin-4-yl)-*N*-(4-fluorobenzyl)-*N*-methylmethanamine (NDM-606): Compound **11c** was Boc-deprotected with trifluoroacetic acid in dichloromethane following the general synthetic procedure for Boc-deprotection and subsequently reacted with 1-(bromomethyl)-4-fluorobenzene and potassium carbonate in acetonitrile following the general procedure for amine alkylation. The product was transformed into a salt using the general procedure for making HCl salts. The product presented as a white solid (50%). ^{1}H NMR (400 MHz, methanol-d$_4$) δ 7.60 – 7.54 (m, 2H), 7.54 – 7.48 (m, 2H), 7.39 (dd, *J* = 8.4, 6.9 Hz, 2H), 7.33 – 7.27 (m, 3H), 7.27 – 7.21 (m, 2H), 7.05 – 6.97 (m, 2H), 3.41 (s, 2H), 3.01 (dt, *J* = 11.8, 3.5 Hz, 2H), 2.85 – 2.77 (m, 2H), 2.65 – 2.56 (m, 2H), 2.18 (d, *J* = 7.1 Hz, 2H), 2.14 (s, 3H), 2.09 (td, *J* = 11.9, 2.5 Hz, 2H), 1.85 – 1.75 (m, 2H), 1.56 (td, *J* = 7.4, 3.7 Hz, 1H), 1.28 – 1.10 (m, 2H); ^{13}C NMR (100.6 MHz, methanol-d$_4$) δ 163.9 (d, *J* = 249.5 Hz), 140.5, 140.2, 135.3, 133.7 (d, *J* = 8.0 Hz), 129,0, 128.9, 128.5, 127.1, 127.0, 126.5, 125.0 (d, *J* = 4.0 Hz), 115.9 (d, *J* = 21.1 Hz), 59.7, 59.3, 57.8, 51.8, 39.6, 29.6, 29.2, 27.2; IR ν_{max} (cm^{-1}) 2924, 2850, 1489, 1458, 1075; UV (λ_{max} nm) 284; LC Trace: 100%; HRMS (ESI) *m/z* calculated for $C_{28}H_{34}FN_2$ [M+H]$^+$: 417.2628, found 417.2629.

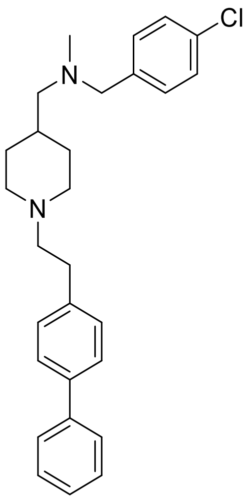

1-(1-(2-([1,1'-Biphenyl]-4-yl)ethyl)piperidin-4-yl)-*N*-(4-chlorobenzyl)-*N*-methylmethanamine (NDM-565): Compound **11c** was Boc-deprotected with trifluoroacetic acid in dichloromethane following the general synthetic procedure for Boc-deprotection and subsequently reacted with 1-(bromomethyl)-4-chlorobenzene and potassium carbonate in acetonitrile following the general procedure for amine alkylation. The product was transformed into a salt using the general procedure for making HCl salts. The product presented as a white solid (31%). ^{1}H NMR (400 MHz, methanol-d$_4$) δ 7.58 (td, *J* = 8.5, 1.7 Hz, 4H), 7.42 (dd, *J* = 8.4, 6.9 Hz, 2H), 7.32 (d, *J* = 8.3 Hz, 7H), 3.47 (s, 2H), 3.27 (d, *J* = 11.6 Hz, 2H), 2.97 – 2.91 (m, 4H), 2.49 (td, *J* = 12.0, 2.6 Hz, 2H), 2.24 (d, *J* = 7.2 Hz, 2H), 2.20 (d, *J* = 2.3 Hz, 3H), 1.99 – 1.90 (m, 2H), 1.74 (ddd, *J* = 11.2, 7.4, 3.8 Hz, 1H), 1.28 (ddd, *J* = 8.7, 5.9, 2.4 Hz, 2H); ^{13}C NMR (126 MHz, methanol-d$_4$) δ 140.9, 139.6, 138.0, 137.9, 132.6, 130.6, 129.0, 128.7, 128.1, 127.1, 127.1, 126.7, 62.8, 61.7, 59.7, 53.1, 41.9, 32.9, 31.6, 29.4.; IR ν_{max} (cm^{-1}) 2944, 2792, 2766, 1488, 1126; UV (λ_{max} nm) 284; LC Trace: 100%; HRMS (ESI) *m/z* calculated for $C_{28}H_{34}ClN_2$ [M+H]$^+$: 433.2406, found 433.2405.

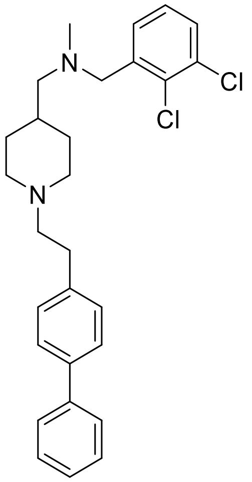

1-(1-(2-([1,1'-Biphenyl]-4-yl)ethyl)piperidin-4-yl)-*N*-(2,3-dichlorobenzyl)-*N*-methylmethanamine (NDM-594): Compound **11c** was Boc-deprotected with trifluoroacetic acid in dichloromethane following the general synthetic procedure for Boc-deprotection and subsequently reacted with 1-(bromomethyl)-2,3-dichlorobenzene and potassium carbonate in acetonitrile following the general procedure for amine alkylation. The product was transformed

into a salt using the general procedure for making HCl salts. The product presented as a white solid (14%). ^{1}H NMR (400 MHz, methanol-d$_4$) δ 7.52 – 7.46 (m, 4H), 7.37 – 7.29 (m, 4H), 7.28 – 7.21 (m, 3H), 7.21 – 7.14 (m, 1H), 3.54 (s, 2H), 3.38 (d, *J* = 12.1 Hz, 2H), 3.11 – 3.03 (m, 2H), 2.98 – 2.90 (m, 2H), 2.77 – 2.65 (m, 2H), 2.25 (d, *J* = 7.3 Hz, 2H), 2.14 (s, 3H), 1.95 (dd, *J* = 14.5, 3.2 Hz, 2H), 1.81 – 1.69 (m, 1H), 1.41 – 1.29 (m, 2H); ^{13}C NMR (126 MHz, methanol-d$_4$) δ 140.8, 140.1, 139.5, 129.3, 129.1, 129.1, 128.7, 127.2, 127.2, 126.7, 62.8, 60.1, 58.4, 52.8, 41.8, 32.3, 30.5, 28.4; IR ν_{max} (cm^{-1}) 2923, 2795, 2636, 1488, 1124; UV (λ_{max} nm) 284; LC Trace: 100%; HRMS (ESI) *m/z* calculated for $C_{28}H_{33}Cl_2N_2$ [M+H]$^+$: 467.1198, found 467.2015.

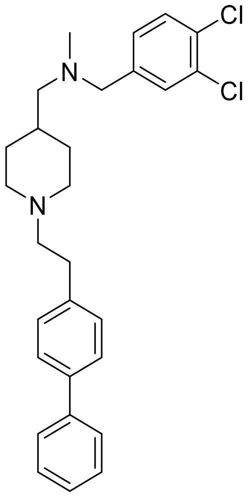

1-(1-(2-([1,1'-Biphenyl]-4-yl)ethyl)piperidin-4-yl)-*N*-(3,4-dichlorobenzyl)-*N*-methylmethanamine (NDM-605): Compound **11c** was Boc-deprotected with trifluoroacetic acid in dichloromethane following the general synthetic procedure for Boc-deprotection and subsequently reacted with 4-(bromomethyl)-1,2-dichlorobenzene and potassium carbonate in acetonitrile following the general procedure for amine alkylation. The product was transformed into a salt using the general procedure for making HCl salts. The product presented as a white solid (41%). ^{1}H NMR (400 MHz, methanol-d$_4$) δ 7.58 (ddd, *J* = 8.0, 5.0, 1.5 Hz, 4H), 7.51 (d, *J* = 2.4 Hz, 1H), 7.48 – 7.39 (m, 3H), 7.35 – 7.31 (m, 3H), 7.25 (d, *J* = 8.3 Hz, 1H), 3.47 (d, *J* = 3.2 Hz, 2H), 3.35 (d, *J* = 12.2 Hz, 2H), 3.04 – 2.93 (m, 4H), 2.61 (t, *J* = 12.3 Hz, 2H), 2.25 (dd, *J* = 7.3, 1.9 Hz, 2H), 2.23 – 2.15 (m, 3H), 1.98 (d, *J* = 14.1 Hz, 2H), 1.84 – 1.71 (m, 1H), 1.36 – 1.26 (m, 2H); ^{13}C NMR (126 MHz, methanol-d$_4$) δ 140.9, 140.5, 139.5, 130.7, 130.2, 129.0, 128.7, 127.0, 127.0, 126.6, 62.9, 61.3, 59.9, 53.2, 41.8, 33.2, 31.8, 29.7; IR ν_{max} (cm^{-1}) 2945, 2802, 2765, 1486, 1127; UV (λ_{max} nm) 286; LC Trace: 100%; HRMS (ESI) *m/z* calculated for $C_{28}H_{33}Cl_2N_2$ [M+H]$^+$: 477.2027, found 477.2015.

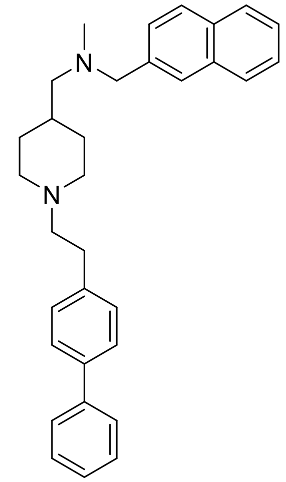

1-(1-(2-([1,1'-Biphenyl]-4-yl)ethyl)piperidin-4-yl)-*N*-methyl-*N*-(naphthalen-2-ylmethyl)methanamine (NDM-593): Compound **11c** was Boc-deprotected with trifluoroacetic acid in dichloromethane following the general synthetic procedure for Boc-deprotection and subsequently reacted with 2-(bromomethyl)naphthalene and potassium carbonate in acetonitrile following the general procedure for amine alkylation. The product was transformed into a salt using the general procedure for making HCl salts. The product presented as a white solid (18%). ^{1}H NMR (400 MHz, chloroform-d) δ 7.81 (ddd, *J* = 13.0, 6.6, 3.3 Hz, 4H), 7.71 (s, 1H), 7.59 – 7.55 (m, 2H), 7.54 – 7.50 (m, 2H), 7.50 – 7.46 (m, 2H), 7.46 – 7.40 (m, 3H), 7.34 (d, *J* = 7.4 Hz, 1H), 7.29 (s, 1H), 3.63 (s, 2H), 2.99 (d, *J* = 63.9 Hz, 4H), 2.68 (s, 2H), 2.30 (d, *J* = 6.8 Hz, 2H), 2.21 (s, 3H), 1.91 (s, 2H), 1.69 – 1.52 (m, 4H), 1.25 (s, 1H); ^{13}C NMR (126 MHz, chloroform-d) δ 141.1, 139.5, 137.3, 133.6, 132.9, 129.4, 128.9, 128.1, 128.1, 127.9, 127.9, 127.5, 127.5, 127.5, 127.4, 127.2, 126.4, 126.6, 126.1, 125.7, 125.6, 125.4, 65.6, 63.2, 60.6, 53.8, 43.1, 33.9, 32.7, 30.2; IR ν_{max} (cm^{-1}) 2922, 2851, 2806, 1458, 1066; UV (λ_{max} nm) 290; LC Trace: 100%; HRMS (ESI) *m/z* calculated for $C_{32}H_{37}N_2$ $[M+H]^+$: 449.2955, found 449.2951.

^{1}H and ^{13}C NMR spectra

***tert*-Butyl 3-(methyl(thiophen-3-ylmethyl)carbamoyl)azetidine-1-carboxylate (3):**

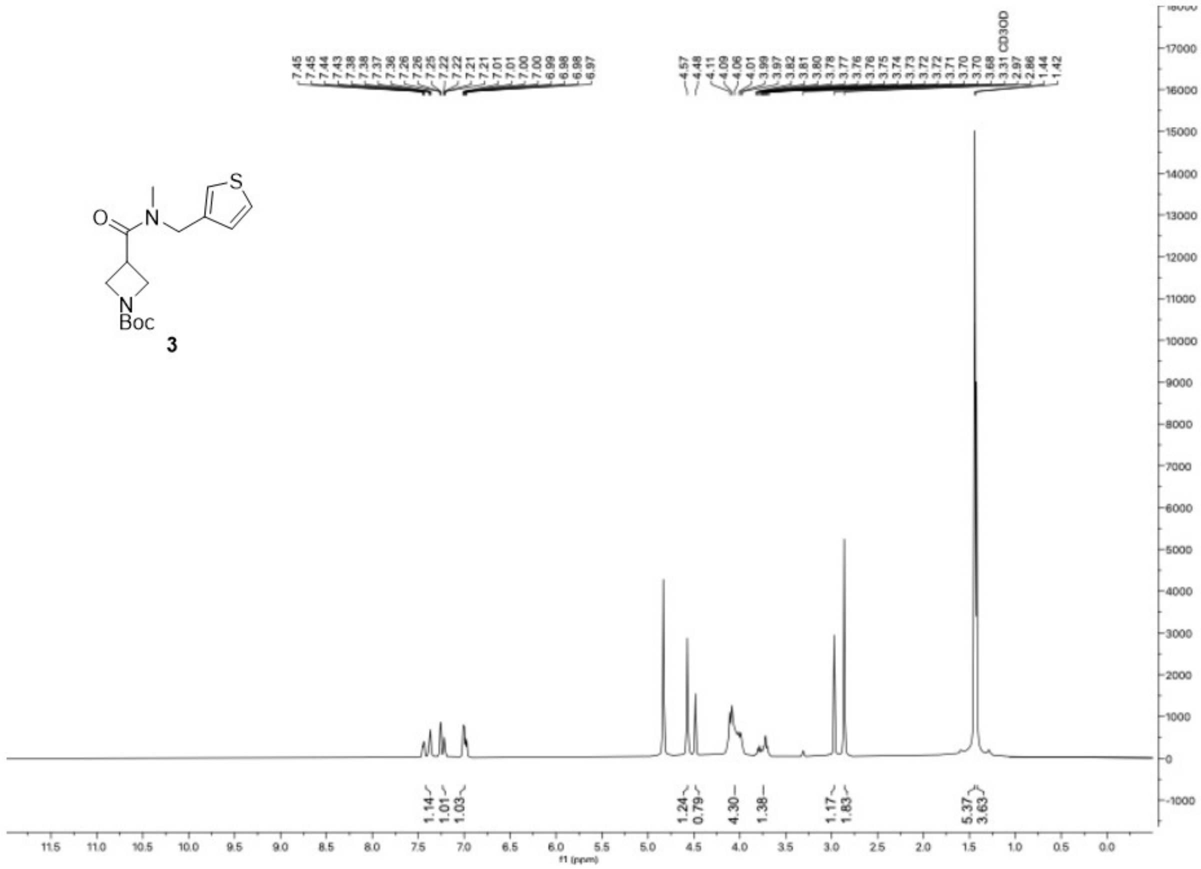

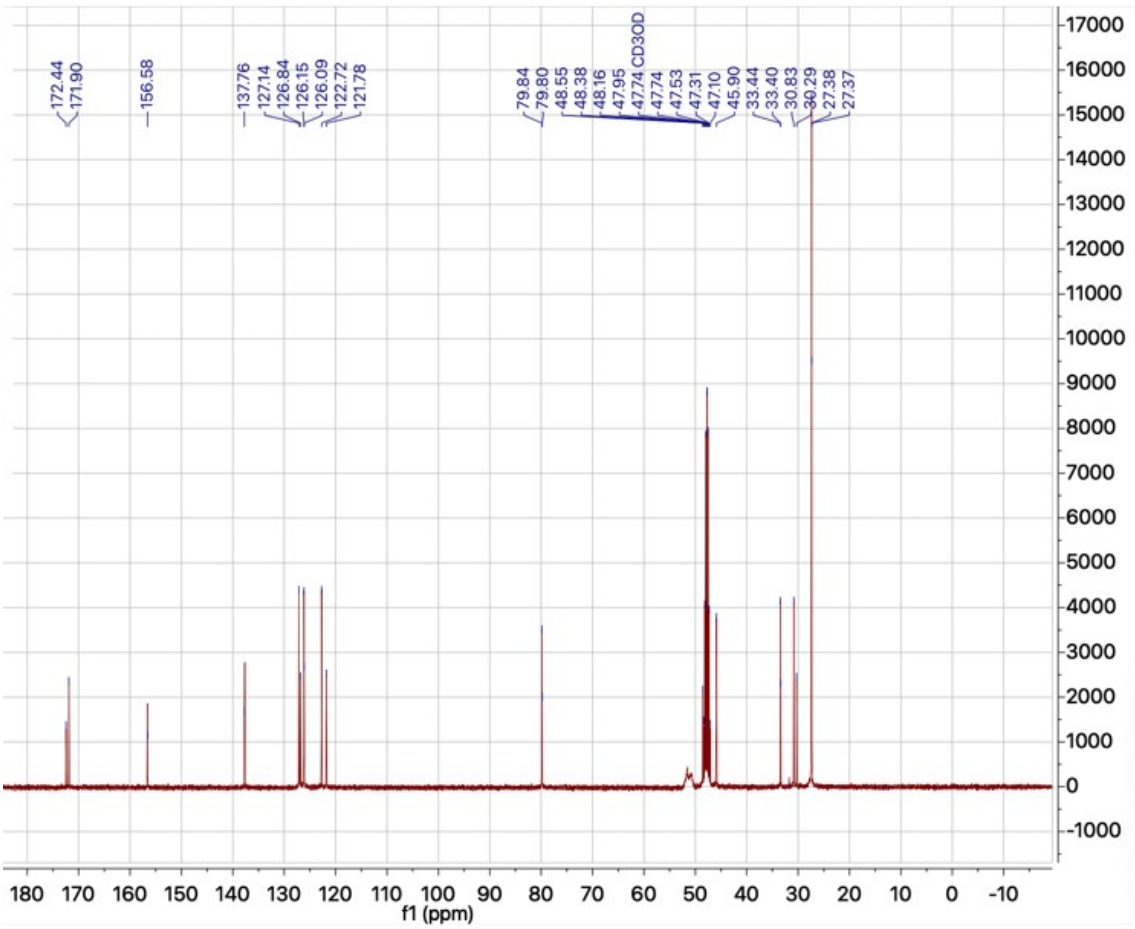
172.44
171.90
156.58
137.76
127.14
126.84
126.15
126.09
122.72
121.78
79.84
79.80
48.55
48.38
48.16
47.95
47.74 CD3OD
47.74
47.53
47.31
47.10
45.90
33.44
33.40
30.83
30.29
27.38
27.37
f1 (ppm)

1-(Azetidin-3-yl)-*N*-methyl-*N*-(thiophen-3-ylmethyl)methanamine (4):

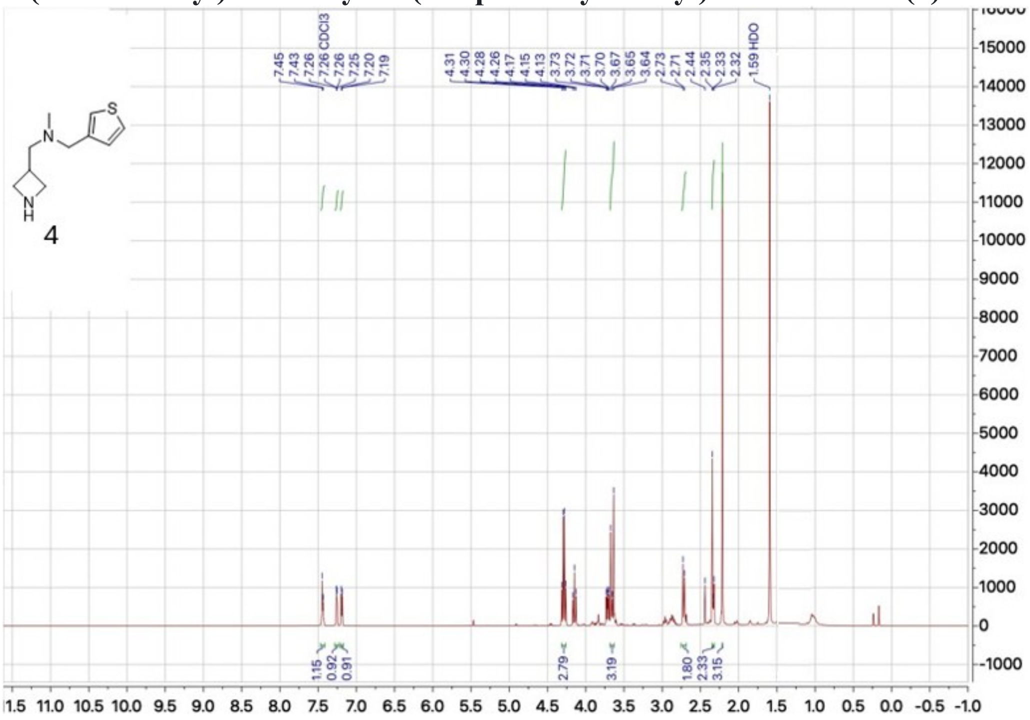

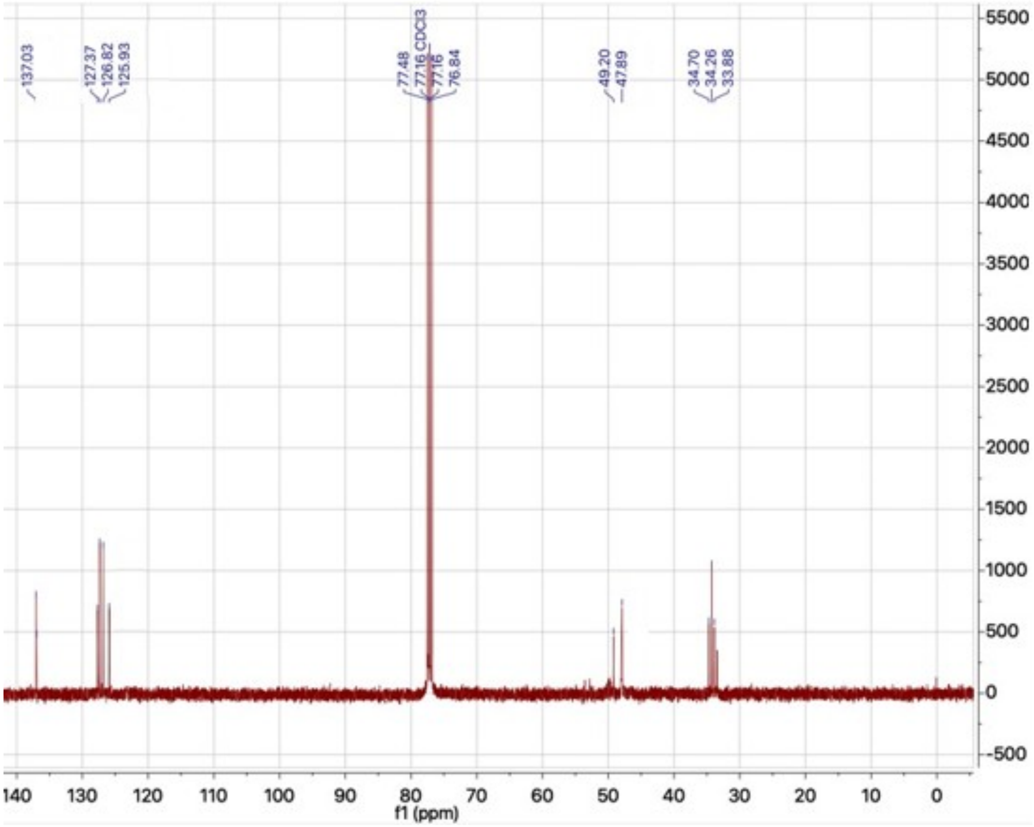

1-(1-(2-Fluorophenethyl)azetidin-3-yl)-*N*-methyl-*N*-(thiophen-3-ylmethyl)methanamine (NDM-506):

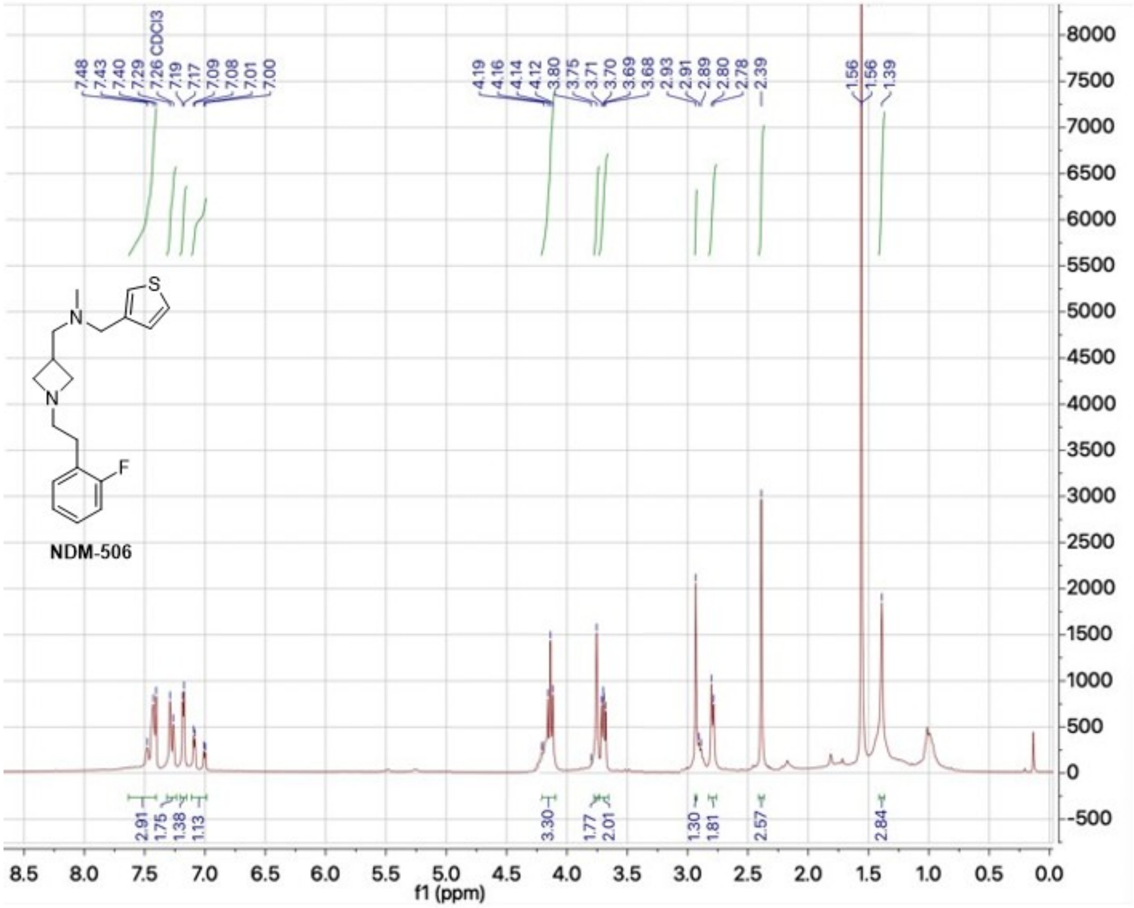

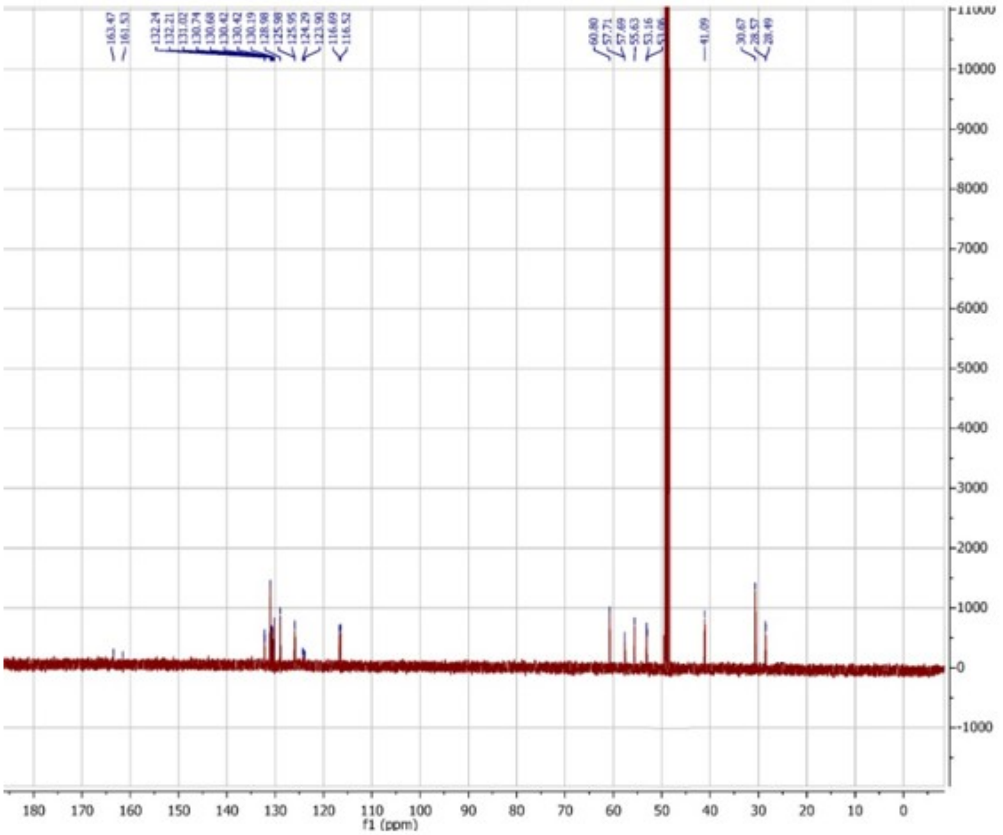

1-(1-(2-([1,1'-Biphenyl]-4-yl)ethyl)azetidin-3-yl)-*N*-methyl-*N*-(thiophen-3-ylmethyl)methanamine (NDM-507):

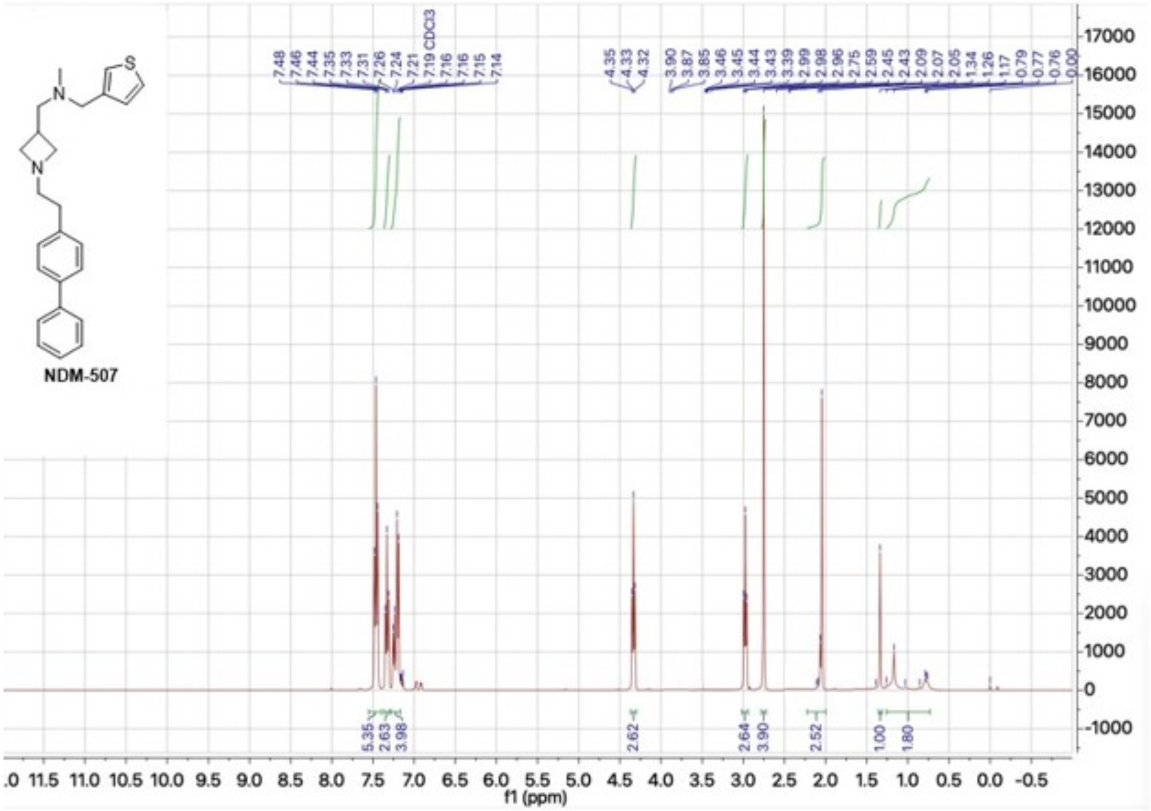
NDM-507
f1 (ppm)

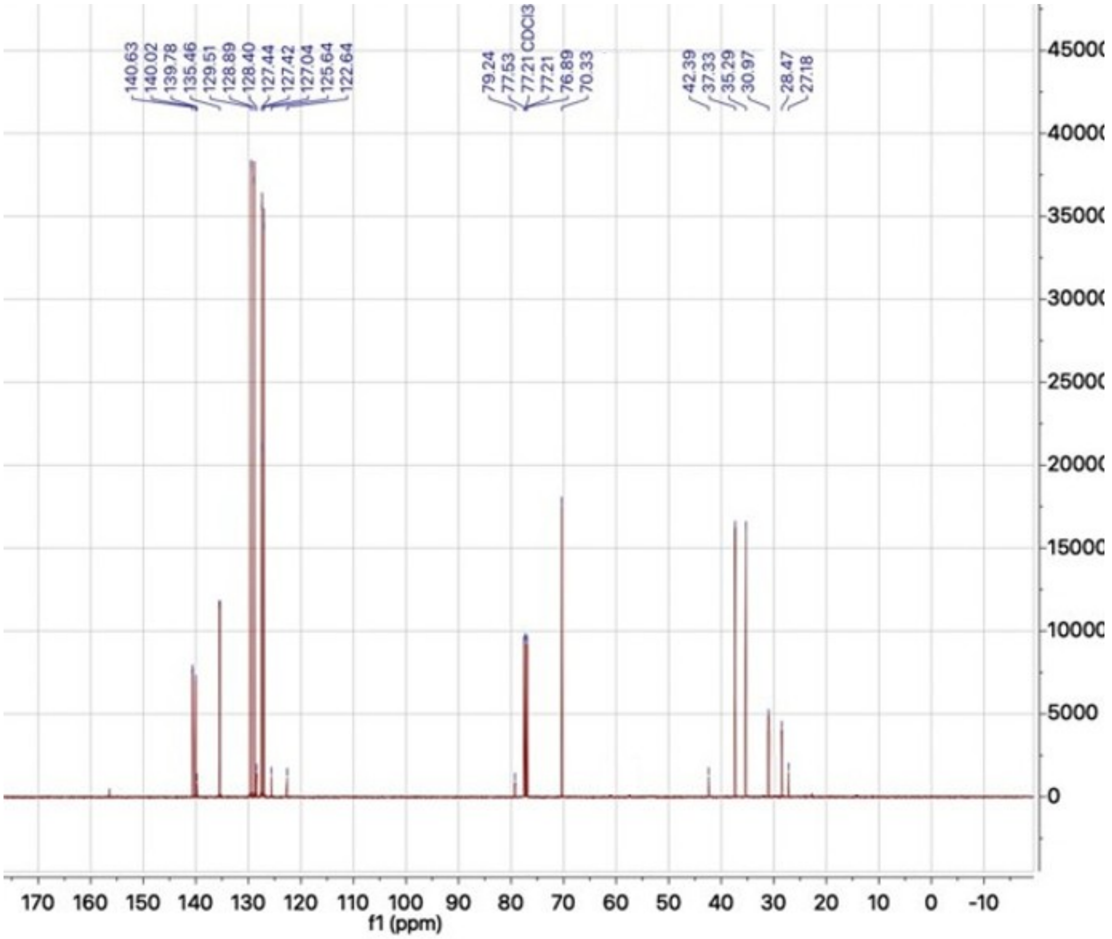

***N*-((1-(2-Fluorophenethyl)piperidin-4-yl)methyl)-*N*-methylthiophene-3-carboxamide (NDM-29):**

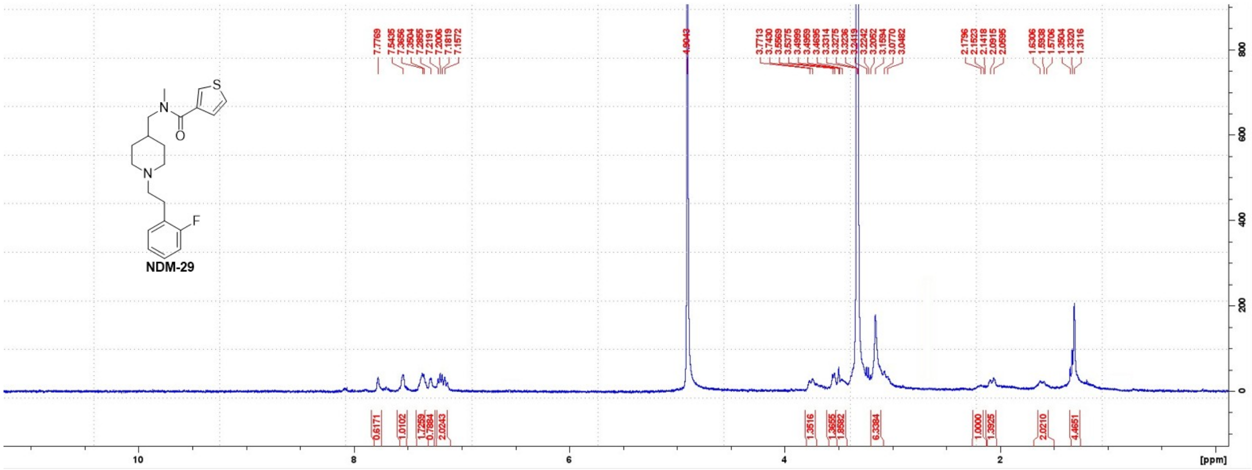
NDM-29
[ppm]

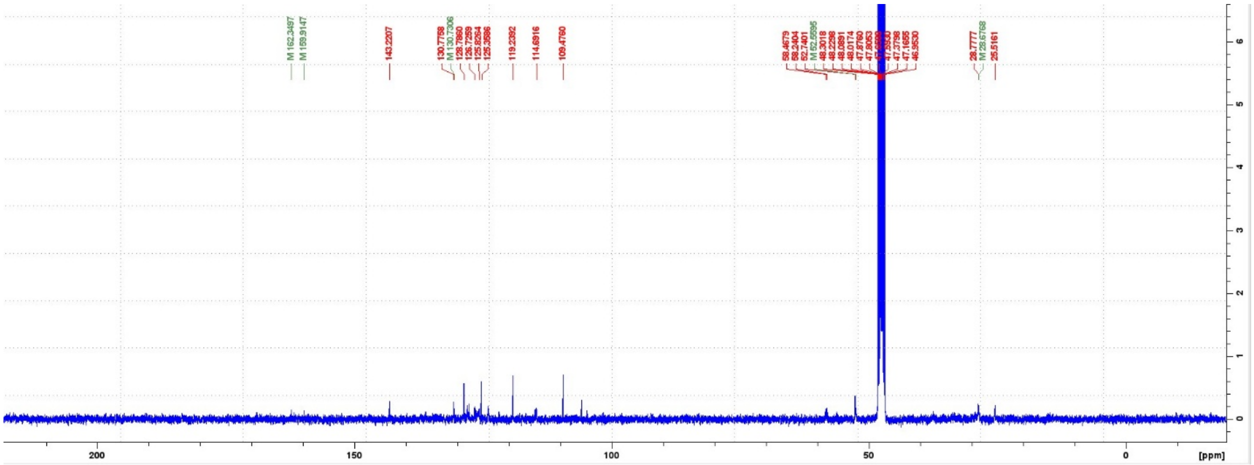

1-(1-(2-Fluorophenethyl)piperidin-4-yl)-*N*-methyl-*N*-(thiophen-2-ylmethyl)methanamine (NDM-559):

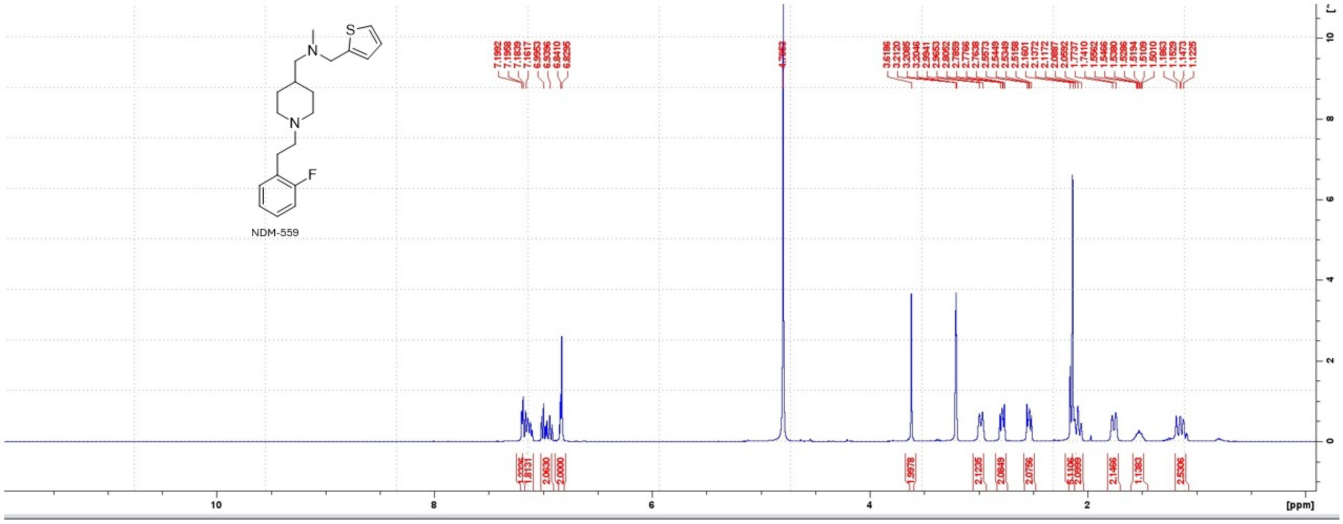

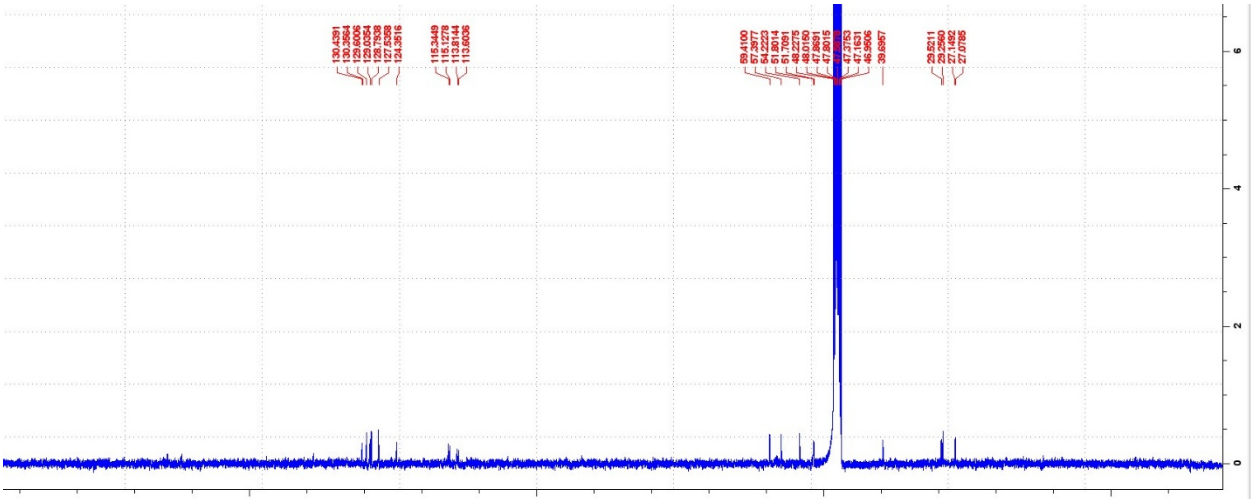

1-(1-(2-Fluorophenethyl)piperidin-4-yl)-*N*-(furan-3-ylmethyl)-*N*-methylmethanamine (NDM-560):

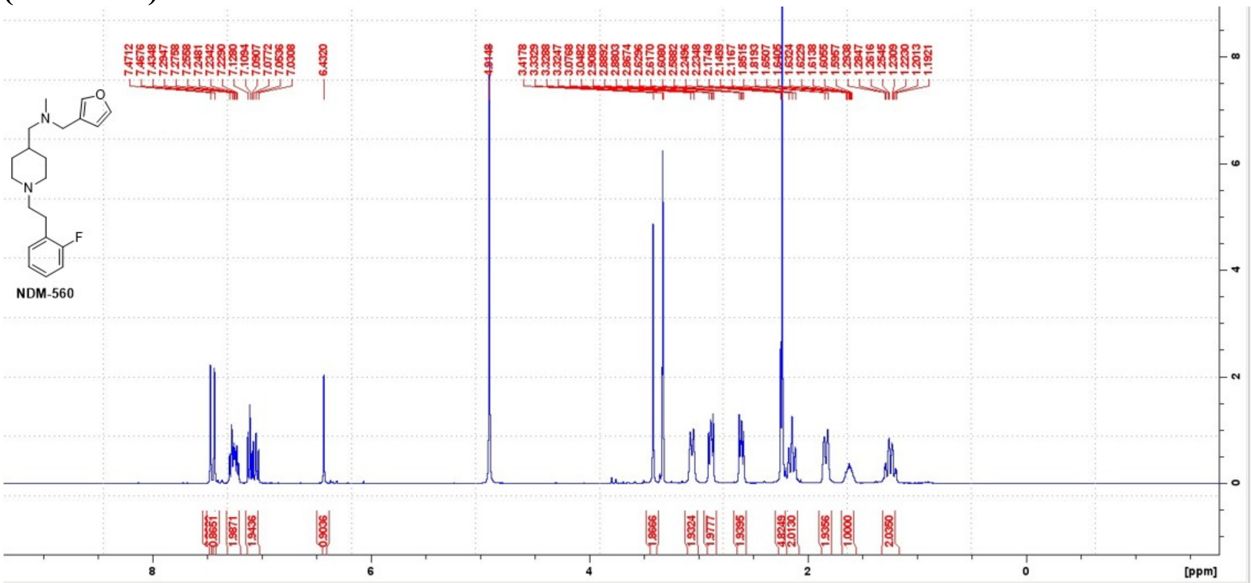

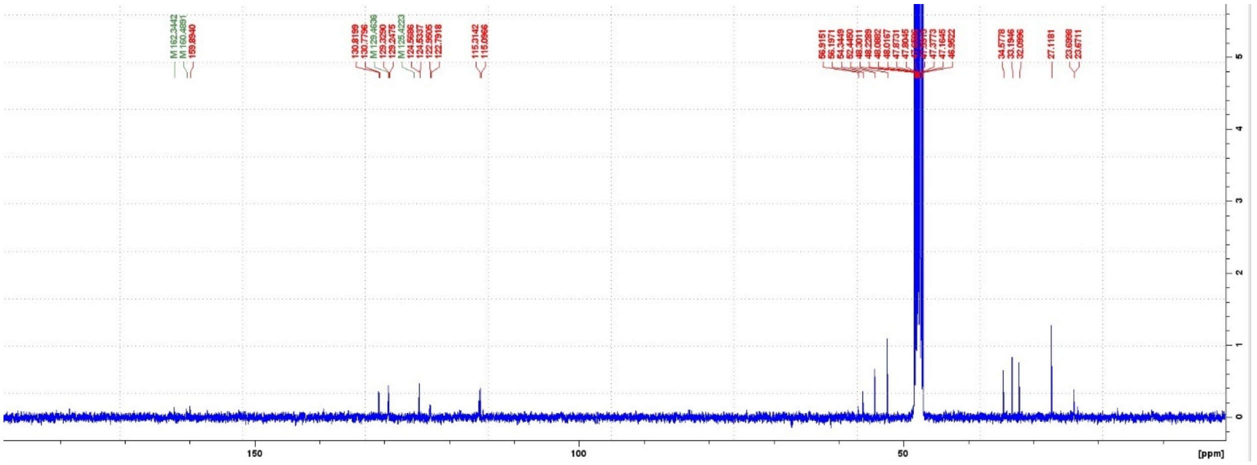

M 162.3442
M 160.4891
159.8940
130.8199
130.7796
M 129.4636
129.3290
129.2475
M 125.4223
124.5686
124.5337
122.9505
122.7918
115.3142
115.0966
56.9151
56.1971
54.3449
52.4450
48.3011
48.2289
48.0882
48.0167
47.8731
47.8045
47.3773
47.1645
46.9522
34.5778
33.1946
32.0996
27.1181
23.6998
23.6711
150
100
50
[ppm]

1-(1-(2-Fluorophenethyl)piperidin-4-yl)-*N*-methyl-*N*-(thiazol-4-ylmethyl)methanamine (NDM-597):

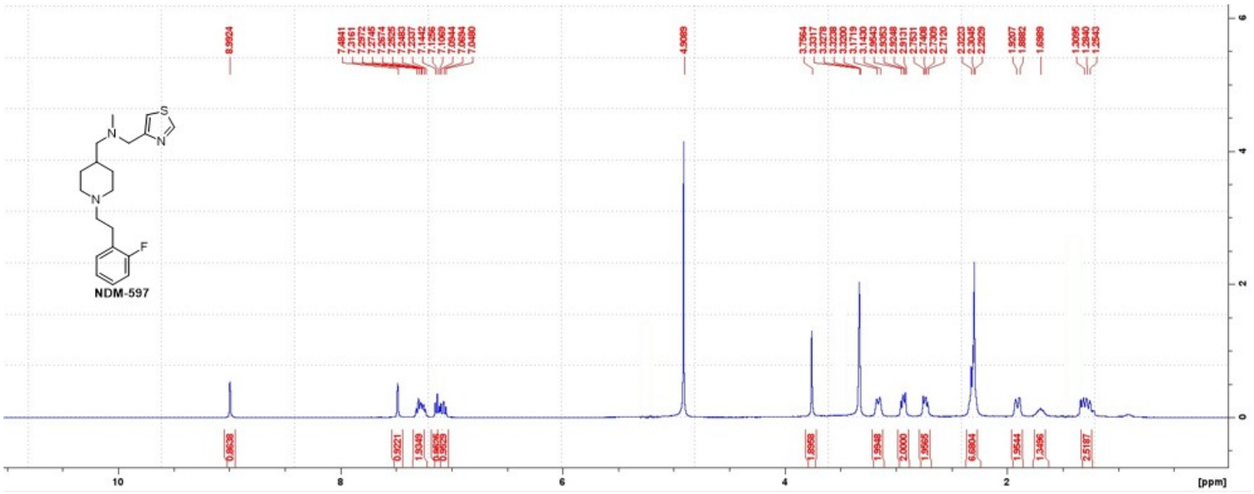

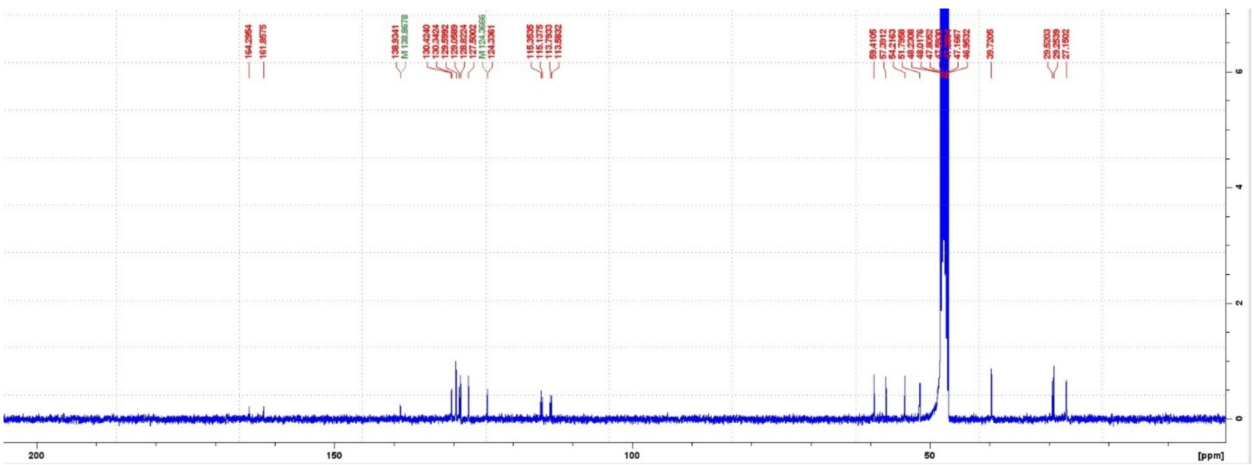

164.2954
161.8575
138.9341
M 138.8678
130.4240
130.3424
129.5992
129.0589
128.8224
127.5002
M 124.3666
124.3361
115.3535
115.1375
113.7933
113.5832
59.4105
57.3912
54.2163
51.7958
48.2308
48.0176
47.8052
47.1667
46.9532
39.7205
29.5203
29.2539
27.1502
200
150
100
50
[ppm]
0
2
4
6

1-(1-(2-Fluorophenethyl)piperidin-4-yl)-*N*-methyl-*N*-(pyridin-2-ylmethyl)methanamine (NDM-73):

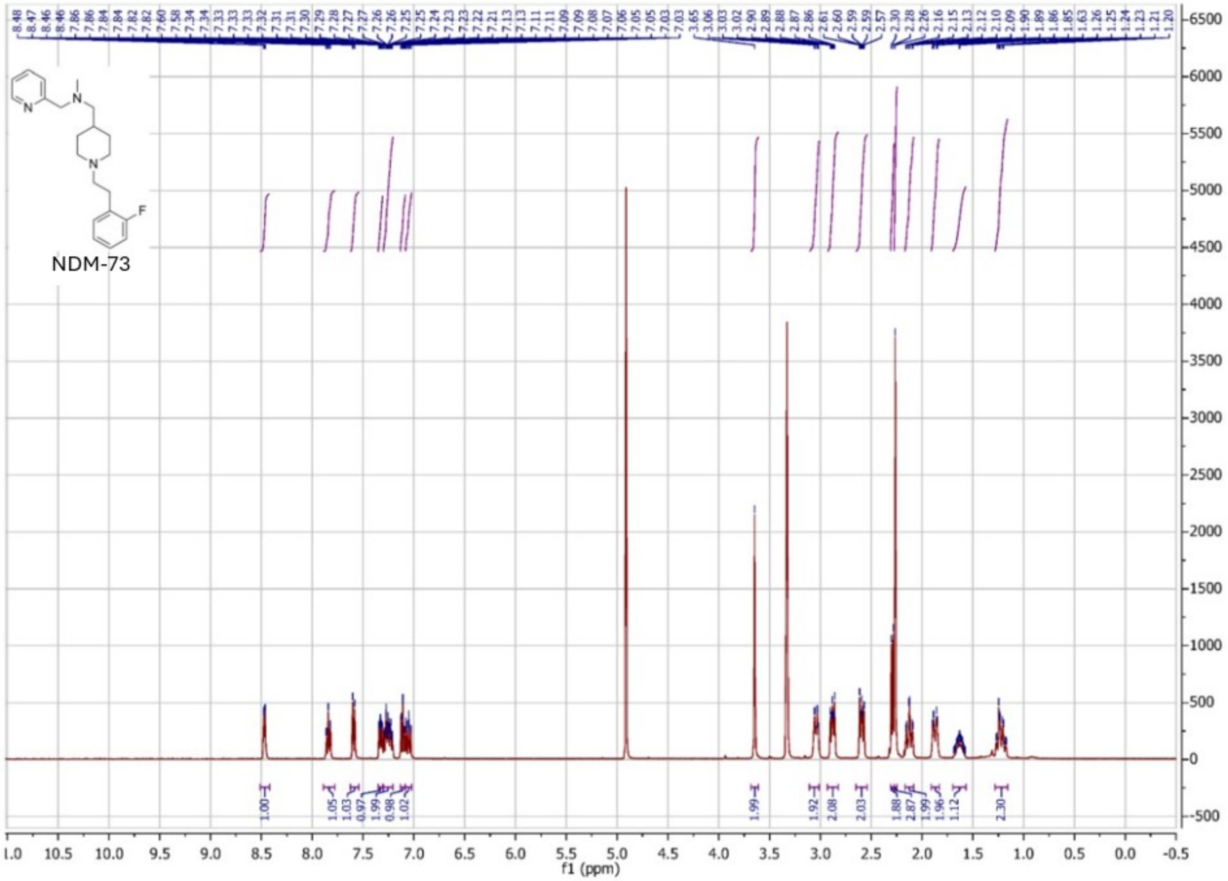

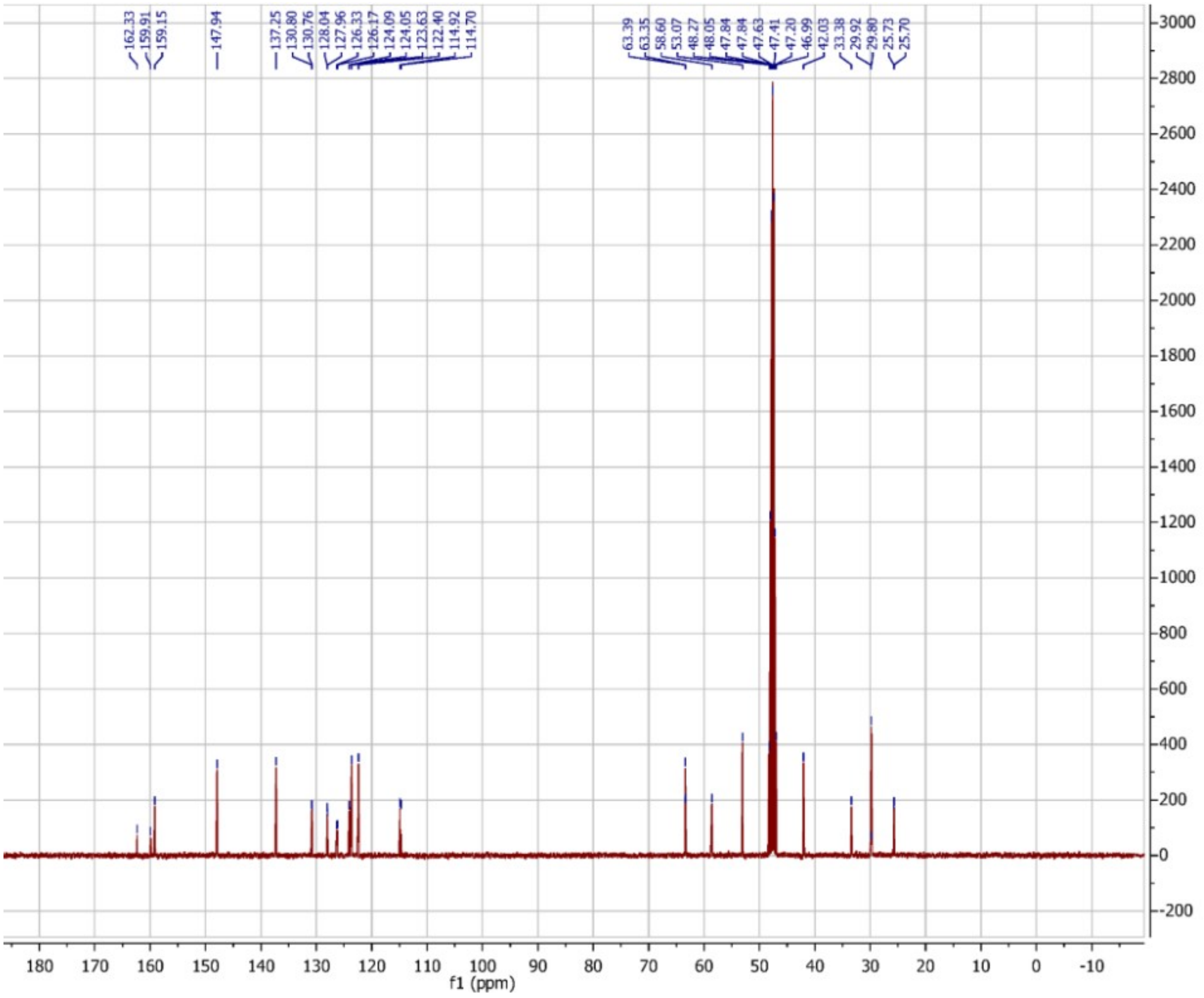
162.33
159.91
159.15
147.94
137.25
130.80
130.76
128.04
127.96
126.33
126.17
124.09
124.05
123.63
122.40
114.92
114.70
63.39
63.35
58.60
53.07
48.27
48.05
47.84
47.63
47.41
47.20
46.99
42.03
33.38
29.92
29.80
25.73
25.70
f1 (ppm)

1-(1-(2-Fluorophenethyl)piperidin-4-yl)-*N*-methyl-*N*-(pyridin-3-ylmethyl)methanamine (NDM-599):

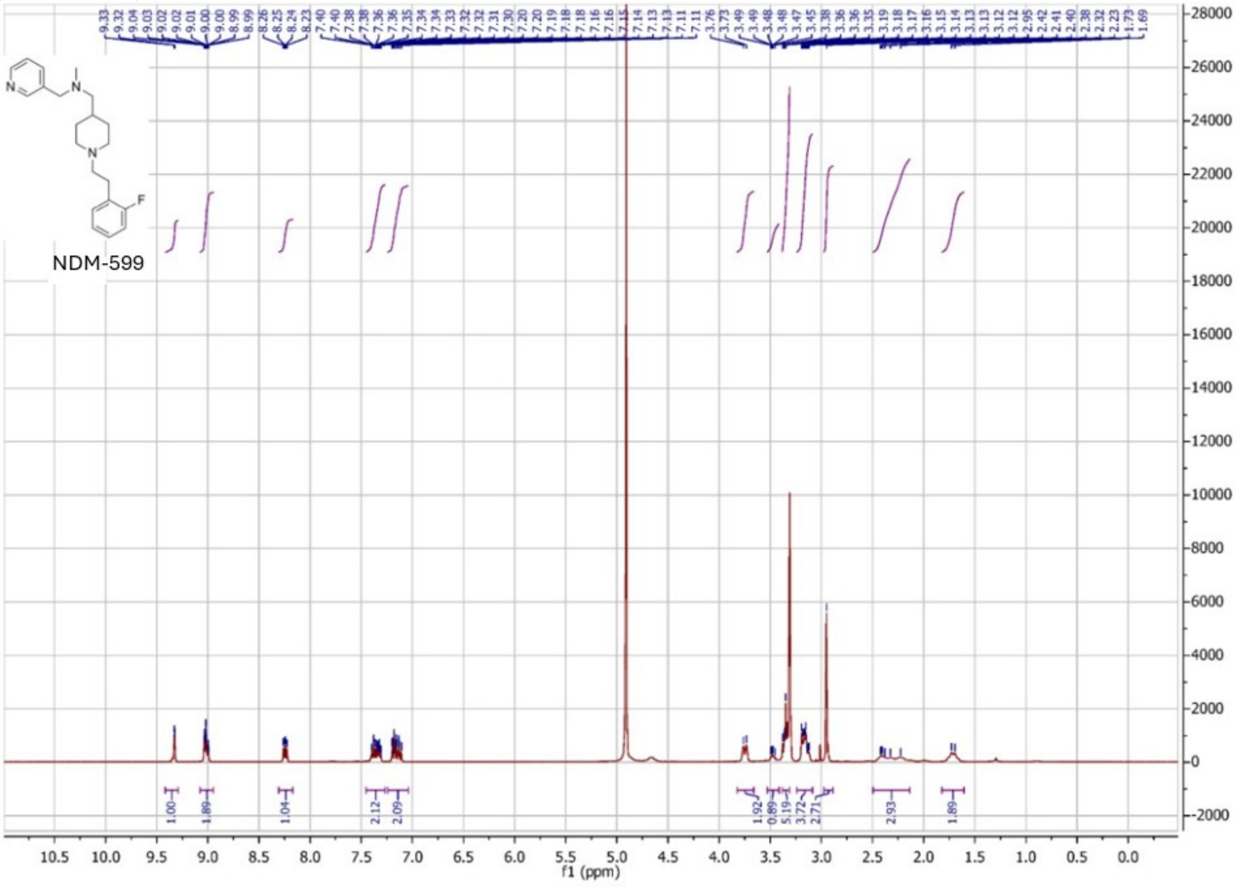

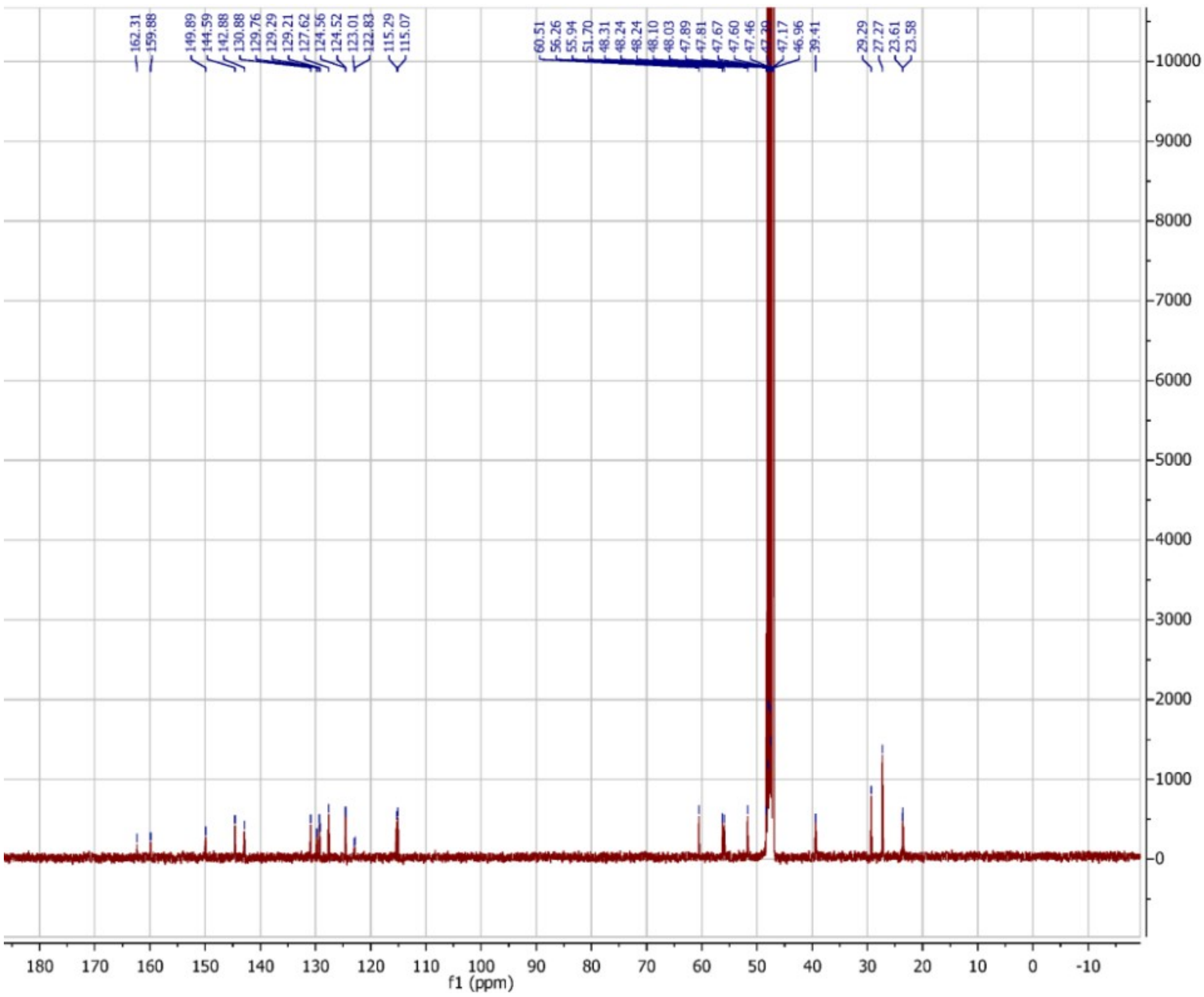
162.31
159.88
149.89
144.59
142.88
130.88
129.76
129.29
129.21
127.62
124.56
124.52
123.01
122.83
115.29
115.07
60.51
56.26
55.94
51.70
48.31
48.24
48.24
48.10
48.03
47.89
47.81
47.67
47.60
47.46
47.17
46.96
39.41
29.29
27.27
23.61
23.58
10000
9000
8000
7000
6000
5000
4000
3000
2000
1000
0
180
170
160
150
140
130
120
110
100
90
80
70
60
50
40
30
20
10
0
-10
f1 (ppm)

1-(1-(2-Fluorophenethyl)piperidin-4-yl)-*N*-methyl-*N*-(pyridin-4-ylmethyl)methanamine (NDM-72):

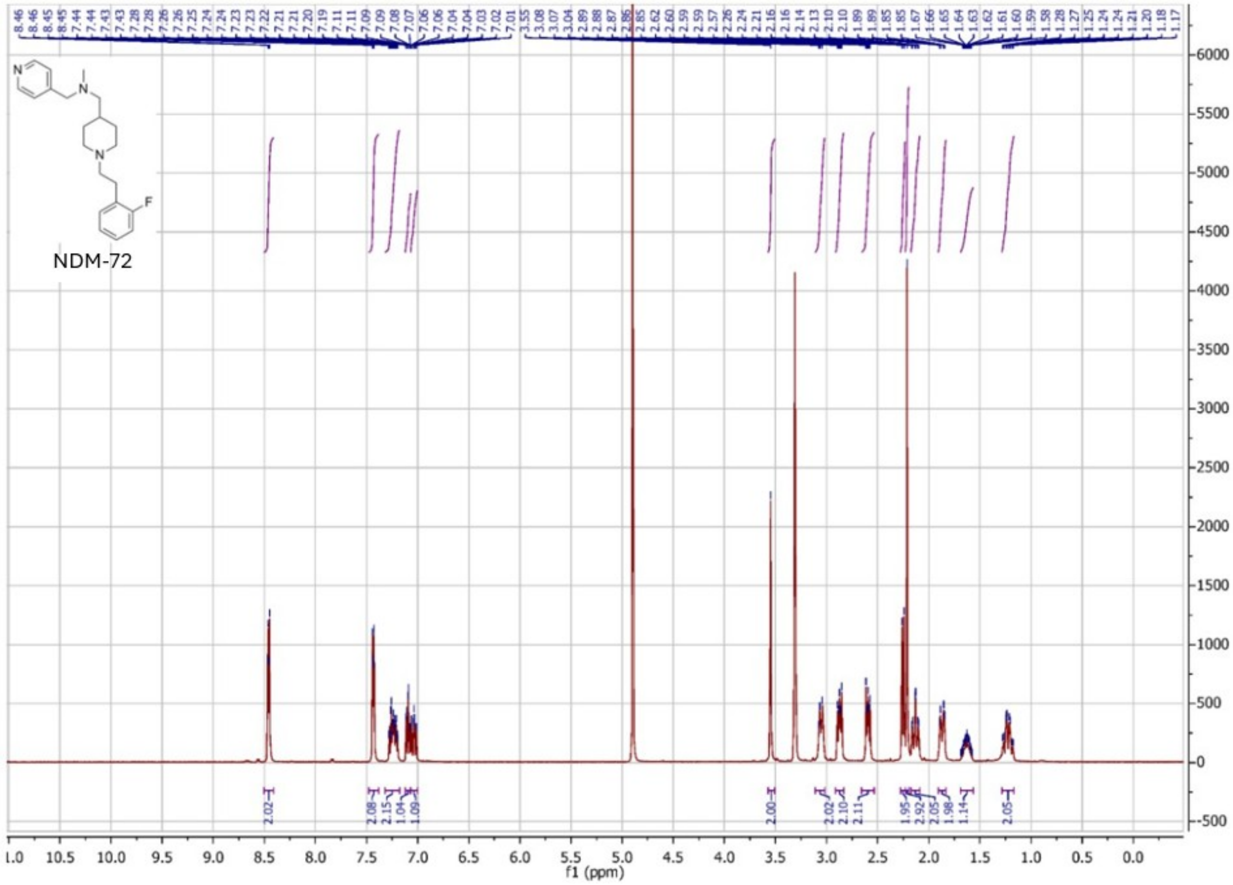

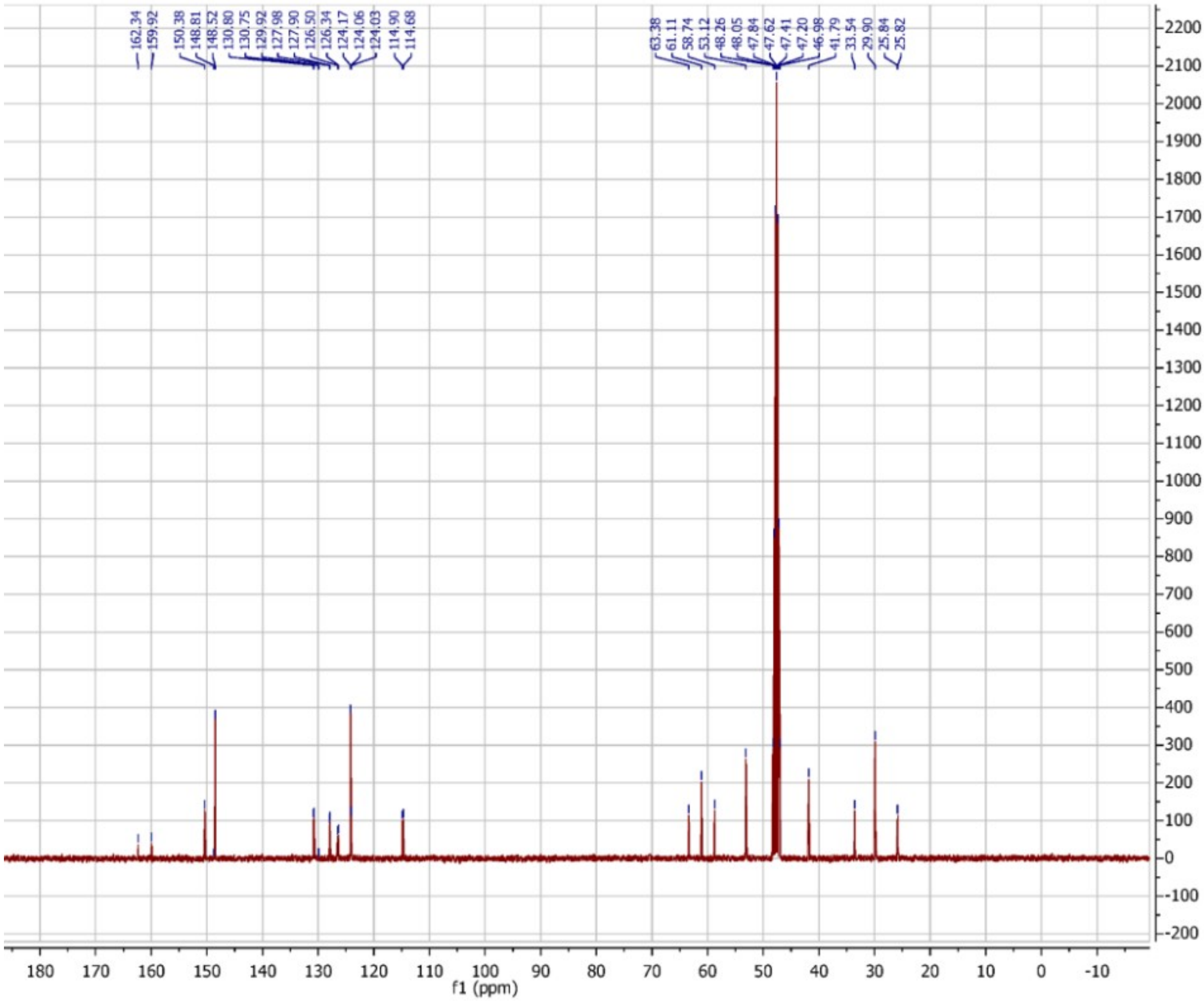
162.34
159.92
150.38
148.81
148.52
130.80
130.75
129.92
127.98
127.90
126.50
126.34
124.17
124.06
124.03
114.90
114.68
63.38
61.11
58.74
53.12
48.26
48.05
47.84
47.62
47.41
47.20
46.98
41.79
33.54
29.90
25.84
25.82
f1 (ppm)

***N*-Benzyl-1-(1-(2-fluorophenethyl)piperidin-4-yl)-*N*-methylmethanamine (NDM-43):**

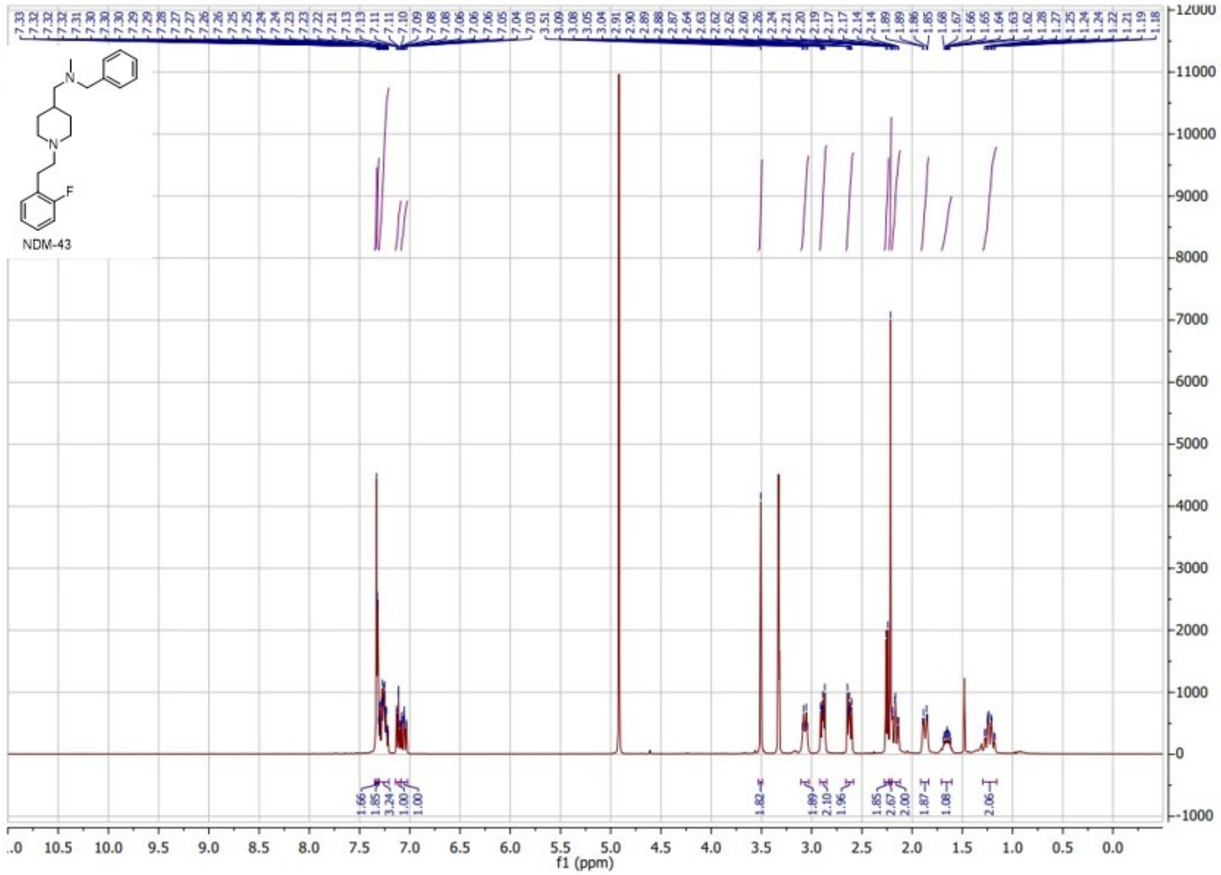

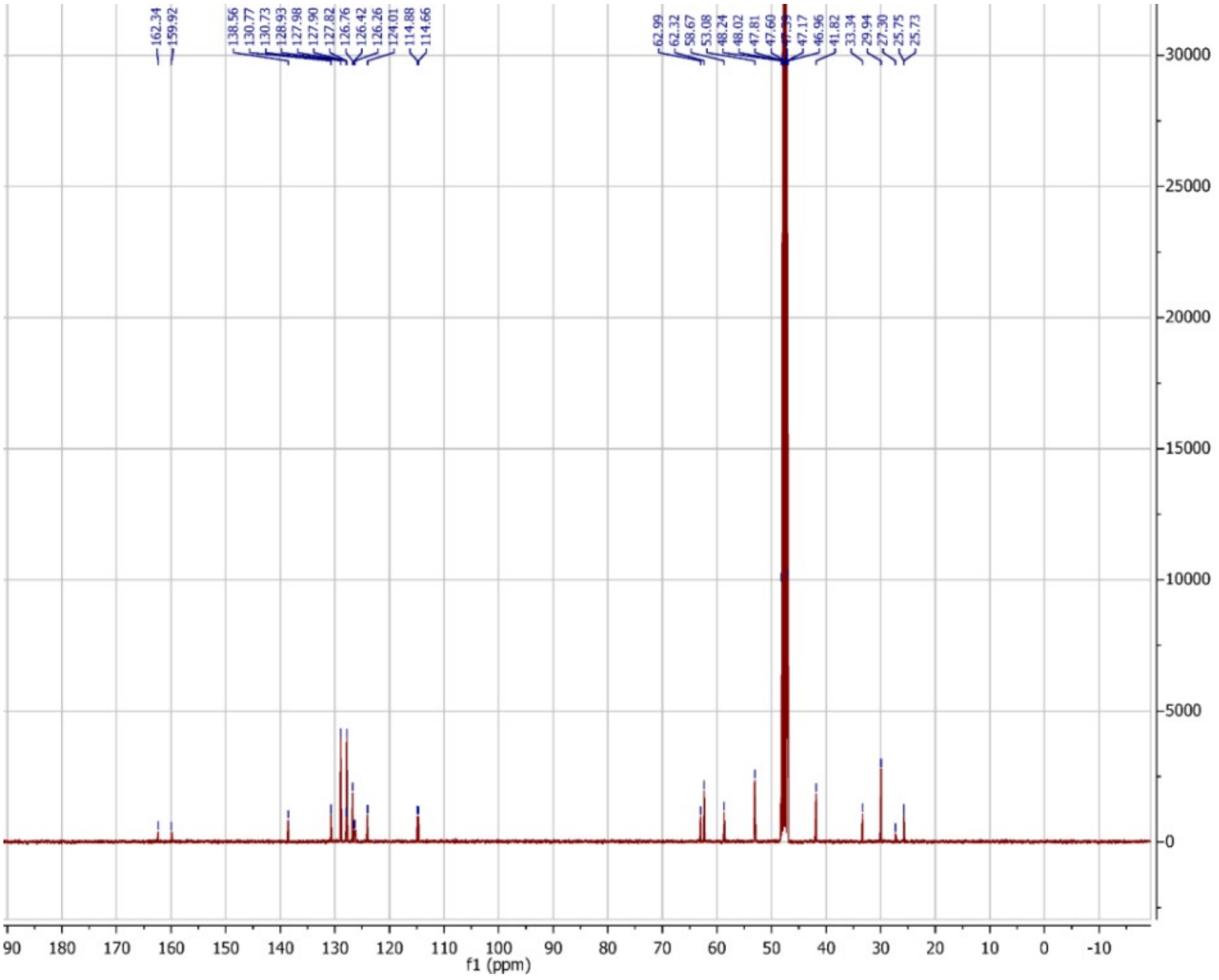
162.34
159.92
138.56
130.77
130.73
128.93
127.98
127.90
127.82
126.76
126.42
126.26
124.01
114.88
114.66
62.99
62.32
58.67
53.08
48.24
48.02
47.81
47.60
47.17
46.96
41.82
33.34
29.94
27.30
25.75
25.73
30000
25000
20000
15000
10000
5000
0
90
180
170
160
150
140
130
120
110
100
90
80
70
60
50
40
30
20
10
0
-10
f1 (ppm)

***N*-(2-Fluorobenzyl)-1-(1-(2-fluorophenethyl)piperidin-4-yl)-*N*-methylmethanamine (NDM-52):**

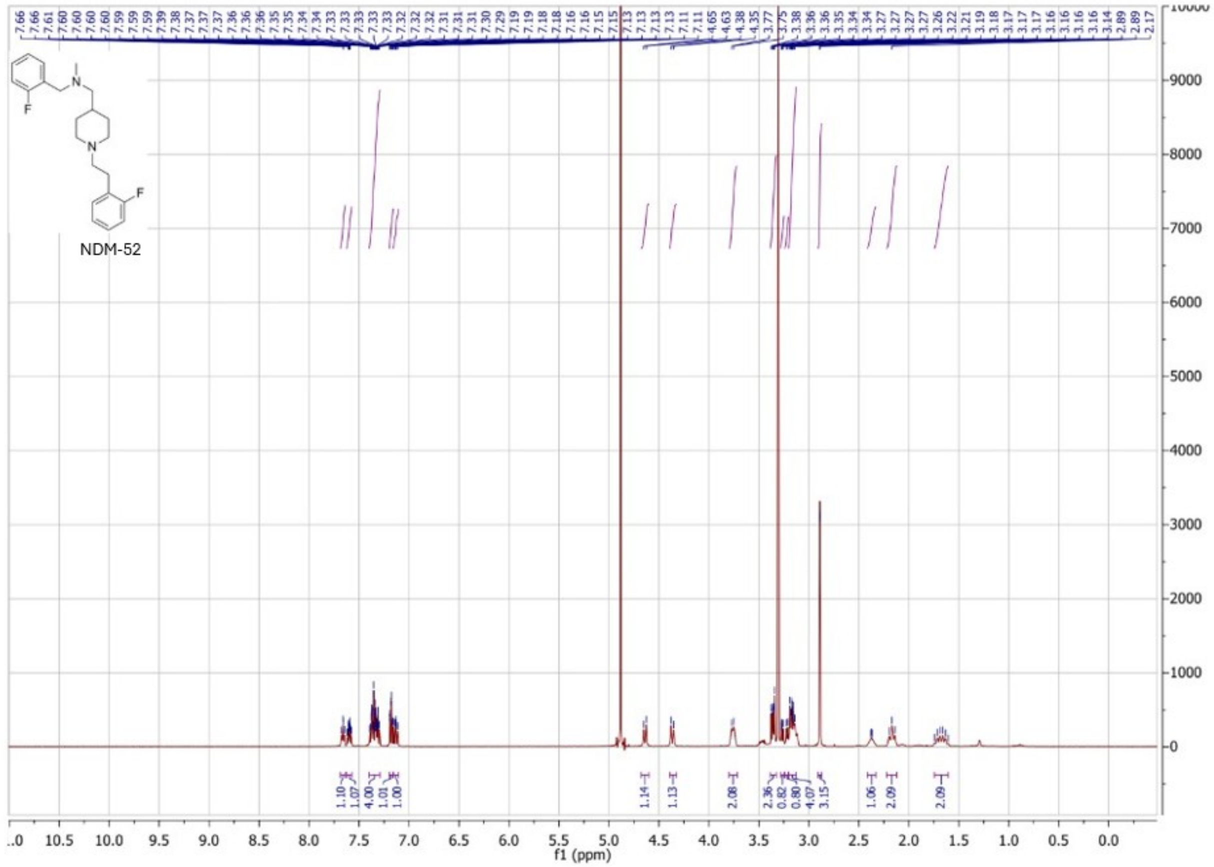

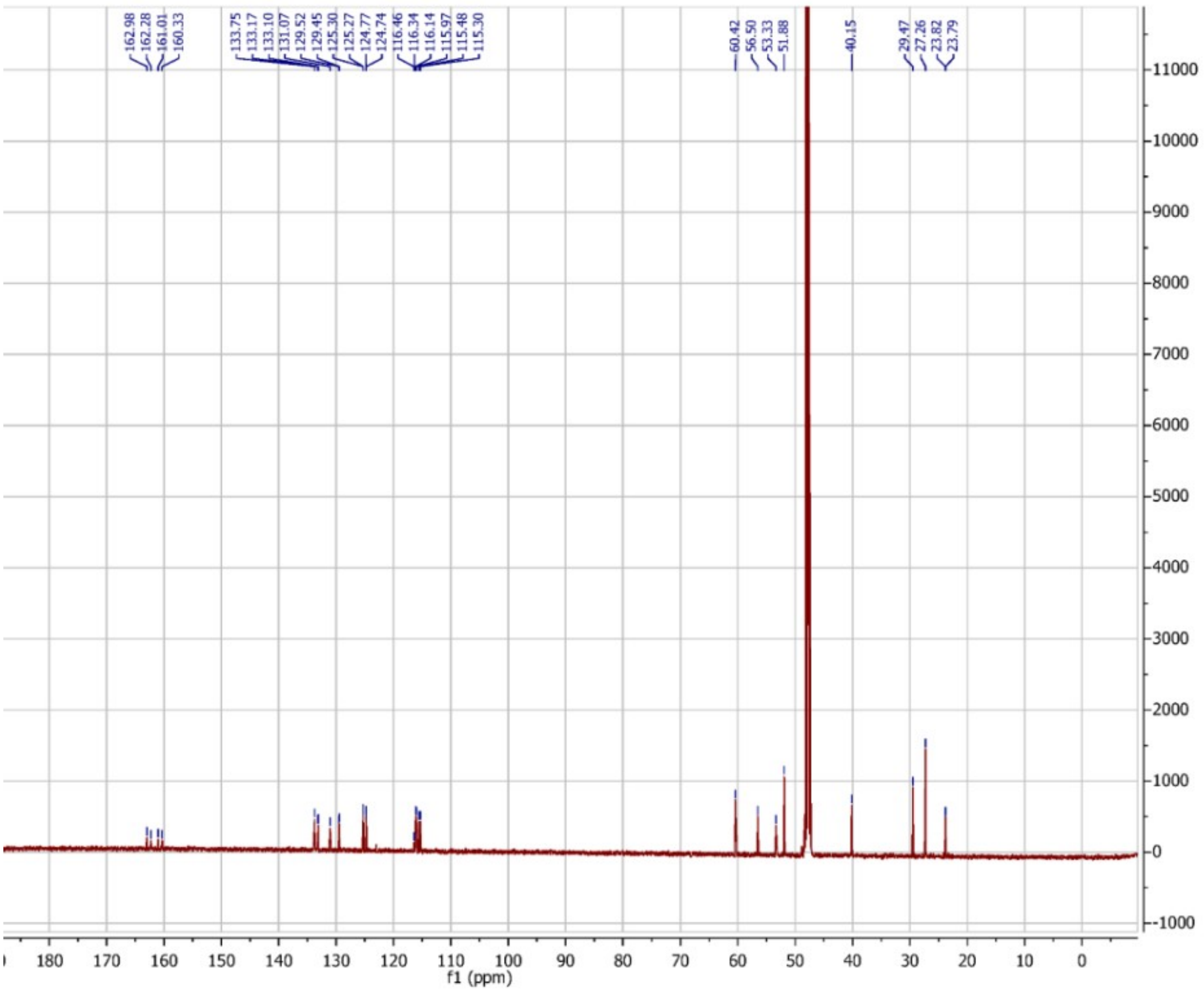

162.98
162.28
161.01
160.33
133.75
133.17
133.10
131.07
129.52
129.45
125.30
125.27
124.77
124.74
116.46
116.34
116.14
115.97
115.48
115.30
60.42
56.50
53.33
51.88
40.15
29.47
27.26
23.82
23.79
f1 (ppm)

***N*-(2-Chlorobenzyl)-1-(1-(2-fluorophenethyl)piperidin-4-yl)-*N*-methylmethanamine (NDM-51):**

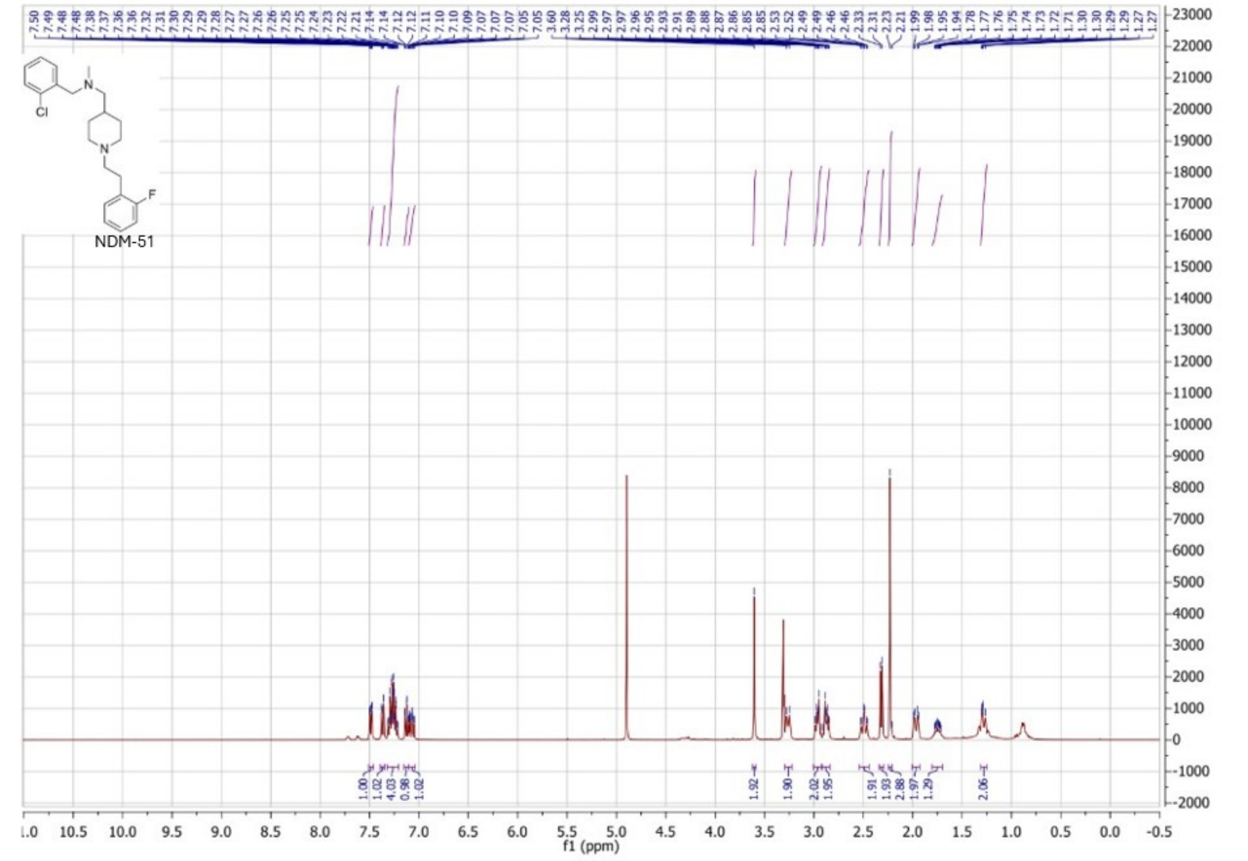

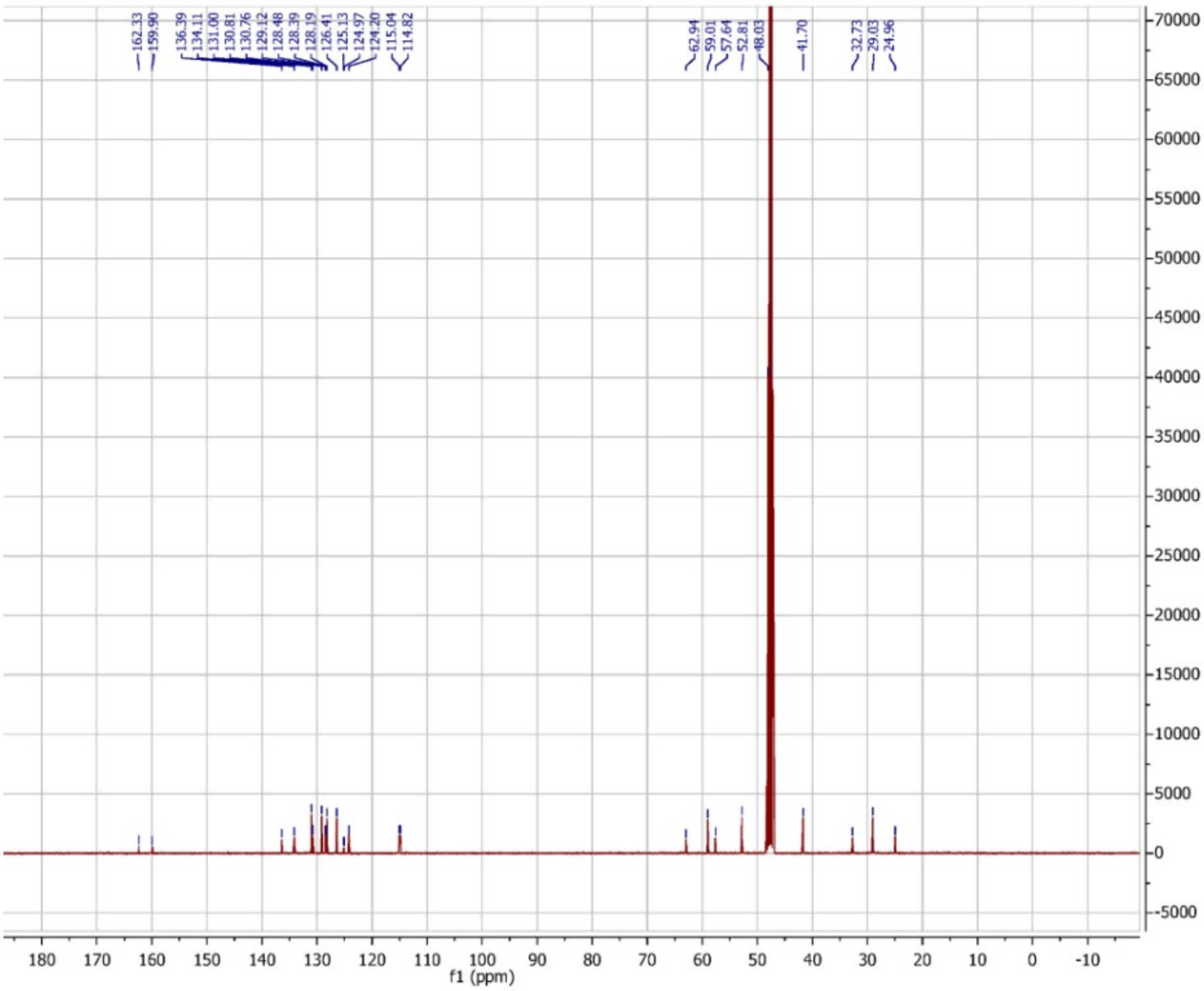

162.33
159.90
136.39
134.11
131.00
130.81
130.76
129.12
128.48
128.39
128.19
126.41
125.13
124.97
124.20
115.04
114.82
62.94
59.01
57.64
52.81
48.03
41.70
32.73
29.03
24.96
f1 (ppm)

***N*-(2-Vromobenzyl)-1-(1-(2-fluorophenethyl)piperidin-4-yl)-*N*-methylmethanamine (NDM-601):**

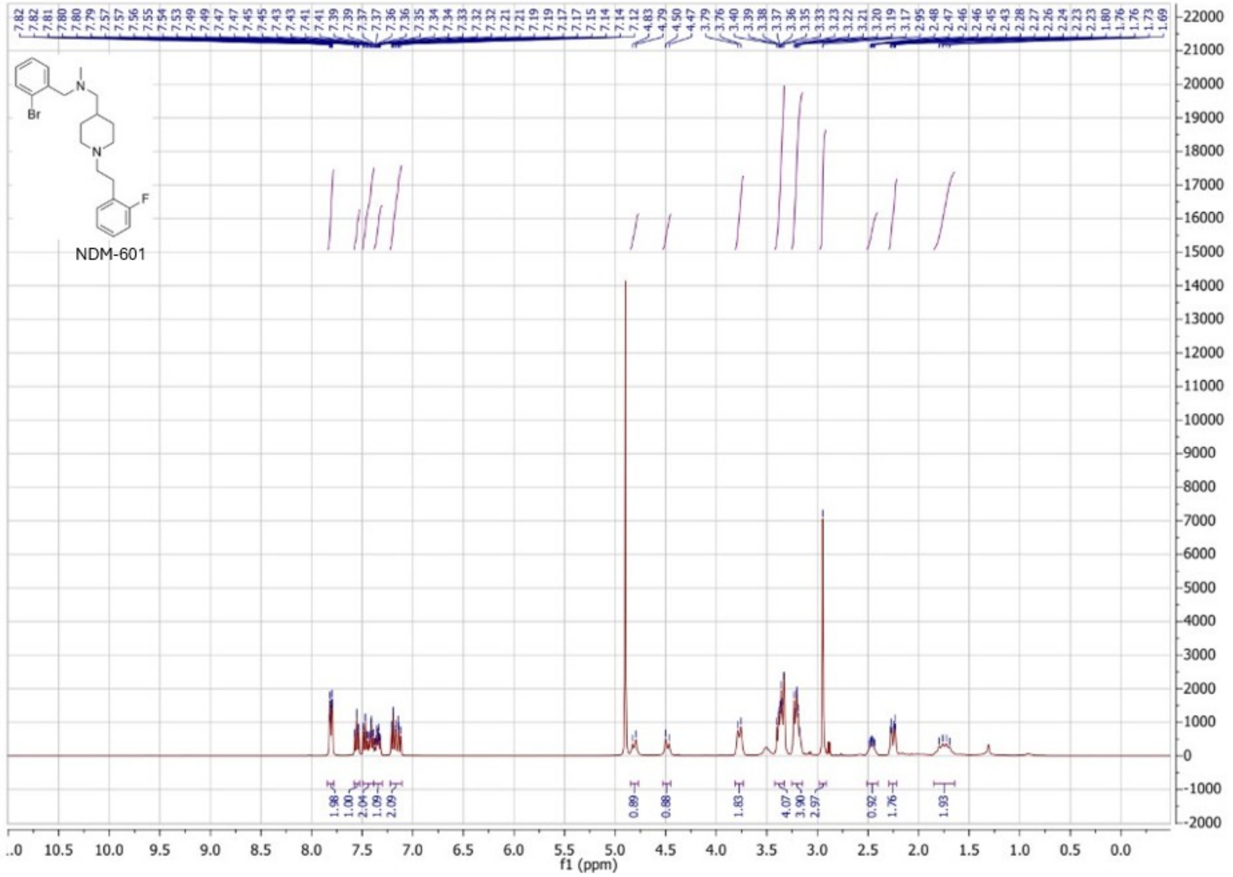

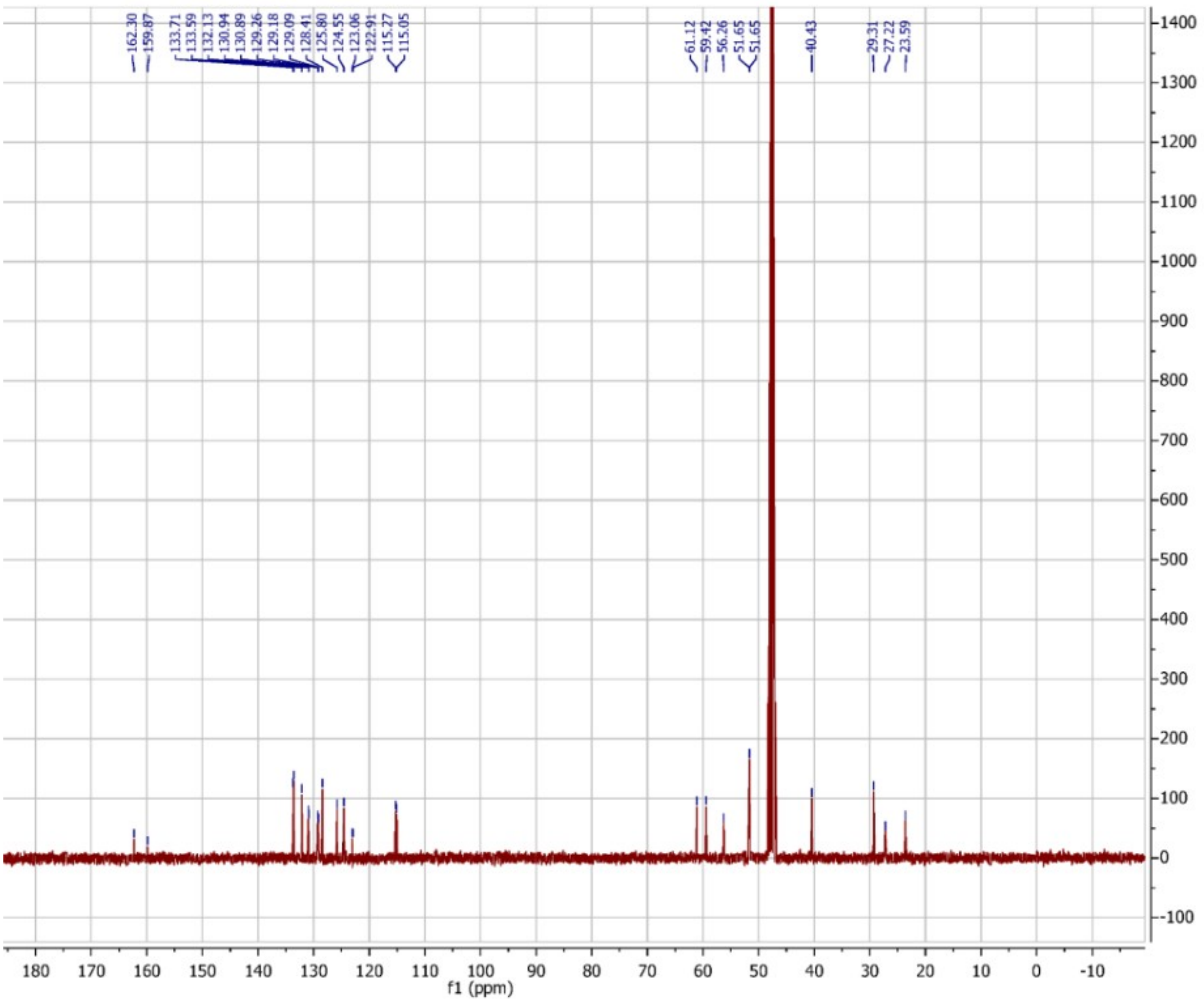
162.30
159.87
133.71
133.59
132.13
130.94
130.89
129.26
129.18
129.09
128.41
125.80
124.55
123.06
122.91
115.27
115.05
61.12
59.42
56.26
51.65
51.65
40.43
29.31
27.22
23.59
f1 (ppm)

1-(1-(2-Fluorophenethyl)piperidin-4-yl)-*N*-(2-iodobenzyl)-*N*-methylmethanamine (NDM-603):

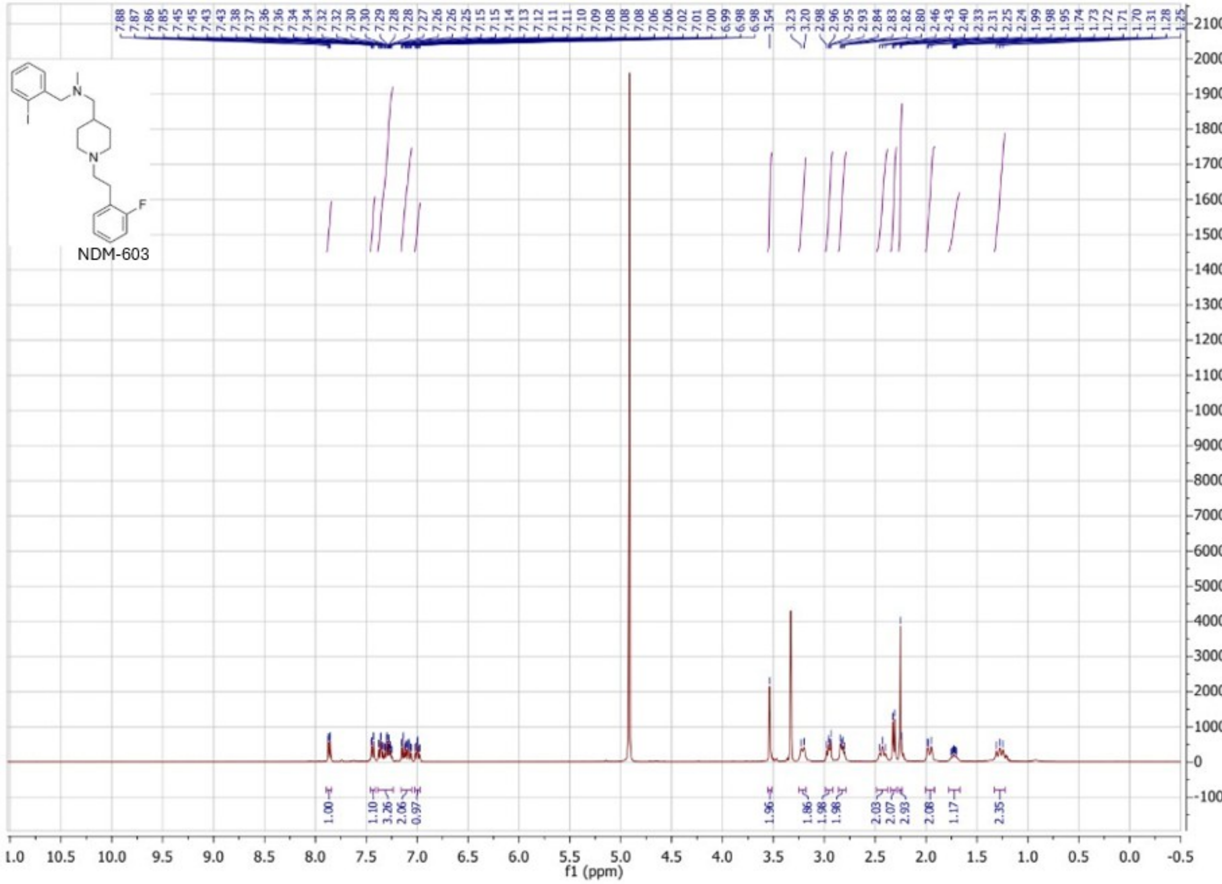

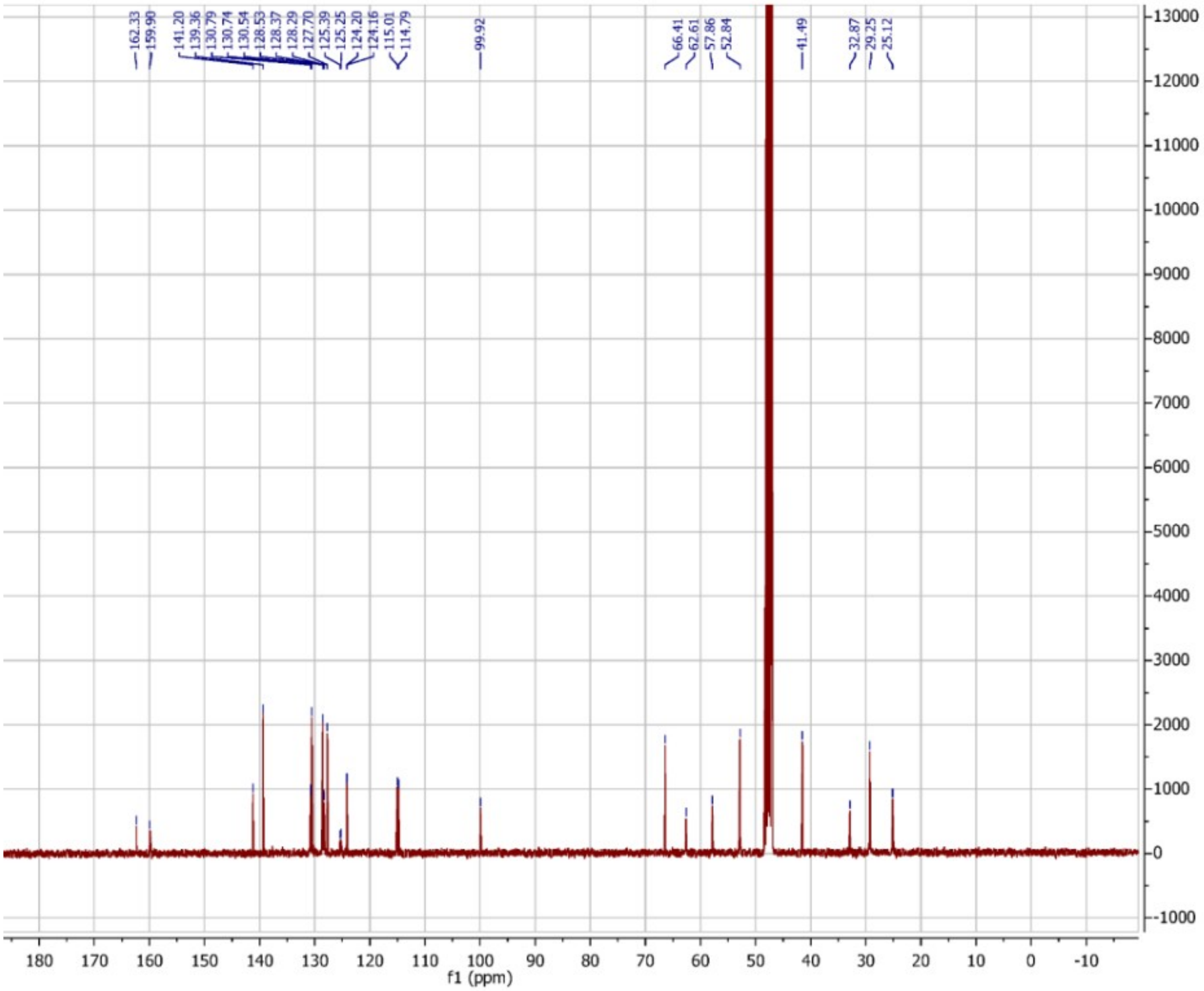
162.33
159.90
141.20
139.36
130.79
130.74
130.54
128.53
128.37
128.29
127.70
125.39
125.25
124.20
124.16
115.01
114.79
99.92
66.41
62.61
57.86
52.84
41.49
32.87
29.25
25.12
f1 (ppm)

1-(1-(2-Fluorophenethyl)piperidin-4-yl)-*N*-methyl-*N*-(2-methylbenzyl)methanamine (NDM-637):

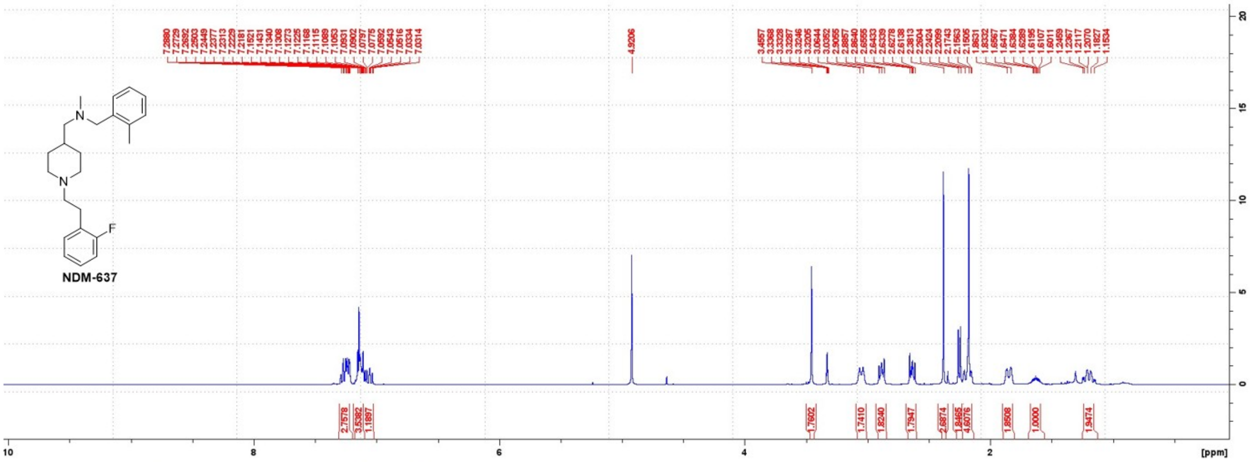

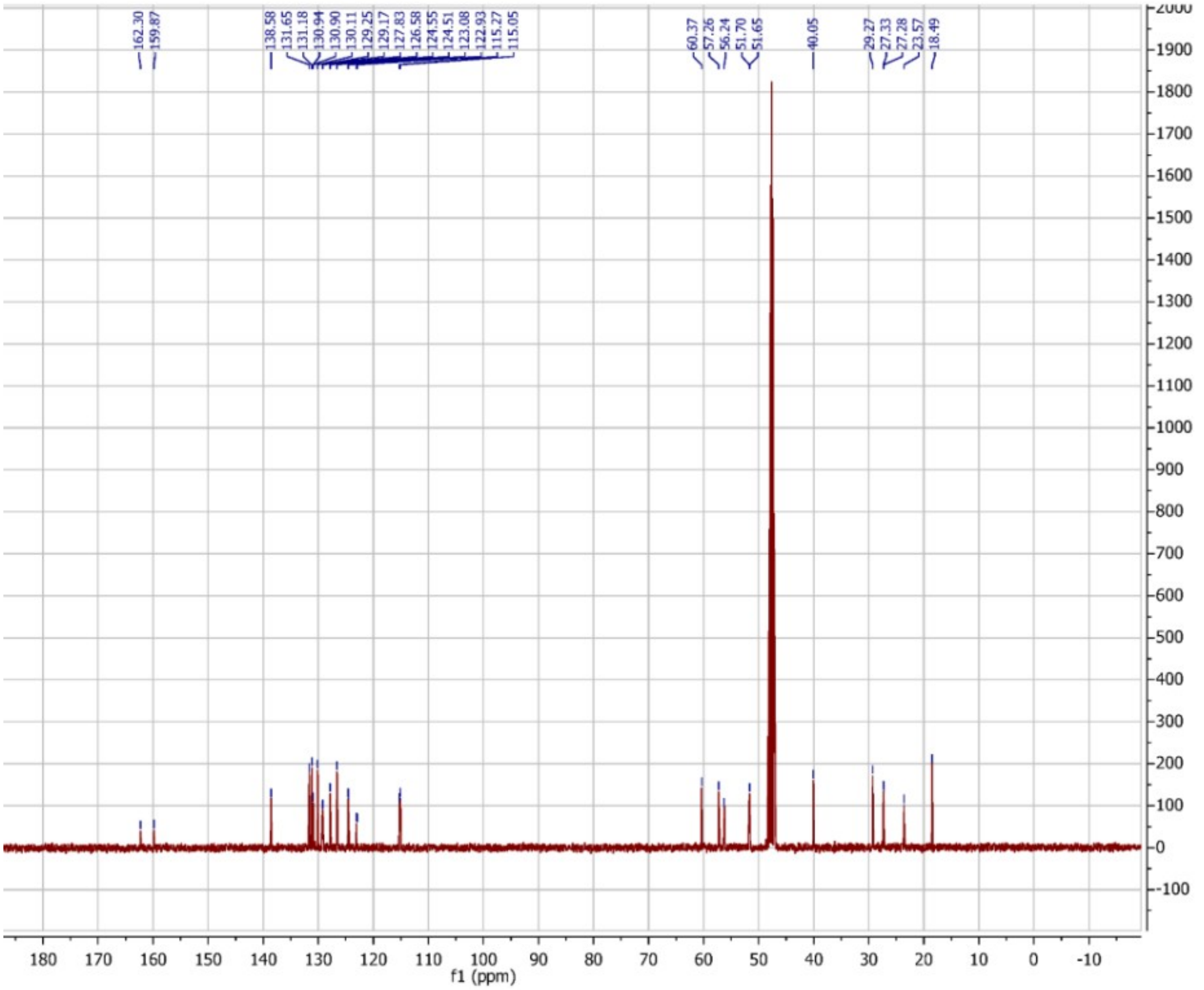

***N*-(3-Fluorobenzyl)-1-(1-(2-fluorophenethyl)piperidin-4-yl)-*N*-methylmethanamine (NDM-556):**

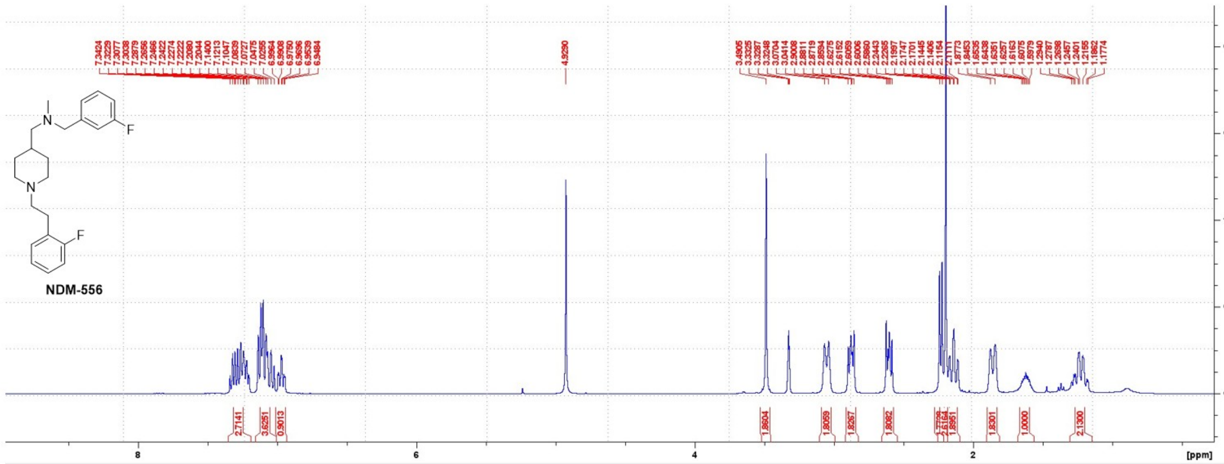

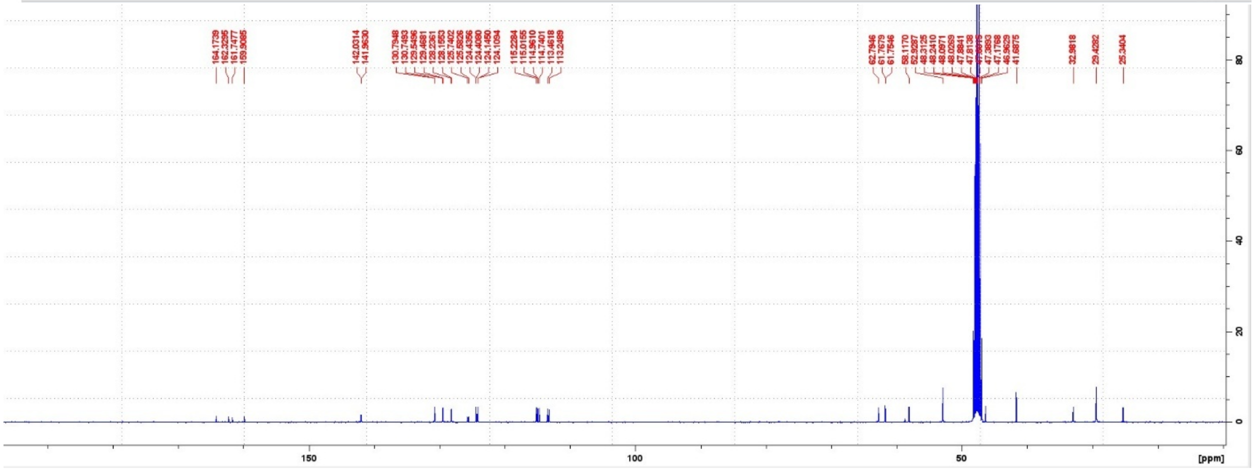

***N*-(3-Chlorobenzyl)-1-(1-(2-fluorophenethyl)piperidin-4-yl)-*N*-methylmethanamine (NDM-49):**

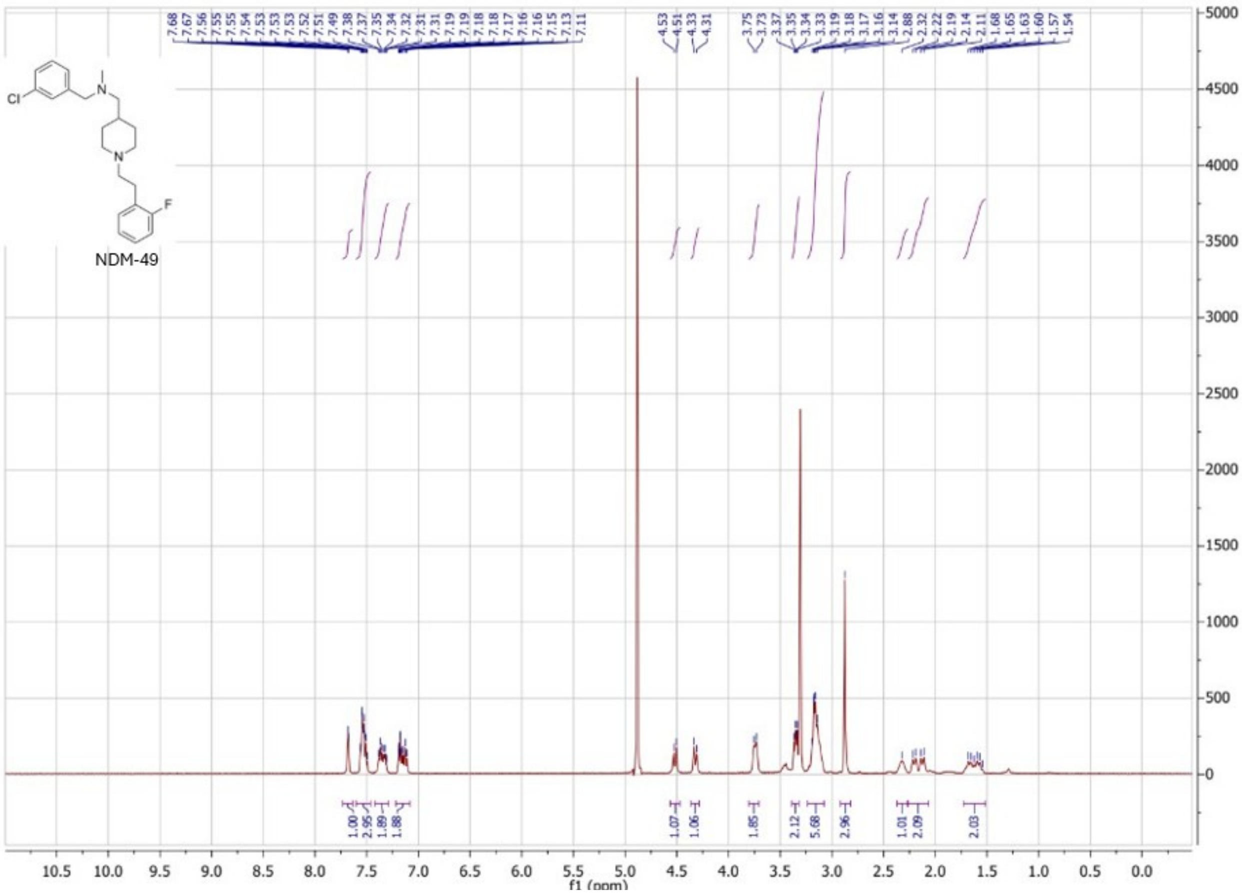
NDM-49
f1 (ppm)

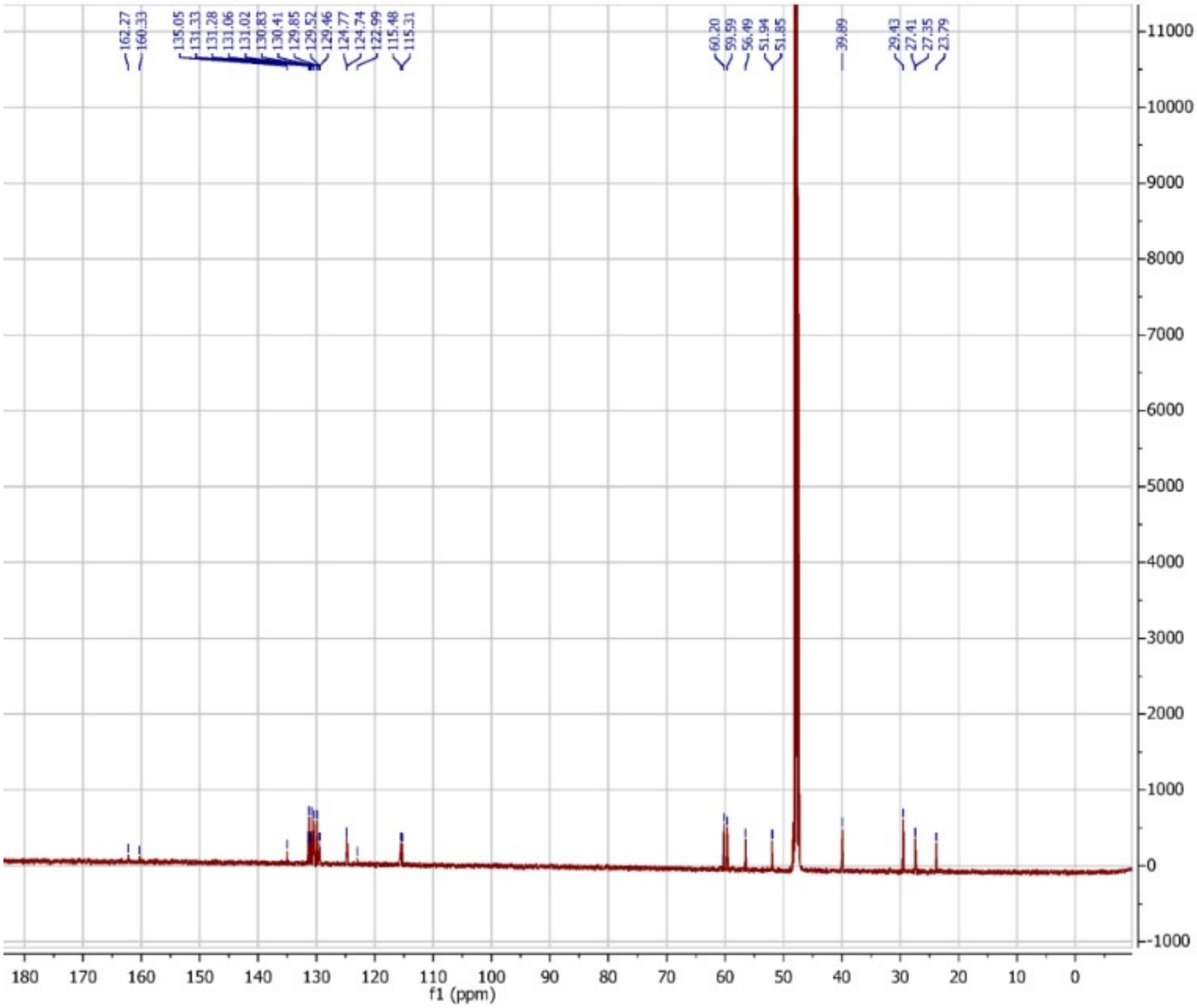

***N*-(3-Bromobenzyl)-1-(1-(2-fluorophenethyl)piperidin-4-yl)-*N*-methylmethanamine (NDM-557):**

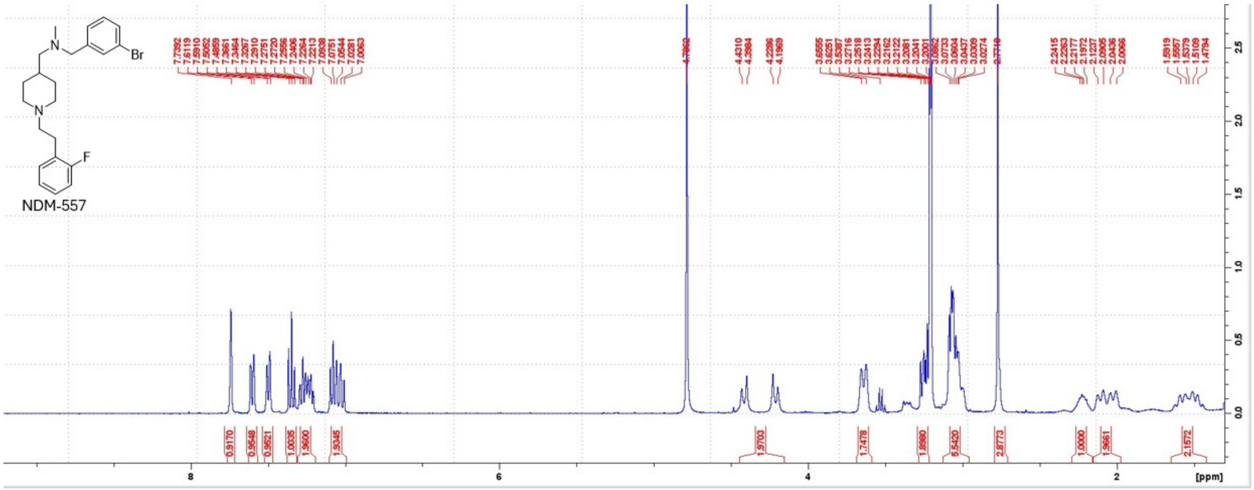

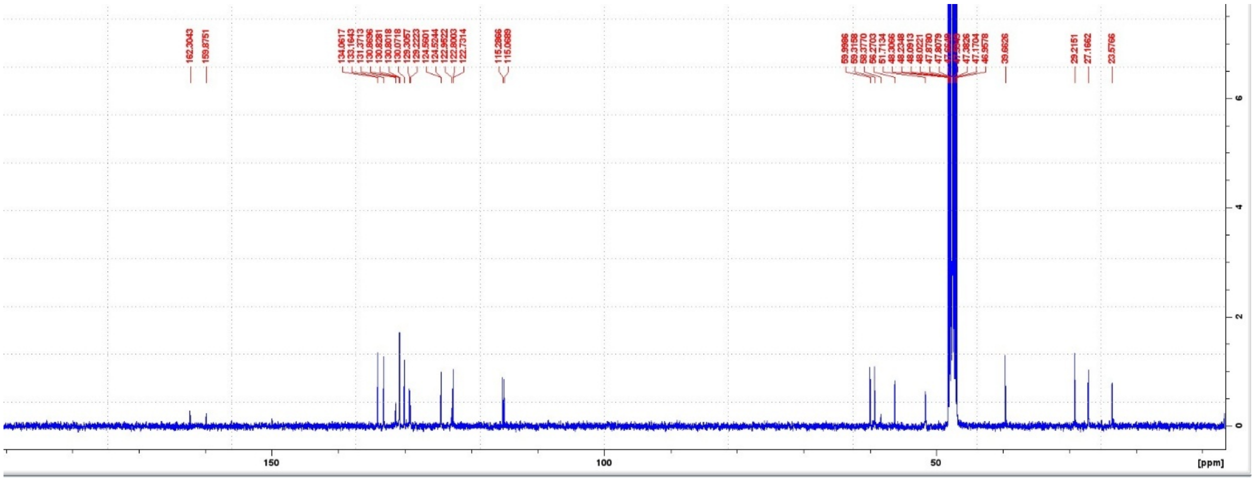

1-(1-(2-Fluorophenethyl)piperidin-4-yl)-*N*-(3-iodobenzyl)-*N*-methylmethanamine (NDM-32):

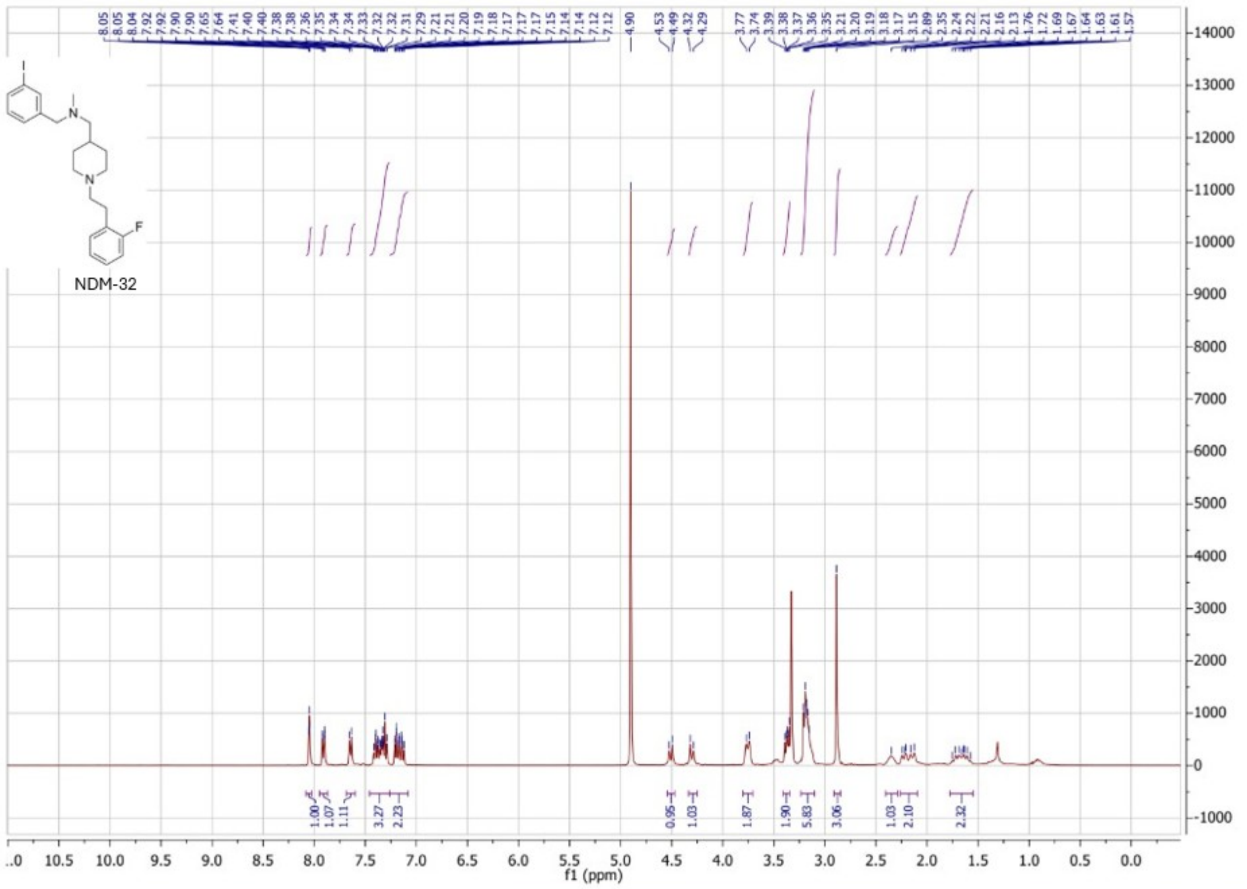

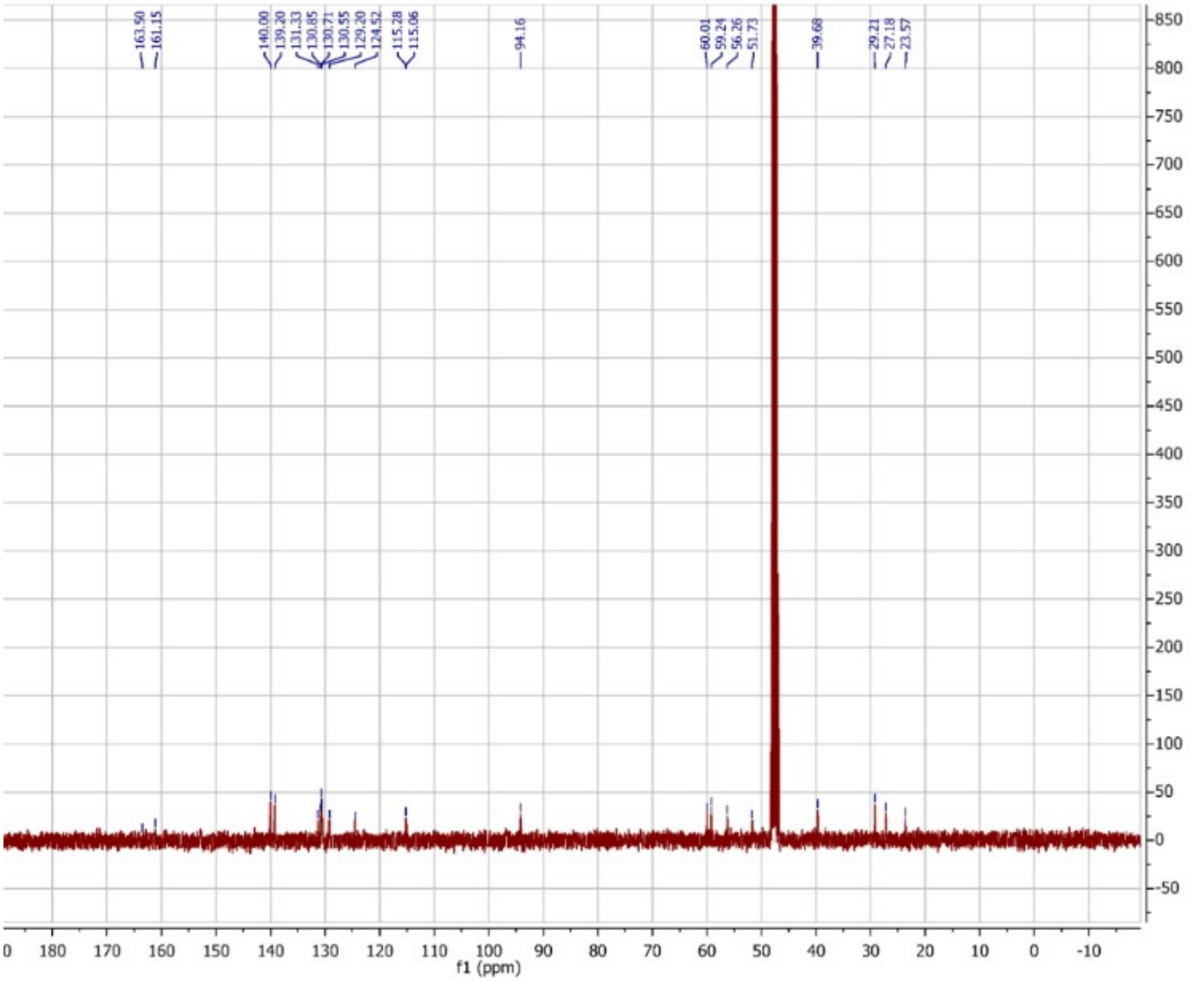

1-(1-(2-Fluorophenethyl)piperidin-4-yl)-*N*-methyl-*N*-(3-methylbenzyl)methanamine (NDM-561):

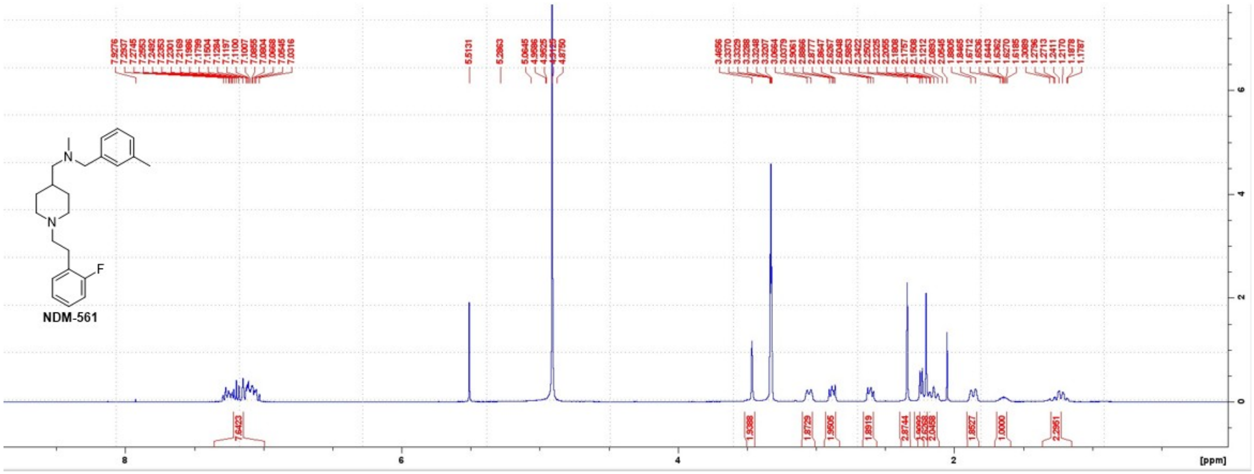

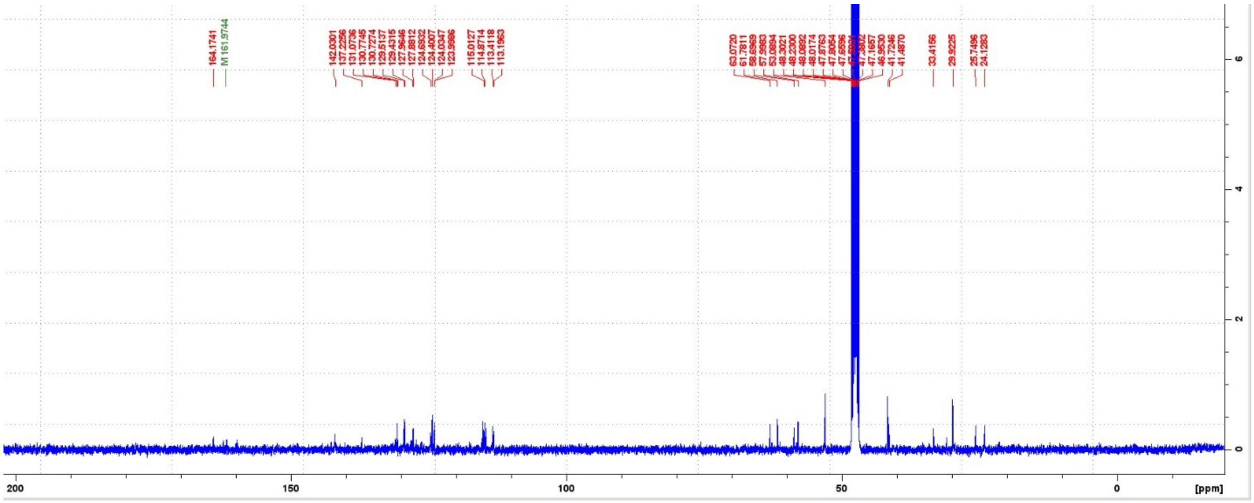

***N*-(4-Fluorobenzyl)-1-(1-(2-fluorophenethyl)piperidin-4-yl)-*N*-methylmethanamine (NDM-48):**

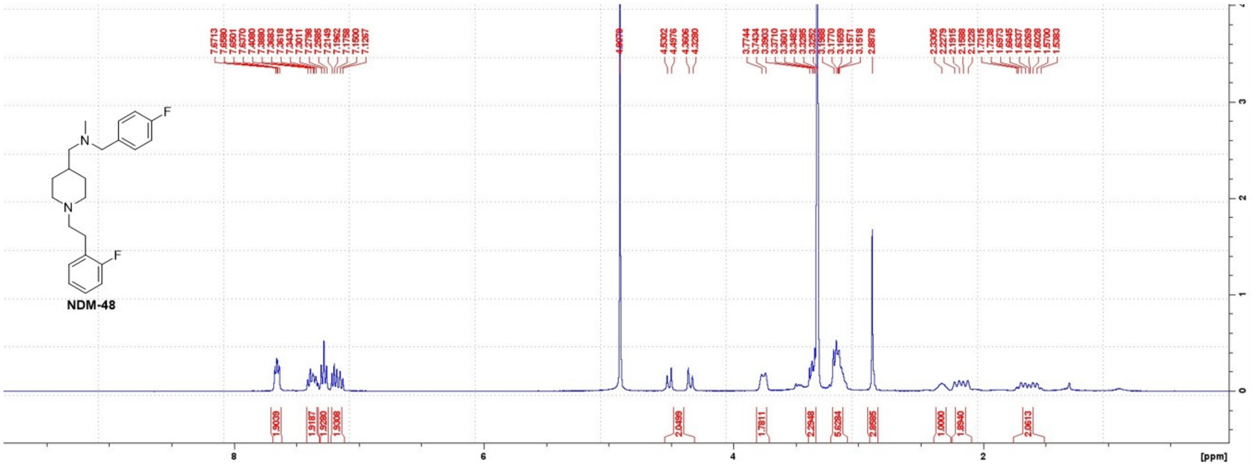

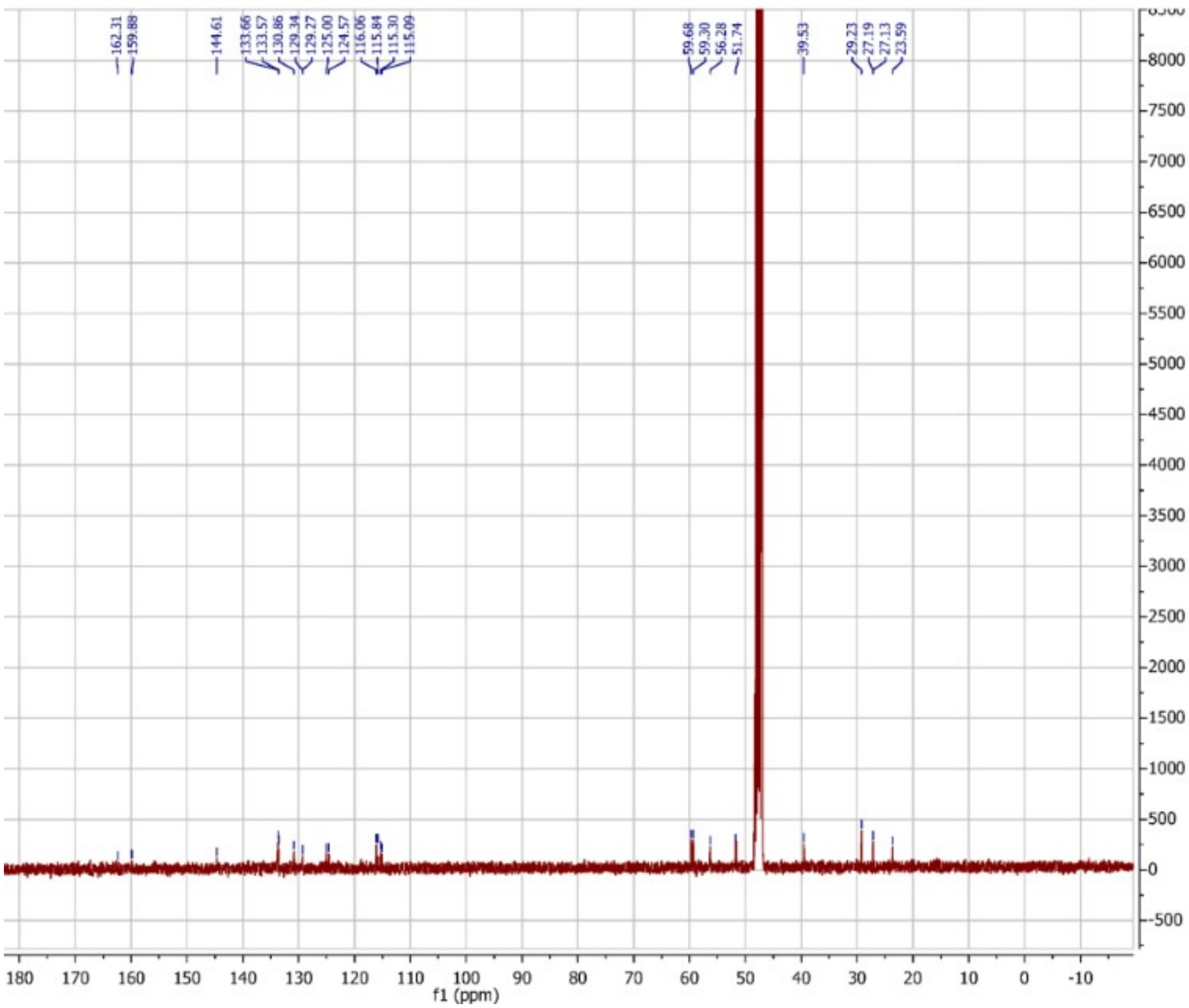
162.31
159.88
144.61
133.66
133.57
130.86
129.34
129.27
125.00
124.57
116.06
115.84
115.30
115.09
59.68
59.30
56.28
51.74
39.53
29.23
27.19
27.13
23.59
f1 (ppm)

***N*-(4-Chlorobenzyl)-1-(1-(2-fluorophenethyl)piperidin-4-yl)-*N*-methylmethanamine (NDM-47):**

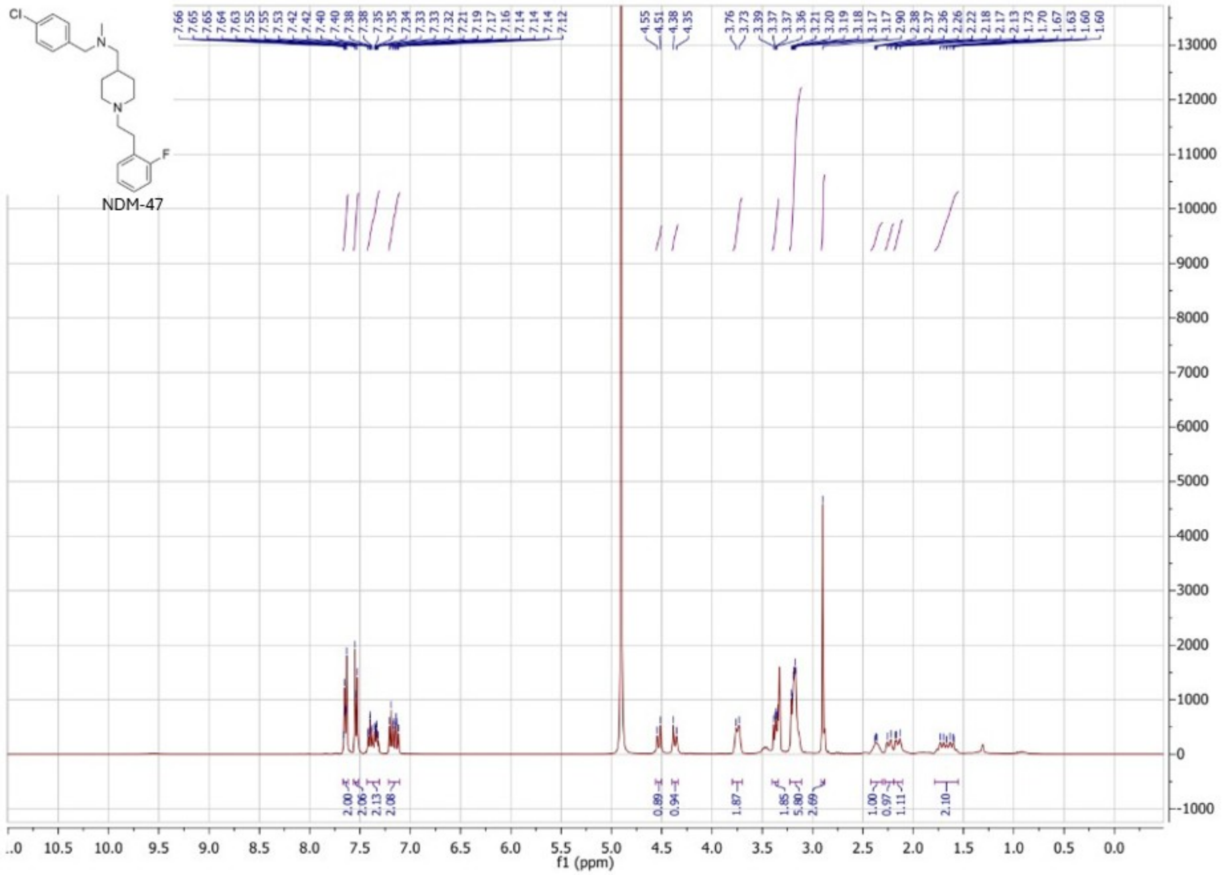

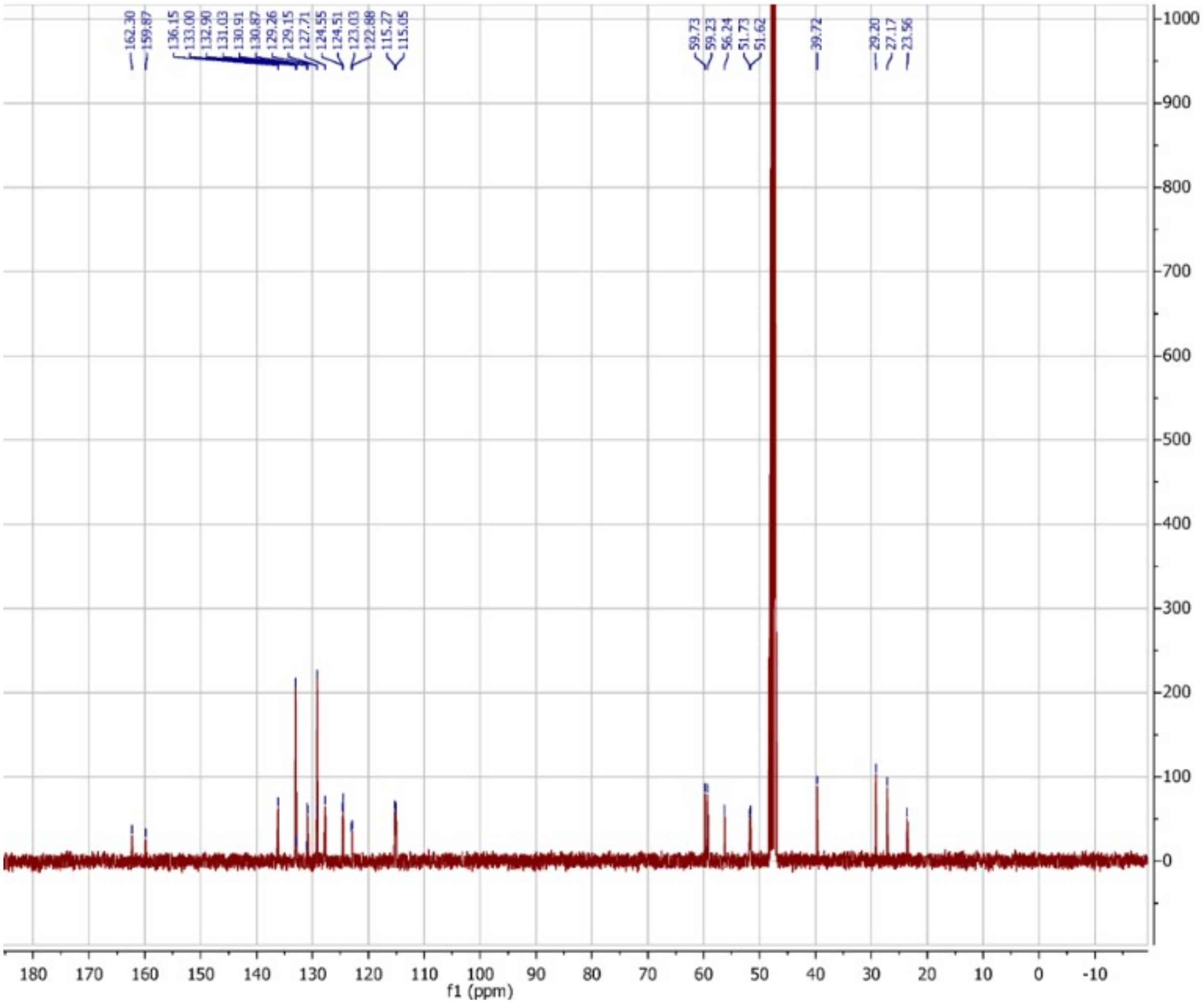
162.30
159.87
136.15
133.00
132.90
131.03
130.91
130.87
129.26
129.15
127.71
124.55
124.51
123.03
122.88
115.27
115.05
59.73
59.23
56.24
51.73
51.62
39.72
29.20
27.17
23.56
1000
900
800
700
600
500
400
300
200
100
0
180 170 160 150 140 130 120 110 100 90 80 70 60 50 40 30 20 10 0 -10
f1 (ppm)

***N*-(4-Bromobenzyl)-1-(1-(2-fluorophenethyl)piperidin-4-yl)-*N*-methylmethanamine (NDM-558):**

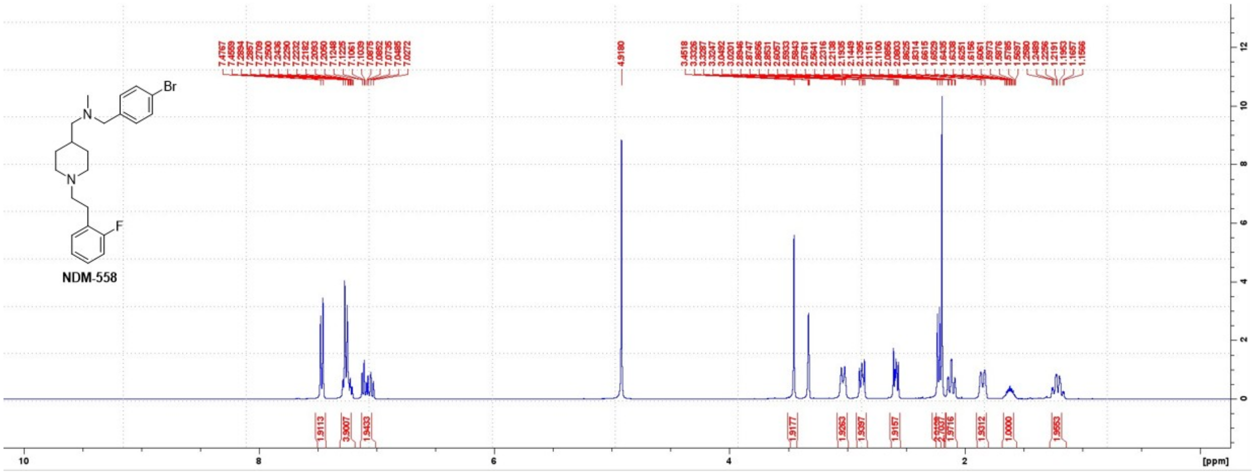

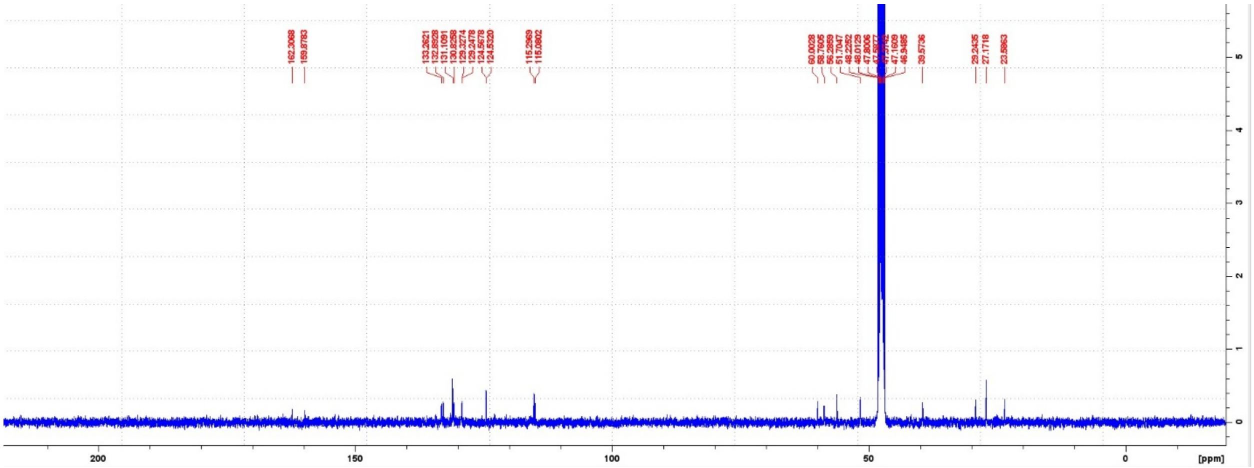
162.3068
159.8783
133.2621
132.8928
131.1091
130.8258
129.3274
129.2478
124.5678
124.5320
115.2969
115.0802
60.0028
58.7605
56.2859
51.7047
48.2252
48.0129
47.8006
47.5877
47.3742
47.1609
46.9485
39.5736
29.2435
27.1718
23.5863
200
150
100
50
0
[ppm]
5
4
3
2
1
0

1-(1-(2-Fluorophenethyl)piperidin-4-yl)-*N*-(4-iodobenzyl)-*N*-methylmethanamine (NDM-563):

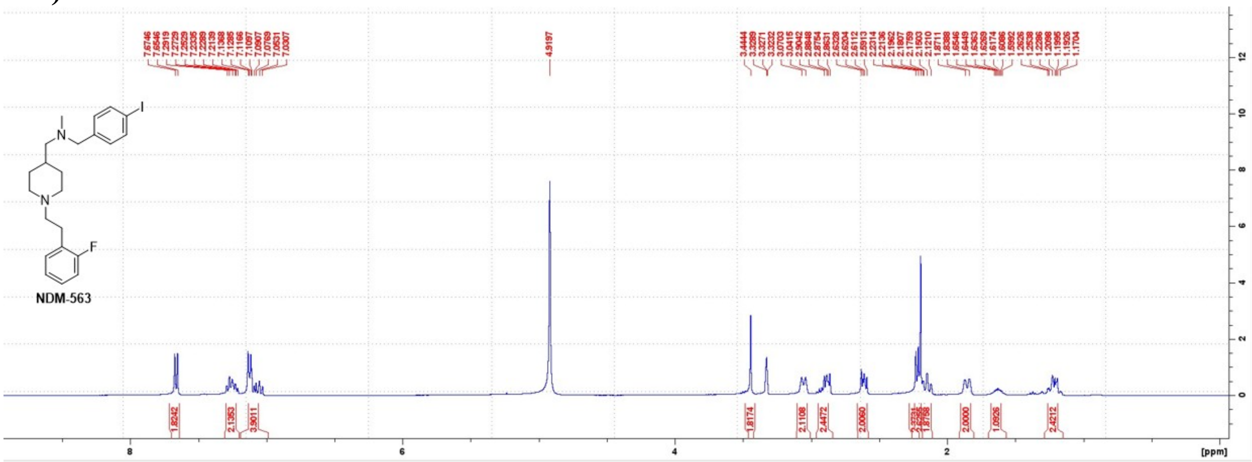

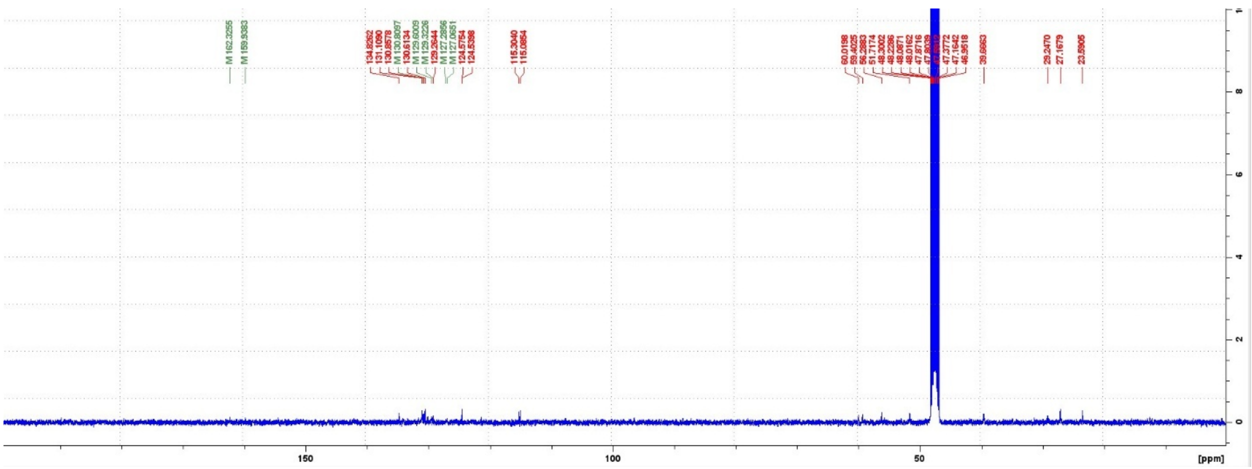

1-(1-(2-Fluorophenethyl)piperidin-4-yl)-*N*-methyl-*N*-(4-methylbenzyl)methanamine (NDM-562):

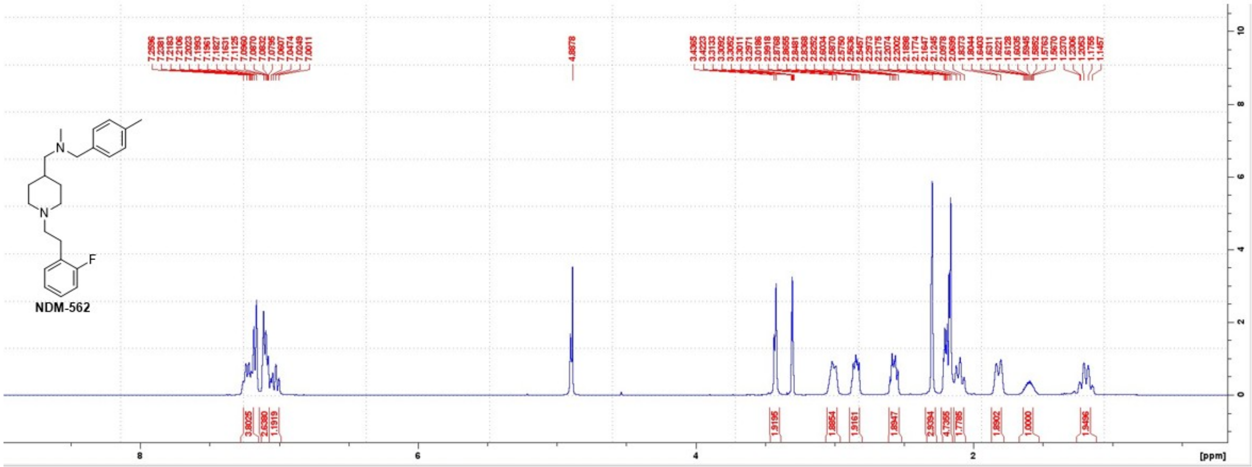

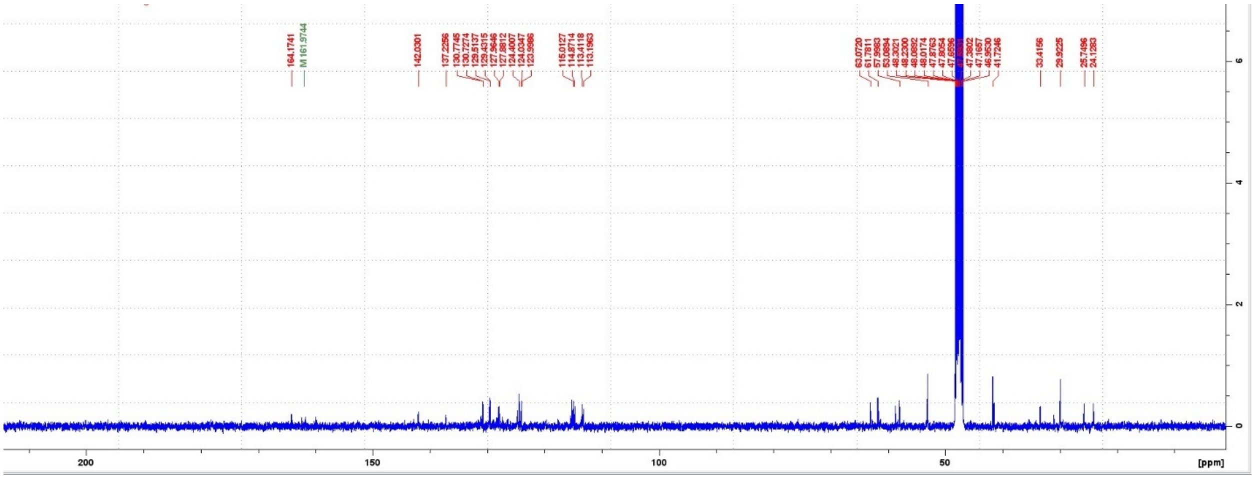

164.1741
M 161.9744
142.0301
137.2256
130.7745
130.7274
129.5137
129.4315
127.9646
127.8812
124.4007
124.0347
123.9986
115.0127
114.8714
113.4118
113.1963
63.0720
61.7811
57.9983
53.0694
48.3021
48.2300
48.0892
48.0174
47.8763
47.8054
47.6596
47.3802
47.1657
46.9530
41.7246
33.4156
29.9225
25.7496
24.1283
200
150
100
50
[ppm]
6
4
2
0

***N*-(2,3-Dichlorobenzyl)-1-(1-(2-fluorophenethyl)piperidin-4-yl)-*N*-methylmethanamine (NDM-45):**

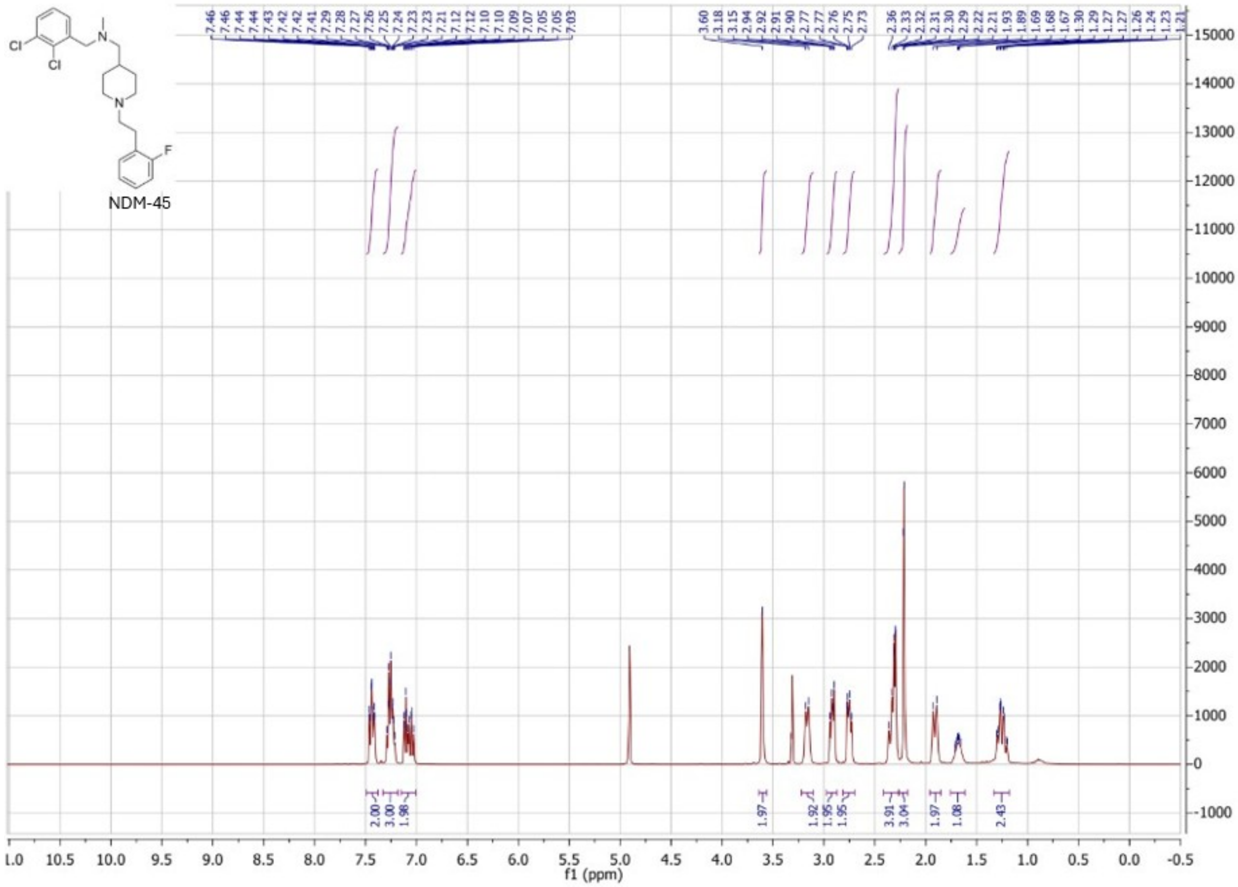

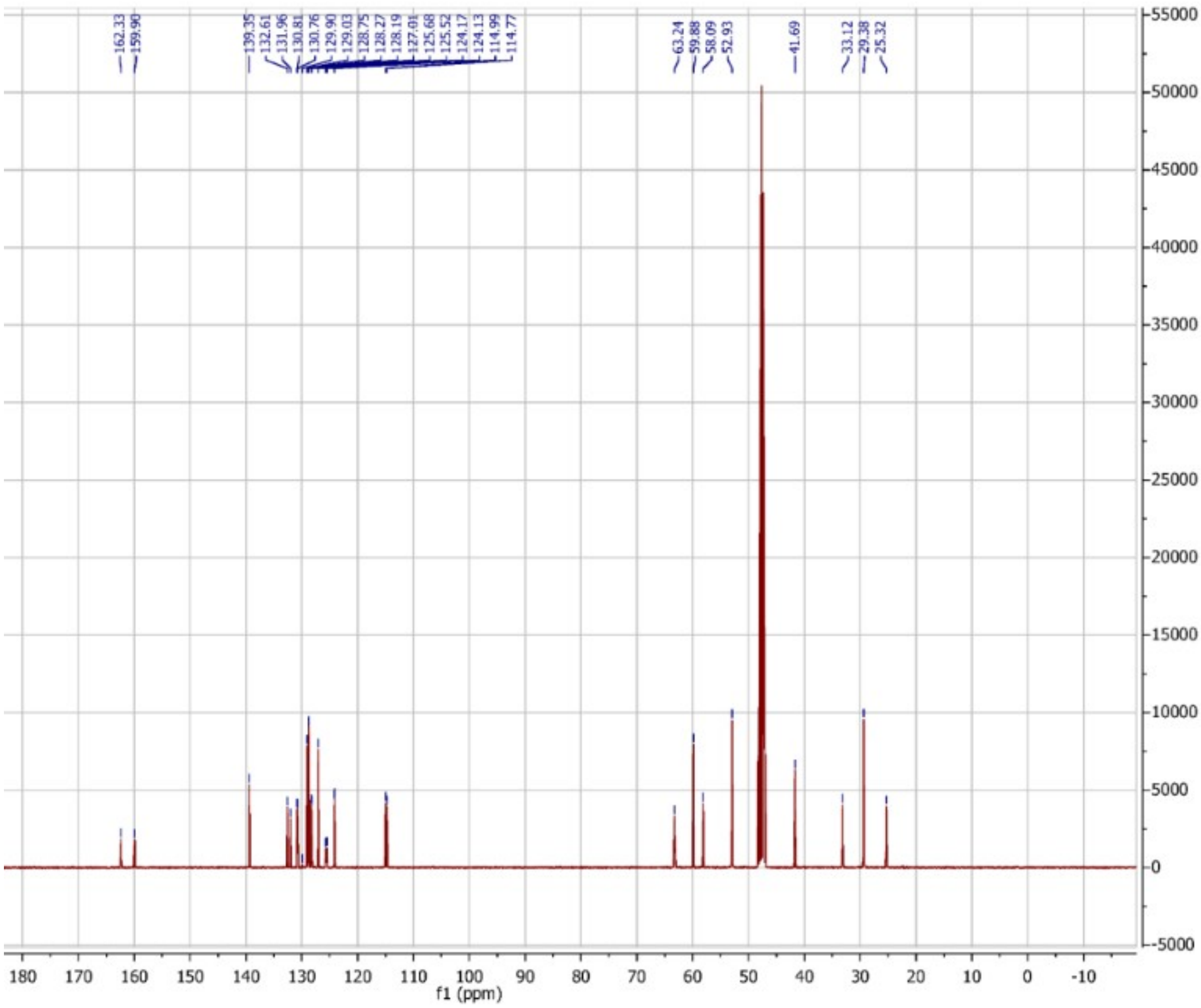
162.33
159.90
139.35
132.61
131.96
130.81
130.76
129.90
129.03
128.75
128.27
128.19
127.01
125.68
125.52
124.17
124.13
114.99
114.77
63.24
59.88
58.09
52.93
41.69
33.12
29.38
25.32
f1 (ppm)

***N*-(3,4-Dichlorobenzyl)-1-(1-(2-fluorophenethyl)piperidin-4-yl)-*N*-methylmethanamine (NDM-46):**

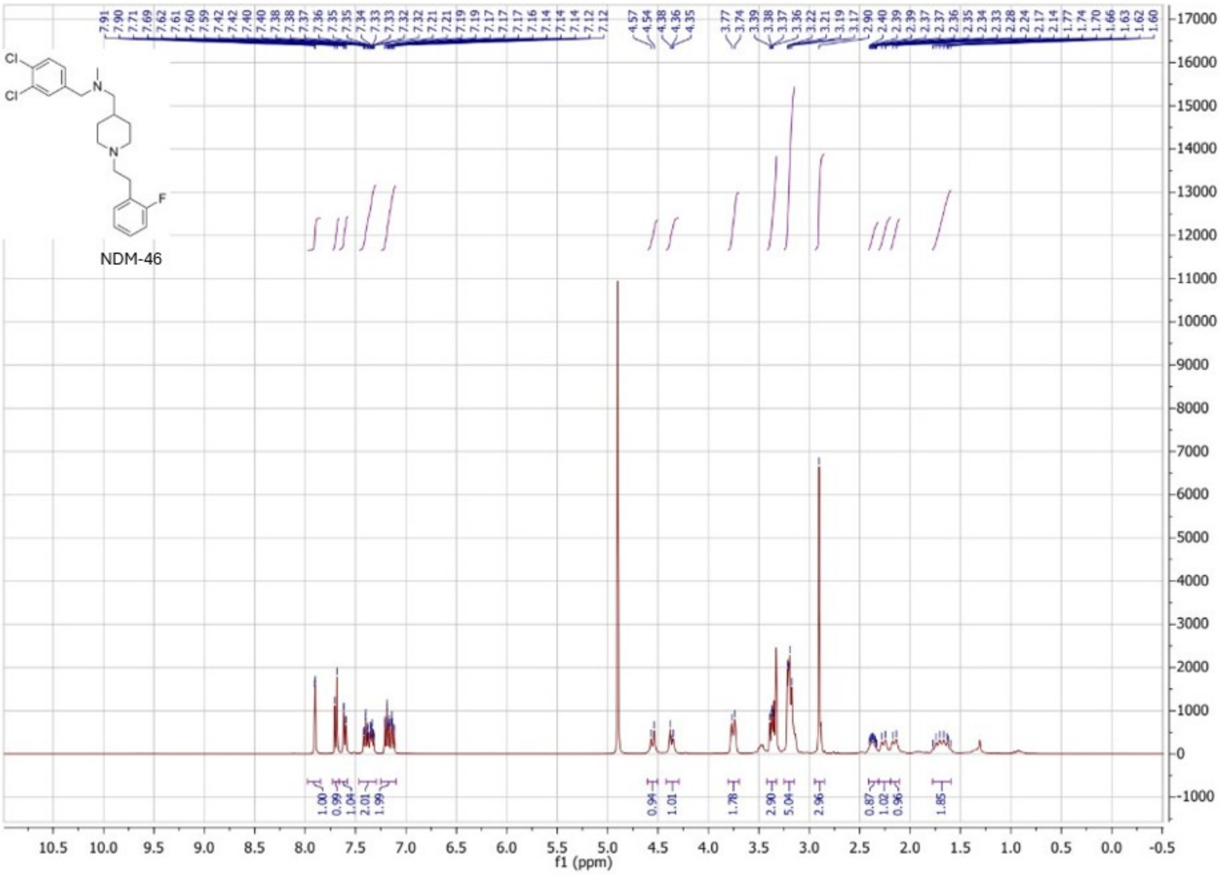

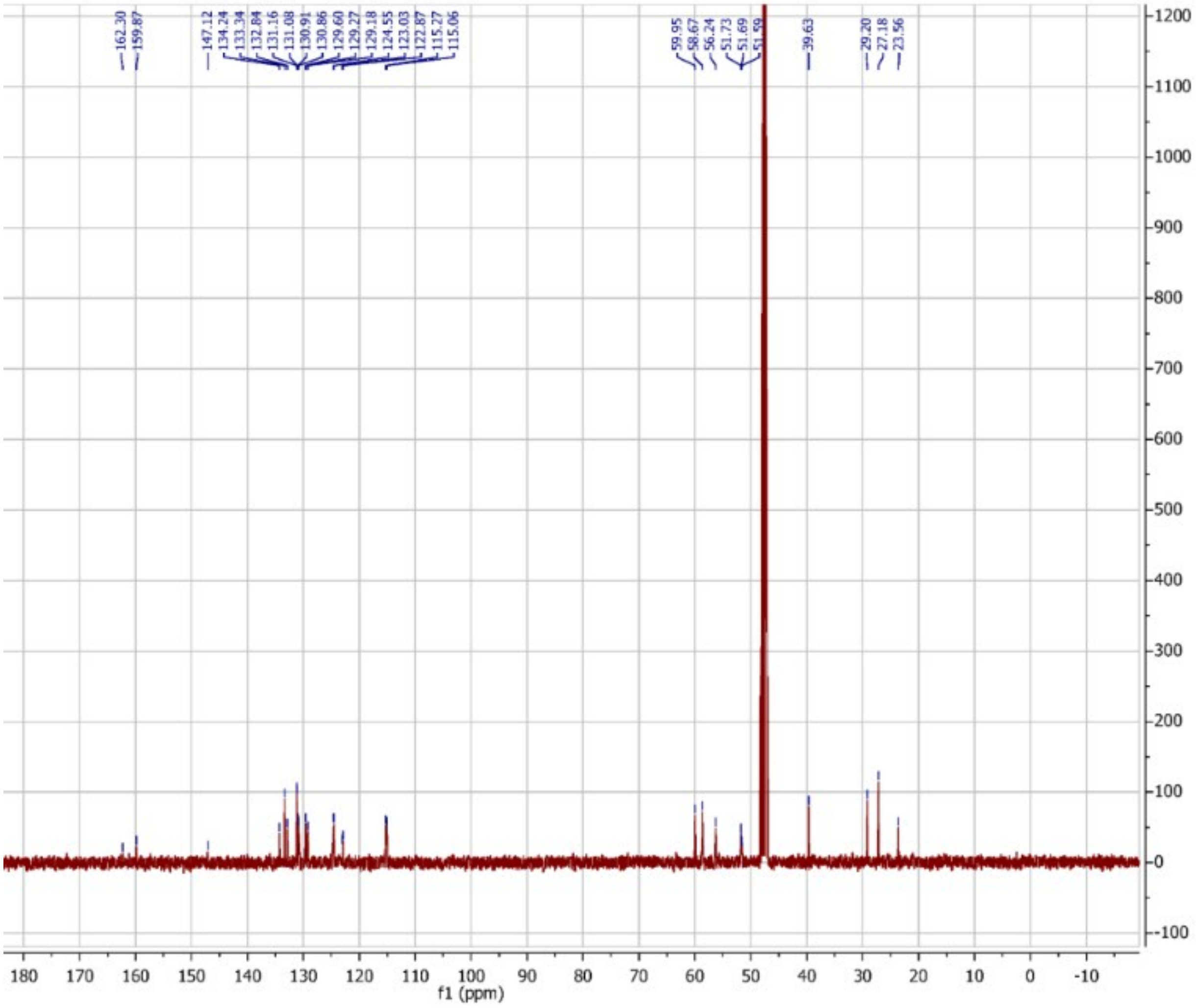

162.30
159.87
147.12
134.24
133.34
132.84
131.16
131.08
130.91
130.86
129.60
129.27
129.18
124.55
123.03
122.87
115.27
115.06
59.95
58.67
56.24
51.73
51.69
51.59
39.63
29.20
27.18
23.56
f1 (ppm)

1-([1,1'-Biphenyl]-4-yl)-*N*-((1-(2-fluorophenethyl)piperidin-4-yl)methyl)-*N*-methylmethanamine (NDM-564):

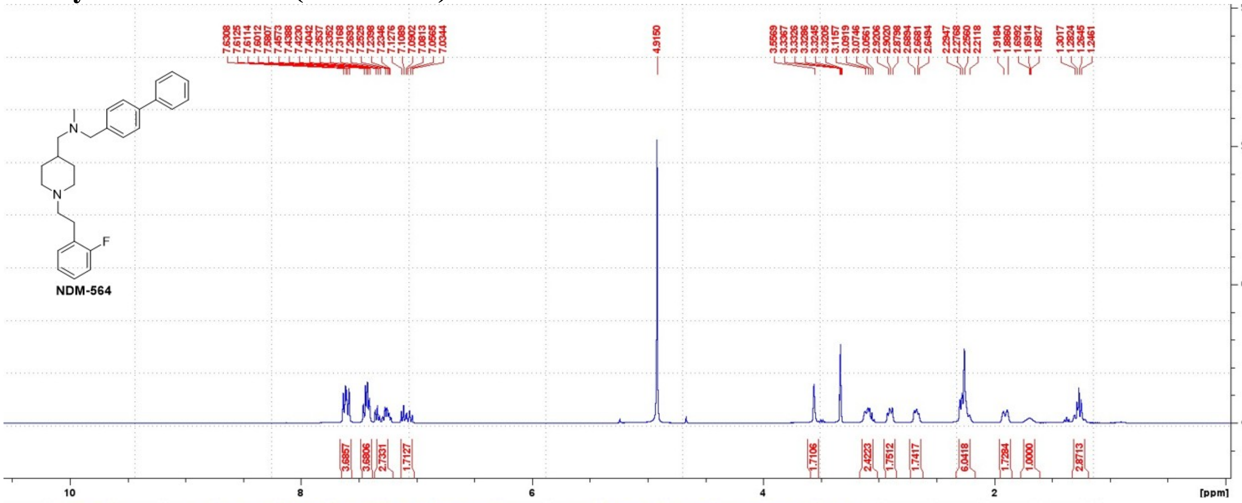

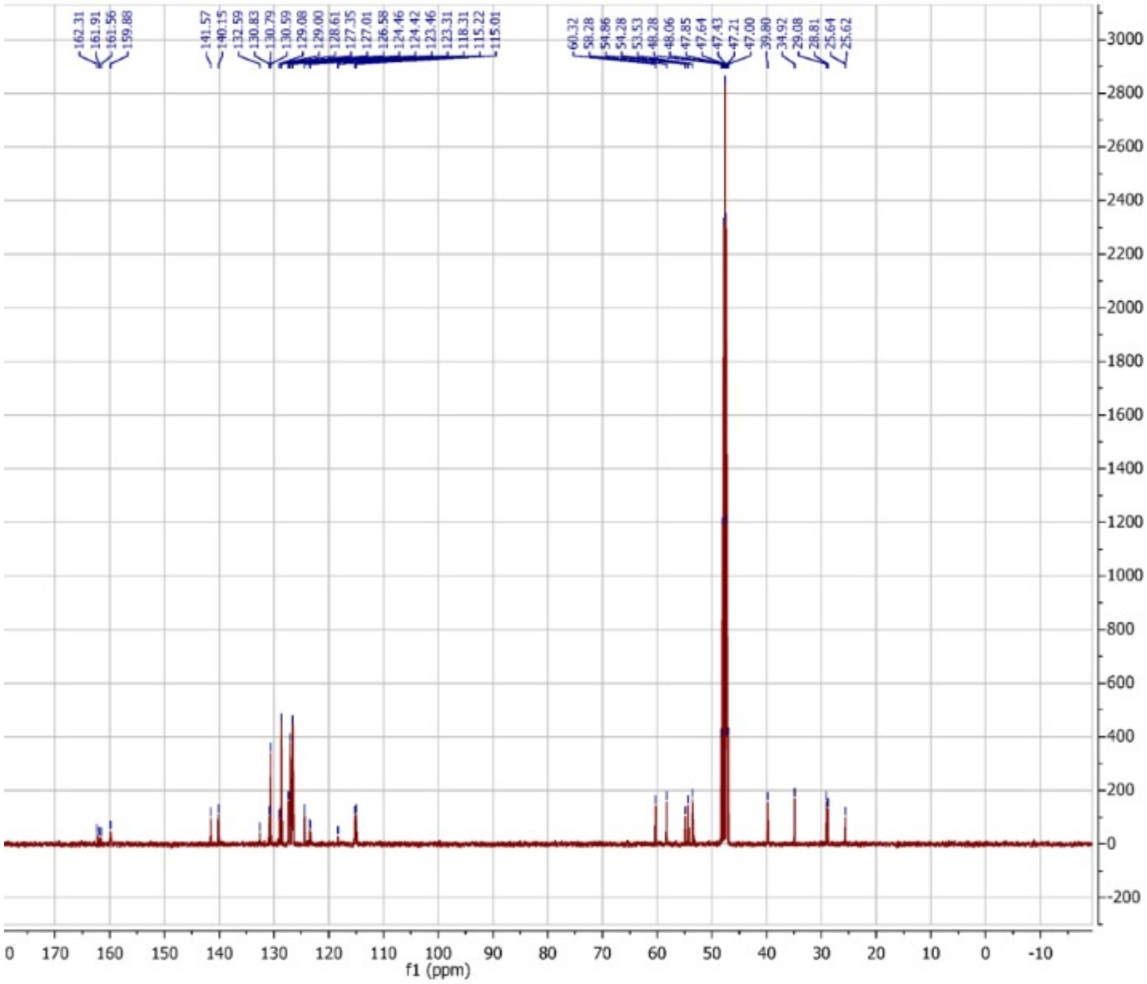

1-(1-(2-Fluorophenethyl)piperidin-4-yl)-*N*-methyl-*N*-(naphthalen-1-ylmethyl)methanamine (NDM-602):

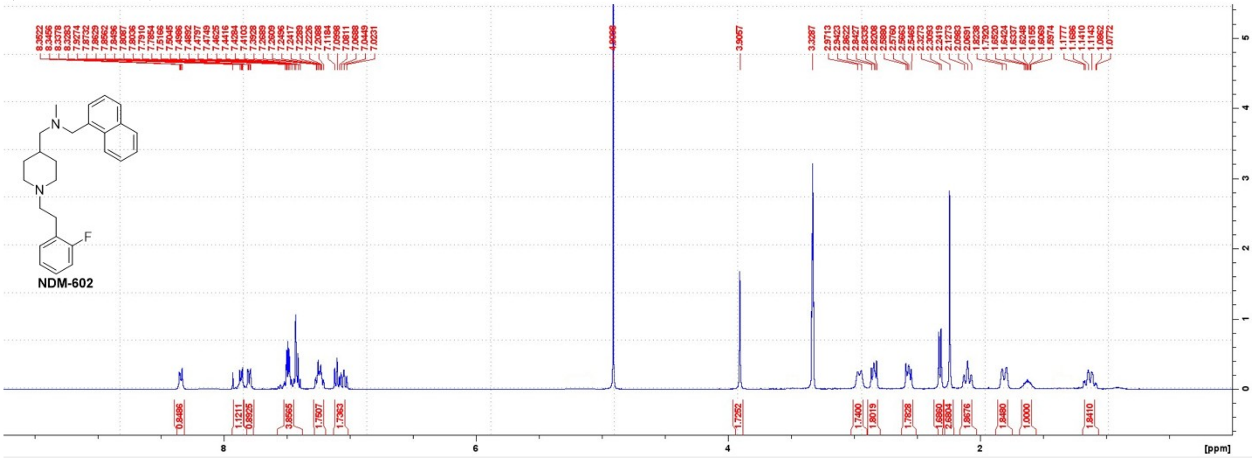

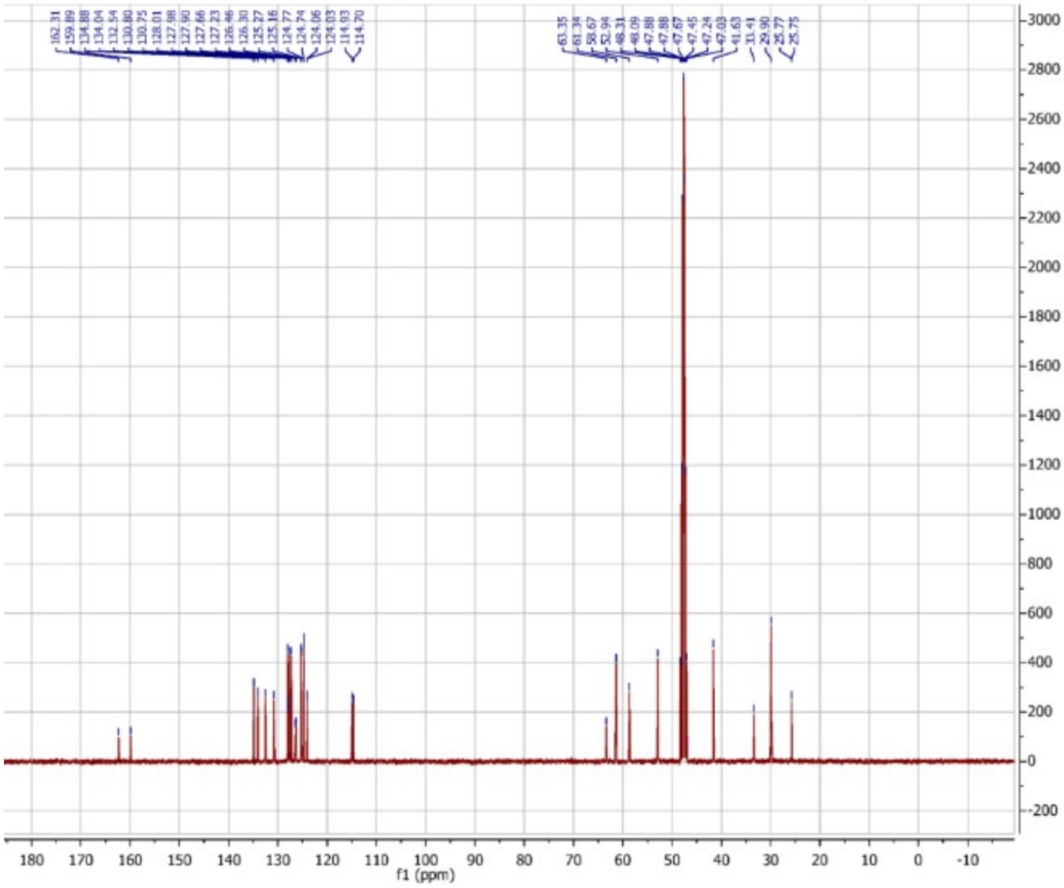

1-(1-(2-Fluorophenethyl)piperidin-4-yl)-*N*-methyl-*N*-(naphthalen-2-ylmethyl)methanamine (NDM-600):

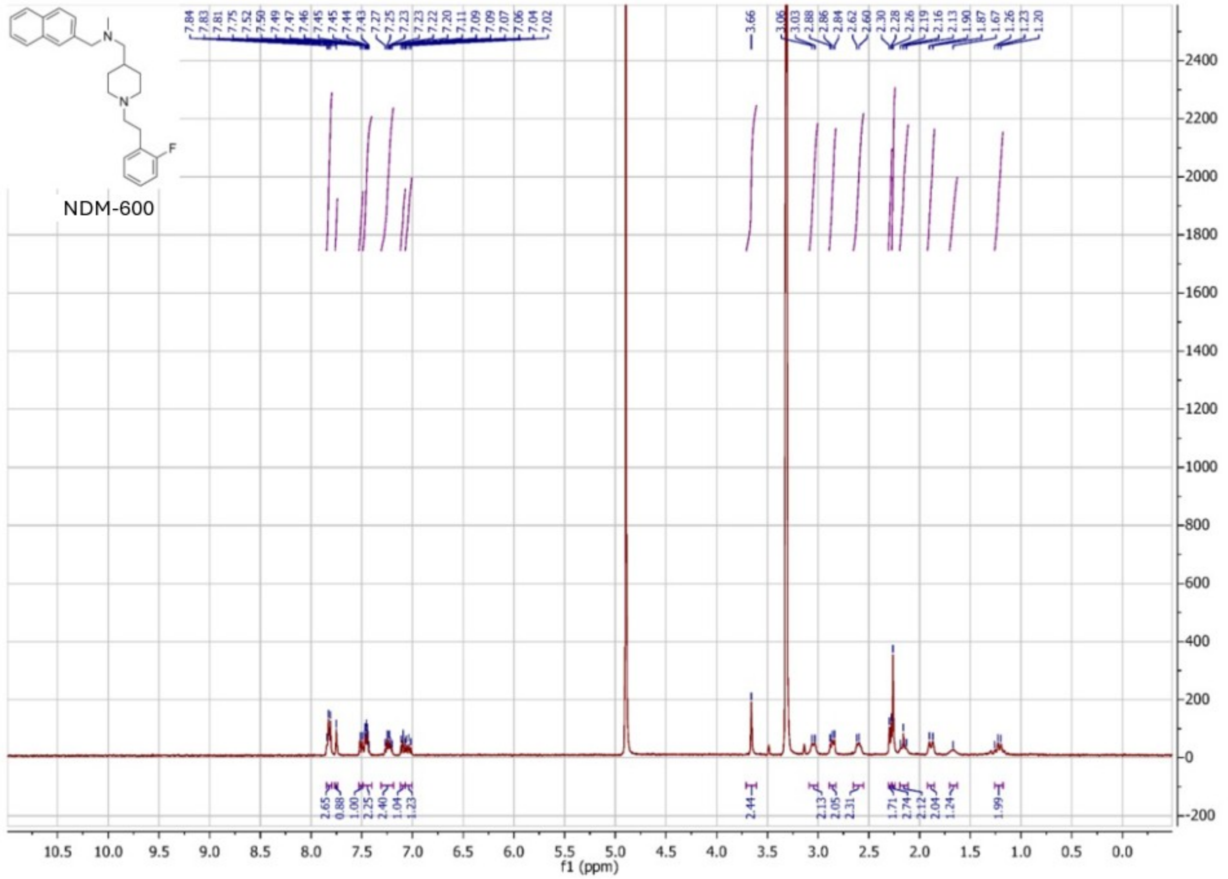

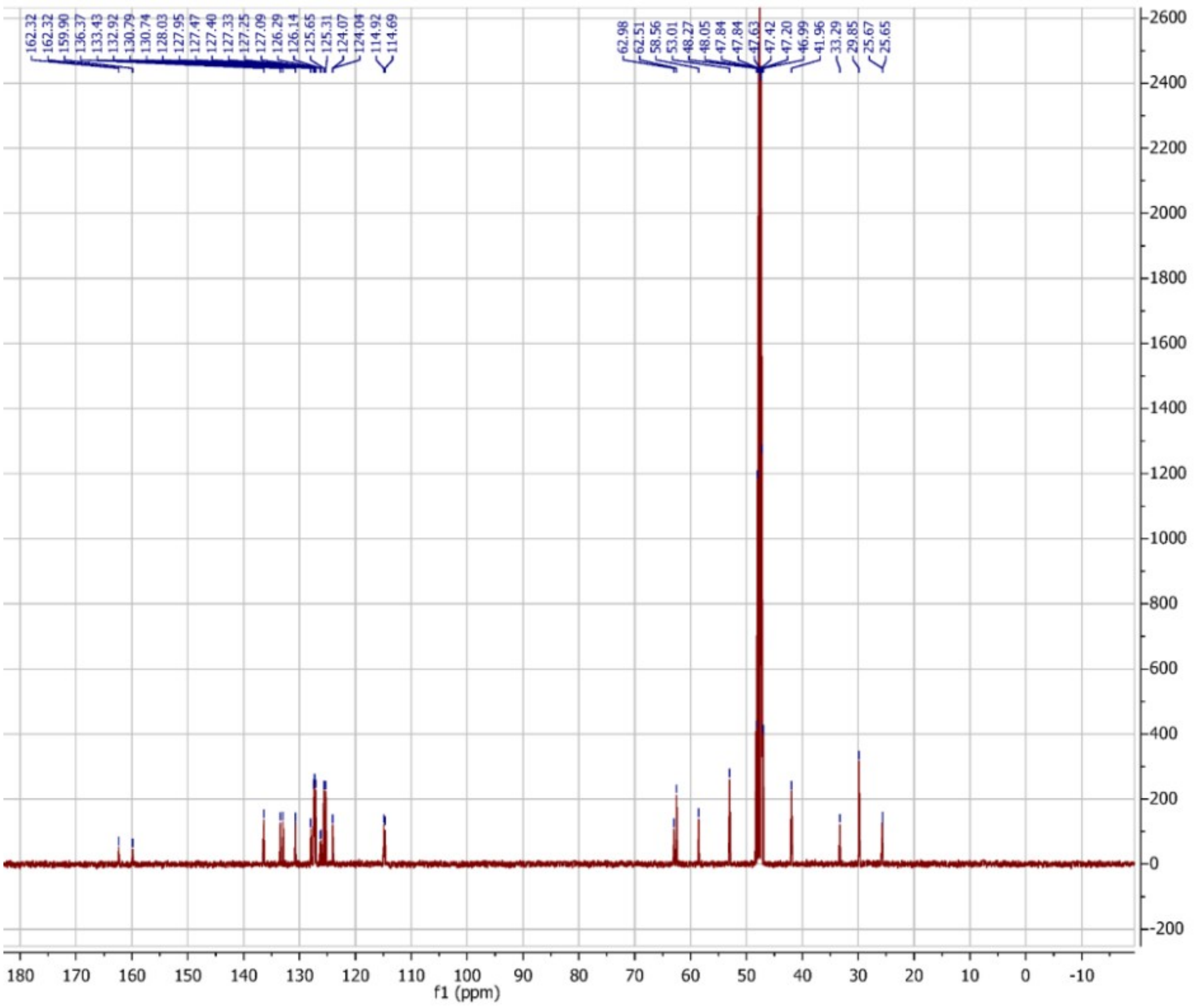

162.32
162.32
159.90
136.37
133.43
132.92
130.79
130.74
128.03
127.95
127.47
127.40
127.33
127.25
127.09
126.29
126.14
125.65
125.31
124.07
124.04
114.92
114.69
62.98
62.51
58.56
53.01
48.27
48.05
47.84
47.84
47.63
47.42
47.20
46.99
41.96
33.29
29.85
25.67
25.65
f1 (ppm)

***N*-Benzyl-1-(1-(2,4-dichlorophenethyl)piperidin-4-yl)-*N*-methylmethanamine (NDM-53):**

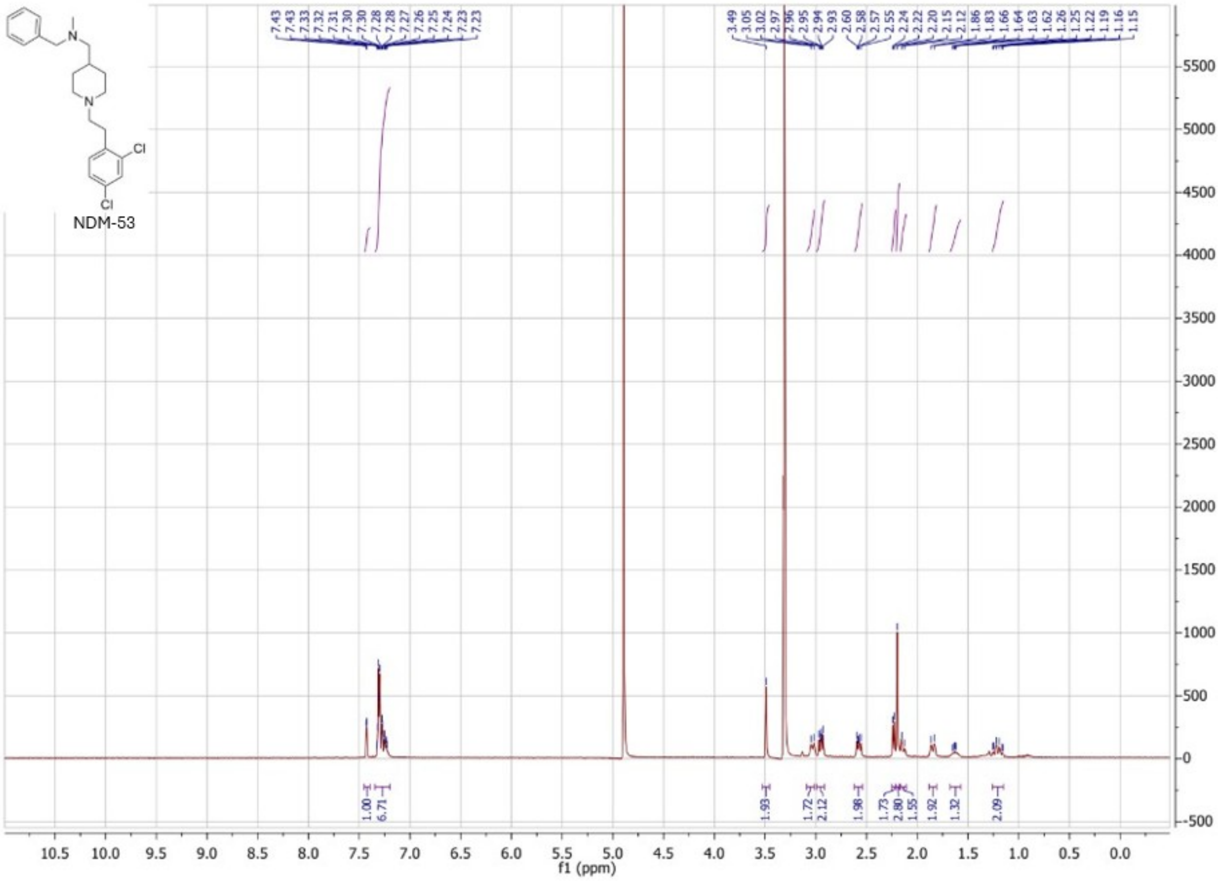

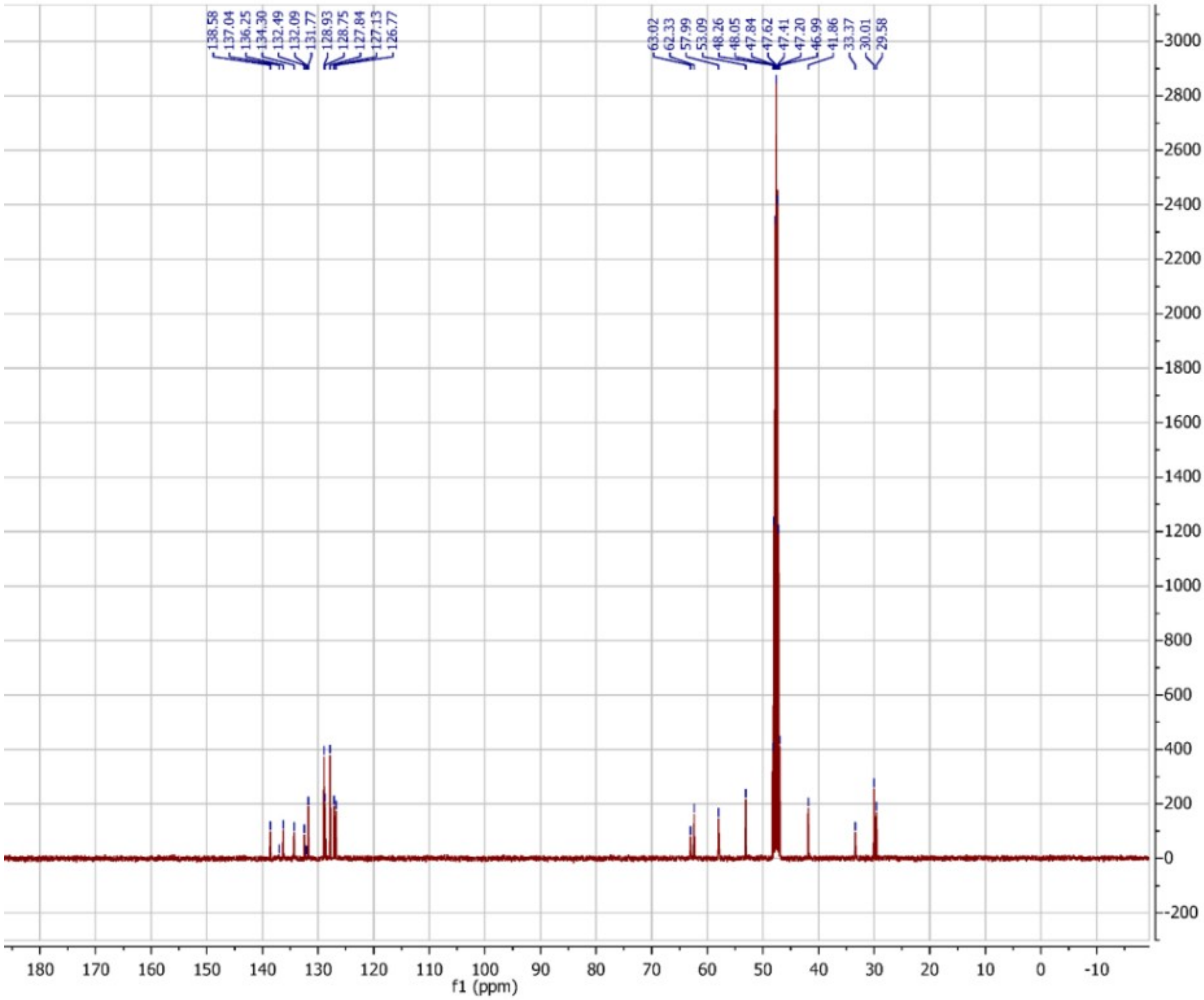
138.58
137.04
136.25
134.30
132.49
132.09
131.77
128.93
128.75
127.84
127.13
126.77
63.02
62.33
57.99
53.09
48.26
48.05
47.84
47.62
47.41
47.20
46.99
41.86
33.37
30.01
29.58
f1 (ppm)

1-(1-(2,4-Dichlorophenethyl)piperidin-4-yl)-*N*-(2-fluorobenzyl)-*N*-methylmethanamine (NDM-590):

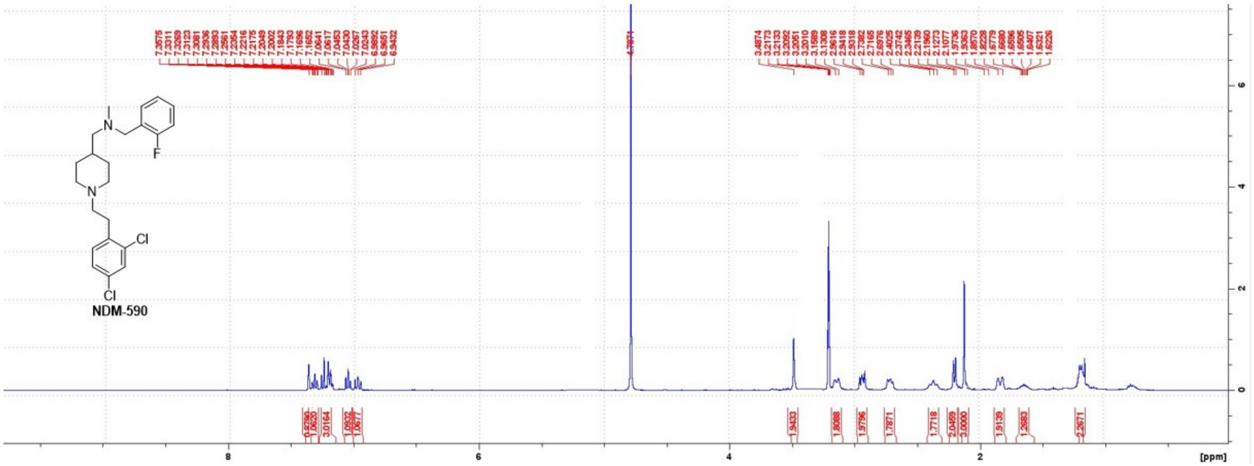

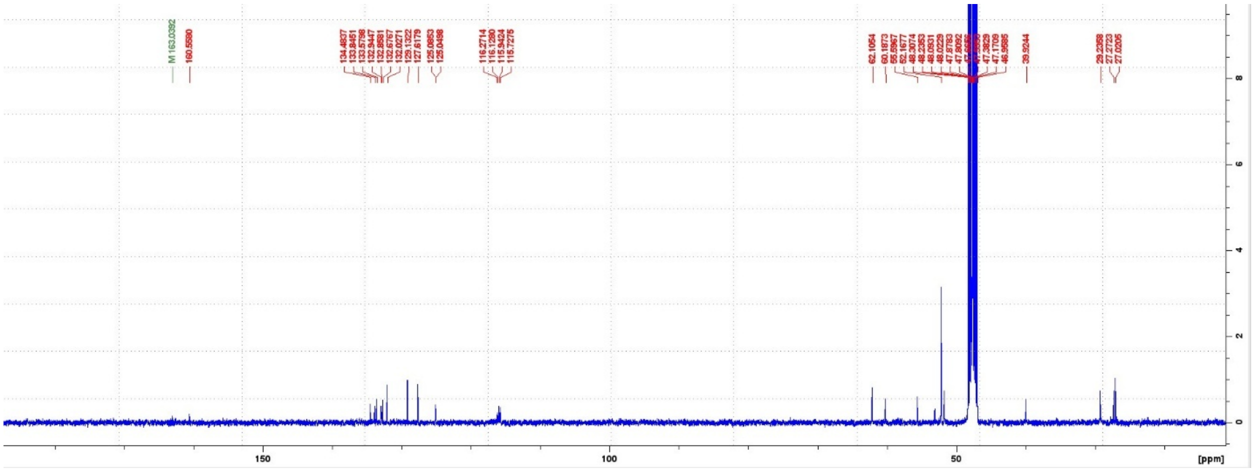

***N*-(2-Chlorobenzyl)-1-(1-(2,4-dichlorophenethyl)piperidin-4-yl)-*N*-methylmethanamine (NDM-59):**

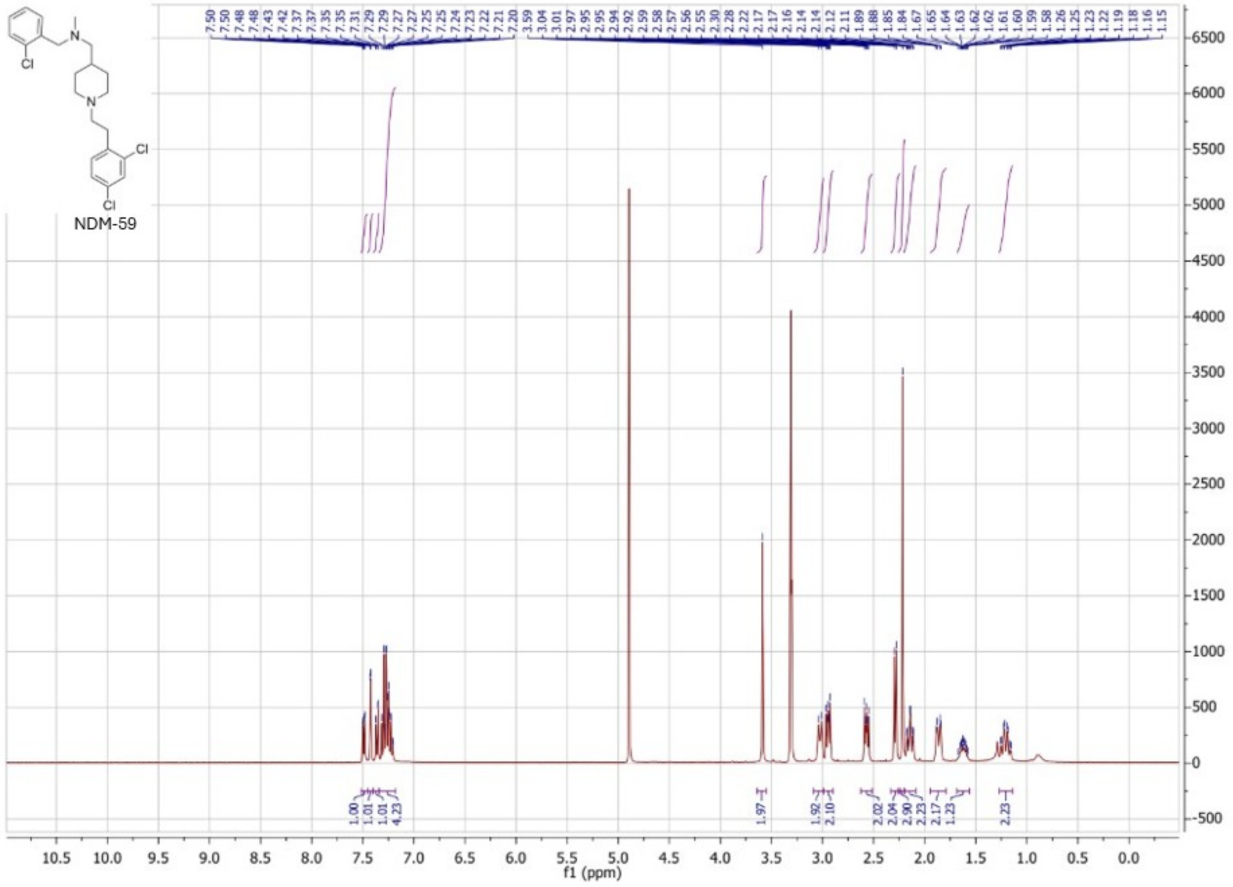

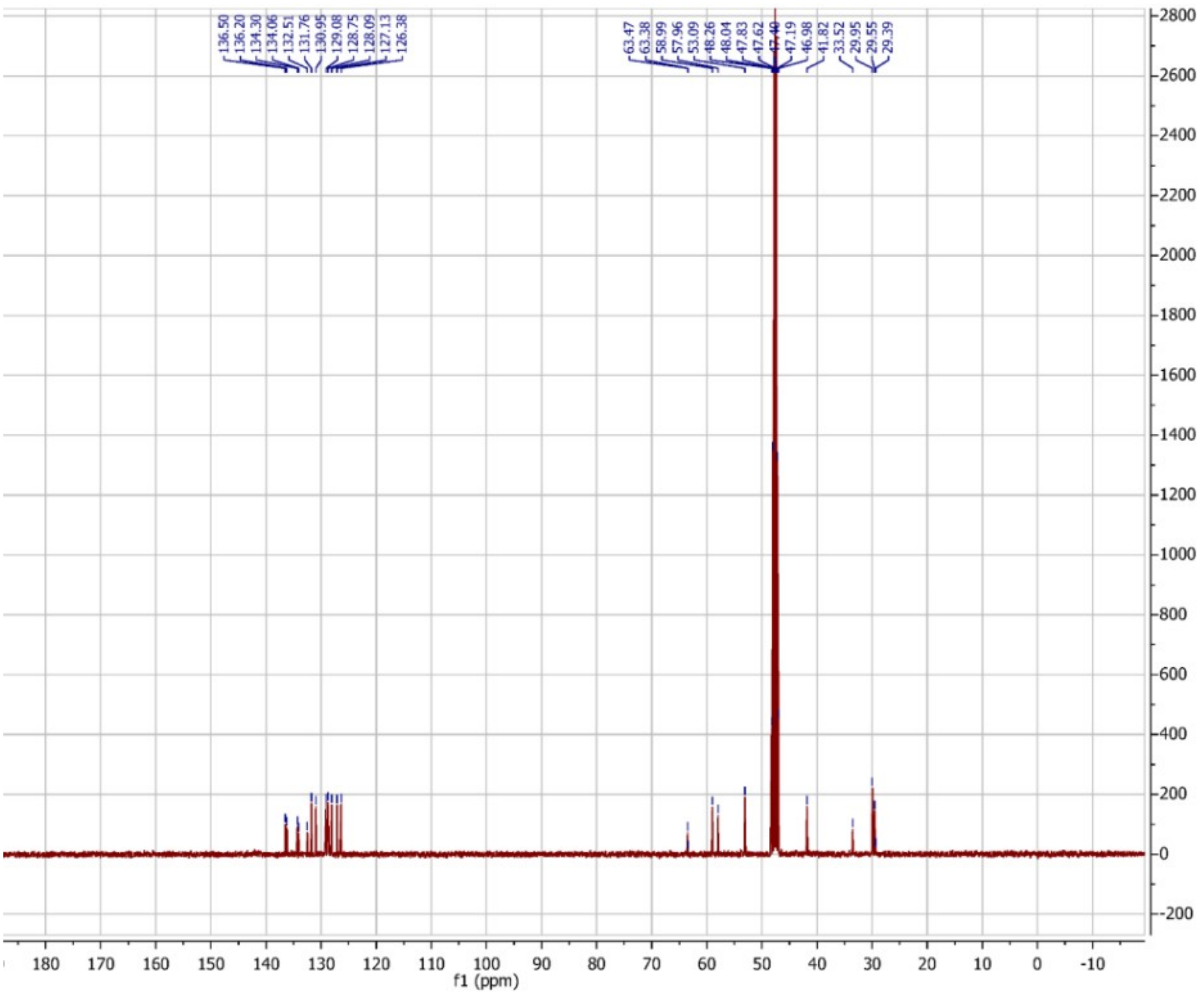
136.50
136.20
134.30
134.06
132.51
131.76
130.95
129.08
128.75
128.09
127.13
126.38
63.47
63.38
58.99
57.96
53.09
48.26
48.04
47.83
47.62
47.40
47.19
46.98
41.82
33.52
29.95
29.55
29.39
f1 (ppm)

N-(3-Chlorobenzyl)-1-(1-(2,4-dichlorophenethyl)piperidin-4-yl)-*N*-methylmethanamine (NDM-58):

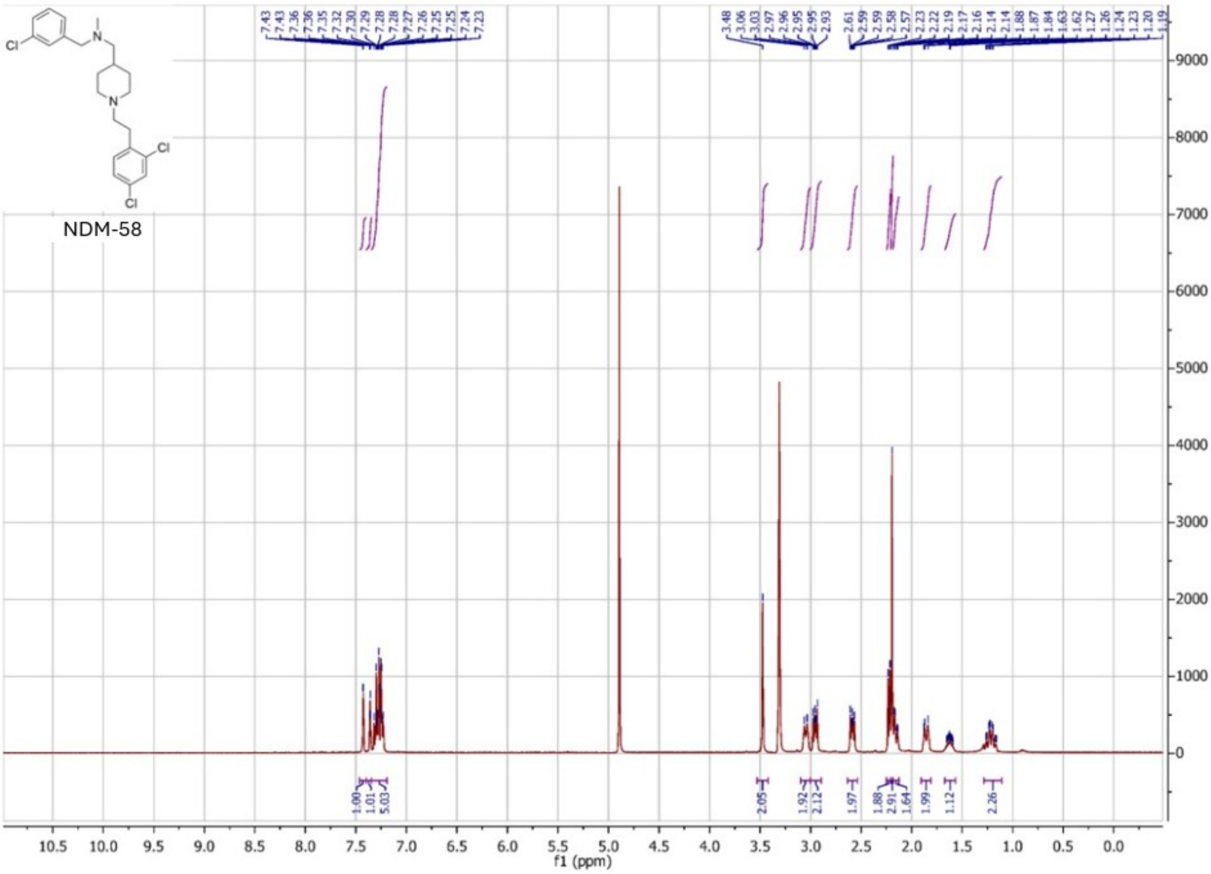

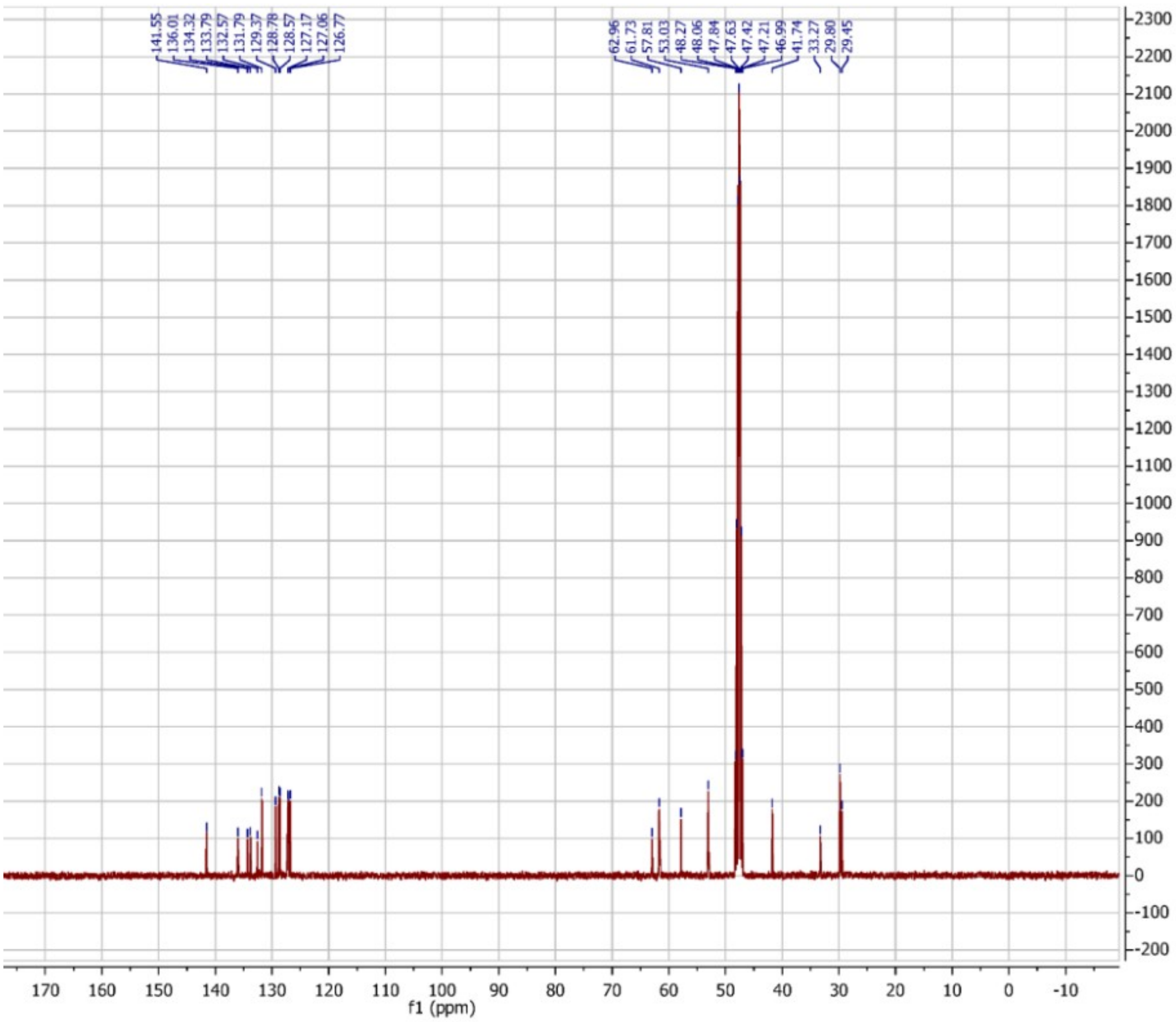
141.55
136.01
134.32
133.79
132.57
131.79
129.37
128.78
128.57
127.17
127.06
126.77
62.96
61.73
57.81
53.03
48.27
48.06
47.84
47.63
47.42
47.21
46.99
41.74
33.27
29.80
29.45
f1 (ppm)

1-(1-(2,4-Dichlorophenethyl)piperidin-4-yl)-*N*-(3-iodobenzyl)-*N*-methylmethanamine (NDM-61):

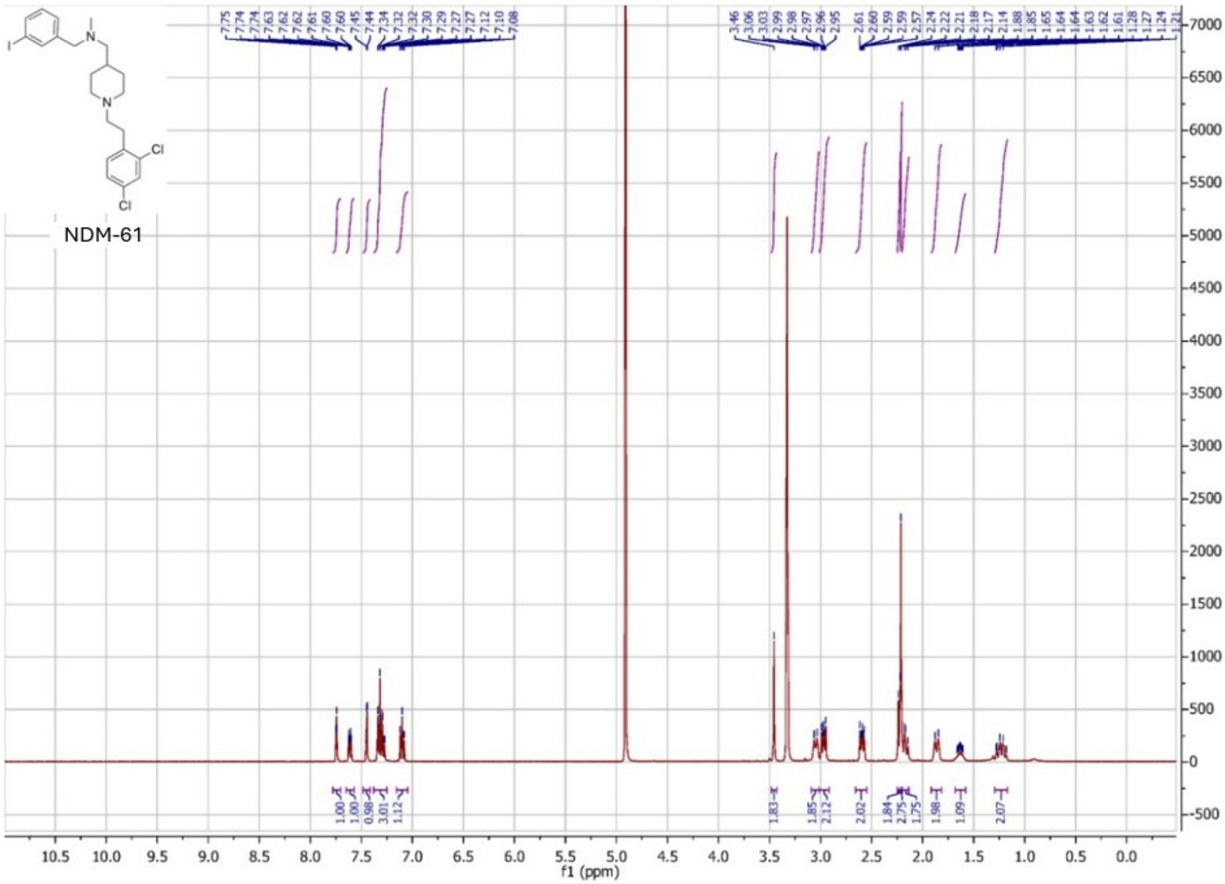

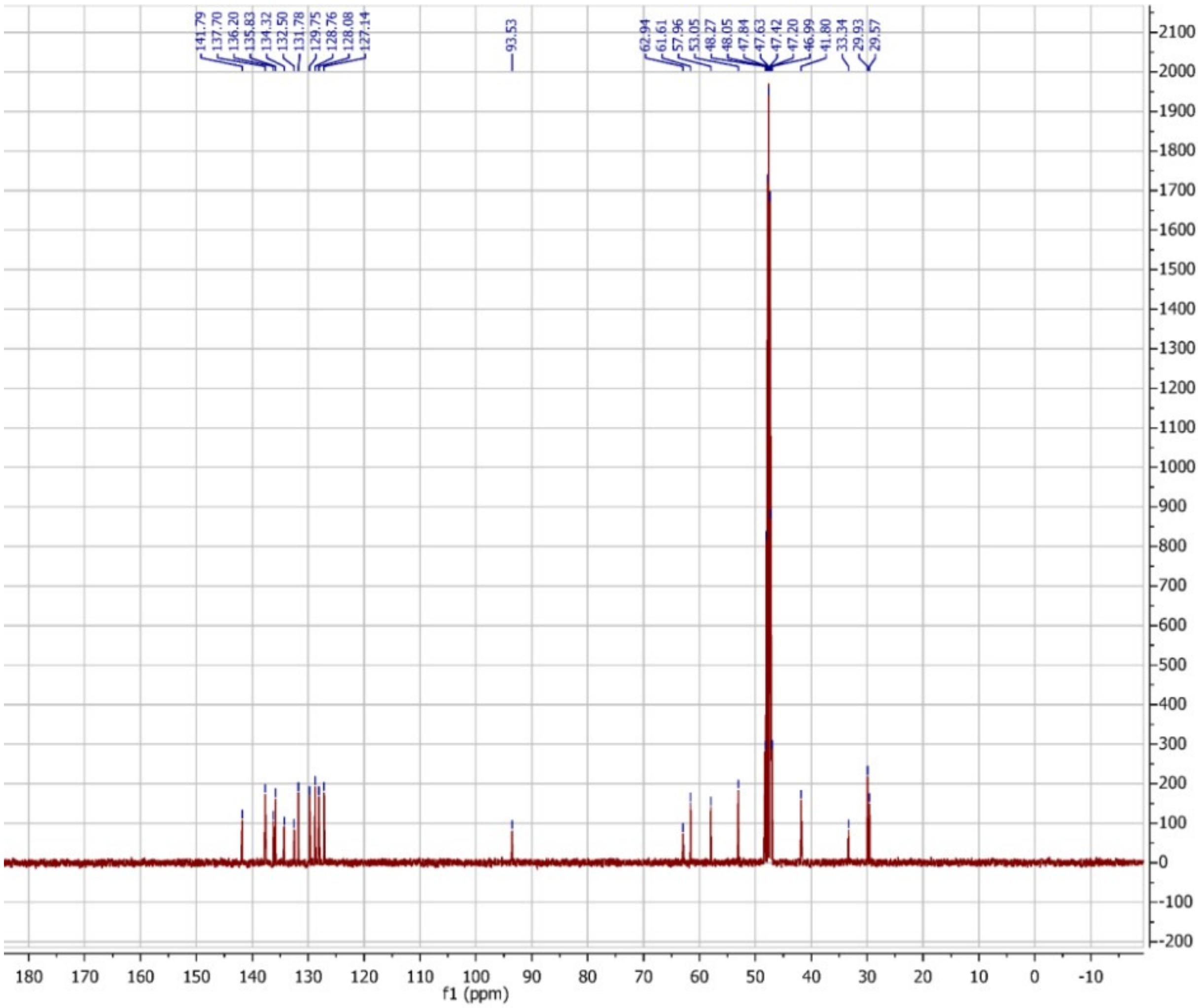

141.79
137.70
136.20
135.83
134.32
132.50
131.78
129.75
128.76
128.08
127.14
93.53
62.94
61.61
57.96
53.05
48.27
48.05
47.84
47.63
47.42
47.20
46.99
41.80
33.34
29.93
29.57
f1 (ppm)

1-(1-(2,4-Dichlorophenethyl)piperidin-4-yl)-*N*-methyl-*N*-(3-methylbenzyl)methanamine (NDM-589):

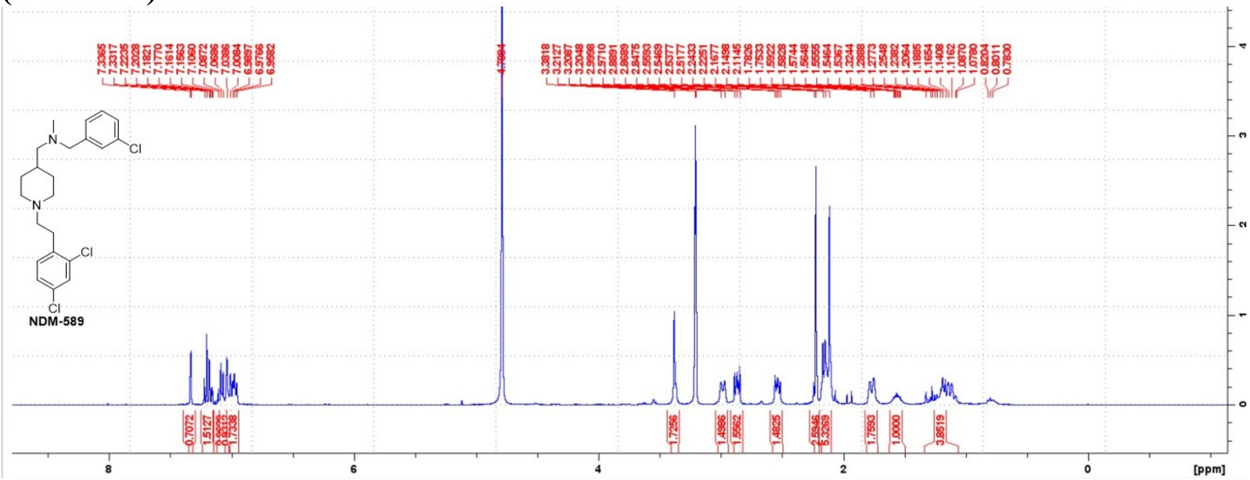

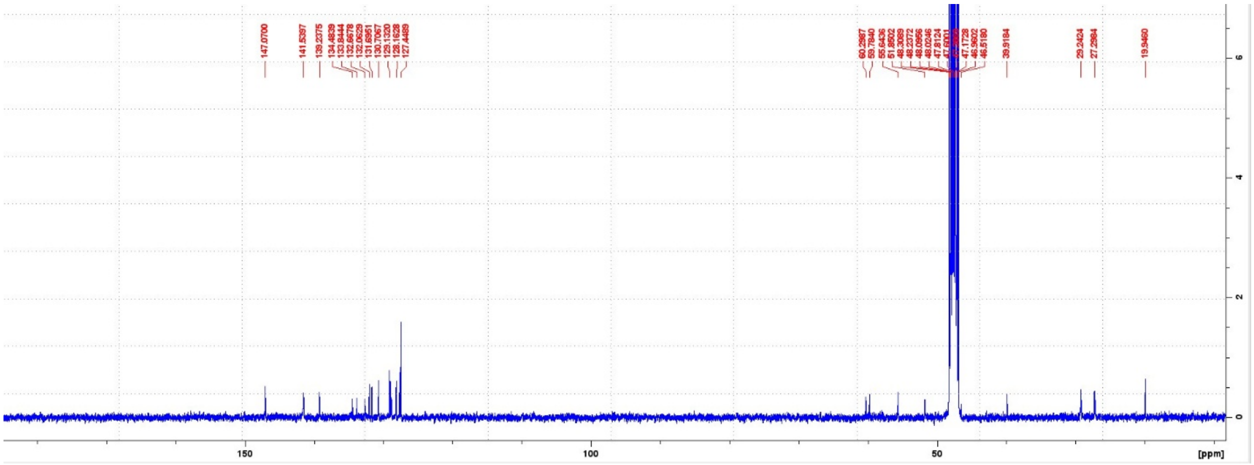
147.0700
141.5397
139.2375
134.4839
133.8444
132.6678
132.0629
131.6951
130.7067
129.1320
128.1628
127.4489
60.2987
59.7840
55.6436
51.8502
48.3089
48.2372
48.0956
48.0246
47.8124
47.1728
46.9602
46.5180
39.9184
29.2424
27.2984
19.9460
150
100
50
[ppm]
6
4
2
0

N-(2,3-Dichlorobenzyl)-1-(1-(2,4-dichlorophenethyl)piperidin-4-yl)-*N*-methylmethanamine (NDM-55):

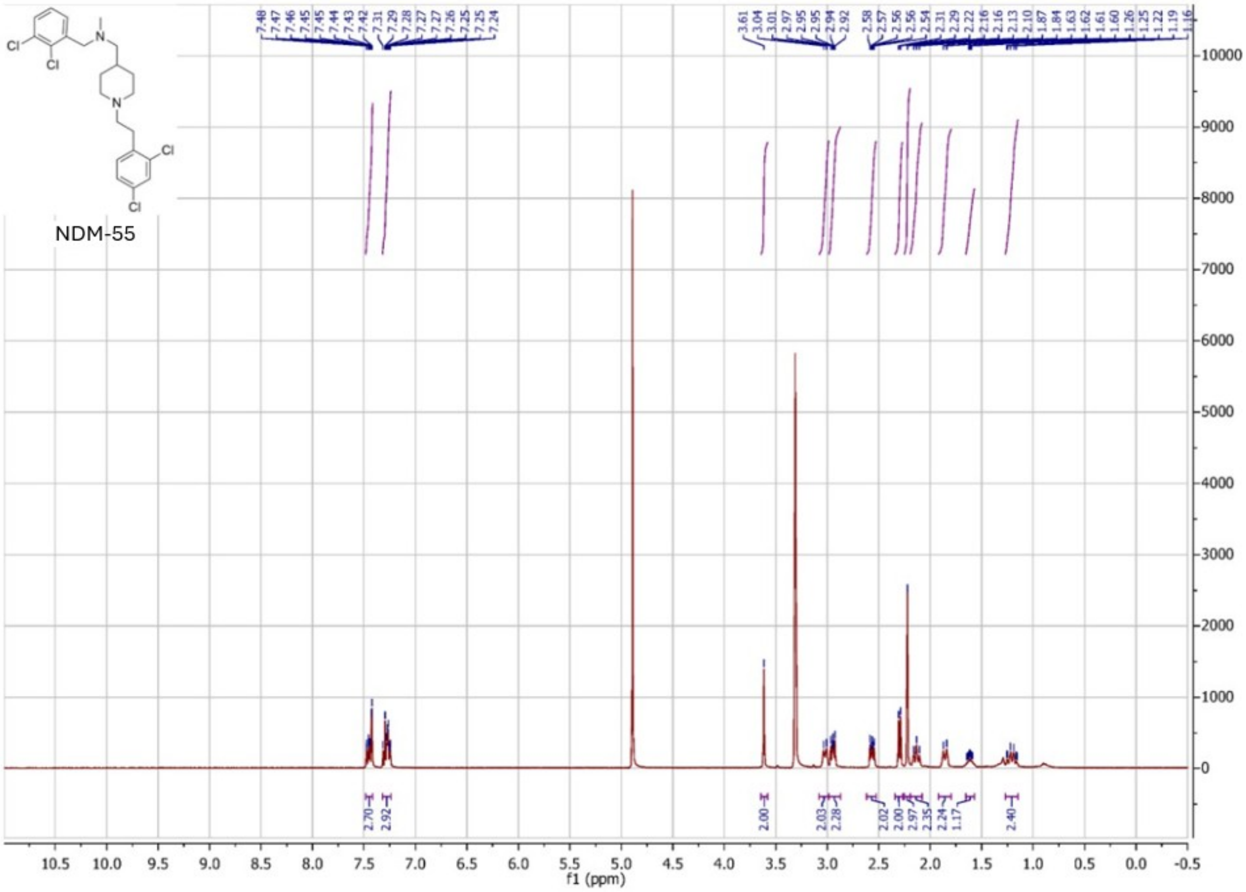

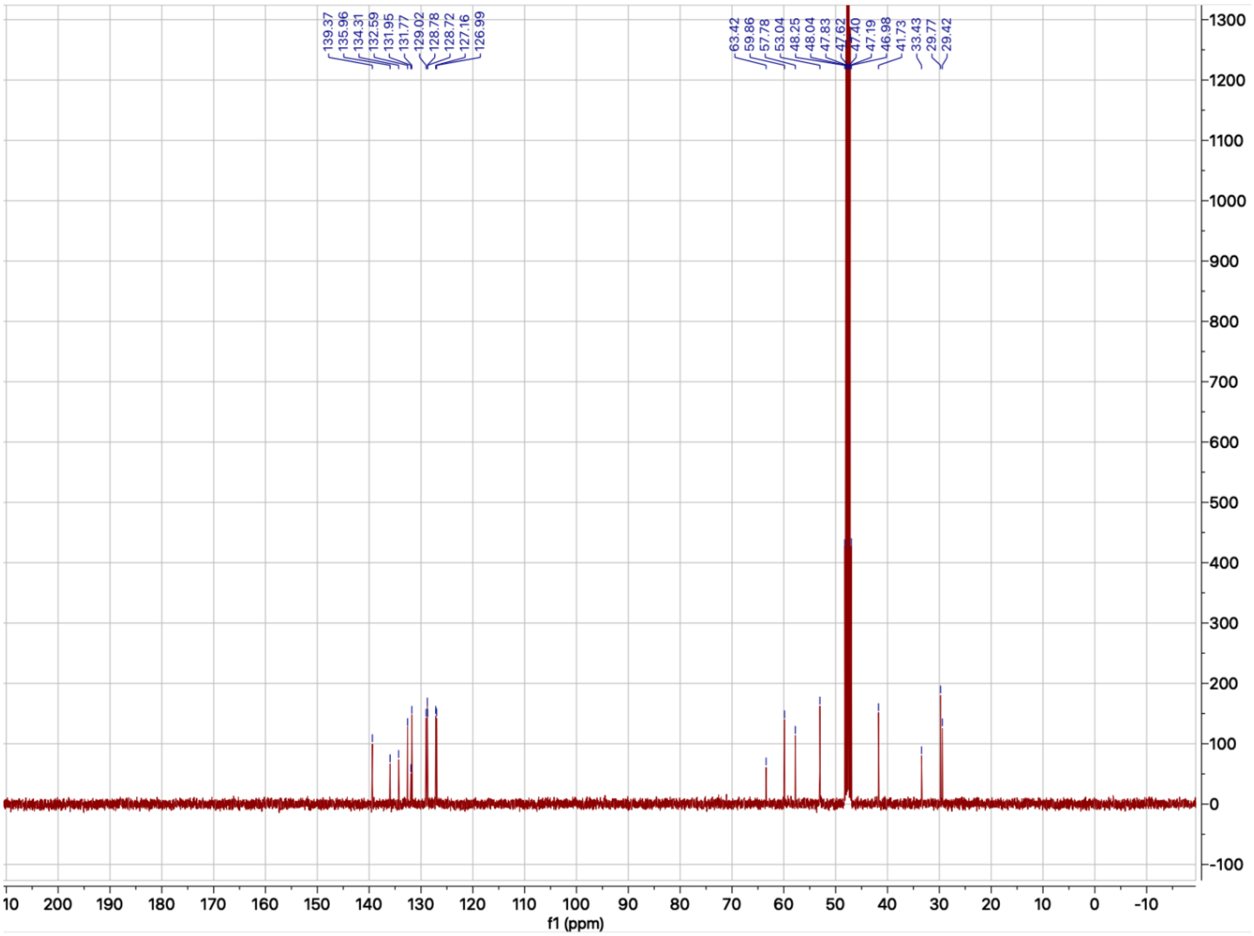
139.37
135.96
134.31
132.59
131.95
131.77
129.02
128.78
128.72
127.16
126.99
63.42
59.86
57.78
53.04
48.25
48.04
47.83
47.62
47.40
47.19
46.98
41.73
33.43
29.77
29.42
f1 (ppm)

***N*-(3,4-Dichlorobenzyl)-1-(1-(2,4-dichlorophenethyl)piperidin-4-yl)-*N*-methylmethanamine (NDM-56):**

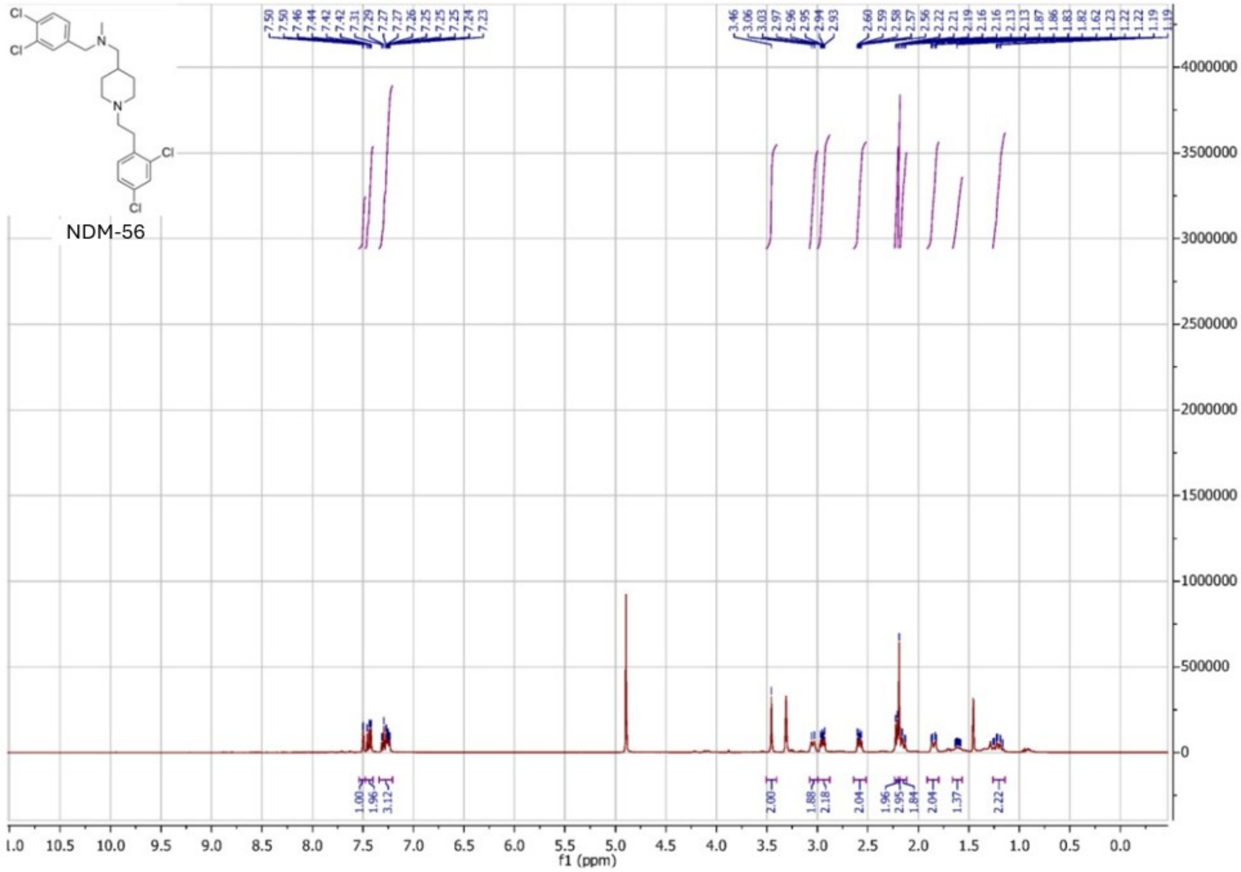

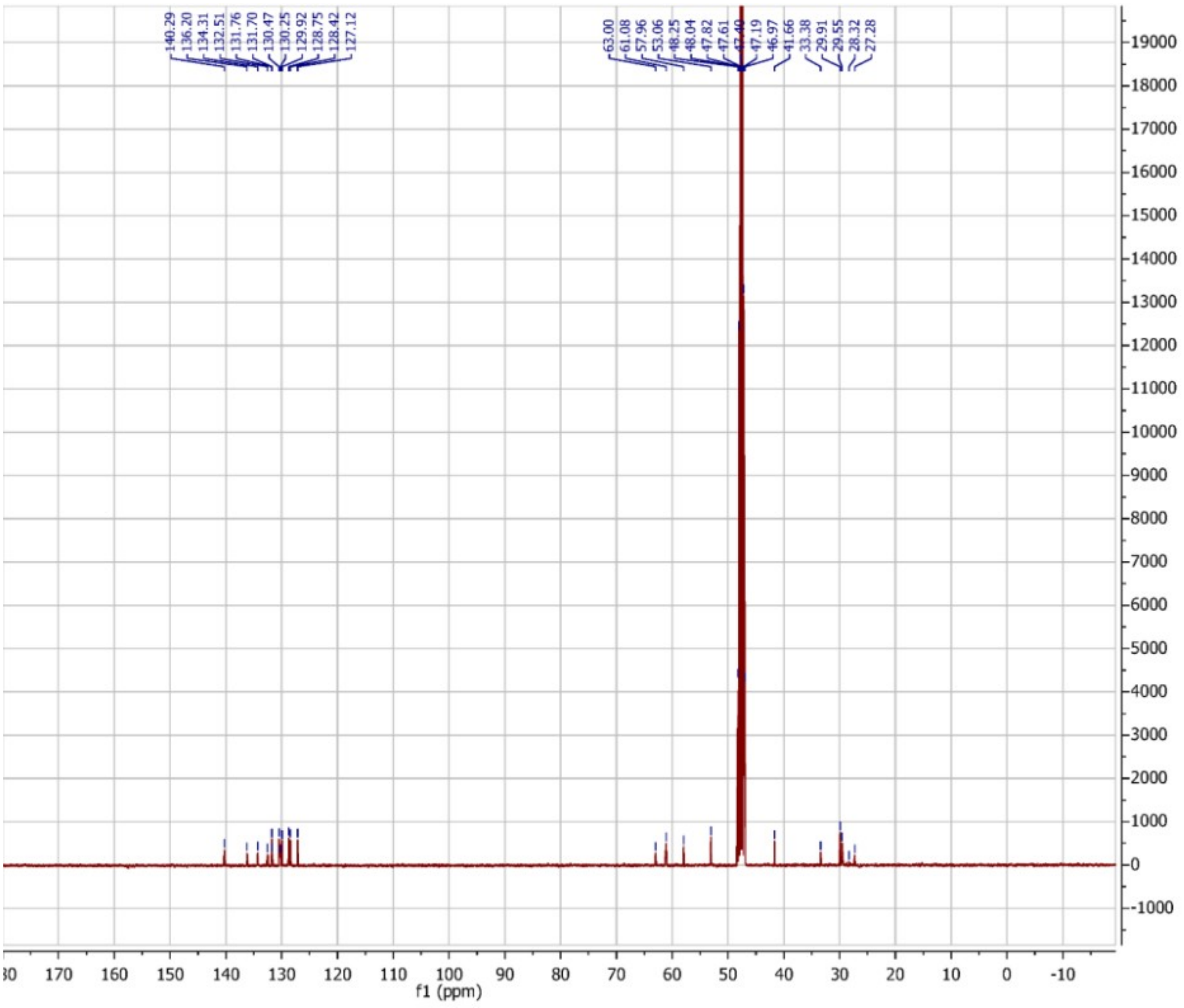
140.29
136.20
134.31
132.51
131.76
131.70
130.47
130.25
129.92
128.75
128.42
127.12
63.00
61.08
57.96
53.06
48.25
48.04
47.82
47.61
47.40
47.19
46.97
41.66
33.38
29.91
29.55
28.32
27.28
f1 (ppm)

1-(1-(2,4-Dichlorophenethyl)piperidin-4-yl)-*N*-methyl-*N*-(naphthalen-2-ylmethyl)methanamine (NDM-598):

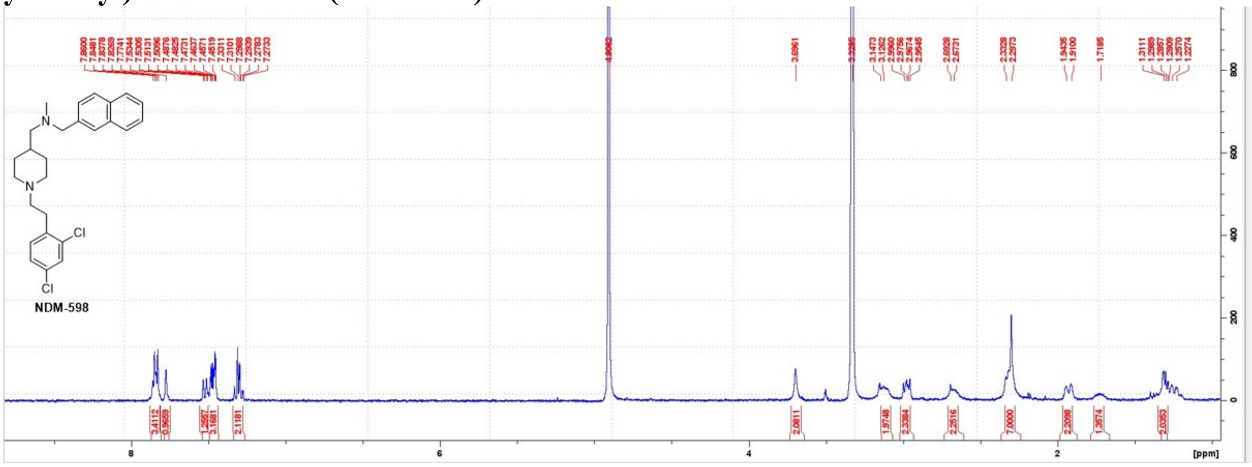

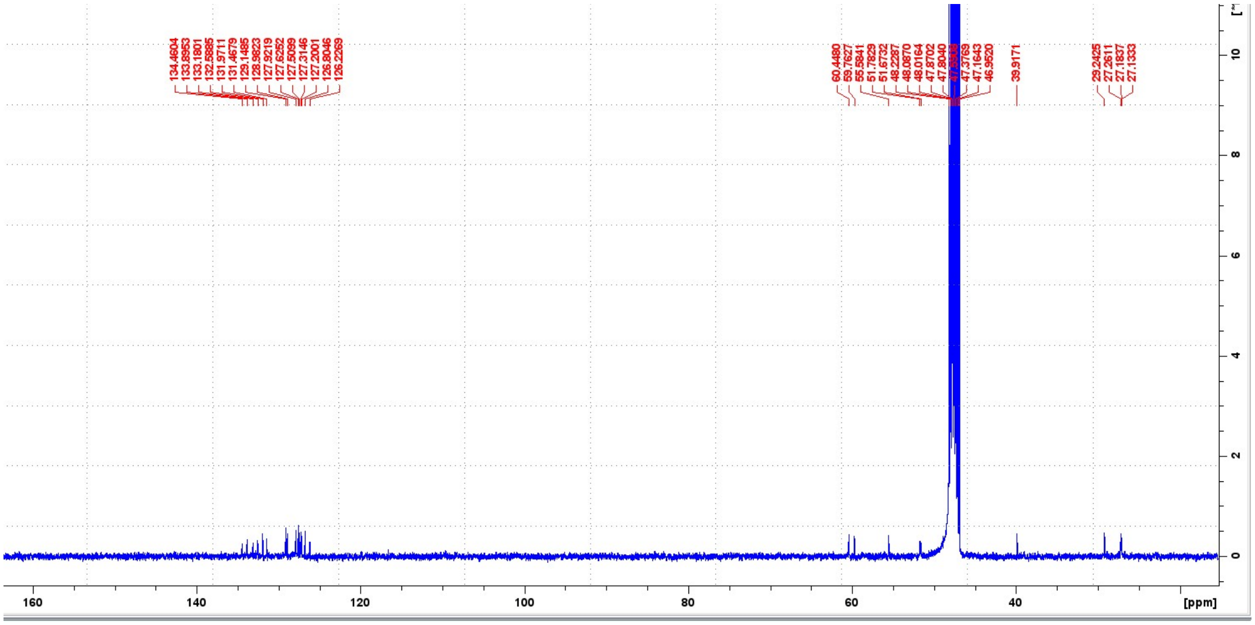

134.4604
133.8953
133.1801
132.5885
131.9711
131.4679
129.1485
128.9823
127.9219
127.6252
127.5099
127.3146
127.2001
126.8046
126.2269
60.4480
59.7627
55.5841
51.7829
51.6732
48.2287
48.0870
48.0164
47.8702
47.8040
47.3769
47.1643
46.9520
39.9171
29.2425
27.2611
27.1837
27.1333
10
8
6
4
2
0
160
140
120
100
80
60
40
[ppm]

***N*-Benzyl-1-(1-(3,4-dichlorophenethyl)piperidin-4-yl)-*N*-methylmethanamine (NDM-37):**

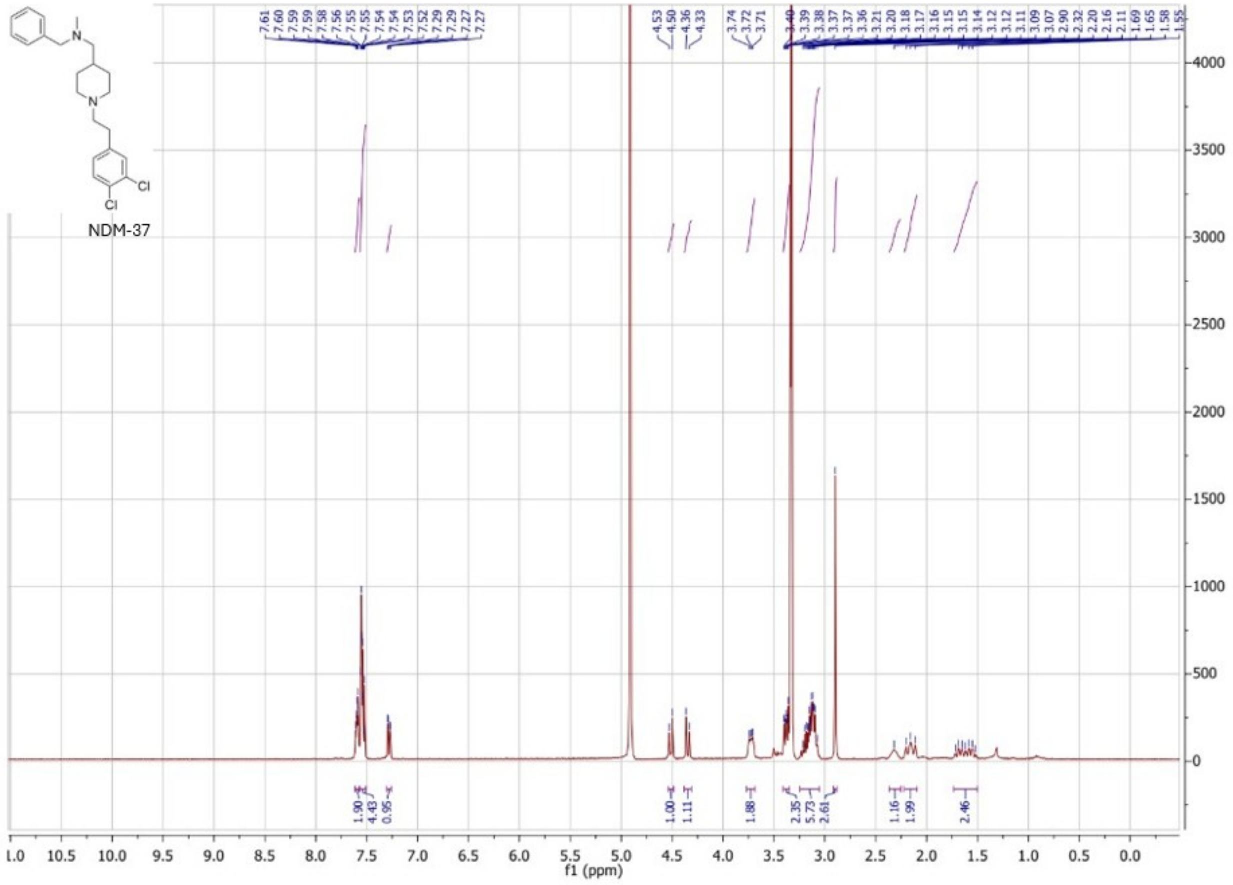

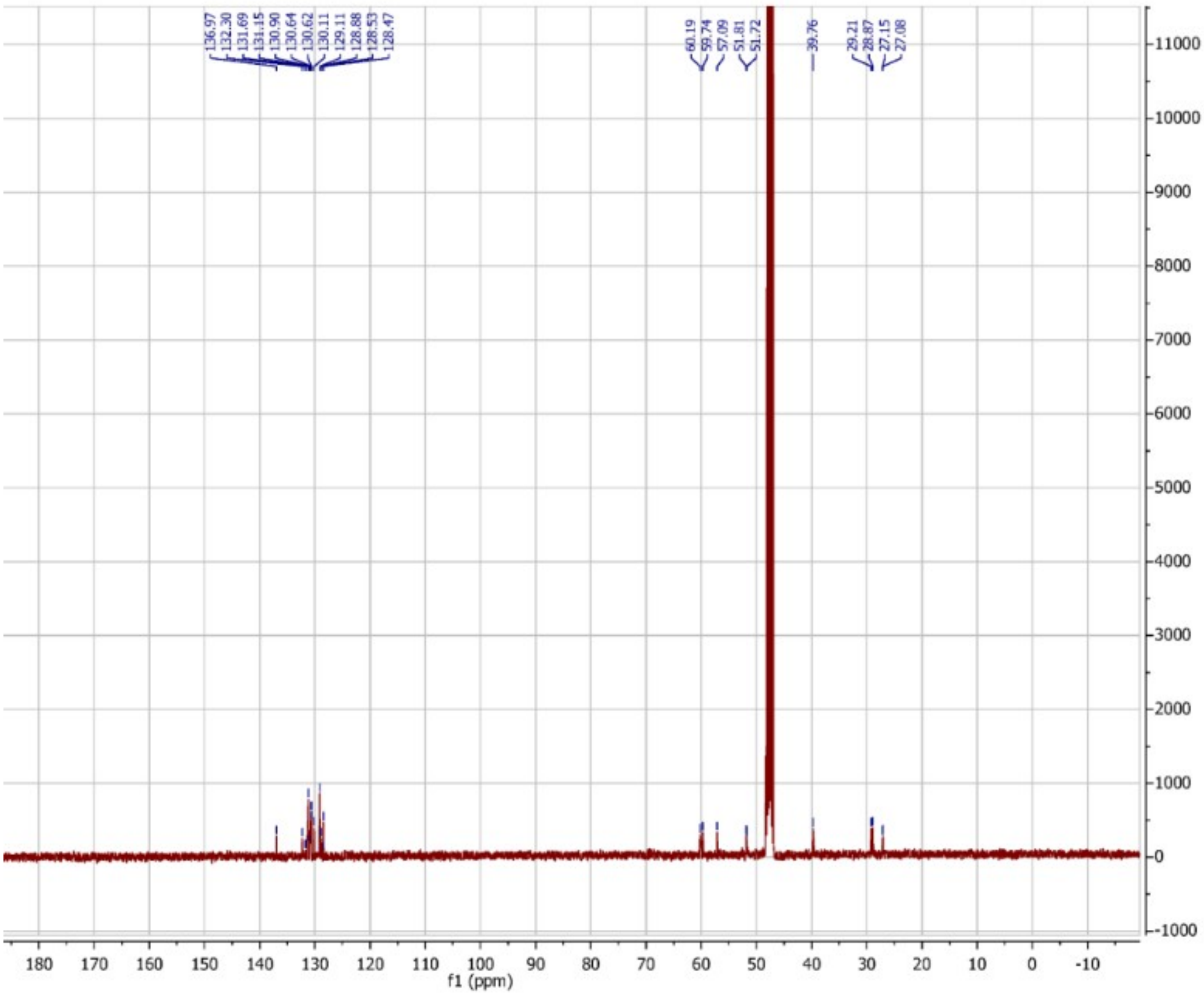

136.97
132.30
131.69
131.15
130.90
130.64
130.62
130.11
129.11
128.88
128.53
128.47
60.19
59.74
57.09
51.81
51.72
39.76
29.21
28.87
27.15
27.08
f1 (ppm)

***N*-(2-Chlorobenzyl)-1-(1-(3,4-dichlorophenethyl)piperidin-4-yl)-*N*-methylmethanamine (NDM-36):**

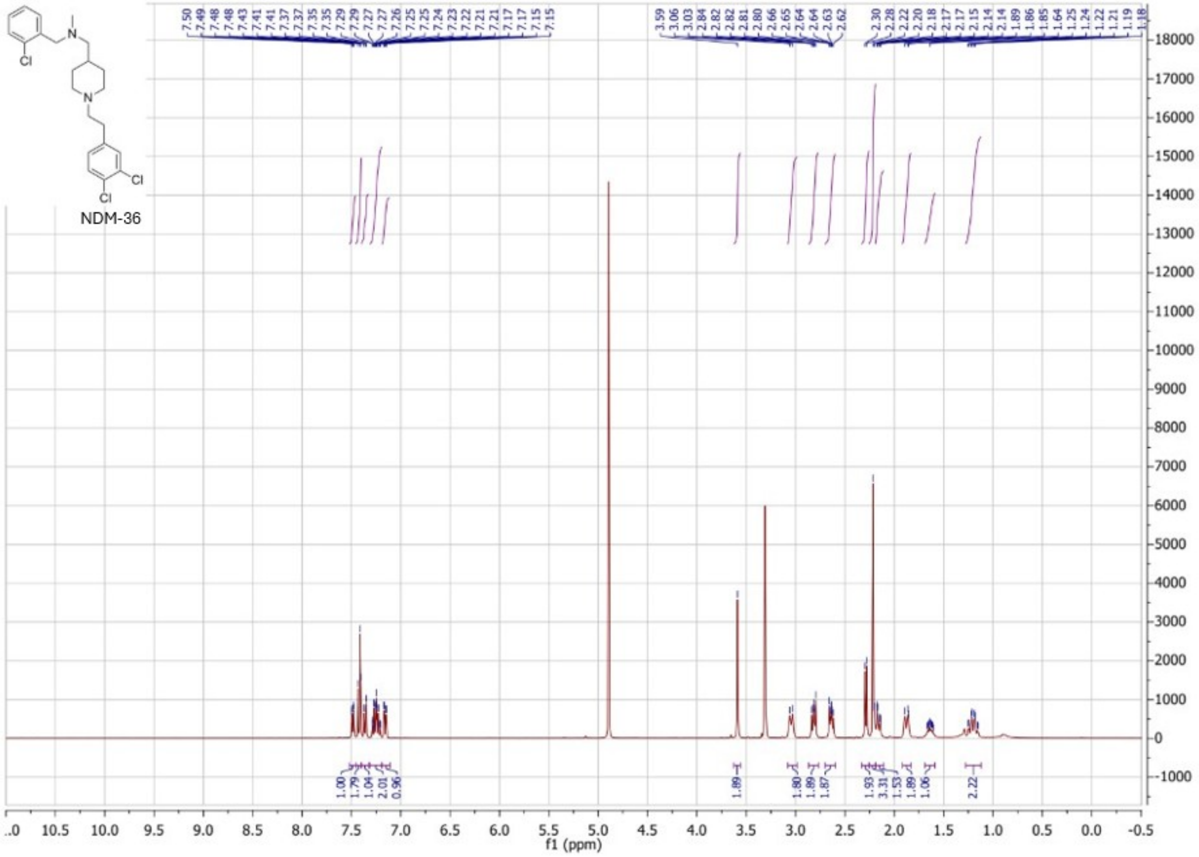

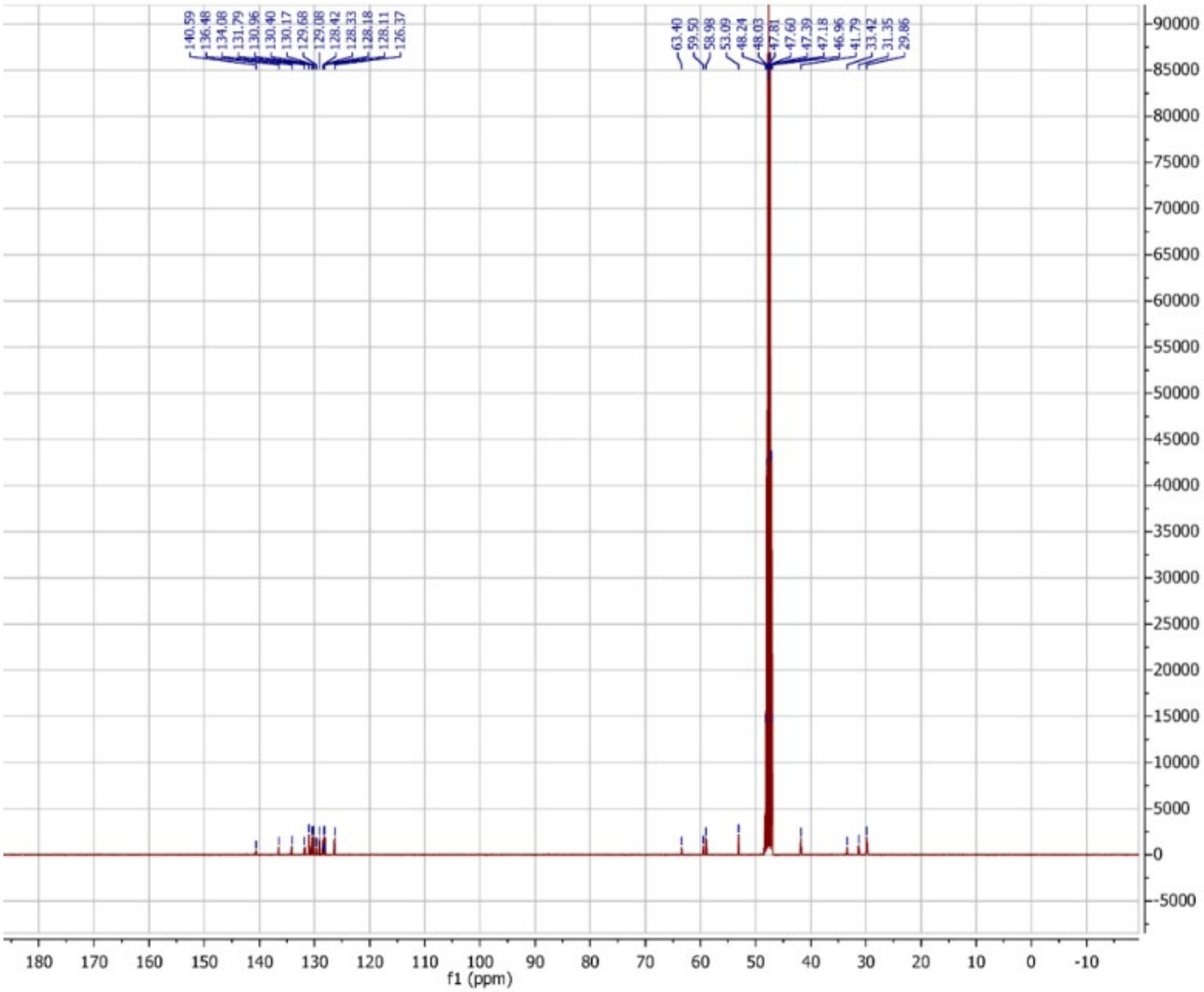
140.59
136.48
134.08
131.79
130.96
130.40
130.17
129.68
129.08
128.42
128.33
128.18
128.11
126.37
63.40
59.50
58.98
53.09
48.24
48.03
47.81
47.60
47.39
47.18
46.96
41.79
33.42
31.35
29.86
f1 (ppm)

1-(1-(3,4-Dichlorophenethyl)piperidin-4-yl)-*N*-methyl-*N*-(2-methylbenzyl)methanamine (NDM-38):

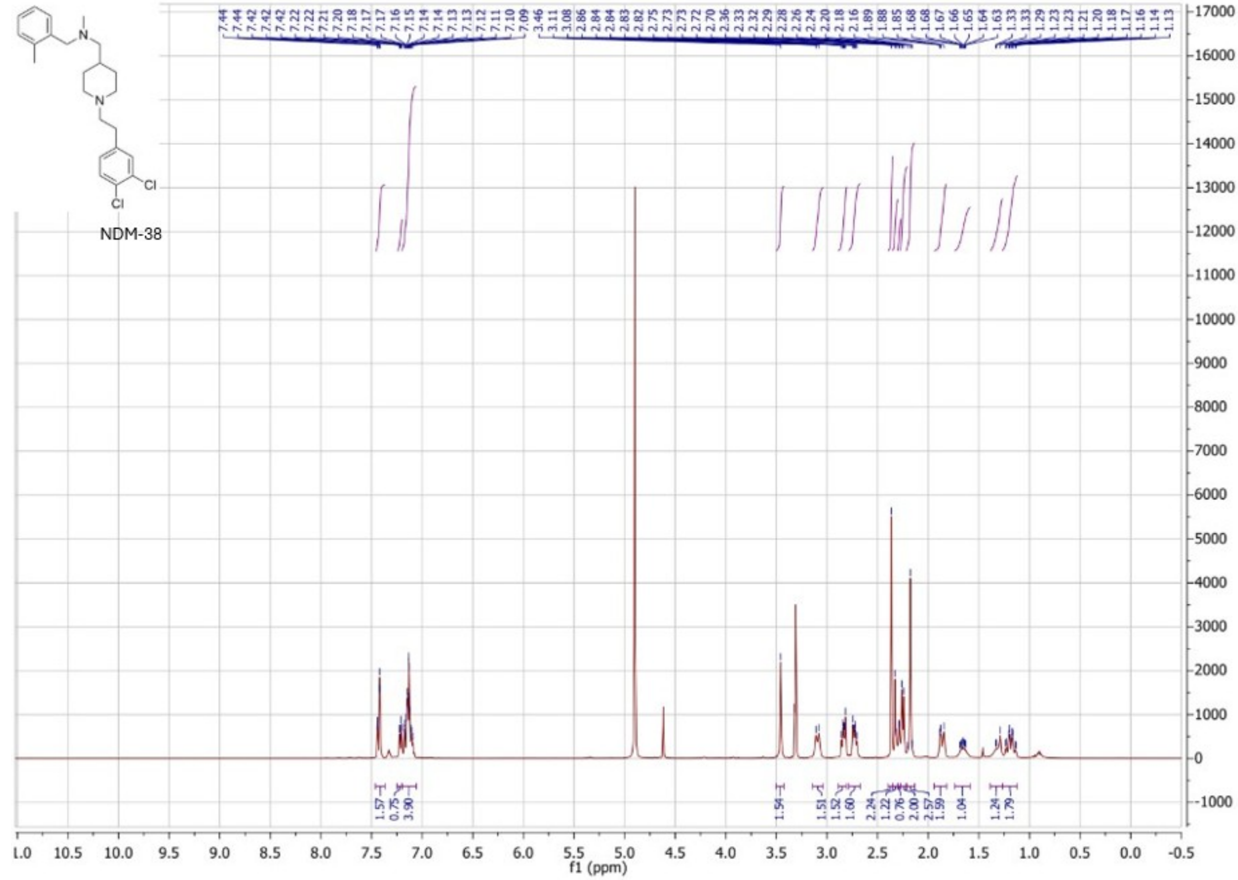

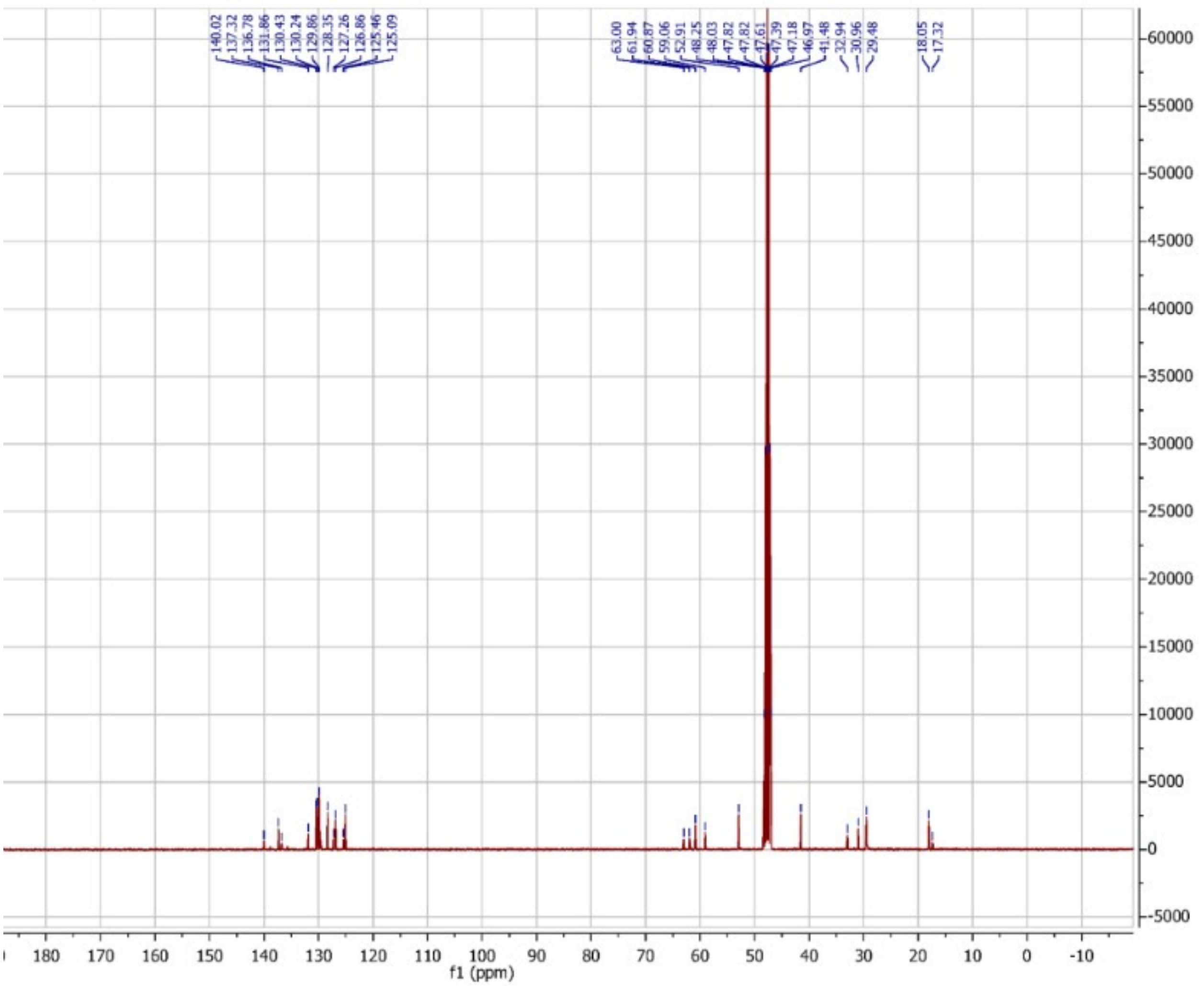
140.02
137.32
136.78
131.86
130.43
130.24
129.86
128.35
127.26
126.86
125.46
125.09
63.00
61.94
60.87
59.06
52.91
48.25
48.03
47.82
47.82
47.61
47.39
47.18
46.97
41.48
32.94
30.96
29.48
18.05
17.32
f1 (ppm)

1-(1-(3,4-Dichlorophenethyl)piperidin-4-yl)-*N*-(3-iodobenzyl)-*N*-methylmethanamine (NDM-35):

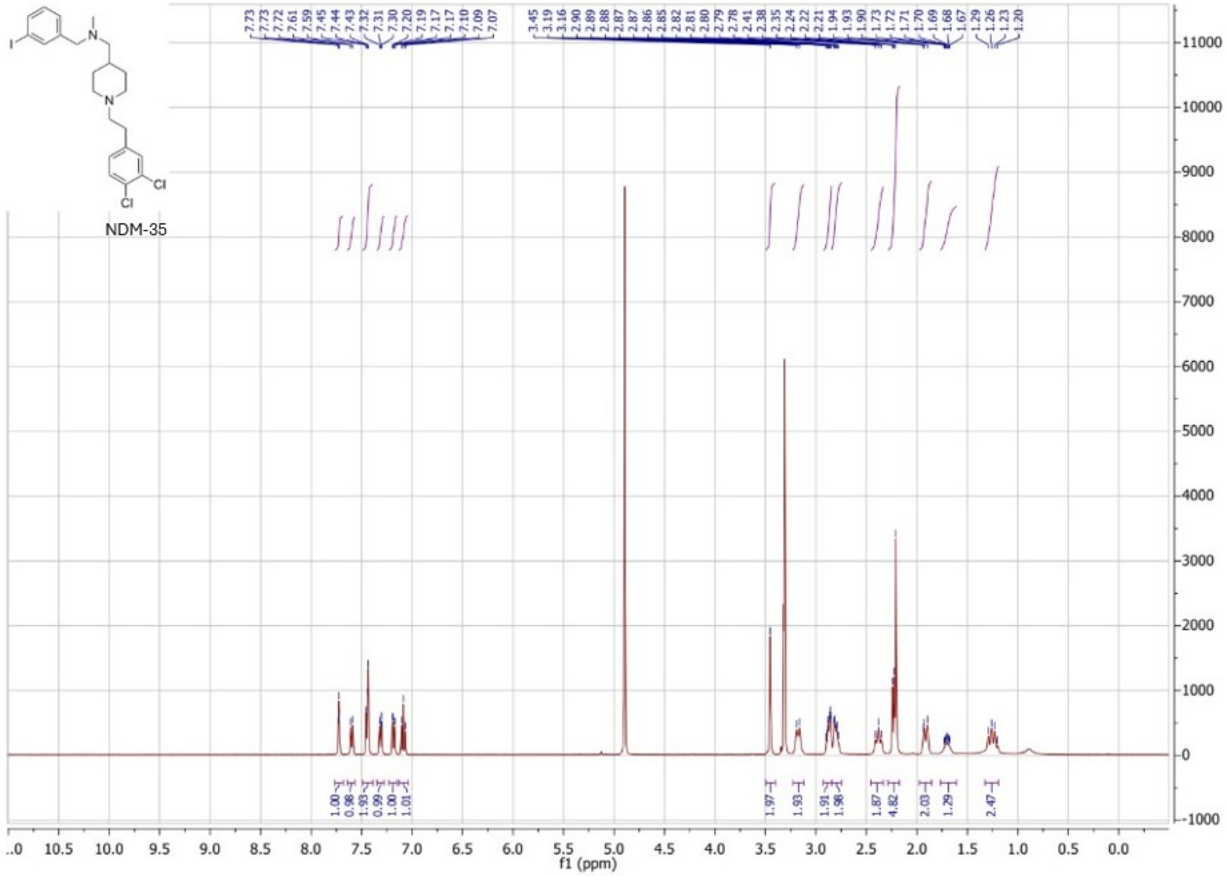

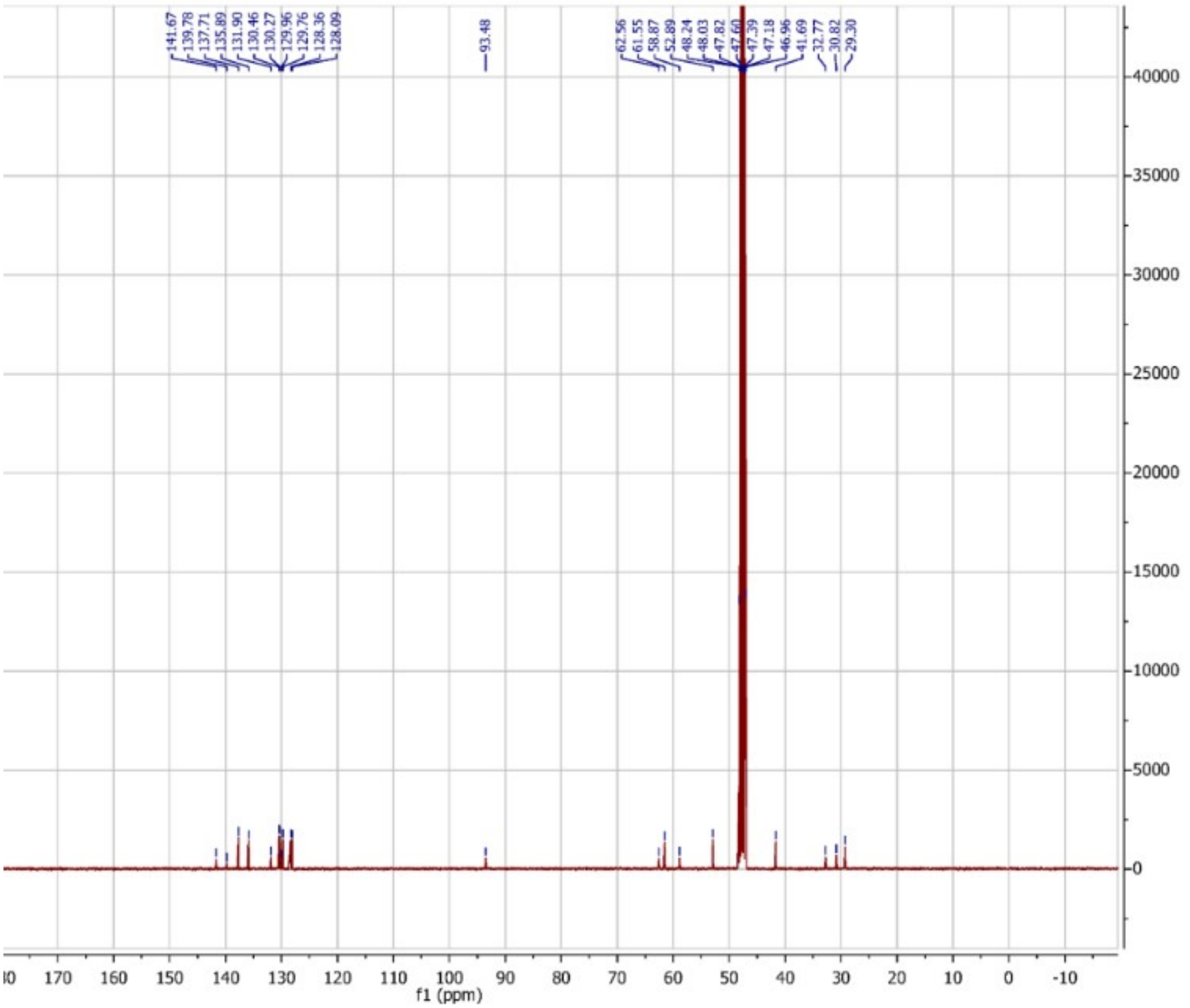

141.67
139.78
137.71
135.89
131.90
130.46
130.27
129.96
129.76
128.36
128.09
93.48
62.56
61.55
58.87
52.89
48.24
48.03
47.82
47.60
47.39
47.18
46.96
41.69
32.77
30.82
29.30
f1 (ppm)

N-(4-Chlorobenzyl)-1-(1-(3,4-dichlorophenethyl)piperidin-4-yl)-N-methylmethanamine (NDM-41):

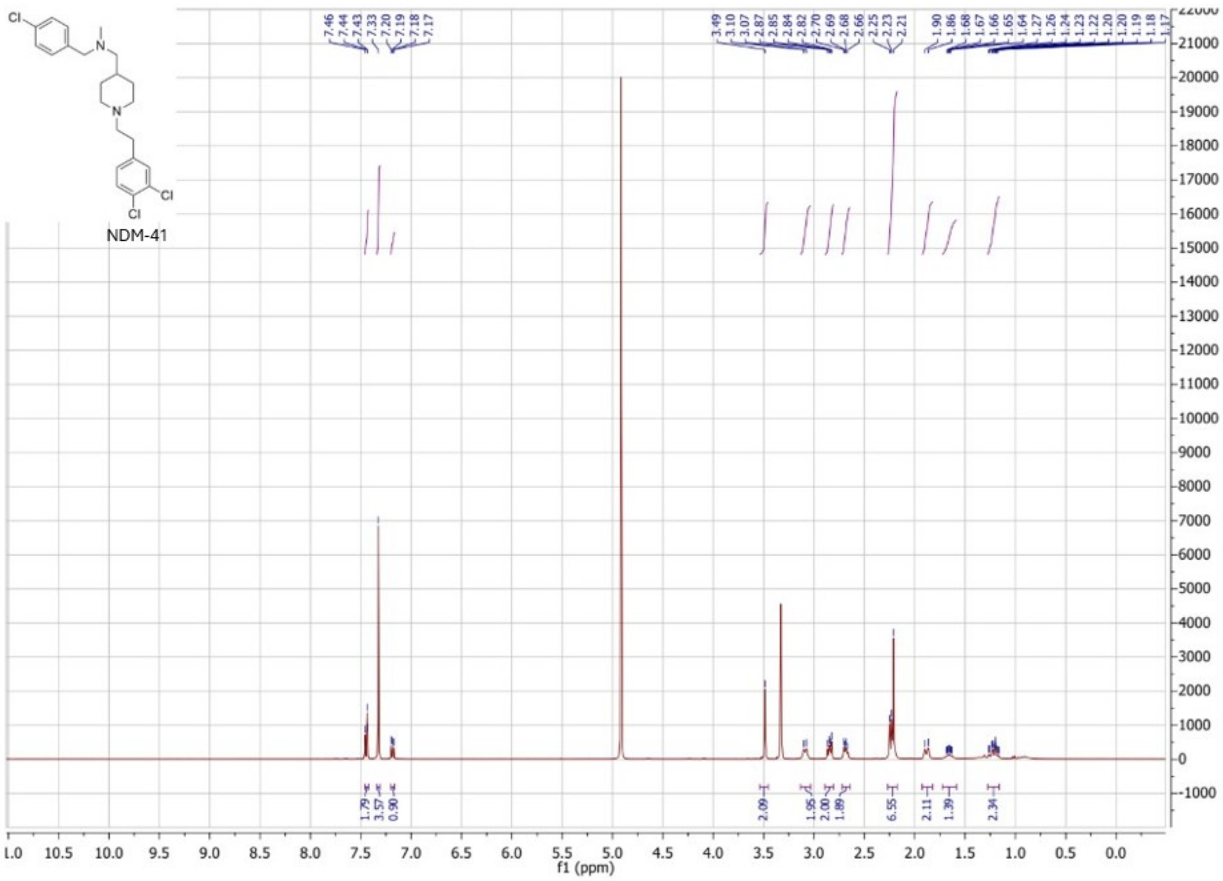

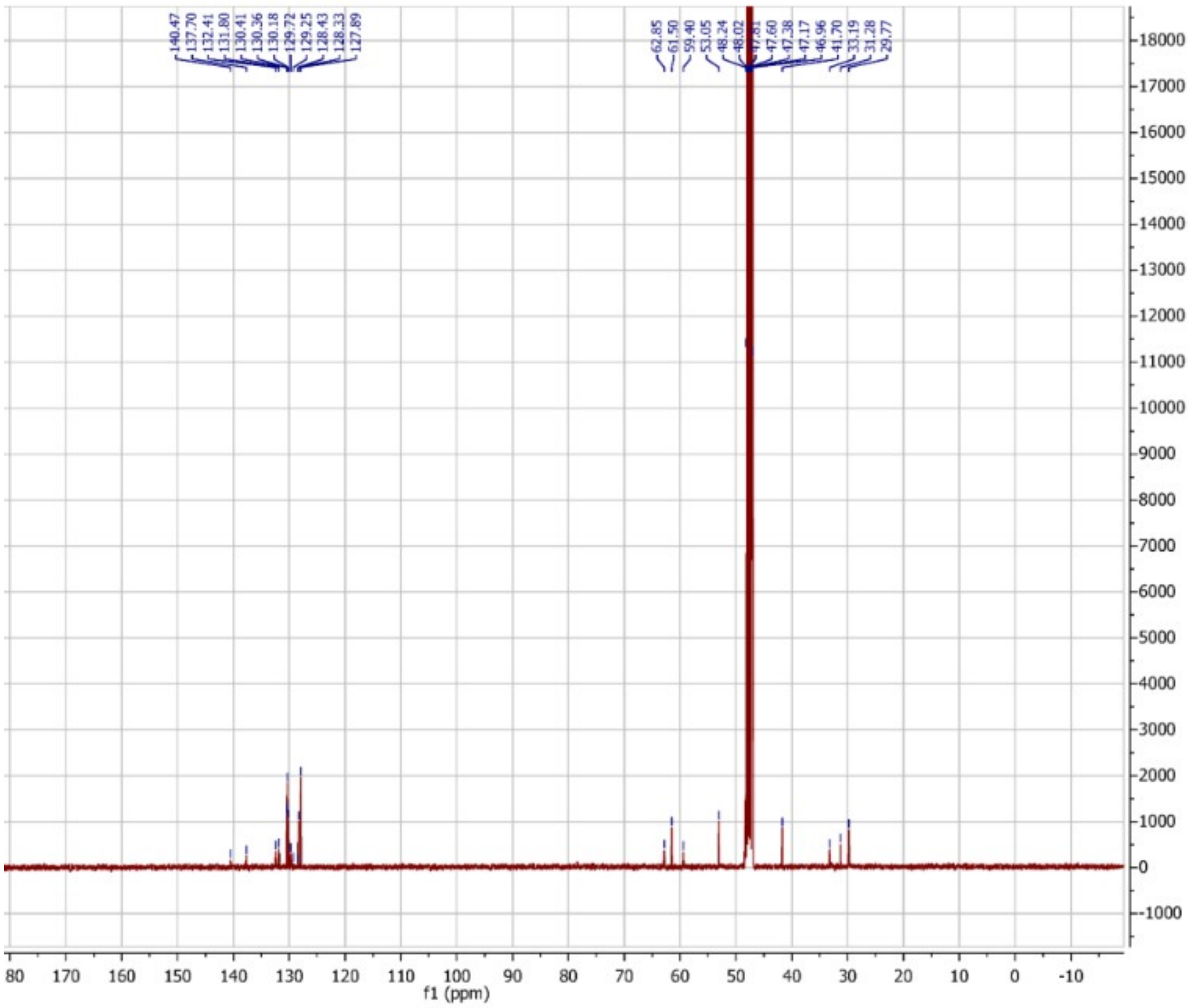

140.47
137.70
132.41
131.80
130.41
130.36
130.18
129.72
129.25
128.43
128.33
127.89
62.85
61.50
59.40
53.05
48.24
48.02
47.81
47.60
47.38
47.17
46.96
41.70
33.19
31.28
29.77
18000
17000
16000
15000
14000
13000
12000
11000
10000
9000
8000
7000
6000
5000
4000
3000
2000
1000
0
-1000
80
170
160
150
140
130
120
110
100
90
80
70
60
50
40
30
20
10
0
-10
f1 (ppm)

***N*-(2,3-Dichlorobenzyl)-1-(1-(3,4-dichlorophenethyl)piperidin-4-yl)-*N*-methylmethanamine (NDM-210):**

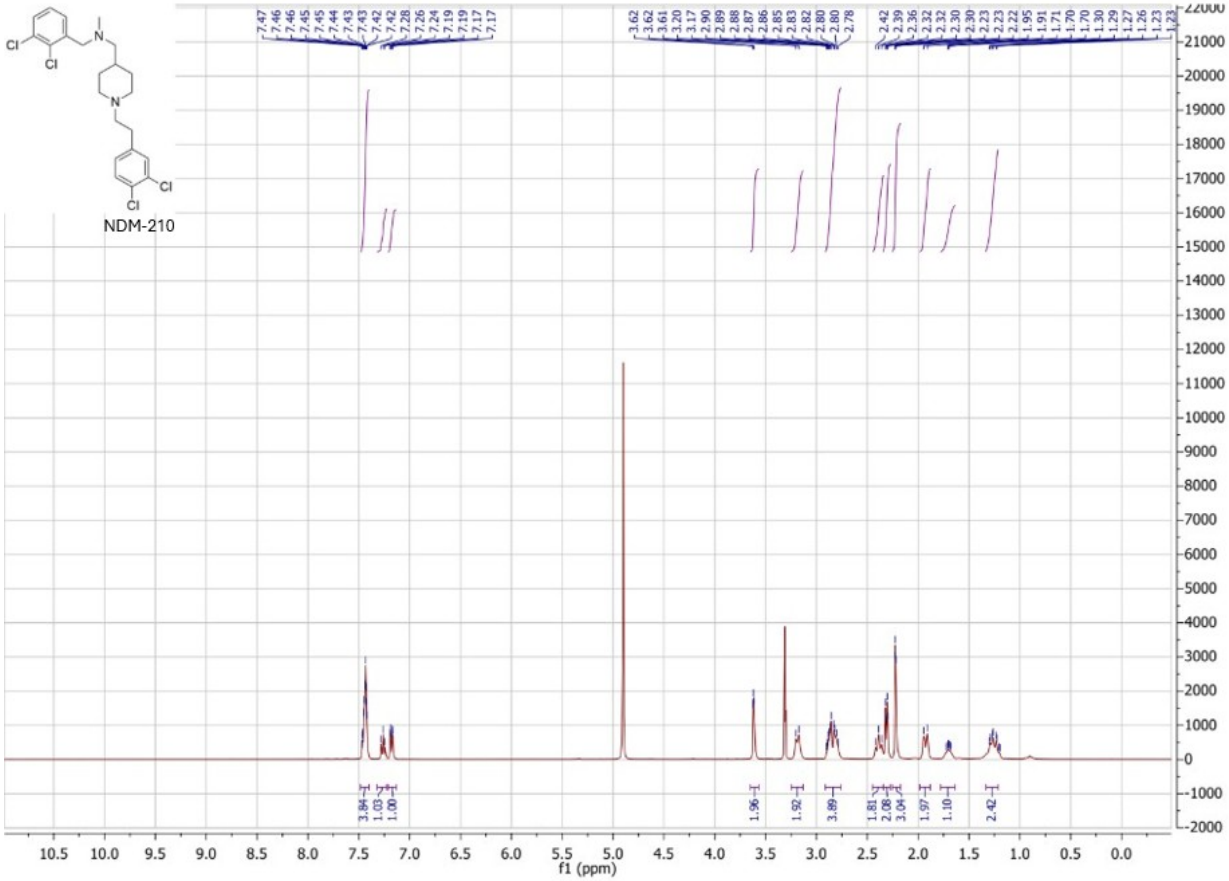

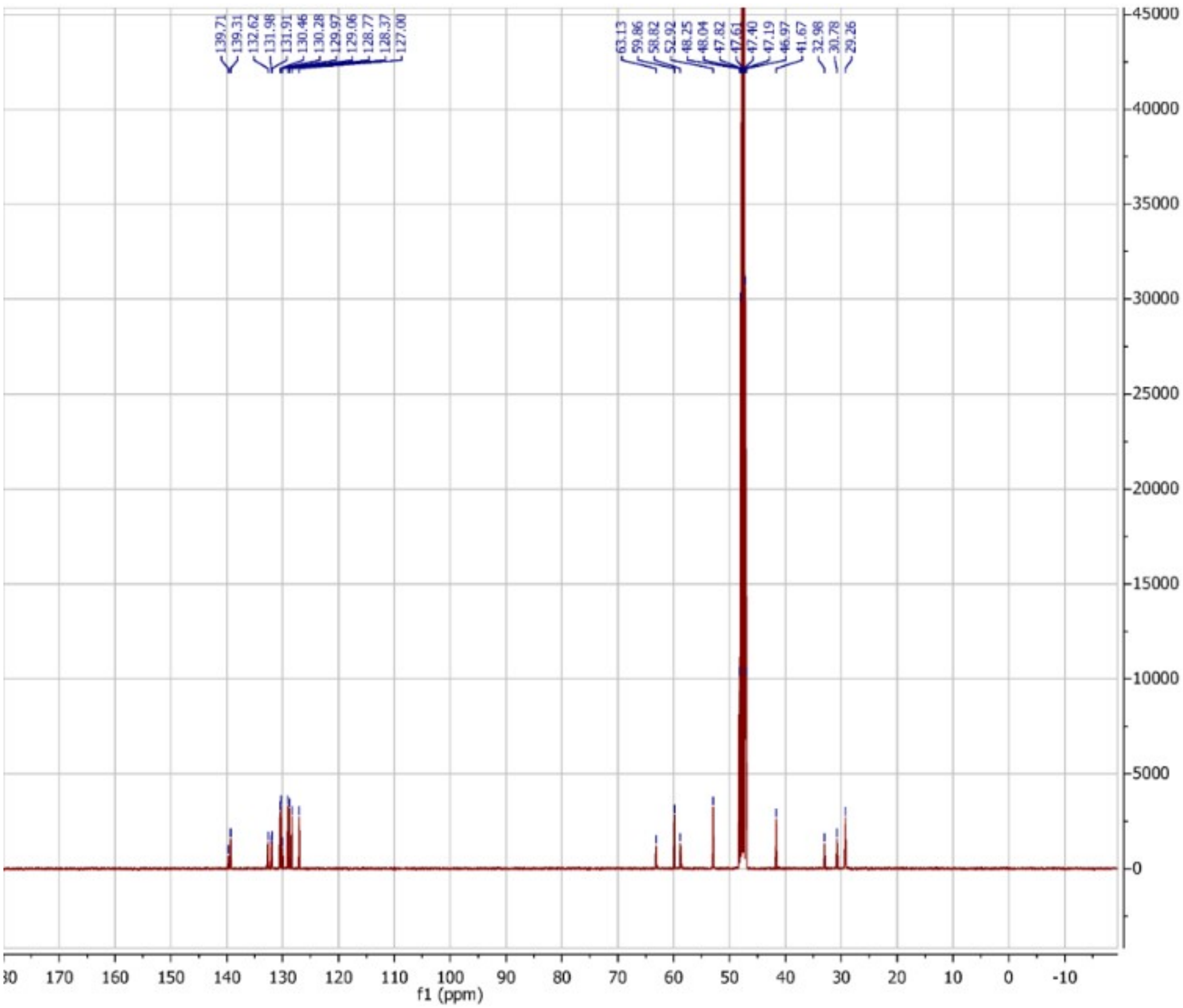
139.71
139.31
132.62
131.98
131.91
130.46
130.28
129.97
129.06
128.77
128.37
127.00
63.13
59.86
58.82
52.92
48.25
48.04
47.82
47.61
47.40
47.19
46.97
41.67
32.98
30.78
29.26
f1 (ppm)

***N*-(3,4-Dichlorobenzyl)-1-(1-(3,4-dichlorophenethyl)piperidin-4-yl)-*N*-methylmethanamine (NDM-40):**

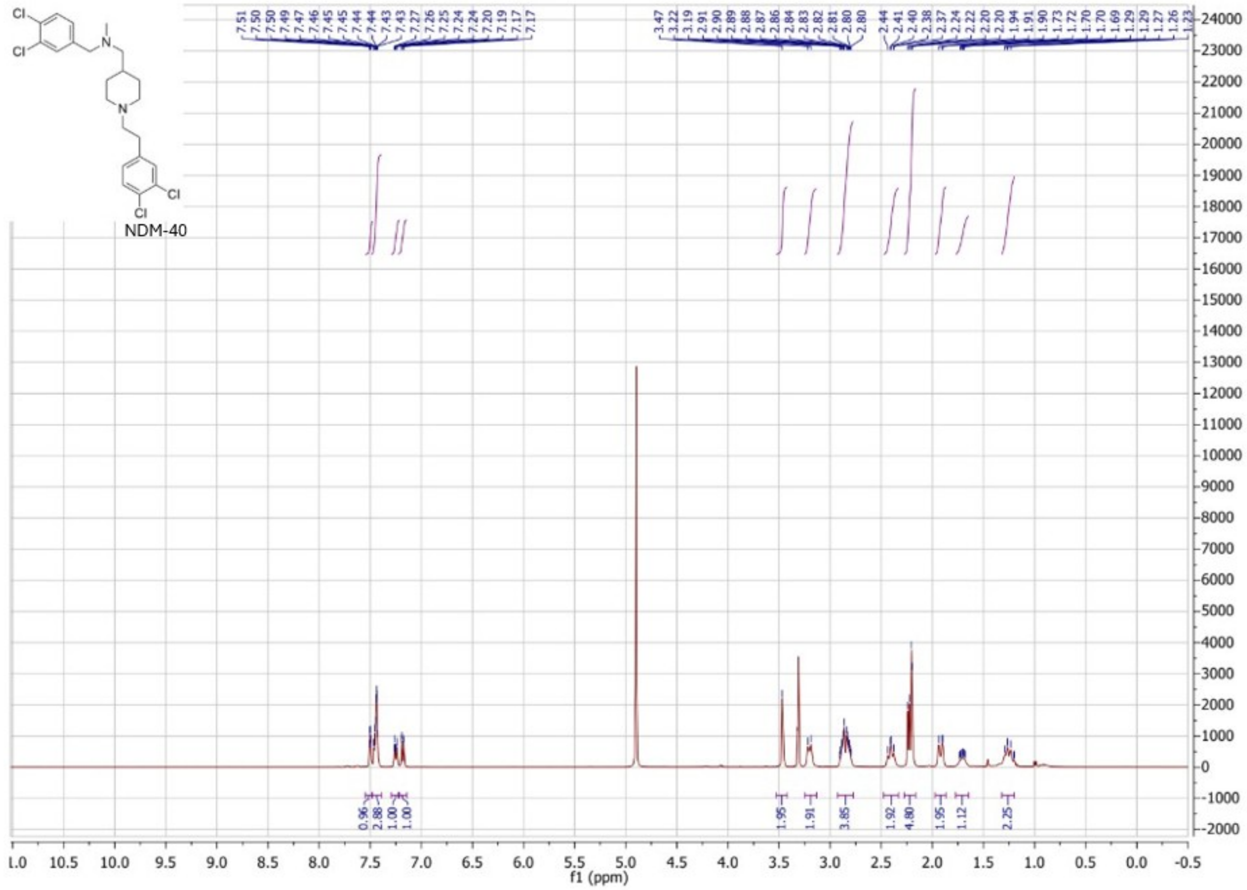

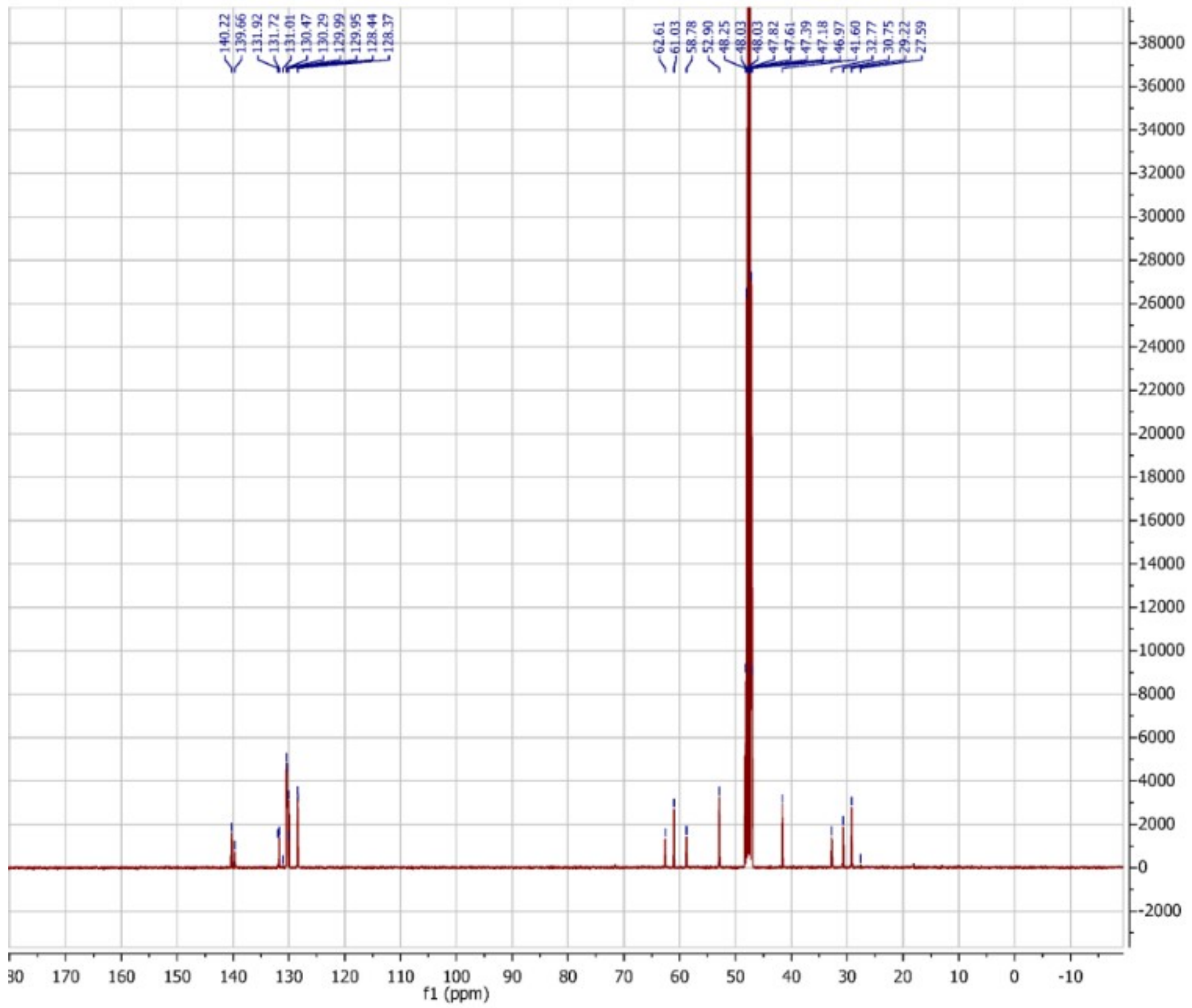

140.22
139.66
131.92
131.72
131.01
130.47
130.29
129.99
129.95
128.44
128.37
62.61
61.03
58.78
52.90
48.25
48.03
48.03
47.82
47.61
47.39
47.18
46.97
41.60
32.77
30.75
29.22
27.59
f1 (ppm)

1-(1-(2-([1,1'-Biphenyl]-4-yl)ethyl)piperidin-4-yl)-*N*-methyl-*N*-(thiophen-2-ylmethyl)methanamine (NDM-592):

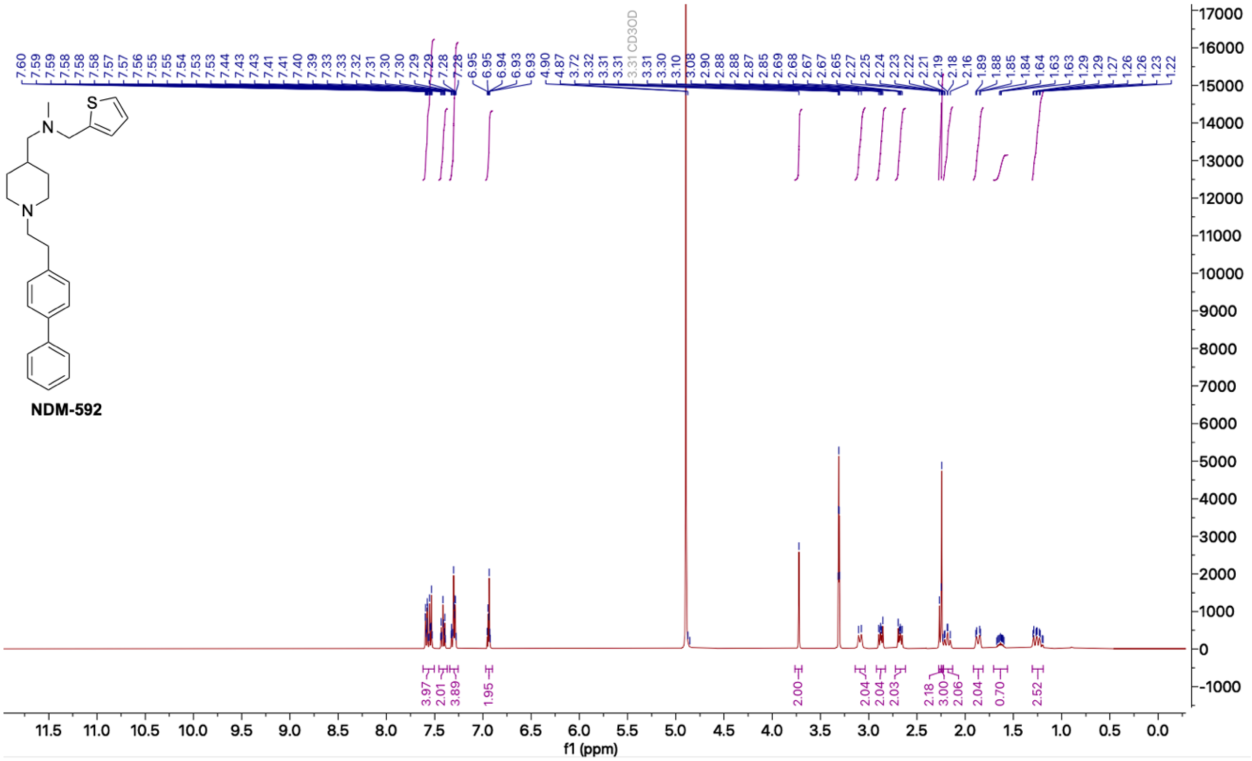

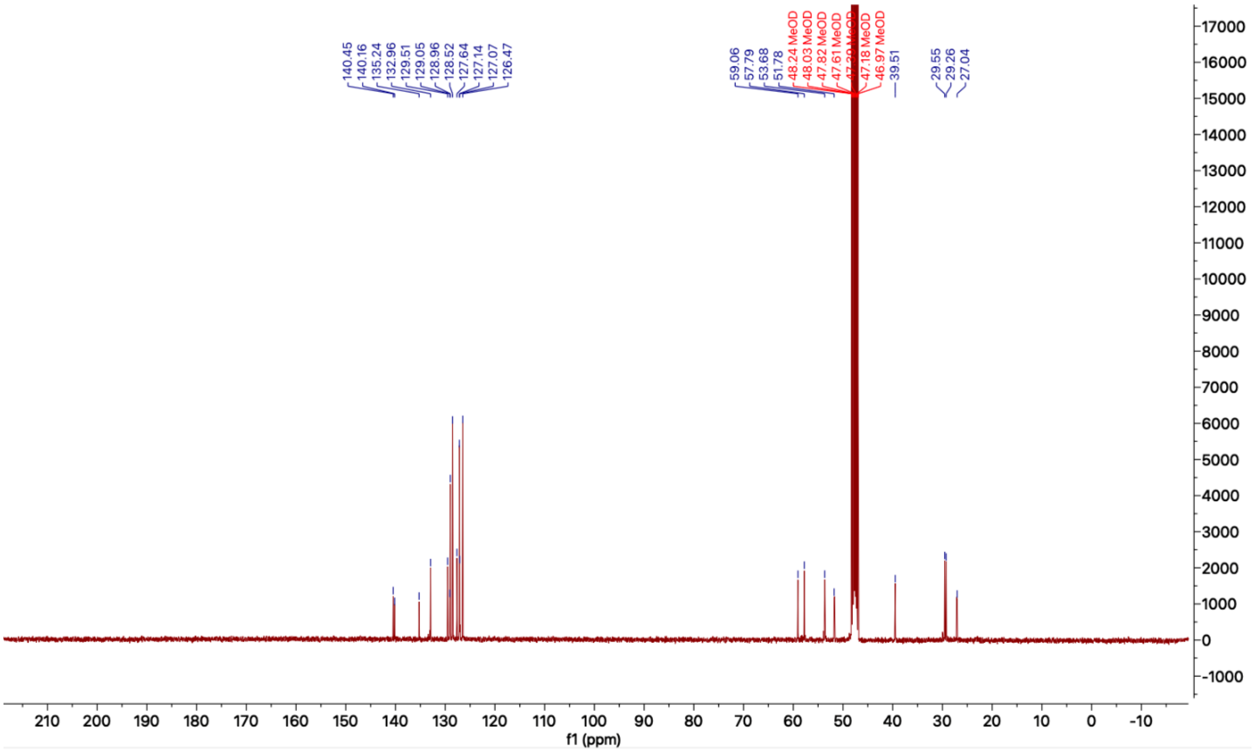
140.45
140.16
135.24
132.96
129.51
129.05
128.96
128.52
127.64
127.14
127.07
126.47
59.06
57.79
53.68
51.78
48.24 MeOD
48.03 MeOD
47.82 MeOD
47.61 MeOD
47.18 MeOD
46.97 MeOD
39.51
29.55
29.26
27.04
f1 (ppm)

1-(1-(2-([1,1'-Biphenyl]-4-yl)ethyl)piperidin-4-yl)-*N*-benzyl-*N*-methylmethanamine (NDM-591):

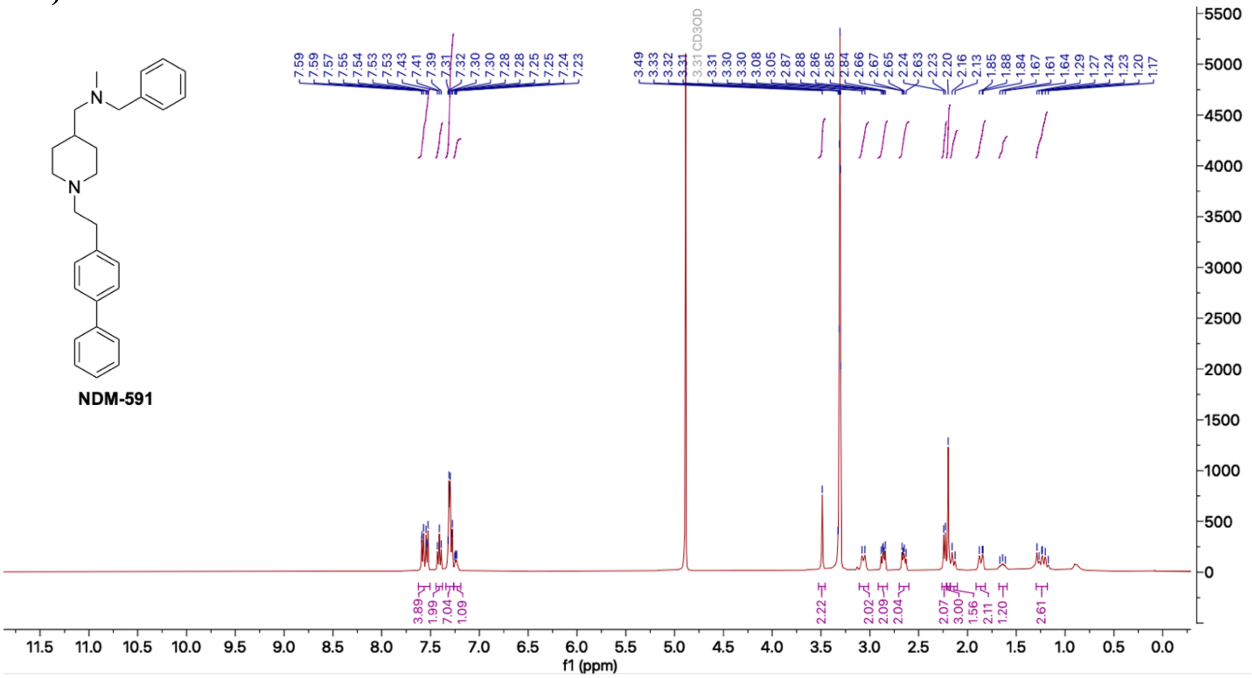

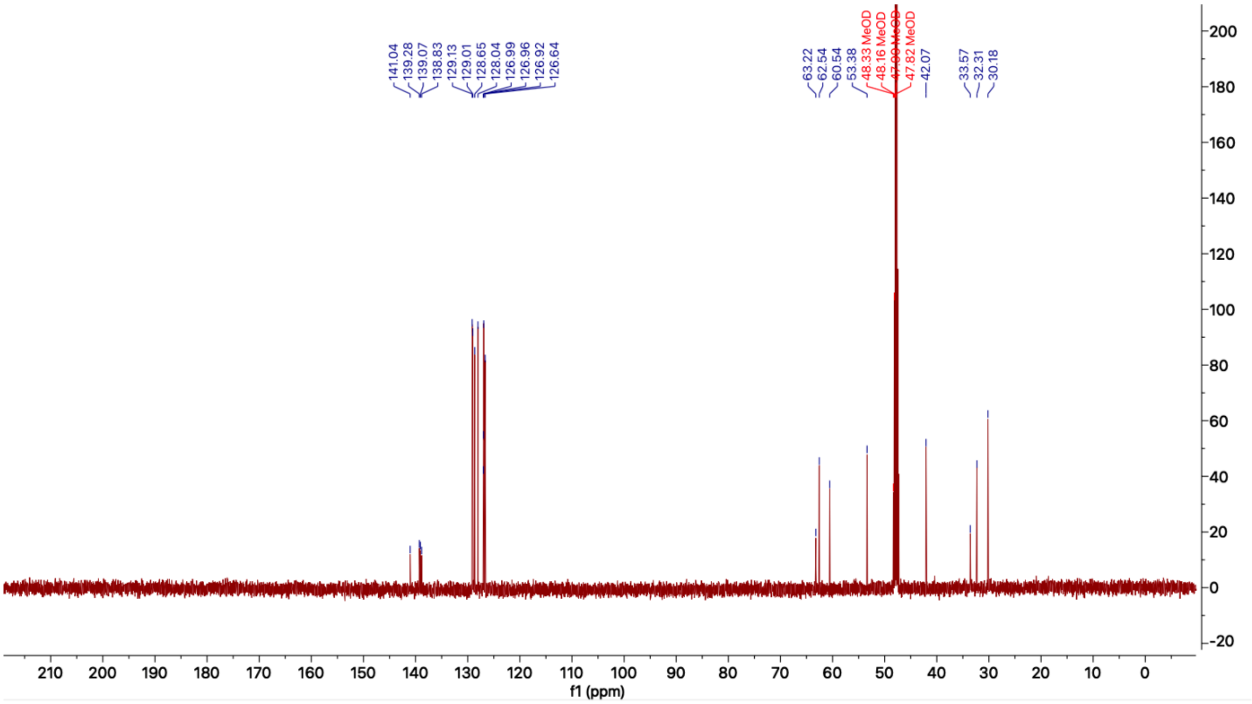
141.04
139.28
139.07
138.83
129.13
129.01
128.65
128.04
126.99
126.96
126.92
126.64
63.22
62.54
60.54
53.38
48.33 MeOD
48.16 MeOD
47.99 MeOD
47.82 MeOD
42.07
33.57
32.31
30.18
f1 (ppm)

1-(1-(2-([1,1'-Biphenyl]-4-yl)ethyl)piperidin-4-yl)-*N*-methyl-*N*-(3-methylbenzyl)methanamine (NDM-604):

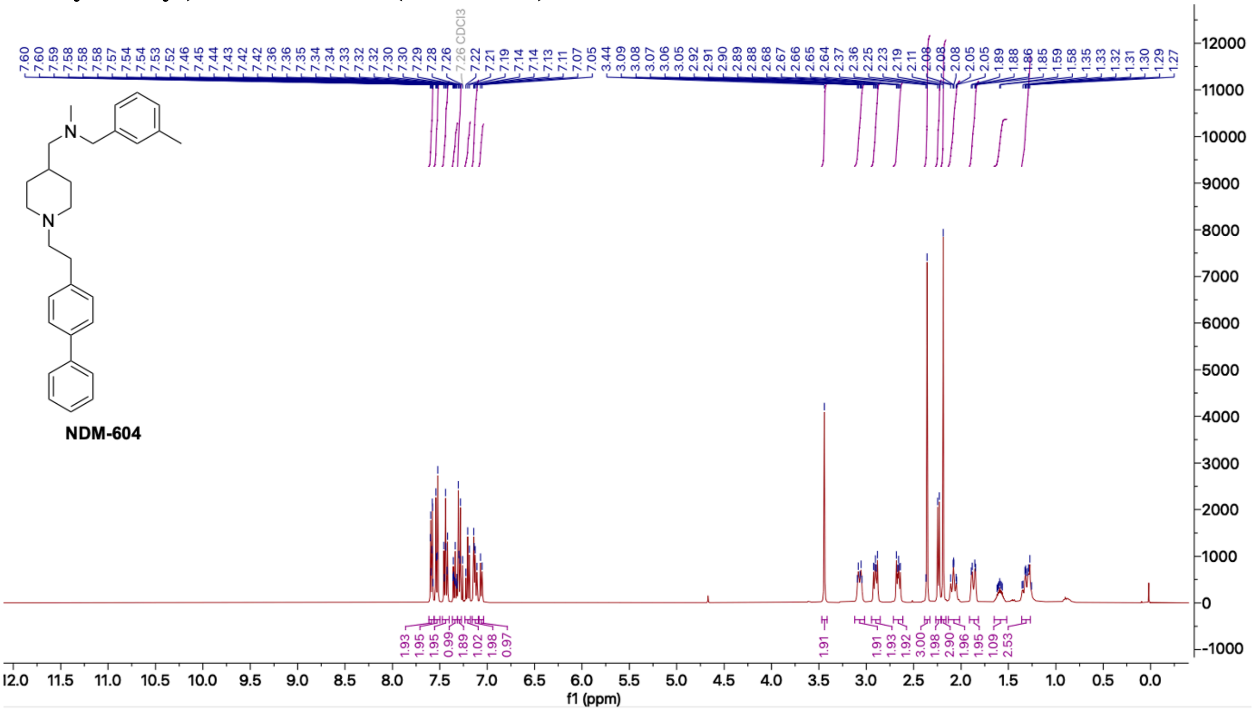

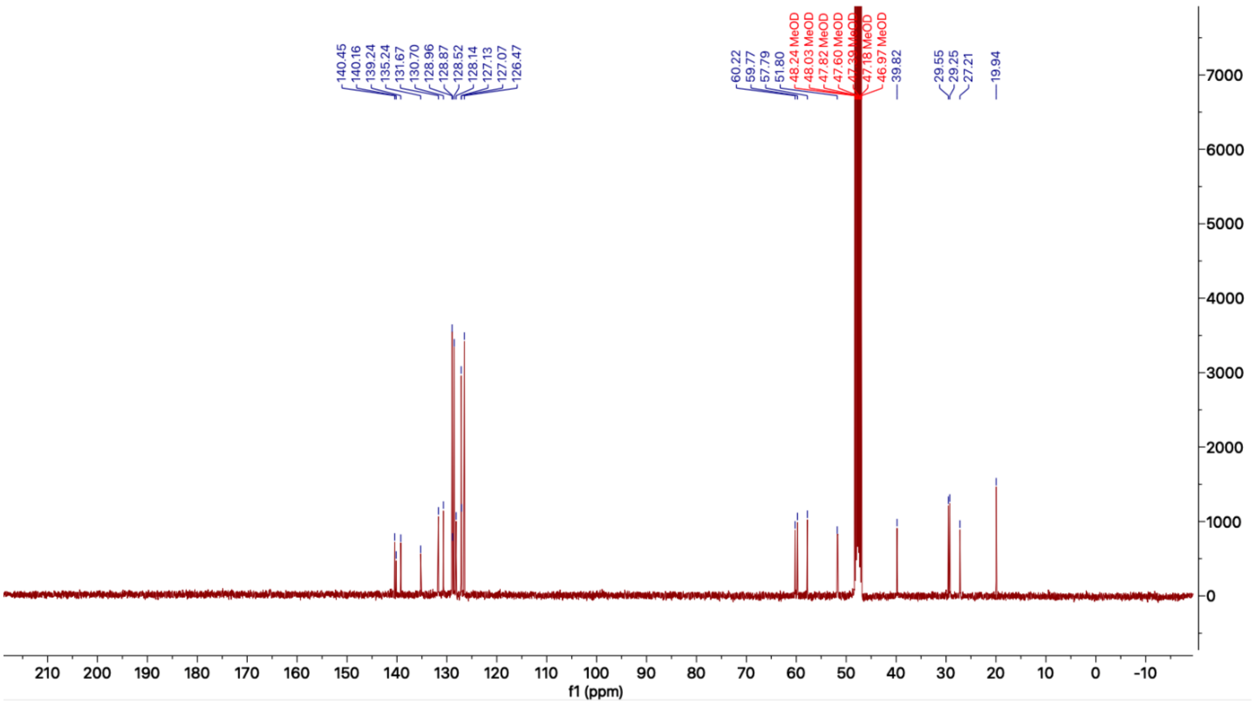

140.45
140.16
139.24
135.24
131.67
130.70
128.96
128.87
128.52
128.14
127.13
127.07
126.47
60.22
59.77
57.79
51.80
48.24 MeOD
48.03 MeOD
47.82 MeOD
47.60 MeOD
47.39 MeOD
47.18 MeOD
46.97 MeOD
39.82
29.55
29.25
27.21
19.94
f1 (ppm)

1-(1-(2-([1,1'-Biphenyl]-4-yl)ethyl)piperidin-4-yl)-*N*-(4-fluorobenzyl)-*N*-methylmethanamine (NDM-606):

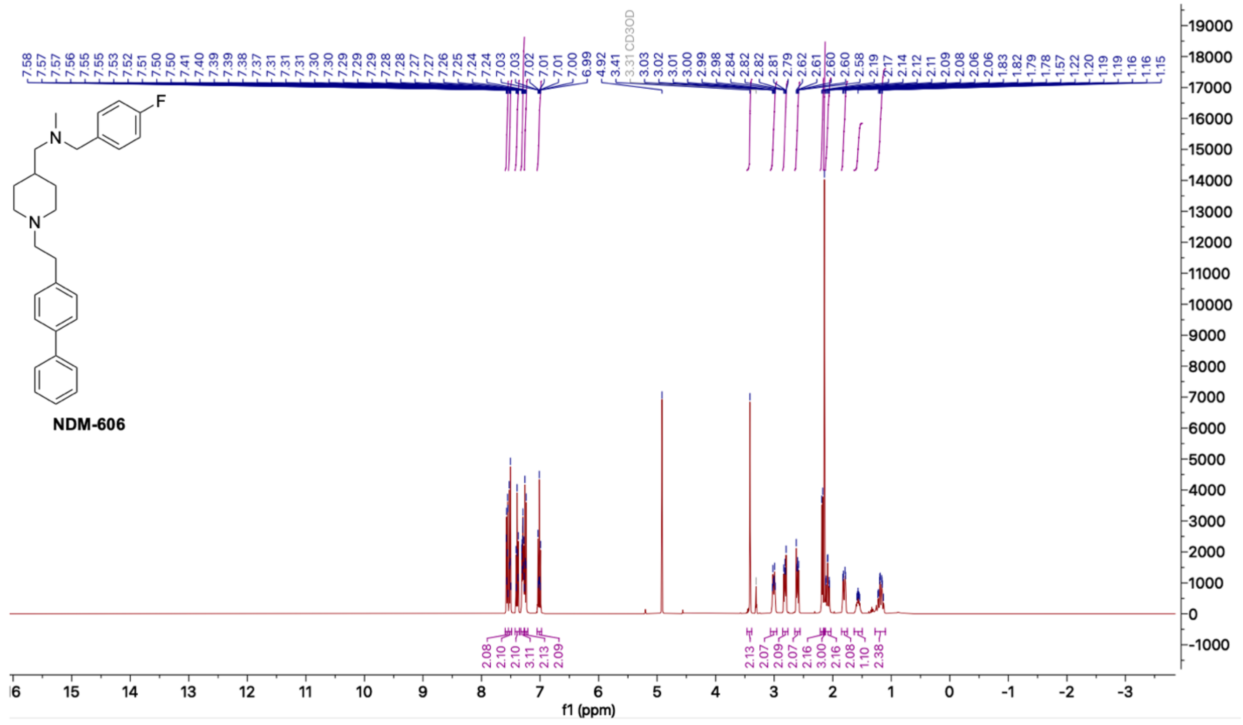

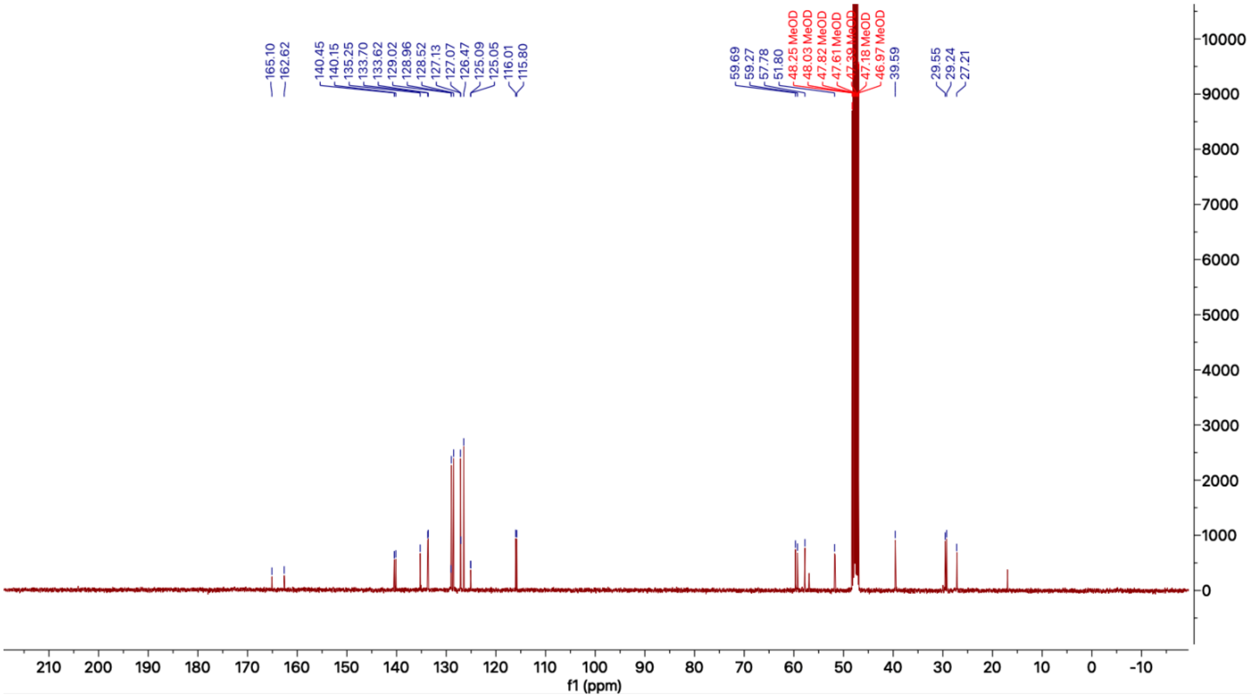
165.10
162.62
140.45
140.15
135.25
133.70
133.62
129.02
128.96
128.52
127.13
127.07
126.47
125.09
125.05
116.01
115.80
59.69
59.27
57.78
51.80
48.25 MeOD
48.03 MeOD
47.82 MeOD
47.61 MeOD
47.39 MeOD
47.18 MeOD
46.97 MeOD
39.59
29.55
29.24
27.21
f1 (ppm)

1-(1-(2-([1,1'-Biphenyl]-4-yl)ethyl)piperidin-4-yl)-*N*-(4-chlorobenzyl)-*N*-methylmethanamine (NDM-565):

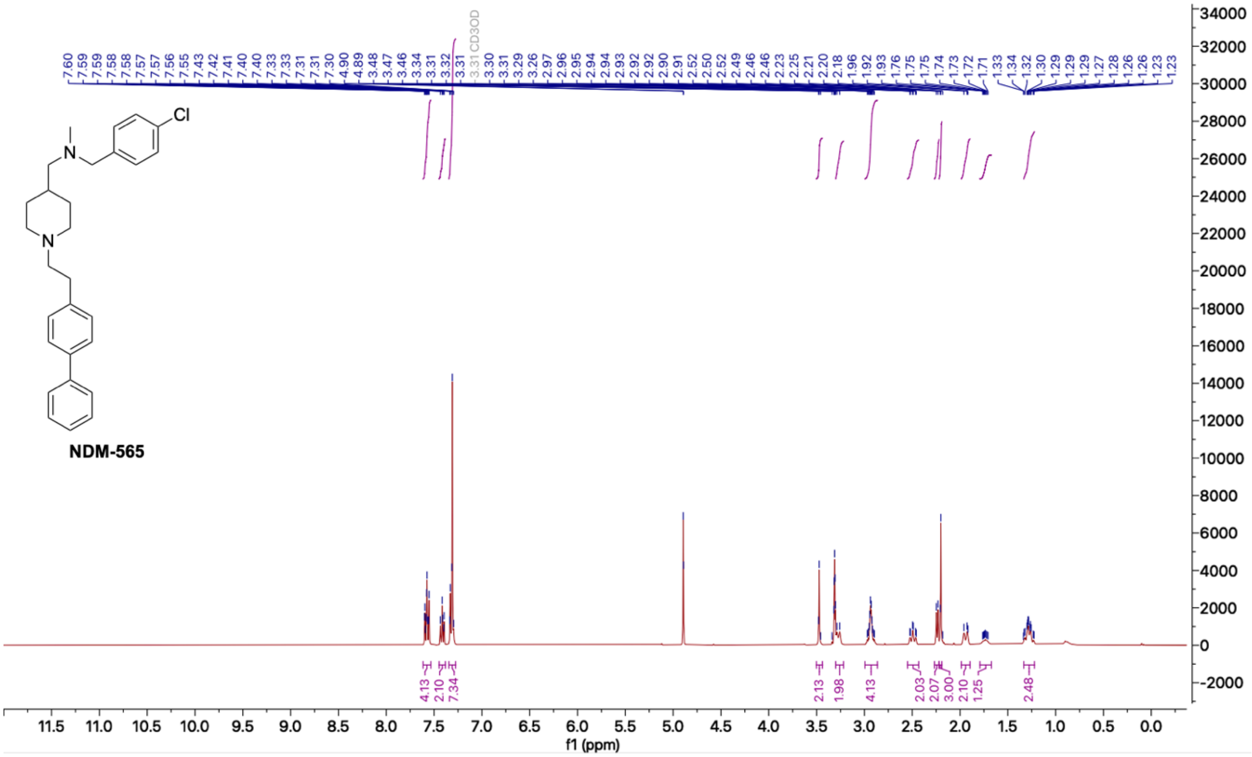

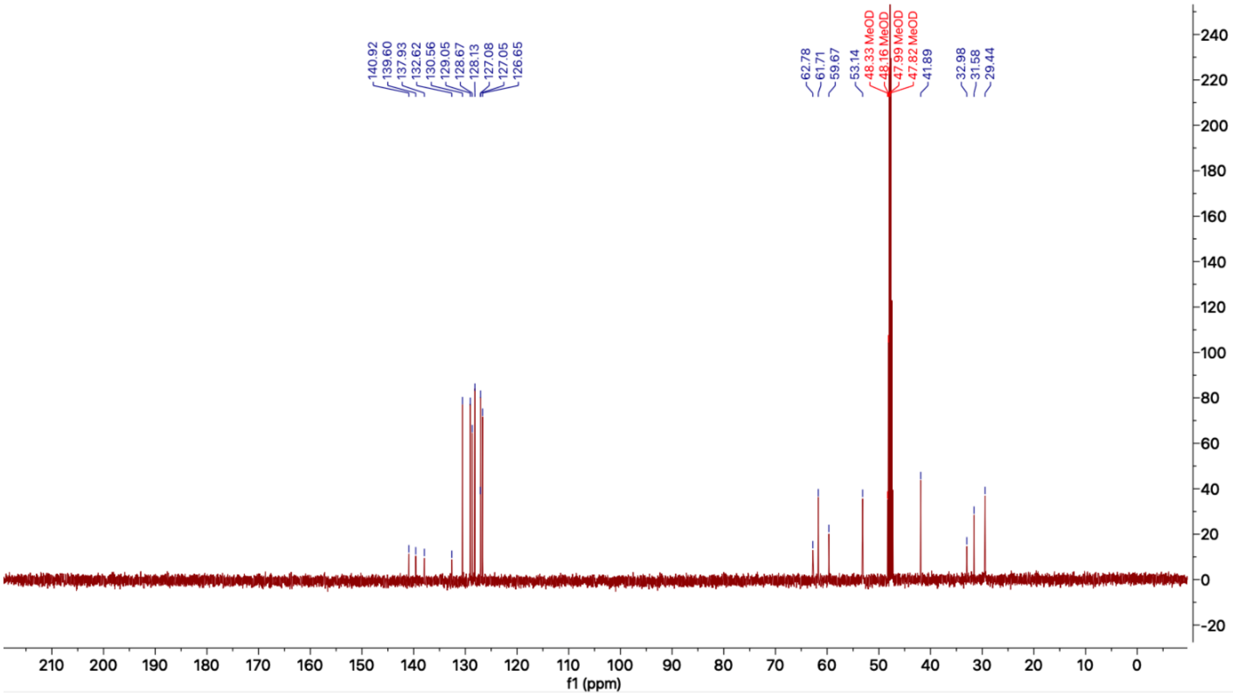

140.92
139.60
137.93
132.62
130.56
129.05
128.67
128.13
127.08
127.05
126.65
62.78
61.71
59.67
53.14
48.33 MeOD
48.16 MeOD
47.99 MeOD
47.82 MeOD
41.89
32.98
31.58
29.44
f1 (ppm)

1-(1-(2-([1,1'-Biphenyl]-4-yl)ethyl)piperidin-4-yl)-*N*-(2,3-dichlorobenzyl)-*N*-methylmethanamine (NDM-594):

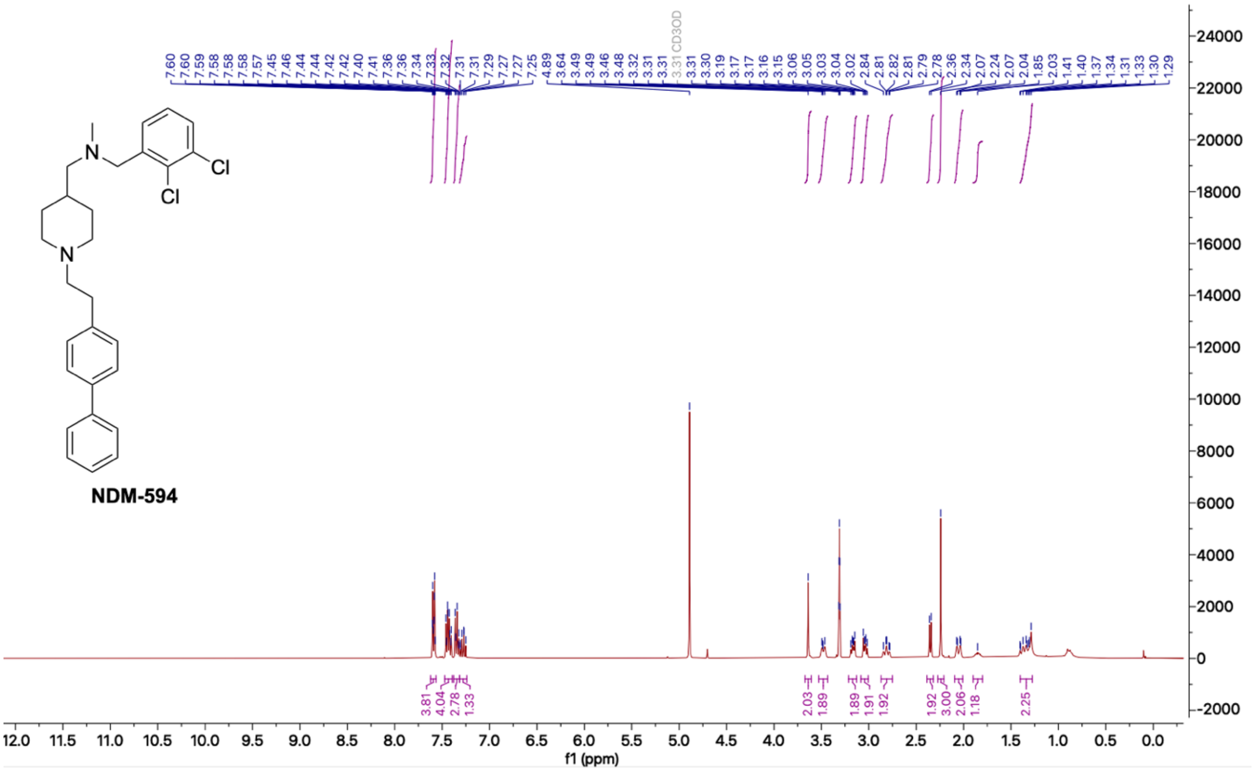

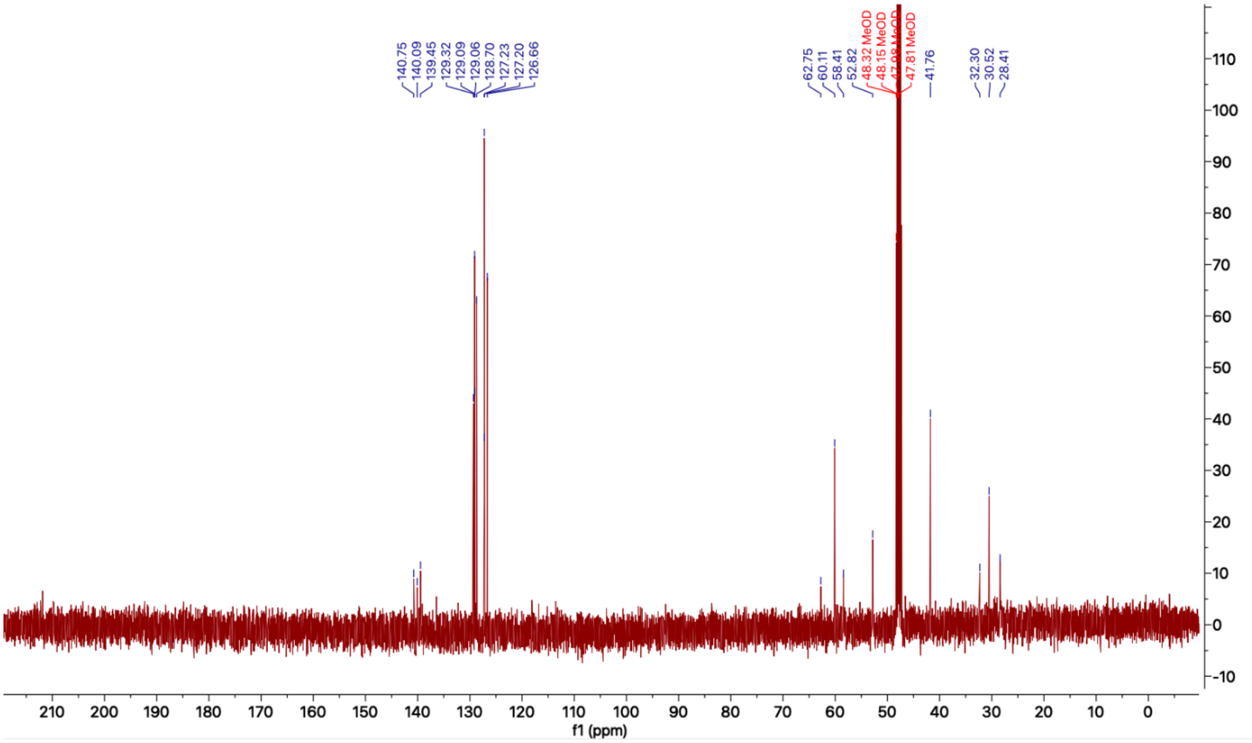

140.75
140.09
139.45
129.32
129.09
129.06
128.70
127.23
127.20
126.66
62.75
60.11
58.41
52.82
48.32 MeOD
48.15 MeOD
47.81 MeOD
41.76
32.30
30.52
28.41
f1 (ppm)

1-(1-(2-([1,1'-Biphenyl]-4-yl)ethyl)piperidin-4-yl)-*N*-(3,4-dichlorobenzyl)-*N*-methylmethanamine (NDM-605):

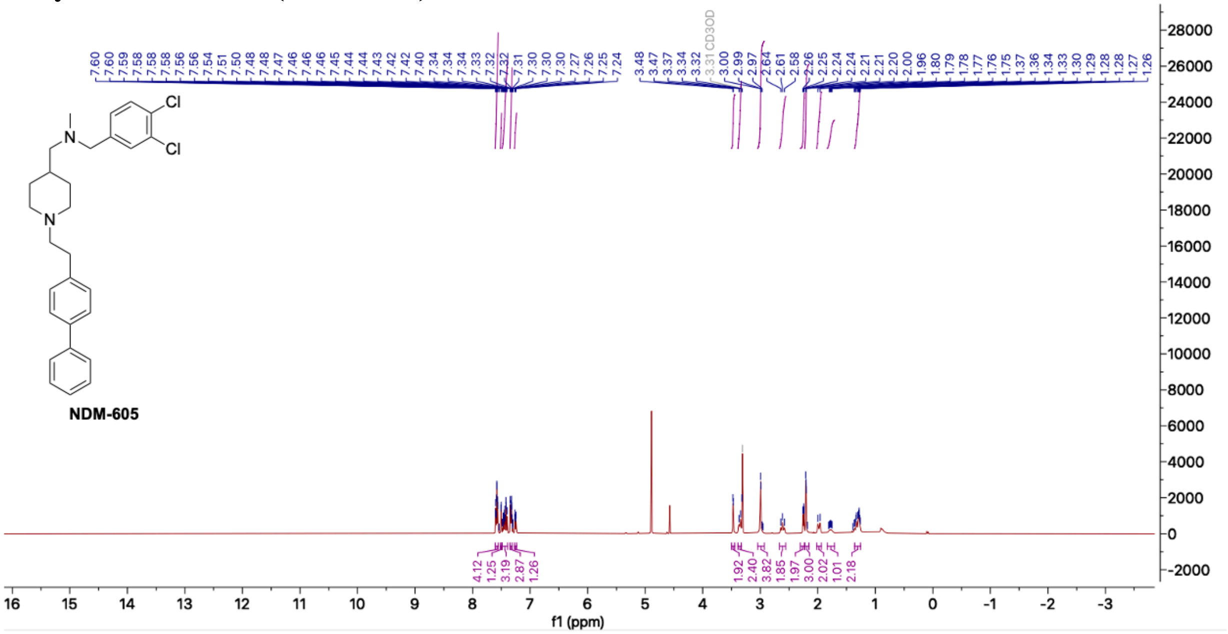

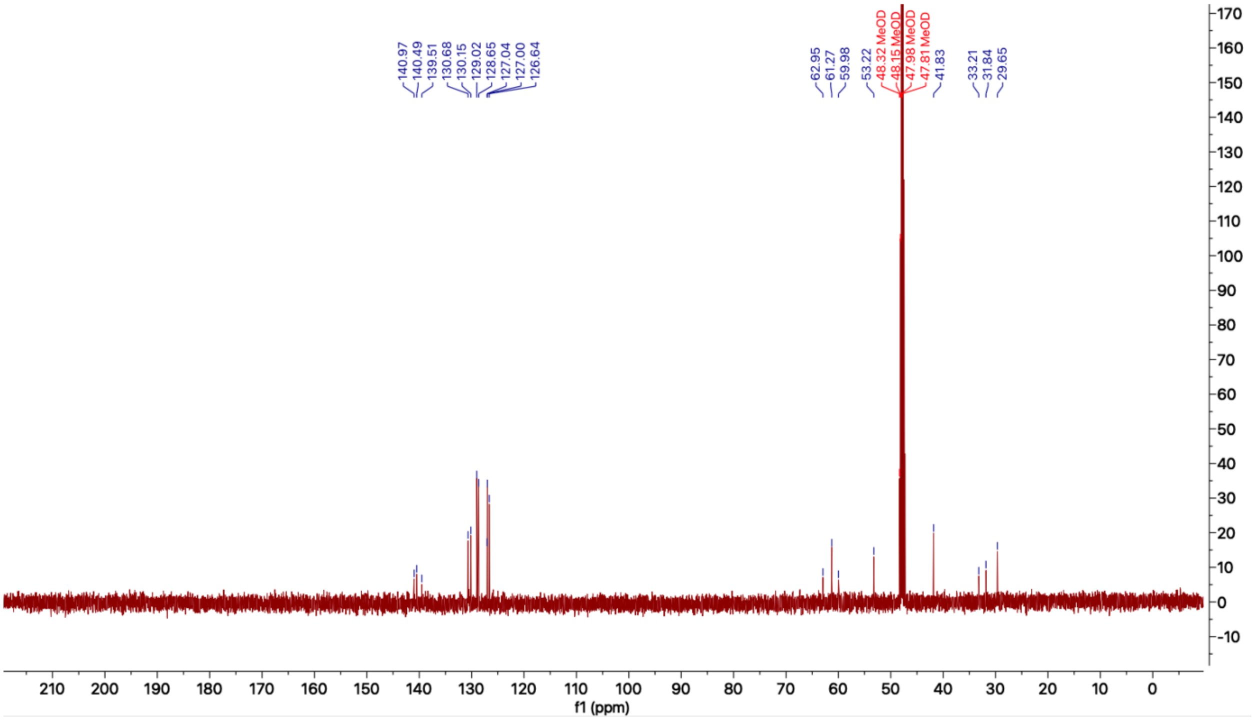
140.97
140.49
139.51
130.68
130.15
129.02
128.65
127.04
127.00
126.64
62.95
61.27
59.98
53.22
48.32 MeOD
48.15 MeOD
47.98 MeOD
47.81 MeOD
41.83
33.21
31.84
29.65
f1 (ppm)

1-(1-(2-([1,1'-Biphenyl]-4-yl)ethyl)piperidin-4-yl)-*N*-methyl-*N*-(naphthalen-2-ylmethyl)methanamine (NDM-593):

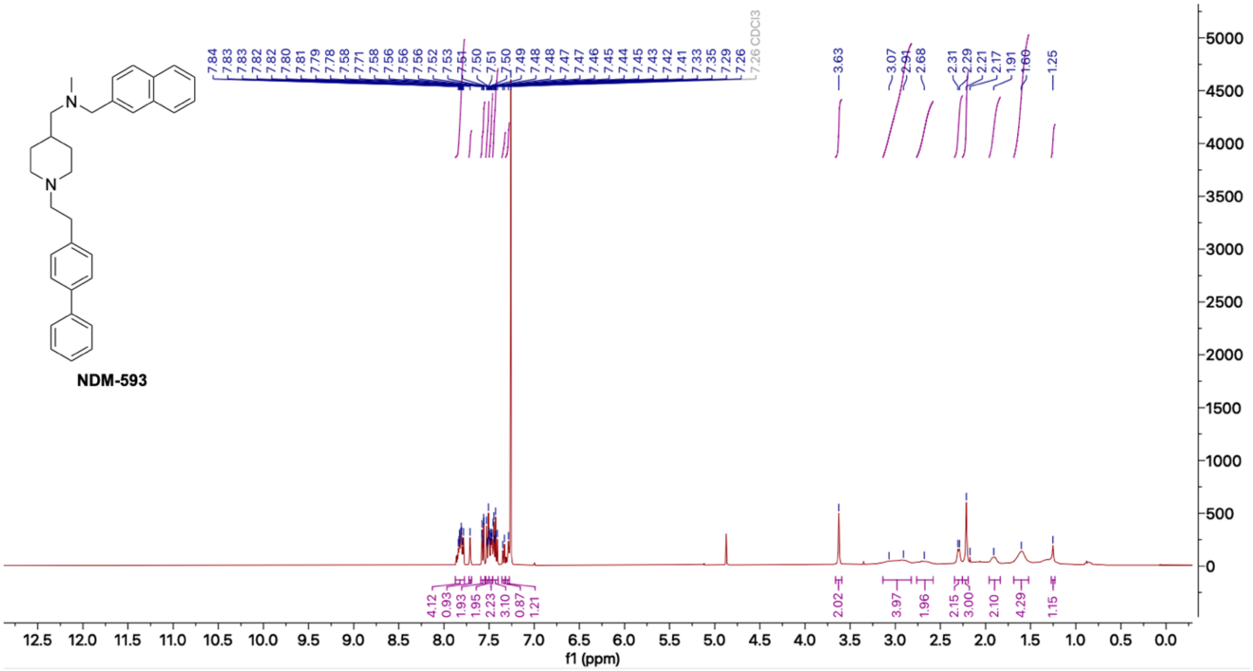

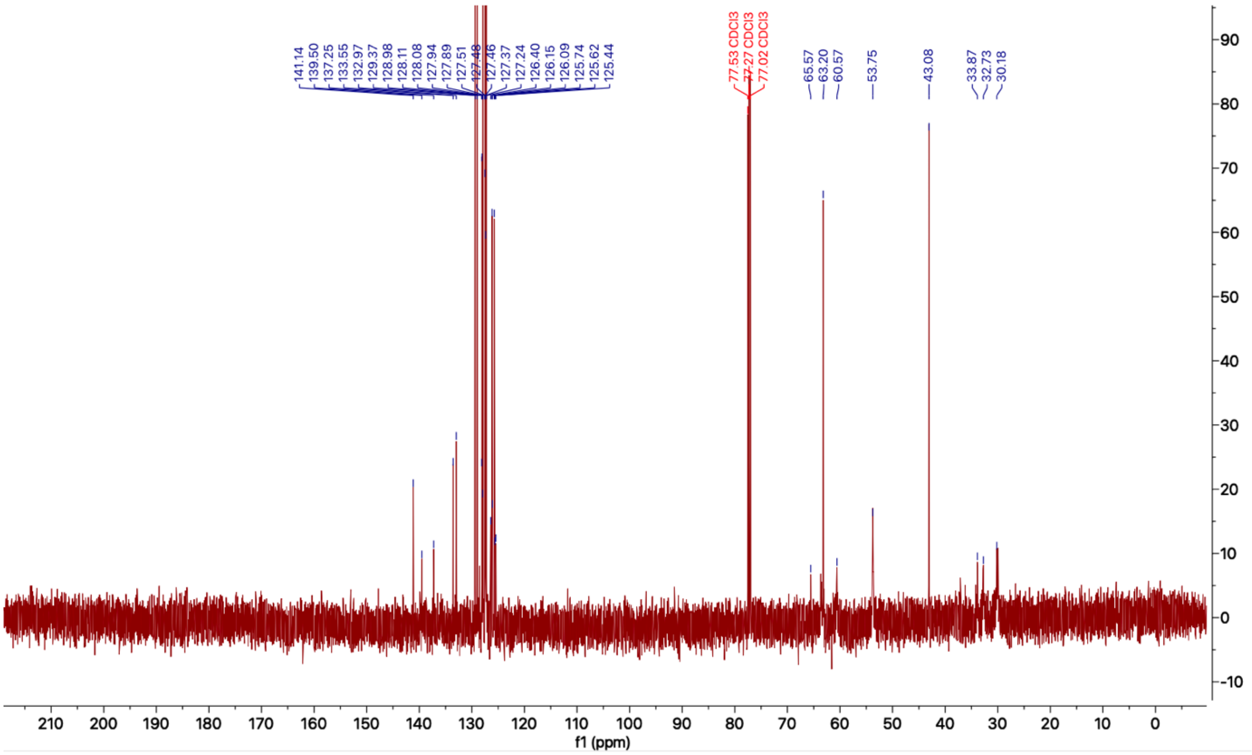
141.14
139.50
137.25
133.55
132.97
129.37
128.98
128.11
128.08
127.94
127.89
127.51
127.48
127.46
127.37
127.24
126.40
126.15
126.09
125.74
125.62
125.44
77.53 CDCl3
77.27 CDCl3
77.02 CDCl3
65.57
63.20
60.57
53.75
43.08
33.87
32.73
30.18
f1 (ppm)

References

(1) Sandala, J. L.; Eichar, B. W.; Kuo, L. G.; Hahn, M. M.; Basak, A. K.; Huggins, W. M.; Woolard, K.; Melander, C.; Gunn, J. S. A dual-therapy appraoch for the treatment of biofilm-mediated *Salmonella* gallbladder carriage. PLOS Pathogens, **2020**.

(2) Marrujo, S. A.; Hubble, V. B.; Yang, J.; Wang, M.; Nemeth, A. M.; Barlock, S. L.; Juarez, D.; Smith, R. D.; Melander, R. J.; Ernst, R. K.; et al. Dimeric 2-aminoimidazoles are highly active adjuvants for gram-postive selective antibiotics against *Acinetobacter baumannii* European Journal of Medicinal Chemistry: **2023**; Vol. 253.

(3) Woolard, K. J.; Sandala, J. L.; Melander, R. J.; Gunn, J. S.; Melander, C. Development of small molecules that work cooperatively with ciprofloxacin to clear salmonella biofilms in chronic gallbladder carriage model. European Journal of Medicinal Chemistry: **2022**; Vol. 232.

(4) Burke, E.; Welsh, E. N.; Robertsonb, K. N.; Speed, A. W. H. Efficient Synthesis and Functionalization of 3-Bromonaphtho[2,3-b]thiophene. Synthesis: **2023**; Vol. 55, pp 2406 - 2414.

(5) Recink, L.-M.; Thatcher, R. J.; Mallah, S.; Butts, C. P.; Collingridge, G. L.; Molnar, E.; Jane, D. E.; Willis, C. L. Synthesis and pharmacological characterisation of arctigenin analogues as antagonists of AMPA and kainate receptors. Organic & Biomolecular Chemistry: **2021**; Vol. 42.

(6) Attardo, G.; Tripathy, S. Preparation of methylsulfanylpyrimidine derivatives for use as antiinflammatories, analgesics, and antiepileptics. Canada **2010**.

(7) Kyo Chul Lee, S.-Y. L., Yearn Seong Choe, Dae Yoon Chi. Metabolic Stability of [18F]Fluoroalkylbiphenyls. *Bulletin of the Korean Chemical Society* **2004**, *25* (8), 1225-1230.

(8) Bennett, A. N., Laipply, B., & Gunn, J. S. (2024). Methods for detecting and monitoring Salmonella infection and chronic carriage in living mice using bioluminescent in vivo imaging. *Access microbiology, 6*(11), 000913.v3. https://doi.org/10.1099/acmi.0.000913.v3
